# Triple-junction solar cells with improved carrier and photon management

**DOI:** 10.1038/s41586-026-10385-y

**Published:** 2026-03-17

**Authors:** Kerem Artuk, Deniz Turkay, Austin Kuba, Stefan Riemelmoser, Julian A. Steele, Julien Hurni, Joël Spitznagel, Hugo Quest, Michele De Bastiani, Jun Zhao, Jonas Diekmann, Chiara Ongaro, Mostafa Othman, Maryamsadat Heydarian, Oliver Fischer, Huagui Lai, Jonathan S. Austin, Stefan Zeiske, Rafael López-Arteaga, Cheng Liu, Mounir D. Mensi, Andrés-Felipe Castro-Méndez, Muzhi Li, Thomas W. Gries, Siddha Hill, Felipe Saenz, Lisa Champault, Hilal Aybike Can, Mohammad Reza Golobostanfard, Umang Desai, Paul Remondeau, Eduardo Solano, Giuseppe Portale, Antonin Faes, Felix Lang, Artem Musiienko, Nicholas Rolston, Fan Fu, Martin C. Schubert, Florian Schindler, Bin Chen, Alfredo Pasquarello, Edward H. Sargent, Aïcha Hessler-Wyser, Quentin Jeangros, Christophe Ballif, Christian M. Wolff

**Affiliations:** 1 Photovoltaics and Thin Film Electronics Laboratory (PV-lab), Institute of Electrical and Micro Engineering (IEM), École Polytechnique Fédérale de Lausanne (EPFL), Neuchâtel, Switzerland; 2 Centre Suisse d’Electronique et de Microtechnique (CSEM), Neuchâtel, Switzerland; 3 Chaire de Simulation à l′Echelle Atomique (CSEA), École Polytechnique Fédérale de Lausanne (EPFL), Lausanne, Switzerland; 4 Australian Institute for Bioengineering and Nanotechnology (AIBN), The University of Queensland, Brisbane, Queensland Australia; 5 School of Mathematics and Physics, The University of Queensland, Brisbane, Queensland Australia; 6 3S Swiss Solar Solutions AG, Gwatt (Thun), Switzerland; 7 Fraunhofer Institute for Solar Energy Systems, Freiburg, Germany; 8 Chair for Photovoltaic Energy Conversion, Department of Sustainable Systems Engineering (INATECH), University of Freiburg, Freiburg, Germany; 9 Laboratory for Thin Films and Photovoltaics, Empa—Swiss Federal Laboratories for Materials Science and Technology, Dübendorf, Switzerland; 10 Department of Chemistry, Northwestern University, Evanston, IL USA; 11 Institute of Chemical Sciences and Engineering (ISIC), École Polytechnique Fédérale de Lausanne (EPFL), Sion, Switzerland; 12 Physik und Optoelektronik weicher Materie, Institut für Physik und Astronomie, Universität Potsdam, Potsdam, Germany; 13 Materials Science and Engineering, Fulton Schools of Engineering, Arizona State University, Tempe, AZ USA; 14 Young Investigator Group, Robotized Material and Photovoltaic Engineering, Helmholtz-Zentrum Berlin für Materialien und Energie (HZB), Berlin, Germany; 15 NCD-SWEET beamline at ALBA Synchrotron Light Source, Cerdanyola del Vallès, Spain; 16 Photophysics and OptoElectronics, Zernike Institute for Advanced Materials, University of Groningen, Groningen, The Netherlands; 17 Department of Electrical and Computer Engineering, Northwestern University, Evanston, IL USA

**Keywords:** Solar cells, Solar cells, Electrical and electronic engineering

## Abstract

Perovskite–silicon triple-junction photovoltaics offer efficiency gains beyond dual-junction devices but at the expense of added complexity^1^. Here we address two key bottlenecks in perovskite–silicon-based triple-junction solar cells: reduced open-circuit voltage (*V*_OC_) in the wide-bandgap (WBG) top cell and limited photocurrent generation in the middle cell^1,2^. A non-volatile additive, 4-hydroxybenzylamine (HBA), regulates WBG perovskite crystallization and passivates defects, promoting oriented growth and suppressing non-radiative recombination. Together with improved energy-level alignment, this yields *V*_OC_s of up to 1.405 V and enhanced stability. To overcome the current limitations in the middle cell, a three-step deposition strategy enables the formation of thick, low-bandgap perovskite absorbers while preserving microstructural integrity and enhancing electron extraction. Also, low-refractive-index SiO_*x*_-nanoparticles (SiO_*x*_-np) that accumulate in the front valleys of the textured silicon bottom cell act as an optical middle reflector, enhancing light absorption in the middle cell. These advances are then combined in 1-cm^2^ perovskite–perovskite–silicon devices, achieving a certified efficiency of 30.02%.

## Abstract (web summary)

The use of a non-volatile additive 4-hydroxybenzylamine to suppress non-radiative recombination, a three-step deposition strategy enabling thick lower-bandgap middle absorber, and low-refractive-index SiO_*x*_-nanoparticles acting as an optical middle reflector together enable the manufacture of perovskite–perovskite–silicon triple-junction solar cells with a certified efficiency of 30.02%.

## Main

Perovskite–silicon tandem solar cells have achieved efficiencies of up to 35%, approaching their practical target of 37.8% (refs. ^3,4^). Although the transition from single-junction to double-junction architecture reduces thermalization losses and enables more efficient use of the solar spectrum, triple-junction devices represent the next frontier, offering a theoretical efficiency limit of 51.8% (ref. ^5^). III–V-based triple-junction devices have demonstrated an efficiency of 39.5% (refs. ^3,6^). Such highly efficient triple-junctions based on III–V semiconductors entail very high fabrication costs and low throughput, making perovskite-based multijunctions a more attractive alternative.

The first perovskite–perovskite–silicon triple-junction device, reported in 2018, achieved an efficiency of 14.0% and a *V*_OC_ of 2.69 V (ref. ^7^). Recent triple-junction devices have reached 28.7% (ref. ^8^), still below state-of-the-art dual junctions (35%), mainly because of two limitations. First, WBG (>1.80 eV) perovskites exhibit high non-radiative losses, owing to limited bulk quality and suboptimal band alignment with the transport layers, and phase segregation, due to high bromide content^8,9^. Second, current matching is constrained by the >1.50 eV bandgap limit of lead-based perovskites, restricting middle-cell photocurrent. Additive and compositional strategies have partially addressed voltage losses^8–14^, yet recent triple junctions remain limited to about 3.08 V and 11.82 mA cm^−^^2^ in the middle cell^15^.

Here we address these limitations through coordinated carrier and photon management. Bulk defects in the WBG perovskite are suppressed using an arylamine-based 4-HBA additive that modulates crystallization, improves interfacial band alignment, reduces phase segregation and suppresses non-radiative recombination. To overcome current limitations in the middle cell, a three-step deposition strategy enables thick low-bandgap absorbers while preserving charge extraction and microstructural integrity. Photon management is enhanced using a low-refractive-index SiO_*x*_-np reflector that accumulates in submicron-textured silicon valleys, increasing middle-cell absorption and balancing sub-cell currents. Together, these strategies establish a transferable optoelectronic framework enabling triple-junction devices with stabilized efficiencies greater than 30%.

## Improving bulk quality of WBG perovskite absorbers

**Fig. 1:**
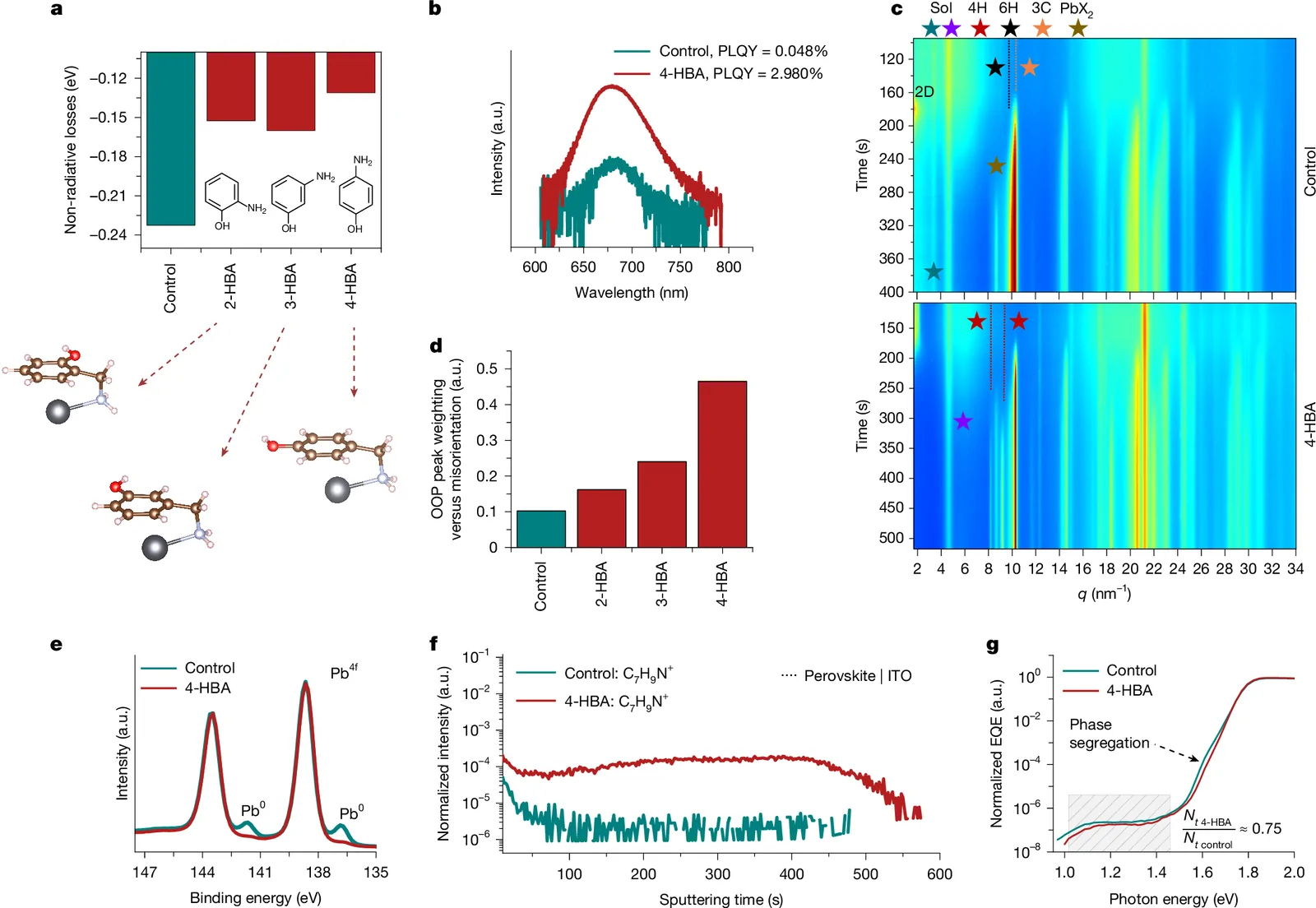
Improving the bulk quality of the WBG perovskite absorber. **a**, Non-radiative losses on half-cells (glass/ITO/Me-4PACz/SiO_*x*_-np (0.1%)) of the control absorber and absorber with various HBA additives. The molecular structures of these compounds and the adsorption of Pb^2+^ to 2-HBA, 3-HBA and 4-HBA are also shown^50^. **b**, Non-radiative losses on glass for the control absorber and the absorber with optimized 4-HBA (0.5 mg ml^−1^). **c**, Azimuthally integrated GIWAXS measurements as a function of time during spinning for control and 4-HBA-modified thin films. **d**, OOP peak weighting versus the misoriented fraction of (001) Bragg reflections, measuring the degree of the texture of the films for control and HBA derivatives. **e**, XPS results: Pb^4f^ spectra of the control and 4-HBA absorber. **f**, ToF-SIMS results of control and 4-HBA absorber on ITO/glass. **g**, High-dynamic-range EQE results for control and 4-HBA devices. a.u., arbitrary units.

The WBG perovskite absorber used in this work is a methylammonium-free Cs_0.22_FA_0.78_Pb(I_0.60_Br_0.30_Cl_0.10_)_3_ composition with a bandgap of 1.80 eV, selected for its enhanced phase stability compared with double-halide and triple-cation counterparts^14^. Non-radiative losses inferred from photoluminescence measurements on half-cells (glass/indium tin oxide (ITO)/(4-(3,6-dimethyl-9H-carbazol-9-yl)butyl)phosphonic acid (Me-4PACz)/SiO_*x*_-np (0.1%)) are >230 meV and >180 meV on glass substrates, respectively (Fig. 1a,b). These losses are notably higher compared with lower-bandgap analogues^16,17^. This suggests that the WBG perovskite exhibits poor bulk and interfacial quality (Fig. 1b).

To improve the bulk film quality, we applied additive engineering to modulate crystallization dynamics and defect formation^18^. Previous work has shown that phenylalkylamines can passivate Pb-halide perovskites, with benzylamine exhibiting particularly effective surface passivation, attributed to favourable molecular stacking interactions^19–21^. Here we explored a wide range of benzylamine derivatives as an additive in the WBG perovskite ink^21^. We tracked changes in non-radiative losses after the deposition of the perovskite (with different benzylamine-based compounds added in the perovskite ink) on a half-cell stack (Supplementary Fig. 1). With three positional isomers of HBA, non-radiative losses decreased from 230 meV to less than 150 meV (Fig. 1a). Among the positional isomers, 4-HBA provided the highest passivation quality, reducing non-radiative losses to about 130 meV. We then optimized the 4-HBA concentration on half-cell stacks, reaching photoluminescence quantum yields (PLQYs) of up to 2.98% on glass (corresponding to a non-radiative loss of about 91 meV) and 0.60% on half-cells (corresponding to a non-radiative loss of about 132 meV) (Fig. 1a,b and Supplementary Fig. 2).

To assess the chemical stability of the HBA additives in the perovskite precursor solution, we performed nuclear magnetic resonance (NMR) analysis on fresh and aged inks. No peak shifts or further resonances associated with HBA derivatives were observed, indicating that the additives remain chemically intact in the precursor solution. After one week of storage, only minor changes in the FAI–NH_2_ signal were detected, which were also present in the reference ink without HBA. The non-exchangeable aromatic resonances of the HBAs remained unchanged, confirming chemical stability during storage (Supplementary Figs. 3–5 and Supplementary Note 1).

To explain the impact of HBA derivatives on precursor chemistry and film formation, we performed density functional theory (DFT) calculations. The calculations show that HBA strongly interacts with Pb^2^^+^ by means of a Pb–N bond, accompanied by out-of-plane (OOP) distortion of the otherwise planar molecule (Fig. 1a), consistent with effective defect passivation. The calculated adsorption energy is 6.8 eV, comparable with the value of 6.4 eV reported in ref. ^21^ for 4-(methoxy)benzylamine bromide. FA^+^ and Cs^+^ can occupy the same binding site but interact weakly (1.6 and 1.2 eV, respectively). To investigate nucleation effects, we examined HBA binding to a simplified CsPbI_3_ model, which forms Cs–N bonds at the same cationic site (Supplementary Fig. 6). For the positional isomers, 2-HBA exhibits enhanced interaction (1.2 eV) relative to 3-HBA (1.0 eV) and 4-HBA (0.9 eV) (Supplementary Table 1), probably because of the proximity of the OH group. The slightly higher interaction energy of 2-HBA with CsPbI_3_ may therefore promote faster reaction kinetics compared with 4-HBA.

To ascertain whether HBA influences crystallization dynamics, we performed in situ grazing incidence wide-angle X-ray scattering (GIWAXS) during spin-coating and annealing (Fig. 1c, Supplementary Figs. 7–10 and Supplementary Note 2). Compared with the control, HBA-modified films exhibit altered intermediate-phase evolution and slower 6H perovskite formation kinetics. 4-HBA results in the slowest process among the three positional isomers, which is then inversely correlated with the adsorption energy calculated from DFT (Supplementary Table 1) to the perovskite building block, CsPbI_3_. In fact, the ‘slowest’ isomer also achieves the lowest non-radiative losses, indicating the importance of crystallization dynamics to the resultant film quality. This moderated crystallization promotes improved halide homogenization and enhanced structural ordering. Ex situ GIWAXS confirms increased OOP texture and reduced phase heterogeneity for 4-HBA films (Fig. 1d and Supplementary Fig. 11), accompanied by a slight lattice contraction (Supplementary Fig. 12). These structural improvements correlate with enlarged domains and reduced horizontal domain boundaries (Supplementary Fig. 13), consistent with the reduced non-radiative losses observed in Fig. 1b.

Films processed with 0.5 mg ml^−^^1^ 4-HBA are hereafter defined as the target condition. Surface chemistry and energetics were examined by X-ray photoelectron spectroscopy (XPS) and ultraviolet photoelectron spectroscopy (UPS). Incorporation of 4-HBA suppresses metallic Pb^0^ formation, an active defect in contact with C_60_ (ref. ^22^) (Fig. 1e), consistent with strong Pb^2+^–4-HBA coordination that inhibits Pb^2+^ reduction and provides chemical passivation. The O_1s_ spectrum of target films shows a distinct –OH signal absent in controls (Supplementary Fig. 14), confirming the presence of 4-HBA at the surface. UPS reveals a valence band maximum shift from −6.39 eV to −6.19 eV following 4-HBA incorporation (Supplementary Figs. 15 and 16), improving alignment at the ITO/Me-4PACz hole contact (*E*_VBM_ at −5.75 eV) and reducing voltage losses^23^.

Time-of-flight secondary ion mass spectrometry (ToF-SIMS) (C_7_H_9_N^+^ fragment) shows that 4-HBA is distributed throughout the absorber thickness (Fig. 1f), in contrast to phosphonic-acid-based passivating additives, which accumulate at interfaces^16^. Increasing the 4-HBA concentration threefold does not substantially alter its depth distribution (Supplementary Fig. 17) and halide distribution remains unchanged (Supplementary Fig. 18).

High-dynamic-range external quantum efficiency (EQE) measurements on WBG single-junction devices (Supplementary Note 3) show a reduced band tail near 1.6 eV in 4-HBA-treated devices, consistent with suppressed phase segregation. Both devices exhibit sub-bandgap absorption; given comparable thicknesses (approximately 350 nm versus approximately 325 nm), differences in subgap EQE are attributed to reduced trap-state density rather than optical interference. Integration of the subgap region indicates roughly 25% fewer optically active trap states with 4-HBA incorporation (Fig. 1g). Low-fluence transient absorption further shows slower ground-state bleach recovery and reduced spectral shifts, consistent with lower non-radiative recombination and diminished phase segregation (Supplementary Figs. 19 and 20).

**Fig. 2:**
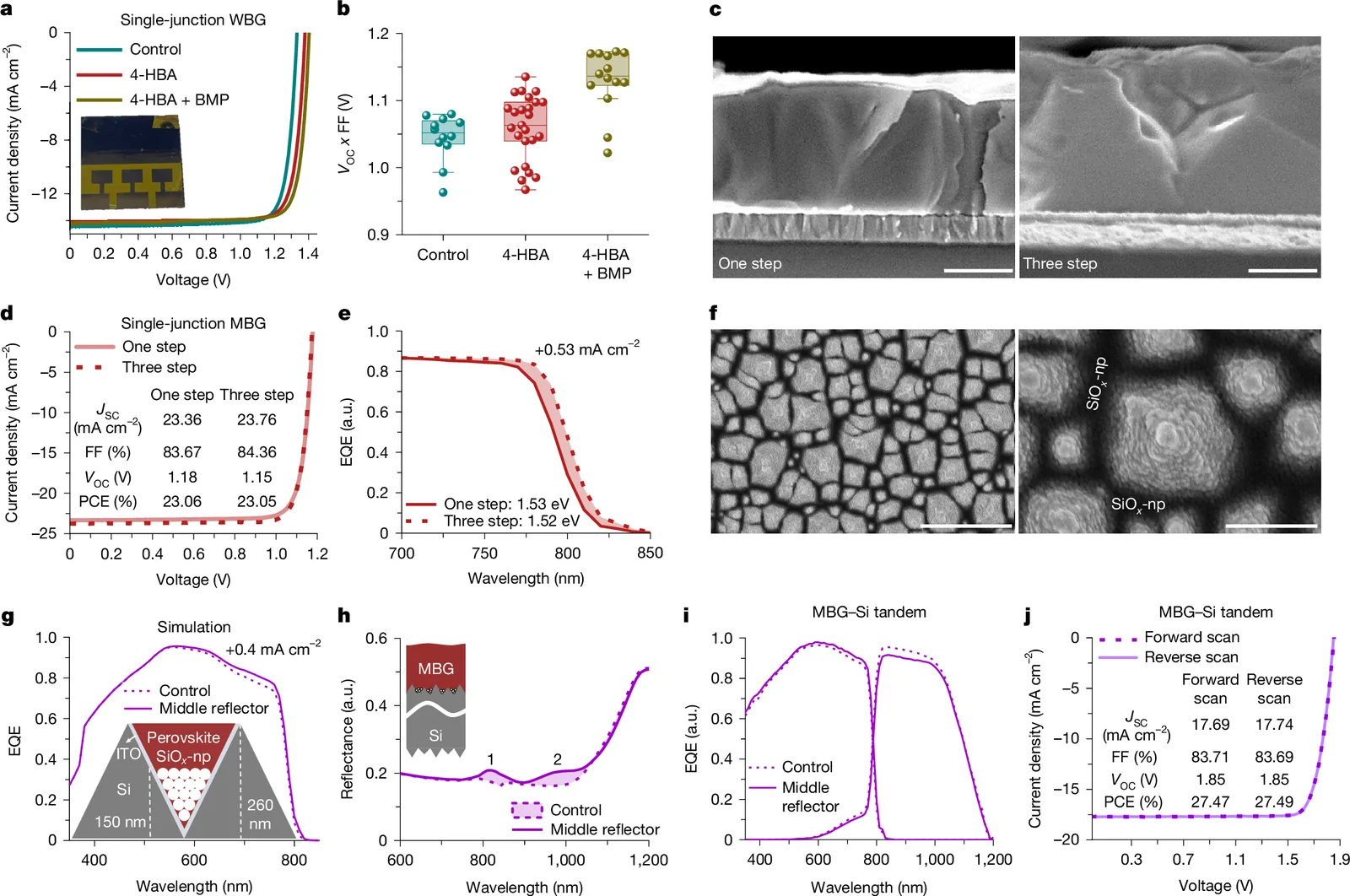
Photon management to increase the current density in the middle cell. **a**, Champion reverse-scan *J*–*V* curves of the opaque WBG single-junction perovskite solar cells with control, 4-HBA and 4-HBA combined with BMP. **b**, *V*_OC_ *x* FF statistics of WBG single-junction opaque perovskite solar cells with control, 4-HBA and 4-HBA combined with BMP. **c**, Cross-section SEM images of one-step and three-step absorbers. Scale bars, 400 nm **d**, Champion reverse-scan *J*–*V* curve of MBG single-junction opaque perovskite solar cells with one-step and three-step absorbers. **e**, EQE of MBG single-junction opaque perovskite solar cells with one-step and three-step absorbers. **f**, Top-view SEM images of submicron textured Si bottom cells with SiO_*x*_-np coating (0.5%). Scale bars, 1,200 nm (left); 400 nm (right). **g**, EQE results from finite-element simulation of a stack with and without a middle reflector (with 600-nm absorber). **h**, Reflectance spectrum of control (perovskite/Si) and the middle reflector (perovskite/SiO_*x*_-np/Si). **i**, EQE of MBG–Si tandem devices with and without a middle reflector (with 600-nm one-step absorbers). **j**, Forward-scan and reverse-scan *J*–*V* curves of a MBG–Si tandem with a middle reflector and three-step absorbers. a.u., arbitrary units.

We then fabricated WBG perovskite single-junction devices with and without 4-HBA with optimized contacts (Supplementary Note 4). As shown in Supplementary Fig. 22, 4-HBA increased *V*_OC_ by about 30 mV, with slight fill factor (FF) and short-circuit current (*J*_SC_) reductions owing to decreased ink molarity. The best WBG device achieved a *V*_OC_ of 1.38 V and FF of 83.38% (Fig. 2a). When 4-HBA WBG absorbers are combined with bimolecular interface passivation, *V*_OC_ and FF improved to 1.405 V and 83.27% without markedly affecting *J*_SC_ (Fig. 2a,b). The universality of 4-HBA, serving as a general molecular modulator of crystallization kinetics and defect chemistry in Pb-perovskite absorbers, was further verified across compositions with different bandgaps in mixed and pure halide systems (1.55 eV, 1.65 eV, 1.68 eV and 1.90 eV; Supplementary Figs. 23 and 24 and Supplementary Note 5).

Taken together, DFT, NMR, GIWAXS and spectroscopic analyses indicate that 4-HBA coordinates with Pb^2+^ in the precursor solution without forming secondary phases, thereby maintaining ink stability while moderating crystallization kinetics. This interaction is consistent with transient stabilization of precursor species and delayed nucleation, resulting in improved film microstructure and reduced defect density. ToF-SIMS measurements show that 4-HBA is distributed throughout the absorber layer, supporting bulk passivation rather than interface-localized modification. Across several compositions, the incorporation of 4-HBA consistently reduces non-radiative recombination losses.

After optimization of the isolated top cell, we now discuss improvements related to the medium-bandgap (MBG) perovskite–silicon tandems that serve as the foundation for the top cell.

## Increasing current density in middle cell

### Hybrid on one-step (three-step) absorbers

Increasing absorber thickness while reducing bandgap without compromising transport is challenging in solution-processed perovskites^24^. We implement a hybrid three-step deposition combining evaporated PbI_2_ and FAI-rich conversion on a one-step absorber^20,25–28^, enabling bandgap reduction and thickness increase while preserving charge extraction. The resulting FAPbI_3_-rich layer incorporates Cs^+^/MA^+^ for phase stability^29^ (Methods, Supplementary Fig. 35 and Supplementary Notes 6 and 7). Cross-sectional scanning electron microscopy (SEM) confirms full intermixing of one-step and hybrid layers, forming a smooth, thickened absorber (Fig. 2c). ToF-SIMS depth profiling shows uniform Cs^+^, Pb^+^, I^+^, FA^+^ and MA^+^ distributions with no vertical gradients (Supplementary Figs. 38–40), ruling out graded junction formation (Supplementary Note 8).

We next investigated the mechanical adhesion of one-step and three-step absorbers on ITO–self-assembled monolayer (SAM) substrates. Three-step-processed films exhibit substantially higher fracture energies than their one-step counterparts, with increases to greater than 1 J m^−^^2^ on (4-(9′-phenyl-9H,9′H-[3,3′-bicarbazol]-9-yl)butyl)phosphonic acid (4PABCz)^30^, a threshold above which materials are generally considered to be mechanically robust^31^ (Supplementary Fig. 33). In line with this trend, three-step absorbers show a fracture path that has a much lower fraction of perovskite delamination from the Si substrate relative to one-step films, indicating markedly stronger bonding at the SAM–perovskite interface (Supplementary Fig. 33e).

The X-ray diffractograms of one-step and three-step films show similar phase composition and texture (Supplementary Fig. 41), with increased peak intensities in the three-step absorber owing to its greater thickness. A weak diffraction feature at 2*θ* ≈ 33° appears only in the three-step film and is confirmed by GIWAXS (Supplementary Fig. 42). Such nominally forbidden reflections are attributed to subtle octahedral tilting in FA-based perovskites, associated with suppression of performance-limiting 4H/6H polytypes^32^.

Three-step deposition increases the thickness of the MBG by more than 250 nm (Fig. 2c) and the current densities by about 0.50 mA cm^−2^ in single-junction opaque devices (Fig. 2d,e). In single junctions on ohmic textured-Si substrates and illuminated from the electron transport layer side, the gain in *J*_SC_ reaches up to about 0.90 mA cm^−2^ (see Supplementary Fig. 43 for EQE). The champion device performance of opaque devices with the one-step (control) and three-step (target) absorbers can be seen in Fig. 2d. The thicker three-step absorber maintained high FF (84.36%), similar to the one-step baseline. The preservation of high FFs indicates efficient charge extraction throughout the three-step absorber. Transient surface photovoltage measurements^33,34^ revealed faster and higher-amplitude electron extraction at the C_60_ interface for three-step films (Supplementary Fig. 37), consistent with enhanced carrier generation and indicate reduced non-radiative recombination at the electron transport layer interface. At the same time, we observed a reduction in hole extraction (Supplementary Fig. 37), attributed to increased transport distances in thicker absorbers rather than interfacial trapping or limited diffusion lengths^35^.

In single-junction devices illuminated from the hole transport layer side, the *V*_OC_ decreases by about 30 mV, consistent with reduced transient surface photovoltage amplitude and a slight increase in non-radiative losses (approximately 11 meV; Supplementary Fig. 44) alongside a modest bandgap shift from 1.53 eV to 1.52 eV in the FAPbI_3_-rich hybrid absorber. We further compared the optoelectronic performance of one-step and three-step absorbers in a MBG–Si tandem configuration (Supplementary Fig. 45). The three-step absorber produces an extra 0.80 mA cm^−^^2^ in current density in the middle cell and shows a slightly higher average *V*_OC_ (consistent with transient surface photovoltage measurements; Supplementary Fig. 37); see Supplementary Fig. 46.

### Middle reflector

To enhance middle-cell current density, we introduced a low-refractive-index SiO_*x*_-np middle reflector (*n*_800__nm_ ≈ 1.33) at the perovskite–silicon interface (Fig. 2f). The resulting refractive-index contrast reflects part of the transmitted light back into the middle cell, increasing absorption near its band edge. Similar index-contrast reflector concepts have been applied in perovskite–silicon and micromorph multijunction architectures^36–43^. The SiO_*x*_-np preferentially accumulate in the valleys of the textured silicon, maintaining electrical contact at the pyramid tips.

First, we simulated the influence on EQE of varying amounts of SiO_*x*_-np within the valleys of submicron Si pyramids (Supplementary Fig. 47a). As the filling of the valleys increases, the absorption near the band edge increases (600–850 nm range), contributing to higher current densities by 0.40 mA cm^−^^2^ (Fig. 2g). We varied the SiO_*x*_-np concentration (0.25, 0.5 and 1 wt%) to confirm the simulation results. Top-view SEM images of three different conditions can be seen in Supplementary Fig. 48, in which the pyramid valleys covered with SiO_*x*_-np appear as dark regions in the SEM images (see Supplementary Fig. 49 for filling height estimations).

The reflectance spectra of double-sided submicron textured Si coated with perovskite, with and without the SiO_*x*_-np middle reflector, are shown in Fig. 2h. Two distinct spectral regions exhibit increased reflectance when the SiO_*x*_-np layer is introduced. Region 1 (≈775–900 nm) corresponds to wavelengths just beyond the perovskite band edge and is directly associated with enhanced back-reflection into the middle cell, thereby boosting the band-edge response. Region 2 (≈900–1,050 nm) encompasses wavelengths transmitted towards the bottom Si sub-cell; here the increased reflectance reduces the photon flux entering the Si. These spectral changes validate the formation of the intended perovskite/SiO_*x*_-np middle reflector arising from the refractive-index decrease (from 2.5 down to 1.33) at this interface (Supplementary Fig. 47b). These observations are confirmed at the EQE level by fabricating MBG–Si tandems: as the SiO_*x*_-np concentration increases, the EQE of the MBG sub-cell increases (Supplementary Fig. 50 and Fig. 2i) and the EQE of the bottom cell decreases over the 900–1,050-nm range.

A representative MBG–Si tandem with the middle reflector achieves a FF of 83.69% and a *V*_OC_ of 1.85 V, along with a power conversion efficiency (PCE) close to 28% (Fig. 2j). We observe that the FF of the 1% SiO_*x*_-np condition is 2%_abs_ lower than the rest of the conditions owing to increasing resistive losses (*R*_s_ extracted around *V*_OC_ increases by 1 Ω cm^2^) (Supplementary Figs. 51 and 52). The origin of this loss is probably the decreasing contact area between the perovskite and the hole transport layer^16^. The 0.25% to 1% SiO_*x*_-np concentration range hence seems optimal, as it leads to the best *J*_SC_ and FF trade-off. The champion EQE of MBG–Si tandems, without and with a middle reflector (1 wt%), are shown in Fig. 2i. The integrated current density from the EQE spectrum of the middle cell reaches 25.1 mA cm^−^^2^ (Supplementary Fig. 53), which exceeds previously reported values for this type of semitransparent device (for comparison, Liu et al. reported a value of 23.85 mA cm^−^^2^)^12^.

With the middle reflector, the current density increases in the middle cell and decreases in the Si bottom cell, hence promising a better current distribution in the triple-junction device. Thus, the SiO_*x*_-np middle reflector is not an isolated optical enhancement but a deliberately balanced optoelectronic element whose net benefit emerges at the triple-junction level. When combined with the three-step-processed absorber, our model predicts a triple-junction PCE greater than 31% (Supplementary Table 2).

### Triple-junction integration

Having established a high-*V*_OC_ WBG top cell and a thickened, low-bandgap middle absorber with optimized optics, we integrate these components into monolithic perovskite–perovskite–silicon triple-junction devices. The bottom-cell architecture used in this study is based on a silicon heterojunction design, initially using a front-flat/rear-textured configuration during early development stages, followed by a transition to a double-sided submicron textured configuration^44,45^.

**Fig. 3:**
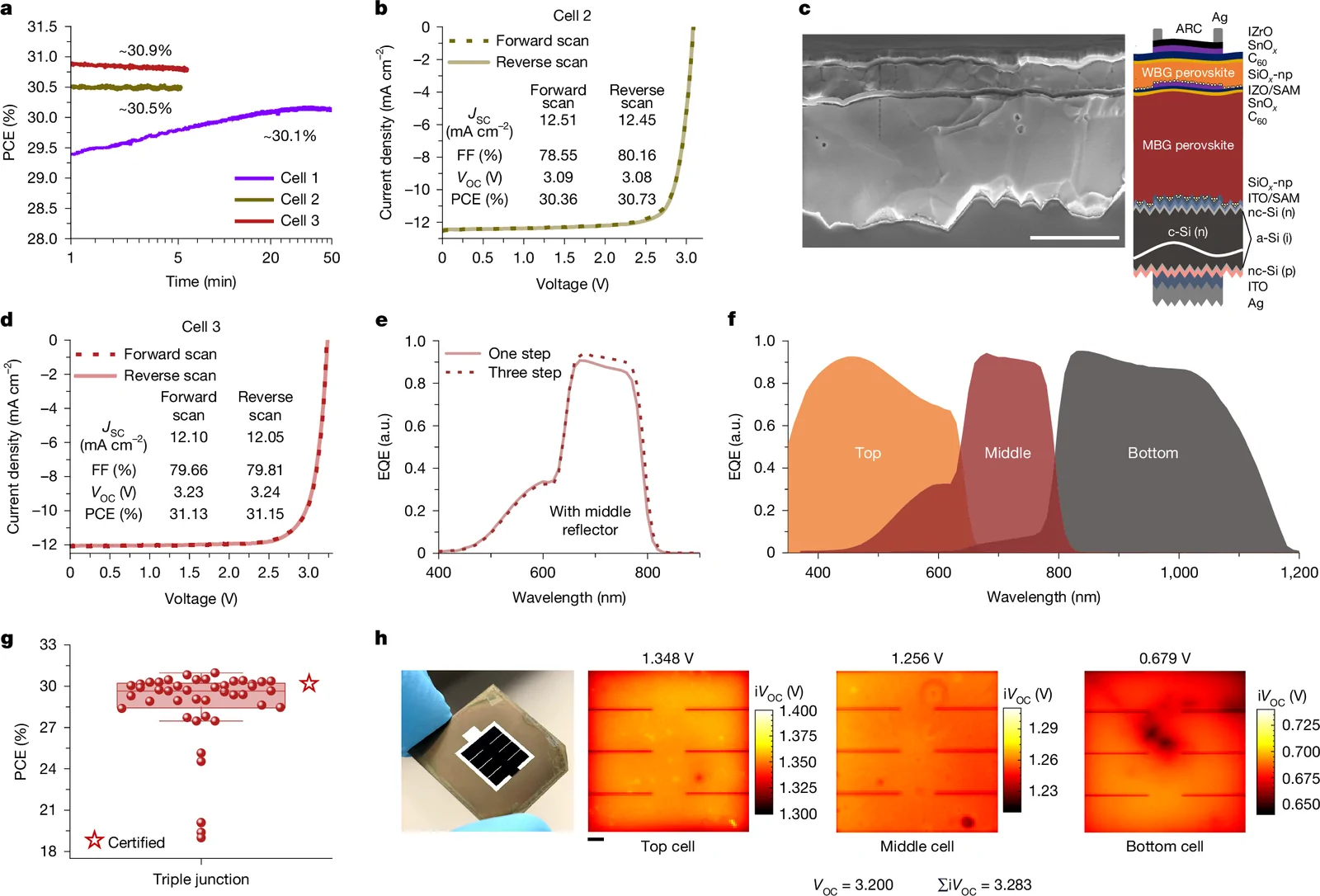
Triple-junction integration. **a**, MPP tracking of the best three triple-junction devices from three different batches, cell 1, cell 2 and cell 3. **b**, Forward-scan and reverse-scan *J*–*V* curves of the triple-junction device labelled as cell 2. **c**, Cross-section SEM image of a triple-junction device with the middle reflector, three-step middle cell and device schematic view. Scale bar, 1 μm. **d**, Forward-scan and reverse-scan *J*–*V* curves of the champion triple-junction device, labelled as cell 3. **e**, EQE of triple-junction middle cell with the middle reflector, with either a one-step or a three-step MBG absorber. **f**, EQE of a sister device of the champion triple-junction device. **g**, Device efficiency statistics from the champion batch of 22 devices with an average efficiency of 29.65% (both forward-scan and reverse-scan data points reported) and the certified value of 30.44%. **h**, Optical image of a triple-junction device and i*V*_OC_ imaging of a sister device of the champion triple-junction device. Scale bar, 0.1 cm. a.u., arbitrary units. Source data

Triple-junction integration started from a front-flat architecture with a one-step middle absorber towards progressively optimized configurations (Supplementary Notes 9 and 10). Incorporation of 4-HBA improved voltage and device yield but revealed current mismatch between WBG and MBG sub-cells. We then proceeded with double-sided submicron textured substrates (Supplementary Notes 9 and 10). Bandgap tuning of the top cell and implementation of the three-step middle absorber improved current balance, yielding >1% absolute efficiency gains (Supplementary Figs. 59–62). A switch to textured substrates and introduction of a SiO_*x*_-np middle reflector further enhanced middle-cell absorption, enabling maximum power point (MPP) efficiencies approaching 30% (Supplementary Fig. 63 and Fig. 3a, cell 1). Subsequent transport-layer optimization and current-matching refinement resulted in stabilized efficiencies exceeding 30% (Fig. 3a,b, cell 2). Cross-sectional SEM images of representative triple-junction devices fabricated on nanotextured substrates and a schematic are presented in Fig. 3c and Supplementary Figs. 62 and 67.

Incorporation of 4PABCz into the hole-selective layers mitigated light-soaking and transient effects that previously limited the *V*_OC_, enabling recovery of the full sub-cell voltage sum. The champion device (cell 3) achieved a reverse-scan efficiency of 31.15% with a *V*_OC_ of 3.24 V and a stabilized efficiency of 30.9% (Fig. 3a,d). Batch statistics yielded an average efficiency of 29.65% and average *V*_OC_ of 3.245 V (Supplementary Fig. 68), indicating repeatable performance. The three-step absorber further increased the middle-cell current density from 11.90 to 12.42 mA cm^−^^2^, as confirmed by EQE measurements (Fig. 3e,f and Supplementary Fig. 69).

To validate device performance, a batch of triple-junction devices was measured at Fraunhofer Institute for Solar Energy Systems, yielding efficiencies consistent with in-house measurements (Supplementary Table 3 and Supplementary Figs. 59 and 70). Later, two devices (cell 3 and a sister cell) were subsequently submitted for certification and achieved stabilized certified efficiencies of 30.02% and 29.76%, with reverse-scan efficiencies of 30.44% and 30.18% and *V*_OC_s exceeding 3.2 V (Supplementary Figs. 71–73). The comparison between in-house and certified measurements is shown in Supplementary Fig. 73 (Supplementary Notes 11 and 12). These results represent an absolute efficiency improvement of 3.3%_abs_ over the certified triple-junction device previously reported^12^.

On 1-cm^2^ cells, we conducted sub-cell-selective i*V*_OC_ imaging^46,47^ (Fig. 3g). On the one hand, the top and middle cells show high levels of homogeneity and average i*V*_OC_ values of 1.348 V and 1.256 V, respectively. On the other hand, the Si bottom cell performs on average rather poorly (i*V*_OC_ = 0.679 V), with detrimental low-photoluminescence areas, attributed to the bottom-cell damage during perovskite processing^29^. The i*V*_OC_ of the three-step middle cell is particularly high and homogeneous. Furthermore, we calculate a roughly 80-mV difference between the *V*_OC_ measured at the terminals of the cell and the sum of the sub-cells’ i*V*_OC_, which is attributed to selectivity losses^48^.

To assess scalability, we fabricated 4-cm^2^ and 54-cm^2^ devices on 4-inch substrates using fully wafer-scale processing, including screen-printed electrodes and an industrially relevant bottom-cell architecture (Supplementary Figs. 74–76 and Supplementary Note 13). Despite the thermal and mechanical constraints of large-area processing, the devices achieved efficiencies exceeding 27% (4-cm^2^ device) and 26% (54-cm^2^ device), demonstrating compatibility with wafer-scale manufacturing.

### Device stability

**Fig. 4:**
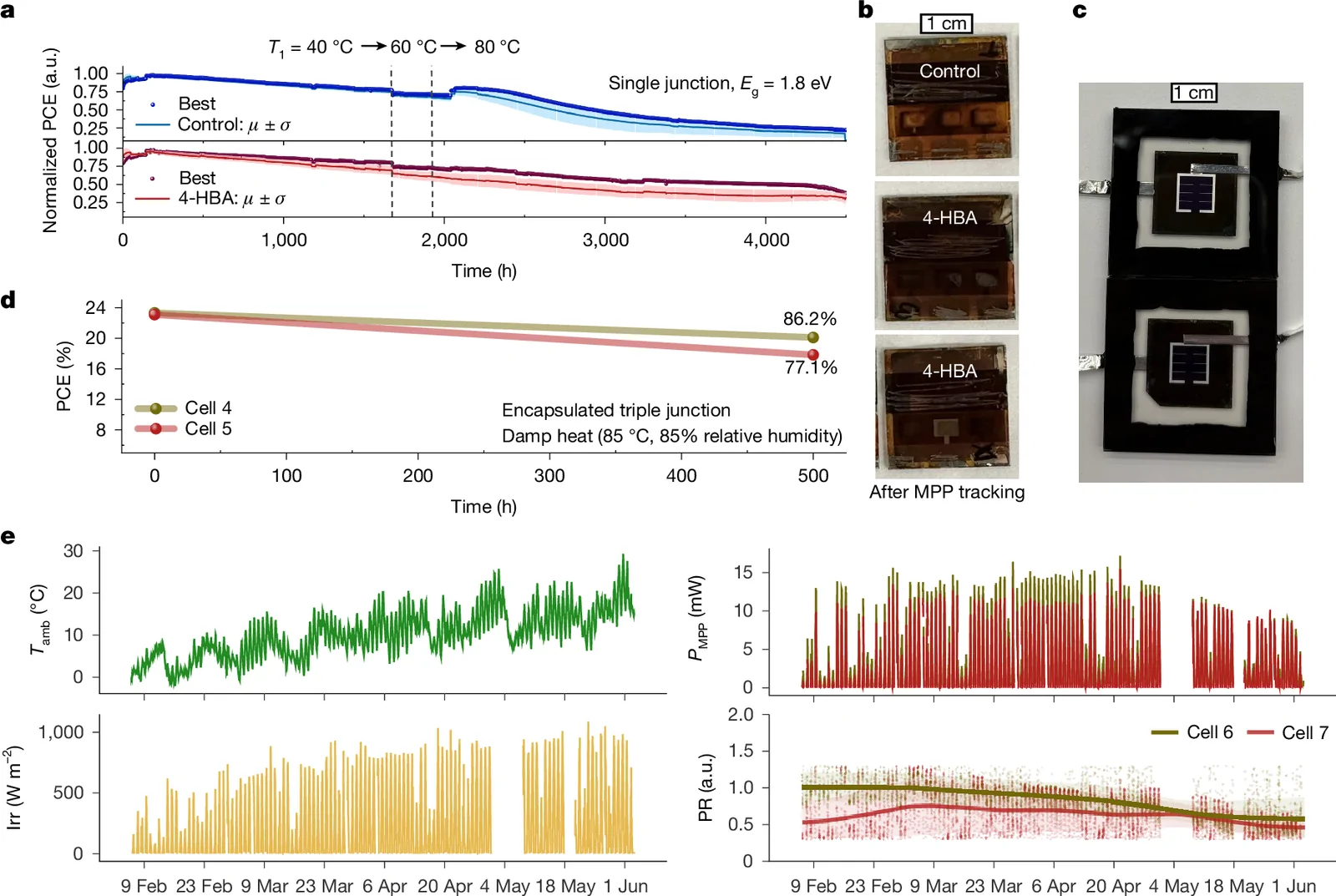
Device stability. **a**, MPP tracking on unencapsulated WBG single-junction cells with and without 4-HBA in N_2_ with temperatures ramping up from 40 °C to 60 °C and 80 °C. **b**, Optical images of single-junction cells after MPP tracking. **c**, Optical images of encapsulated triple-junction devices, cell 4 and cell 5. **d**, Damp heat testing of two encapsulated triple-junction devices, cell 4 and cell 5. **e**, Outdoor testing results for two encapsulated triple-junction devices, cell 6 and cell 7 (ambient temperature (*T*_amb_), power output (*P*_MPP_), irradiance (Irr) and performance ratio (PR); see Methods for calculation details of the performance ratio). Measurements were interrupted from 1 May to 8 May 2025 and again from 18 May to 20 May 2025 owing to a technical issue. a.u., arbitrary units.

The operational stability of unencapsulated WBG single-junction devices (with and without 4-HBA) was evaluated under MPP tracking in N_2_ at progressively elevated temperatures up to 80 °C for a total of 4,500 h (Fig. 4a, Supplementary Fig. 77 and Supplementary Note 14). Although both control and 4-HBA devices retained approximately 70% of their initial efficiency after about 2,000 h, control devices subsequently exhibited accelerated degradation, resulting in a final PCE retention of 24.3% (best case) after 4,500 h. By contrast, 4-HBA devices showed slower degradation kinetics, retaining up to 39.5% of their initial efficiency. Optical inspection revealed more pronounced formation of PbI_2_-rich degradation phases in the control devices (Fig. 4b), consistent with the lower Pb^0^ content observed by XPS in 4-HBA-modified films (Fig. 1e).

Encapsulated triple-junction devices (glass/glass) were further subjected to damp-heat testing (85 °C/85% relative humidity). After 500 h, devices exhibited relative PCE losses of 13.8% and 22.9% (Fig. 4d and Supplementary Fig. 78). Outdoor testing in Neuchâtel, Switzerland, over four months showed stable performance ratios during low-temperature operation, with moderate reductions at elevated temperatures for cell-6 but no visible degradation (Fig. 4e and Supplementary Figs. 79 and 80).

The stability demonstrated here provides an initial benchmark for perovskite–silicon triple-junction devices; however, extended damp heat, thermal cycling^49^, outdoor monitoring and high-temperature light-operational testing will be required to meet industry qualification standards.

## Conclusions

We report a perovskite–perovskite–silicon triple-junction solar cell achieving a certified stabilized efficiency of 30.02% through coordinated carrier and photon management. Non-radiative recombination in the WBG top cell is suppressed through additive-controlled crystallization, whereas a three-step deposition strategy enables thick, lower-bandgap middle absorbers without compromising structural or ionic stability. A SiO_*x*_-np middle reflector improves current balance across sub-cells. Together, these design principles establish a transferable framework for optoelectronic optimization in perovskite–silicon multijunction devices, with demonstrated stability and wafer-scale compatibility.

## Methods

### Perovskite fabrication and deposition

All organohalide precursors were purchased from Dyenamo. Lead halide precursors were obtained from TCI and CsI was supplied by Alfa Aesar. PDAI_2_ was purchased from TCI. SAMs were supplied by Dyenamo, except for 4PABCz, which was obtained from Suzhou LiWei Tech Co., Ltd. 4F-PEACl was purchased from GreatCell Solar and 2-HBA, 3-HBA and 4-HBA were obtained from Sigma-Aldrich. All solvents were purchased from Sigma-Aldrich.

#### WBG absorber

The WBG absorber used in this work was Cs_0.__22_FA_0.__78_Pb(I_0.__60_Br_0.__30_Cl_0.__10_)_3_. The bandgap was tuned by adjusting the Br ratio between 30% and 40%. The chloride amount was fixed at 10%. By adjusting the molarity between 1 M and 1.2 M, the thickness was also tuned. The 1 M perovskite solution was prepared by mixing 0.707 M PbI_2_, 0.1 M PbCl_2_, 0.3 M PbBr_2_, 0.1 M MACl, 0.48 M FAI, 0.3 M FABr and 0.22 M CsI in DMF:DMSO 3:1 (volume ratio). The solution was shaken for at least 1 h to make sure the precursors were dissolved. Then additives from the stock solution (for example, 10 mg ml^−1^ in DMF:DMSO) were added into the perovskite ink and shaken for at least 15 min. The whole solution was filtered using 0.22-μm PTFE filters. The absorber was fabricated with the one-step antisolvent method. The precursor solution (100 µl) was spread onto the substrate (2.5 × 2.5 cm^2^) and then spun at 5,000 rpm with a rate of 1,000 rpm s^−1^ for 32 s. When 10 s remained during the spin programme, 200 µl of anisole was dropped onto the substrate. Then the samples were annealed at 100 °C for 15 min inside the glovebox.

#### MBG absorber: one step

The MBG absorber used in this work was Cs_0.__05_FA_0.__9_MA_0__.05_PbI_3_ and 5% excess MAPbCl_3_. The solution was prepared by mixing 1.61 M PbI_2_, 0.048 M PbCl_2_, 0.08 M MAI, 0.2 M MACl, 1.43 M FAI and 0.08 M CsI in DMF:DMSO 4:1 (volume ratio). The solution was shaken for at least 1 h to make sure the precursors were dissolved and then filtered using 0.22-µm PTFE filters. The absorber was fabricated with the one-step antisolvent method. The precursor solution (100 µl) was spread onto the substrate (2.5 × 2.5 cm^2^) and then spun at 3,000 rpm at a rate of 300 rpm s^−1^ for 40 s. When 10 s remained during the spin programme, 250 µl of anisole was dropped onto the substrate. Then the samples were annealed at 100 °C for 15–20 min inside the glovebox.

#### Three-step absorber fabrication

On fabrication of the one-step perovskite absorber with the antisolvent method, the samples were loaded into a Lesker evaporation chamber. Inorganic halide template consisting of 180 nm PbI_2_ and 3 nm CsI was co-evaporated onto the one-step absorber at rates of 1 A s^−1^ and 0.015 A s^−1^, respectively. Then, organohalides (0.3 M FAI:MAI at a ratio of 95:05 in IPA and 2.5 mg ml^−1^ as additive) were spin-coated dynamically at 4,000 rpm for 30 s. To form the three-step absorbers, samples were annealed at 100 °C for 10 min.

### Solar cell fabrication

#### Fabrication of single-junction perovskite solar cells

0.7 mm ITO (15 ohm per square, TKing) coated glass substrates were cleaned with 3% Hellmanex and deionized water using ultrasonic baths for 15 min. Samples were exposed to ultraviolet ozone for 30 min just before the SAM deposition. SAM (for example, Me-4PACz) solution (1 mg ml^−1^) was spin-coated onto substrates for 100 µl with a programme of 10 s resting time, 3,000 rpm and 300 rpm s^−1^. Then the substrates were annealed for 10 min at 115 °C. To improve the wetting, 0.25% SiO_*x*_-np solution in ethanol (for WBG) and 0.1% SiO_*x*_-np for MBG in ethanol (20 nm diameter) was spin-coated for 100 µl with a programme of 2,000 rpm with 500 rpm s^−1^ for 30 s and annealed at 115 °C for 10 min (ref. ^16^).

##### Interface passivation

For the passivation on top of the MBG absorber, 0.5 mg ml^−1^ PDAI_2_ in IPA was dynamically spun at 6,000 rpm, followed by annealing at 100 °C for 5 min or if 1 nm EDAI_2_ had evaporated.

For the bimolecular passivation (BMP) on top of the WBG perovskite absorber, 0.4 mg ml^−1^ PCl was dynamically spun at 6,000 rpm and annealed at 100 °C for 5 min. After cooling down, 0.5 mg ml^−1^ 4F-PEACl was dynamically spun at 6,000 rpm and annealed at 100 °C for 5 min.

##### Top electrode stack

25 nm of C_60_ (CreaPhys or Nano-C, >99.9%, resublimed) was deposited through thermal evaporation at a rate of 0.2 A s^−1^ in an Angstrom thermal evaporator. Then ALD-SnO_*x*_ was deposited at 95–100 °C with pulse purge times of 0.3 s/6 s/0.1 s/6 s for 150–175 cycles with TDMASn and H_2_O sources. The thicknesses used for baseline devices are 20–22.5 nm measured with an ellipsometer on c-Si. For the metal contact, 150 nm of Ag was deposited through thermal evaporation at a rate of 1.5–2.0 A s^−1^. The active area of the device is 0.2 cm^2^.

### Fabrication of monolithic MBG–Si heterojunction tandem solar cells: 1 cm^2^

The bottom-cell fabrication process was performed on 200-µm-thick n-type, 2 ohm cm, FZ Si wafers. For the front-flat devices, a layer of SiN_*x*_ was applied to the front side using plasma-enhanced chemical vapour deposition (PECVD) and the rear side was textured using a KOH solution to create a random pyramid pattern. The SiN_*x*_ masking layer was then removed and samples underwent a cleaning sequence before the PECVD process. For the double-sided nanotextured devices, KOH solution with an additive was used to create submicron textured pyramids^51–53^.

Through PECVD, hydrogenated amorphous Si layers were deposited on both sides. On the front side, a-Si:H(i), a-Si:H(n) or nc-Si:H(n) were deposited at 200 °C. On the rear side, a-Si:H(i) and a-Si:H(p) or nc-Si-:H(p) were deposited at 200 °C (ref. ^45^). On the rear side, an 80-nm-thick ITO layer and a 500–600-nm-thick Ag layer were deposited by sputtering through a shadow mask. The front side was completed by sputtering a 20-nm-thick ITO layer for interconnection. As the final step before top-cell processing, 4-inch wafers were then laser-cut into 2.5 × 2.5-cm^2^ substrates, which were annealed at 210 °C for 15 min in ambient to recover the sputter-induced damage.

A 1:1 mixture of Me-4PACz (1 mg ml^−1^ in ethanol) and 4PADBC (1 mg ml^−1^ in ethanol) or 4PABCz (1 mg ml^−1^ in ethanol) was spin-coated statically at 3,000 rpm for 30 s with 10 s resting time, followed by an annealing at 125 °C for 10 min. To construct the middle reflector, 0.25–0.50 wt% SiO_*x*_-np (approximately 20 nm diameter) in ethanol was spin-coated statically at 2,000 rpm with 500 rpm s^−1^ acceleration, followed by an annealing step of 10 min at 125 °C. Either one-step or three-step absorbers were used for MBG–Si tandems with the recipes given above. For MBG solar cells, PDAI_2_ or EDAI_2_ passivation was used with the recipes given above. The thickness of C_60_ was reduced to 15–20 nm for the tandem devices and 150–175 cycles of ALD-SnO_*x*_ was used in tandems. After the ALD-SnO_*x*_, 30–35 nm of IZrO with sheet resistance around 250 ohm per square was deposited from 4-inch 98% I_2_O_3_ + 2% ZrO target with a deposition power of 70 W and working pressure of 2.7 µbar with about 0.14% of O_2_ to Ar ratio. After the TCO, about 500 nm of Ag grid was evaporated at a rate of 2 Å s^−1^, which was followed by 100 nm of NaF at a rate of 1 Å s^−1^.

We note that NaF, although offering slightly better anti-reflective properties (owing to slightly lower refractive index compared with MgF_*x*_) and improved evaporation repeatability (with evaporation temperatures lower than MgF_*x*_), is highly hygroscopic. Under high humidity conditions (>60% relative humidity), NaF can form a ‘skyscraper-like’ microstructure (Supplementary Fig. 80) and therefore its use is not recommended in humid environments.

### Fabrication of triple-junction solar cells: 1 cm^2^

Fabrication of the bottom cell is similar to the MBG–Si tandems. For the middle cell, the C_60_ thickness was increased to 30 nm and the number of cycles of SnO_*x*_ increased to 300. The goal of using thicker interconnection films (C_60_, SnO_*x*_) is to improve the barrier properties. For the interconnection layer, 15 nm of IZO was deposited with a mask of 1.3 × 1.3 cm. For the top cell, a 1:1 mixture of Me-4PACz:2PACz or Me-4PACz:4PABCz (1 mg ml^−1^) in ethanol was used. 100 µl of mixed SAM solution was spun statically with 5 s resting time at 3,000 rpm for 30 s, followed by an annealing at 95 °C for 5 min. To improve the wetting, 0.25% SiO_*x*_-np was spun statically at 2,000 rpm for 30 s, followed by an annealing at 95 °C for 5 min. 100 µl of WBG perovskite solution was spread and spun at 5,000 rpm with an acceleration of 1,000 rpm s^−1^ for 35 s. In the final 10 s, 200 µl of anisole was dropped on the substrate. Samples were annealed at 100 °C for 10 min. For the interface passivation, the exact procedure mentioned above was applied. 15 nm of C_60_, 20 nm of SnO_*x*_ and 25–30 nm of IZrO with sheet resistance around 350 ohm per square and 500 nm of Ag were deposited as the negative electrode stack. 110 nm of NaF or MgF_*x*_ was deposited as an anti-reflective coating.

### Fabrication of large-area triple-junction solar cells on 4-inch wafers

Fabrication of the bottom cell is similar to that in MBG–Si tandems, except instead of doped nc-Si-based layers, a-Si-based n-doped and p-doped layers were used for simplicity. For the middle cell, the C_60_ thickness was increased to 35 nm and the number of cycles of SnO_*x*_ was increased to 350. The goal of using thicker interconnection films (C_60_, SnO_*x*_) is to improve the barrier properties. For the perovskite–perovskite interconnection layer, 20 nm of ITO was deposited through a mask. For the top cell, 4PABCz (1 mg ml^−1^) in ethanol was used. 100 µl of SAM solution was spun statically with 5 s resting time at 3,000 rpm for 30 s, followed by an annealing at 95 °C for 5 min. To improve the wetting, 0.25% SiO_*x*_-np was spun statically at 2,000 rpm for 30 s, followed by an annealing at 95 °C for 5 min. WBG perovskite solution was spread and spun at 5,000 rpm with an acceleration of 1,000 rpm s^−1^ for 35 s. In the final 10 s, anisole was dropped on the substrate. Samples were annealed at 95 °C for 10 min. For the interface passivation of both sub-cells, about 1.5 nm EDAI_2_ was evaporated^54^. 20 nm of C_60_, 20 nm of SnO_*x*_ and 40 nm of ITO with a sheet resistance of around 150 ohm per square were deposited as the negative electrode stack. Instead of the evaporated metal contacts, screen-printed Ag (about 10 µm) cured at 130 °C was used^55^. 110 nm of MgF_*x*_ was deposited as an anti-reflective coating.

### Encapsulation

The devices were encapsulated using a thermoplastic polyolefin encapsulant with temperatures lower than 140 °C. A polyisobutylene edge sealant was applied to ensure effective moisture and oxygen barrier protection. Electrical interconnection was achieved using adhesive conductive ribbons.

### Characterization

#### DFT

DFT calculations were performed with the electronic structure code VASP^56,57^. We use the C_GW_new, N_GW_new, O_GW_new, H_GW_new, Pb_d_GW, Cs_sv_GW and I_GW PAW pseudo-potentials^55^, as distributed with vasp.6.4.2. Furthermore, the calculations used a plane-wave cut-off of 750 eV (ENCUT in VASP) and simulation boxes of dimensions 20 × 20 × 20 Å. Geometries were fully relaxed using the r^2^SCAN + rVV10 density functional^58^, which combines the regularized-restored version^59,60^ of the SCAN meta-GGA functional^61,62^ with the rVV10 van der Waals correction^63,64^. We used *b* = 11.95 for the rVV10 parameter as suggested by Ning et al.^58^. Calculations were performed in vacuum and we neglected spin–orbit coupling corrections and vibrational contributions to the binding energies.

#### *J*–*V*

The 4PP measurement method was used. *J*–*V* measurements were obtained using a two-lamp (halogen and xenon) class AAA WACOM sun simulator with an AM1.5G irradiance spectrum at 1,000 W m^−^^2^. 0.1 cm^2^ shadow masks were used to measure cells with an area of 0.2 cm^2^ unless otherwise stated. Opaque devices were illuminated from the glass side. The single-junction cells were measured with a scan rate of roughly 0.50 V s^−1^, first reverse scan and then forward scan. Both reverse and forward scans are included in the *J*–*V* statistic plots. Three-point weight MPP measurements were performed using an in-house written LabVIEW code. For the measurement of tandem and triple-junction devices, before each measurement, the calibration of the AAA WACOM system was adjusted with three different certified cells with different spectral responses to minimize the spectral mismatch of the sources. Initially, to adjust the intensity of the Xe lamp, a calibrated encapsulated cell (WPVS, blue cell) containing a monocrystalline silicon solar cell, which is covered with a KG5-filter, was used. Then to adjust the intensity of Ha lamp, a calibrated encapsulated cell (WPVS, red cell) containing a monocrystalline silicon solar cell, which is covered with a RG780 filter, was used. Then the overall spectrum was checked with a calibrated encapsulated cell (WPVS without filter broadband, black cell). All calibrated Si cells were provided by Fraunhofer Institute for Solar Energy Systems. Then the cells of about 1 cm^2^ were measured with a scan rate of roughly 0.1 V s^−1^ and a similar MPP tracking algorithm was used as the single-junction perovskite solar cells. A temperature-controlled (25 °C) brass chuck was used. 4-cm^2^ cells on 4-inch wafer were contacted using two Kelvin probes on either side of the metal grid. Large-area cells (53.9 cm^2^) were contacted using Kelvin probe bars by contacting each bus bar. Shading as a result of probe bars was corrected after the measurements by application of the correction factor (×1.016) to *J*_SC_, following our previous work^65^.

#### EQE

EQE spectra were measured with a custom-made spectral response set-up in which the samples were irradiated with chopped light at a frequency of about 200 Hz and the response was measured with a lock-in amplifier. For tandem cells, visible and infrared light biases were used to saturate complementary sub-cells and to measure each sub-cell near short-circuit conditions, 0.6 V and 1.1 V bias voltages were applied to the cell when measuring the top and bottom cells, respectively.

EQEs of the triple junctions were measured over the full area, including shadowing from the fingers, using the pv-tools LOANA system. For the measurement of triple-junction devices, the signal in the junction that is measured was maximized and other junctions were minimized by adjusting the light and voltage bias. For example, while measuring the top cell, the signal in the top cell (for example, at 550 nm) was maximized by adjusting the voltage bias (for example, 0.5 to 2 V) and light bias (for example, infrared LED) and the middle cell and bottom cell were minimized (for example, 1,000 nm). For the middle cell, a voltage range of 0.5–2 V was used with blue LEDs. For the bottom cell, a voltage range of 0.5–2 V was used with blue LEDs with white light.

#### *J*–*V* and EQE at Fraunhofer Institute for Solar Energy Systems

EQEs were measured with an in-house measurement set-up containing a xenon lamp as a light source. The light was chopped at about 133 Hz and directed through a double-grating monochromator to produce monochromatic light. A transimpedance amplifier provided a bias voltage during the measurements and strengthened the signal, which was then detected by a lock-in amplifier. Selective infrared and red LEDs (940 nm and 740 nm, respectively) were used for the measurement of the perovskite top cell. Blue (460 nm) and infrared LEDs were used for the measurement of the perovskite middle cell. The silicon bottom cell was measured under selective blue and red LEDs. A bias voltage was applied to the device for each sub-cell measurement, following the international standard procedure^66^. The EQE was recorded in 10-nm increments and the temperature was maintained at 25 °C.

The *J*–*V* measurements of triple-junction solar cells were conducted using a Wavelabs SINUS 220 light-emitting diode (LED)-based solar simulator containing 20 different LED channels. The spectral responses were measured before the *J*–*V* measurement. The spectrum was then calculated using the relative spectral responses according to the procedure described in ref. ^67^. This ensured that the spectrum was correctly adjusted such that the photocurrent generated by each sub-cell under the simulator spectrum matched the photocurrent generated under the AM1.5G spectrum^68,69^. The *J*–*V* curves were then measured from 3,200 mV up to 0 mV with a voltage step of 10 mV and a scan rate of about 0.1 V s^−1^ first in the reverse scan direction and then in the forward scan direction. The temperature of the measurement chuck was maintained at 25 °C.

#### Long-term stability

The Cicci Research stability measurement set-up was used, in which the samples were kept in N_2_ atmosphere, under 1-sun illumination by tracking the MPP with the perturb and observe algorithm. The N_2_ chambers are temperature-controlled and cells saturate about 10–15 °C higher than the chamber temperature.

For stability testing for WBG devices in MPP, 2PACz hole transport layer and no perovskite–C_60_ interface passivation was used, for simplicity.

#### Outdoor testing

To evaluate the field performance of the encapsulated triple-junction cells, time series data were analysed from two devices tested outdoors in Neuchâtel, Switzerland, between 6 February and 3 June 2025. Global horizontal irradiance was measured using a pyranometer co-located with the test set-up.

The performance ratio was computed and analysed over time to extract long-term trends and identify potential anomalies. The performance ratio is a dimensionless metric that compares the actual output to its expected output under standard test conditions (STC). It normalizes the power output of the device by both its nominal rating and the incident irradiance and is defined as:

$${\rm{PR}}(t)=\frac{{P}_{{\rm{MPP}}}(t)/{P}_{{\rm{nom}}}}{G(t)/{G}_{{\rm{STC}}}}$$

in which *P*_MPP_(*t*) is the MPP measured at time *t* (in mW), *P*_nom_ is the nominal power rating of the device under STC, *G*(*t*) is the measured irradiance at time *t* (in W m^−^^2^) and *G*_STC_ is the reference irradiance under standard conditions (1,000 W m^−^^2^).

Performance ratio values outside the plausible range (that is, below 0.3 or above 1.3) were excluded to filter out erroneous data and outliers. To study temporal evolution, the 10-min performance ratio values were aggregated into daily statistics. For each day, the average performance ratio was calculated along with its day-to-day variability, using standard deviation as a measure of spread. To reduce noise in the daily data and emphasize long-term behaviour, a Gaussian smoothing filter was applied to the daily performance ratio averages (*σ* = 8) to effectively smooth out short-term fluctuations while preserving broader performance patterns over time. Furthermore, confidence intervals were added above and below the smoothed trend line using the daily standard deviation.

#### High-dynamic-range EQE

High-dynamic-range EQE measurements were conducted using a home-built set-up. Here the output of a tungsten–halogen light source (Thorlabs, SLS201L) was fibre-coupled into a Monochromator-F system (Technologie Manufaktur). The monochromator fibre output light was collimated and physically chopped at 273 Hz (Thorlabs, MC2000B) and different optical components were used to guide the light onto the device under test. Various long-pass and short-pass filters from Thorlabs and Edmund Optics were used to suppress optical noise and parasitic stray light. The device under test photocurrent signal was first amplified by a current amplifier with variable gain up to 10^9 ^V A^−1^ (Femto, DLPCA-200) before being analysed with a lock-in amplifier (Stanford Research, SR865A). During the measurement, the devices under test were under constant dry nitrogen flow. Before EQE measurements, the system was calibrated using a NIST-calibrated silicon (Newport, 818-UV) and germanium (Newport, 818-IR) reference photodiode. Electrical bandwidths of up to 1 MHz were used for sub-bandgap EQE measurements.

#### Transient absorption measurements

Femtosecond transient absorption measurements were performed in a HARPIA commercial instrument (Light Conversion) equipped with a Kymera 193i spectrometer (Oxford Instruments) and a broadband Si NMOS detector (Hamamatsu S3901 + C7884). An amplified 260-fs pulse train, centred at 1,026 nm, with an average power of 20 W and a repetition rate of 50 kHz, was generated from a Pharos Yb:KGW laser (Light Conversion). The pulse train was divided into three parts. An optical parametric amplifier (Orpheus, Light Conversion) was pumped with 17.5 W and generated a pulse train centred at 640 nm. This train served as the pump pulse and was further modulated at 40 Hz. The pump beam profile was measured with the knife-edge method yielding a full width at half maximum of 2.68 mm from an error function fit. The pump intensity was adjusted to yield an irradiance per pulse of 50 nJ cm^−2^. For the probe pulse, 0.5 W of the initial 1,026-nm pulse train was routed to a motorized delay stage, providing 7 ns of delay. A white-light supercontinuum was generated by attenuating it with a variable neutral density filter and focusing it into a YAG crystal. The remaining 2 W were not used in the experiment. The probe beam was focused onto the sample with a pair of parabolic mirrors and overlapped spatially and temporally with the pump pulse. All experiments were performed at room temperature.

#### SEM

SEM images were acquired with acceleration voltages ranging from 1 to 5 kV using either an Everhart–Thornley or an in-lens detector (JEOL JSM-7500TFE or Zeiss NVision 40 microscopes).

#### Steady-state photoluminescence

A custom-built steady-state photoluminescence set-up was used to measure in situ photoluminescence and PLQY. The light from the laser diode was coupled into a fibre directed into the integrating sphere and sample was illuminated. Then the emission from the sample was homogenized by several reflections within the integrating sphere and coupled out into another fibre that is connected to a spectrometer. As long as the emission (600–825 nm) and the excitation (532 nm) are well separated, a careful comparison of the peaks enables us to measure the PLQY (down to 10^−6^). An OD filter wheel helps to change the light intensity between 0.01 sun and 3 sun. As a calibration check, three fluorescent test samples with high specified PLQY (about 70%) supplied from Hamamatsu Photonics were measured, for which the specified value could be accurately reproduced to within a small relative error of less than 5%. For quasi-Fermi level splitting (QFLS) measurements, the light intensity is tuned to 1 sun depending on the bandgap of the absorber using an external c-Si photodetector.

Non-radiative losses are extracted from the PLQY using the following formula

$${{\rm{NR}}}_{{\rm{loss}}}=-\left(\frac{{k}_{{\rm{B}}}T}{q}\right)\mathrm{ln}({\rm{PLQY}})$$

#### i*V*_OC_ imaging

A commercial measurement system developed by Fraunhofer Institute for Solar Energy Systems and manufactured by Intego GmbH was used to measure the photoluminescence images. A laser with a peak wavelength of 450 nm, an LED with a peak wavelength of 700 nm and a laser with a peak wavelength of 808 nm were used to selectively excite the sub-cells. Optical filters (transmissive range 600–650 nm for the top solar cell, 788–812 nm for the middle solar cell and 950–1,000 nm for the bottom solar cell) were used to separate the luminescence signal from the sub-cells, before it was captured in a silicon charge-coupled device camera. The relative EQEs were used to calculate the 1-sun-equivalent illumination intensities of the two lasers following the approach described in ref. ^69^. The intensity of the 700-nm LED was roughly adjusted (here we note a possibility of +35 mV overestimation in the middle-cell i*V*_OC_) using a silicon reference solar cell. The photoluminescence images were calibrated to obtain i*V*_OC_ images following the approach described in ref. ^46^, adapted to triple-junction solar cells.

#### XPS/UPS

XPS measurements were performed using an Axis Supra (Kratos Analytical) with a monochromatic Al Kα X-ray line. The pass energy was set to 20 eV with a step size of 0.1 eV for XPS and to 40 eV with a step size of 0.15 eV for angle-resolved XPS. The emission current was set to 15 mA. The samples were electrically grounded to limit charging effects. UPS was measured using a He I ultraviolet source, with the pass energy set at 10 eV and a step size of 0.025 eV. The intensity of the ultraviolet source together with the measurement area was selected to prevent the energy analyser from saturating. Secondary electron cut-off was used to assign the work function of the material, whereas the valence band onset was used to estimate the valence band maximum with respect to the Fermi level.

#### GIWAXS

GIWAXS experiments were performed at the NCD-SWEET beamline at the ALBA Synchrotron Light Source, Spain. An in vacuo undulator (IVU21) generates a low-divergence X-ray beam that was monochromatized at 12.95 keV with a Si (111) channel cut monochromator. After the beam-defining slits, the X-ray beam was collimated using an array of beryllium compound refractive lenses, obtaining a beam size at the sample position of 20 × 80 µm^2^ (full width at half maximum: H × V). The sample was positioned on the rotation point of a Huber double semicircle located on a complete translation stages tower, which allowed precise tilts and movements of the sample. A Rayonix LX255-HS area detector (88.54 × 88.54 µm^2^ pixel size for the binning mode used of 2 × 2) was used to record the scattered radiation. The reciprocal space was calibrated using Cr_2_O_3_ from NIST, obtaining a sample to detector distance of 230 mm. Data were reduced using a home-built programmed routine based on PyFAI^70^. In situ spin coating during GIWAXS data collection was performed using a brushless motor (Mclennan BLDC 48) with a 0–5 V speed control, calibrating the rotation speed with the integrated frequency generator. The spinner was covered with a 3D-printed cover to ensure an inert N_2_ atmosphere with Kapton windows. The solution injection was remotely controlled using a Spetec Symax syringe pump, injecting 150 µl and setting a rotation speed of 2,000 rpm. For the in situ annealing experiments, the sample temperature was controlled using a Linkam stage and the sample was under continuous N_2_ gas flow during the in situ measurements.

#### NMR

1H NMR spectra were recorded on a Bruker AV4 spectrometer (1H: 400.1 MHz) equipped with a 5-mm BBO probe at 298 K. Spectra were acquired with a 30° pulse of 3.3 μs, a spectral width of 7.8 kHz (20 ppm), an acquisition time of 4 s, a preparation delay of 1 s and between 16 and 128 accumulations. The free induction decays were zero-filled to 65 K points and multiplied by a 0.3-Hz exponential line-broadening function before Fourier transformation into the frequency domain. 1H chemical shifts were referenced to tetramethylsilane using the signals of the residual protons of the deuterated solvent (δ1H = 2.50 ppm (DMSO-d6)) as the secondary reference.

#### Finite element simulations

Finite element simulations were performed using the commercially available software COMSOL. The simulated tandem stack from top to bottom is NaF/IZrO/SnO_*x*_/C_60_/perovskite/SiO_*x*_-np/ITO/Si, with the respective layer thicknesses being 110 nm/30 nm/20 nm/15 nm/500 nm/0–150 nm/20 nm/200 µm. This stack was implemented in a 2D geometry, in which the Si top structure was approximated as periodic triangles with a height of 260 nm and a base angle of 54.7°, whose valleys are filled by circular nanoparticles. The perovskite height is adjusted according to the nanoparticle filling height to maintain the volume. Using the electromagnetic waves module in COMSOL, which solves the wave equation numerically for the given stack with supplied boundary conditions, the wavelength of the incoming light is varied. For each wavelength, the EQE of a layer is calculated by integrating over the absorption of each of its mesh points and dividing by the input power.

#### ToF-SIMS

ToF-SIMS measurements were performed with a time-of-flight secondary ion mass spectrometer (ToF-SIMS V system, ION-TOF). The primary beam was 25 keV Bi3+ with a total current of 0.46 pA and a raster size of 50 × 50 μm^2^. Cs^+^ ions were used with 1,000 eV ion energy and 39 nA pulse current or O_2_^+^ ions were used with 1,000 eV ion energy, 19 nA pulse current on a 150 × 150 μm^2^ raster size to bombard and etch the film. The collected secondary ion spectra were normalized to the total counts.

#### X-ray diffraction

X-ray diffraction was performed in Bragg–Brentano geometry with a copper Kα source with a Kβ filter using a PANalytical X’pert Pro MPD.

#### Transient surface photovoltage

Surface photovoltage measurements at 1 Hz were performed on encapsulated samples using 688-nm excitation laser light from a diode-pumped tunable pulse laser (Nd:YAG, EKSPLA, NT230-50, duration time of laser pulses 3–5 ns) with a spectral cleaning unit. The measurement frequency was 1 Hz, 30 averages were taken per transient and the laser intensity (fluence of 0.010 μJ cm^−2^ equivalent to 1 sun) was controlled using neutral density filters. The surface photovoltage transients were measured with an oscilloscope card (GaGe, CSE 1622-4GS, 200 MS s^−1^) using in-house-developed software for logarithmic readout^33–35,71^. Excitation was performed from the perovskite side.

#### Bias-assisted charge extraction

The device was initially biased at a voltage equivalent to the *V*_OC_. After a delay time of 30 s, a bias of 0 V was applied to extract the injected and capacitive charge within the device. The transient current was recorded using a Keithley 2400 instrument controlled by a custom LabView program. The extracted charge was calculated by integrating the transient current, whereas the charge carrier density was determined by dividing the total charge by the elementary charge and the cell volume^72,73^.

#### Mechanical characterization

Double cantilever beam (DCB) samples used a structure of glass/super glue/Si wafer/SAM/one-step or three-step perovskite/PMMA/Ag/epoxy/glass. The PMMA layer (about 800 nm) was spin-coated onto the perovskite for protection against the Ag, using a 0.1 g ml^−1^ PMMA solution in chlorobenzene at 3,000 rpm for 30 s. The epoxy used was Loctite Epoxy Instant Mix 5min, which was applied to a bare glass superstrate and bonded to the glass substrate deposited with perovskite stacks to form DCB samples. Specific details of the testing method can be found elsewhere^74^. Briefly, a pre-crack was introduced into the DCB sample along the orientation of the crack propagation by inserting the tip of a razor blade into the DCB sample. The resulting pre-crack can protect the DCB sample from excessive tensile loads and initiate crack propagation. By using the DTS Delaminator system, the pre-cracked DCB sample was mounted and loaded in tension at a displacement rate of 1 μm s^−1^. When a clear drop occurred in the load–displacement curve, the DCB sample was partially unloaded to calculate the compliance and then loaded again to gradually propagate the crack until a complete separation was achieved for the two bonded glass substrates, suggesting the completion of the measurement. In the measurement, the load (*P*)–displacement (*Δ*) curves were continuously recorded and used to extract the fracture energy (*G*_c_), which can be calculated from equation (1) (ref. ^75^)

1 $${G}_{{\rm{C}}}=\frac{12{P}_{{\rm{c}}}^{2}{a}^{2}}{{B}^{2}E{\prime} {h}^{3}}{\left(1+0.64\frac{h}{a}\right)}^{2}$$

in which *P*_c_ is the critical load that deviates from the linearity in the *P*–*Δ* curve during the loading cycle; *a* is the crack length; *B* and *h* are the width and half-height of the sample, respectively; and *E*′ is the plane-strain elastic modulus of the substrate. The crack length was estimated using the compliance method in equation (2)

2 $$a={\left(\frac{{\rm{d}}\varDelta }{{\rm{d}}P}\times \frac{BE{\prime} {h}^{3}}{8}\right)}^{\frac{1}{3}}-0.64\times h$$

All *G*_c_ testing was carried out in laboratory air environment at about 20 °C and about 20% relative humidity.

## Online content

Any methods, additional references, Nature Portfolio reporting summaries, source data, extended data, supplementary information, acknowledgements, peer review information; details of author contributions and competing interests; and statements of data and code availability are available at https://doi.org/10.1038/s41586-026-10385-y.

## Supplementary information

Supplementary Information Supplementary Notes 1–14, Supplementary Tables 1–3, Supplementary Figs. 1–82 and Supplementary References.

Peer Review File

## Source data

Source Data Fig. 3

## Data Availability

Source data are provided with this paper. All other data of this work are available from the corresponding authors on request.

## Code availability

The custom LabVIEW code used for data acquisition and three-point MPP tracking, as well as the MATLAB code used for numerical calculations of the equivalent circuit model, are available from the corresponding authors on request.
