# Supplementary material for: Triple-junction solar cells with improved carrier and photon management
Source: Nature. 2026 Mar 17;653(8113):90–7. doi: 10.1038/s41586-026-10385-y (PMC13658016; doi:10.1038/s41586-026-10385-y)
Supplement: Supplementary file 1 [file 41586_2026_10385_MOESM1_ESM.pdf]

nature portfolio

https://doi.org/10.1038/s41586-026-10385-y

Supplementary information

Triple-junction solar cells with improved carrier and photon management

In the format provided by the authors and unedited

Supplementary Information for

Triple-junction solar cells with improved carrier and photon management

Artuk *et al.*

Corresponding authors: Kerem Artuk, kerem.artuk@epfl.ch; Christian M. Wolff, christian.wolff@epfl.ch

The PDF file includes:

Supplementary Notes 1 to 14
Tables S1 to S3
Figures S1 to S82
References (*76–106*)

Supplementary Note 1: NMR analysis of HBA additive stability in perovskite precursor solutions

To evaluate the chemical stability of the hydroxybenzylamine (HBA) additives in the perovskite precursor solution, we performed proton nuclear magnetic resonance (^1H NMR) spectroscopy on simplified inks comprising FAI and PbI_2, both with and without 2-, 3-, and 4-HBA. Spectra of the isolated HBA molecules were also recorded for reference (Fig. S3).

For freshly prepared inks, no detectable shifts in the characteristic HBA resonances were observed relative to the isolated molecules. In addition, no new peaks emerged in the additive-containing solutions, indicating that the HBAs remain chemically intact and do not form detectable byproducts upon mixing with the perovskite precursor components.

To assess stability during storage, NMR spectra were acquired after one week. Minor changes were observed in the $-NH_2$ resonance of FAI (Figs. S4–S5). However, similar changes were detected in the reference ink without HBA additives, indicating that this evolution is not induced by HBA incorporation. Importantly, the non-exchangeable aromatic resonances of the HBA derivatives remained unchanged upon aging (Fig. S4b), and no additional resonances corresponding to decomposition products were detected.

These results demonstrate that the HBA additives remain chemically stable in the perovskite precursor solution, both immediately after preparation and after extended storage.

Supplementary Note 2: Crystallization dynamics and texture evolution with HBA additives

The identified interactions between the HBA and perovskite precursors are thus suspected to not only facilitate passivation but also influence the film formation kinetics. To test this hypothesis, we tracked the film evolution during crystallization using in-situ GIWAXS measurements performed during spin-coating and annealing. During spin-coating (**Fig. 1(c)**), we monitored the evolution of the structural building blocks of the perovskite film. Initially, a low-angle peak emerges at ~2 nm^{-1} at 170 s and disappears by 190 s, coinciding with the onset of the 6H perovskite forming (~10.2 nm^{-1}) for both the control and the 2-HBA samples. In contrast, for samples containing 3- and 4-HBA, the low-angle perovskite peaks are present from the beginning of the spinning. Furthermore, we observe additional intermediate building blocks (e.g., additional 4H phase duplets at ~8.5 & 9.5 nm^{-1}, **Fig. 1(c)** and **Fig. S7-S8**) for the 2-, 3-, and 4-HBA cases, while being absent in the control (*76*, *77*).

After the in-situ spin-coating process, the control perovskite exhibits a broad 6H peak distribution with two predominant phases (one richer in Br, one richer in I, based on the nature of peak splitting), indicative of heterogeneous perovskite phases with varying halide content. The disordered 6H signal is likewise present in the HBA modified samples, albeit to a much lesser extent. Subsequently, during annealing (see **Fig. S9**), the disordered perovskite phases merge into a singular, more ordered phase for all samples. However, for samples containing 2-, 3-, and 4-HBA, this process is slower, allowing more time for halide homogenization, which is also reflected in the overall slower 6H formation kinetics when employing the HBA additives (example shown in **Fig. S10**, for convenience plotted in the Avrami representation). 4-HBA results in the slowest process among the three positional isomers, which is then inversely correlated with the adsorption energy calculated from DFT (see **Table S1**) to the perovskite building block, $CsPbI_3$. In fact, the slowest isomer also achieves the lowest non-radiative losses, pointing out the importance of crystallization dynamics to the resultant film quality. Furthermore, we deem it likely that the excess solvent evaporation is mostly completed before we started the in-situ annealing experiment. Hence, during this period, the only remaining interactions are between the perovskite (precursor, including Pb-solvent complexes) phases themselves and the additive, without the impact of excess solvent.

After the perovskite formation, ex-situ GIWAXS measurements were performed on cooled samples to compare film textures, **Fig. S11**. The out-of-plane (OOP) peak weighting versus misorientation, indicating film texture, progressively increases from the control to 4-HBA samples (**Fig. 1(d)**). Additionally, another notable difference is a reduction in the unit cell size with the incorporation of HBA additives (see **Fig. S12** for peak shift to higher degrees). Particularly for 4-HBA, the slowed crystallization is evident, and it is plausible that it is caused by the variations in the evolution of the building blocks, influenced by interactions with HBA molecules. Such slower kinetics allow the domains additional time and flexibility to align during growth, all the while providing sufficient time for halide homogenization. A consequence of this deceleration is the enlargement of domain sizes, as demonstrated by the increase in average top-view domain size from 250 nm to 325 nm for 4-HBA. Notably, the 4-HBA absorber also features fewer horizontal domain boundaries, as observed by cross-sectional electron microscopy images (see **Fig. S13**). Ultimately, these combined effects facilitate the formation of higher-quality, preferentially out-of-plane-oriented crystalline domains with fewer recombination-active defects and domain boundaries in and out of plane.

Supplementary Note 3. High-dynamic-range EQE and transient absorption analysis of 4-HBA additive

High-dynamic-range external quantum efficiency (EQE) measurements were performed on both control and target (i.e., with 4-HBA additive) wide-bandgap perovskite single-junction solar cells. **Fig. 1(g)** presents the normalized EQE spectra as a function of photon energy. The control device exhibited a pronounced absorption feature in the band tail region near 1.6 eV, less prominent in the 4-HBA device. This observation suggests a suppression of phase segregation in the freshly fabricated, additive-treated device. Both devices furthermore displayed sub-bandgap absorption features at photon energies well below the bandgap. Although low-finesse optical cavity effects can influence the spectral line shape in this regime, the active layer thicknesses, approximately 350 nm for the control and 325 nm for the target device, differ only marginally (*78*). Consequently, the observed variation in sub-gap EQE intensity can be attributed to differences in sub-gap (trap) state density (*79*). Integration of the sub-gap EQE (highlighted in grey in **Fig. 1(g)**) indicates a ~25% reduction in the optically active trap density in the target device relative to the control. Using low-fluence transient absorption measurements, we confirm these observations from a dynamical perspective. The passivation with 4-HBA reduces the non-radiative losses, evident from a longer ground-state bleach recovery time constant (**Fig. S19**). The higher thin-film quality also results in a less-pronounced spectral shift of the ground-state bleach after excitation, a fingerprint of a reduction of phase-segregation **(Fig. S20)**, in line with the above-shown results.

Supplementary Note 4. Optimized contacts in WBG devices

After reducing bulk non-radiative losses by 100 mV using 4-HBA, we introduced a partial contacting strategy to address interfacial losses at the perovskite/hole contact. Insulating SiO_x-np were inserted between the perovskite and Me-4PACz to reduce the contact area (*80*). The low current in triple-junction cells allows higher SiO_x-np coverage than in two-junction tandems. Significant non-radiative losses (~240 meV) were also observed at the perovskite/C_{60} contact **(Fig. S21)**. Using bimolecular passivation with piperazinium chloride and 4-fluorophenethylammonium chloride (4F-PEACl), non-radiative losses decreased to ~ 130 meV (*81-83*).

The reduction of bulk non-radiative recombination by 4-HBA constitutes a general, material-level improvement that is expected to be broadly transferable across device architectures and absorbers (see Supplementary Note 5), as it originates from suppressed defect formation and the passivation of existing

within the perovskite absorber. However, in complete devices, bulk improvements can only be translated into voltage gains if the contact interfaces do not impose additional non-radiative recombination losses. Accordingly, to fully benefit from the bulk enhancement induced by 4-HBA and reach 1.405 V, both interfaces were passivated to minimize surface recombination velocities.

Importantly, once interfacial recombination channels are sufficiently suppressed, bulk losses become more clearly reflected in QFLS and device-level metrics. Conversely, recombination-active interfaces can mask bulk improvements; without optimized SAM and C_{60} passivation, the intrinsic bulk benefits introduced by 4-HBA would not fully translate into device-level voltage gains. Therefore, while 4-HBA addresses broadly applicable bulk limitations, optimized hole- and electron-contact interfaces remain essential to reveal and harness these improvements in high-performance WBG devices.

Supplementary Note 5. Universality of 4-HBA

To evaluate whether the effect of 4-HBA is limited to a specific absorber composition, we investigated perovskite films spanning bandgaps of 1.55 eV, 1.65 eV, 1.68 and 1.9 eV (Fig. S23). Across all compositions examined, incorporation of 4-HBA led to reduced non-radiative recombination losses, as evidenced by improvements in voltage-related metrics.

In single-junction solar cells based on $Cs_{0.10}FA_{0.90}PbI_3$ absorbers, the average open-circuit voltage increased by approximately 30 mV upon addition of 4-HBA to the precursor solution. This improvement corresponds to an absolute power conversion efficiency increase of 0.85% (Fig. S24). These results indicate that the influence of 4-HBA is not restricted to a single bandgap composition but extends across a range of iodide-rich perovskite systems.

Supplementary Note 6: Three-step hybrid deposition of the middle-bandgap absorber

To enable controlled bandgap reduction and absorber thickening, we implement a hybrid three-step deposition strategy that combines thermally evaporated lead halides with spin-coated organohalides in alcohol-based orthogonal solvents. This approach separates thickness and bandgap tuning from the constraints of single-step solution processing while preserving surface quality and transport characteristics.

Hybrid conversion approaches have been explored previously. Azmi et al. and Lin et al. demonstrated evaporation of inorganic lead-halide templates followed by solution-based conversion into organic–inorganic perovskites, typically forming thin conversion layers or 2D/3D capping structures (*84-85*). More recently, Ullah *et al.* reported layered hybrid deposition to increase absorber thickness and improve crystallinity (*86*). These studies established the feasibility of combining vapor-deposited inorganic precursors with solution processing to modify the absorber structure.

In contrast to prior implementations that primarily formed thin conversion layers or surface modifications, the present strategy converts an evaporated PbI_2 layer into a thick $FAPbI_3$-rich absorber integrated with a pre-formed one-step middle-bandgap layer. The objective is to tune the effective bandgap and increase absorber thickness across the full active region rather than introducing a surface-limited modification.

After deposition of the one-step middle-bandgap absorber, PbI_2 is thermally evaporated under high vacuum conditions (detailed deposition parameters are provided in the Methods). An FAI-rich organohalide solution in an alcohol-based orthogonal solvent is subsequently spin-coated onto the PbI_2 layer. The orthogonality of the solvent prevents dissolution of the underlying perovskite. Conversion is completed by annealing under controlled humidity. To reduce thermal load on the underlying layer, the annealing temperature is

lowered, enabled by the addition of urea to the organohalide solution to facilitate conversion at reduced temperature, following prior reports on low-temperature perovskite formation (*87*).

The resulting hybrid absorber is $FAPbI_3$-rich, yielding a reduced effective bandgap compared to the initial one-step middle-bandgap layer. Partial incorporation of Cs^+ and MA^+ supports stabilization of the perovskite phase during processing. This three-step architecture, therefore enables thickness and bandgap engineering while maintaining compatibility with the underlying device stack.

Supplementary Note 7: Why thick one-step absorbers fail: mechanical and ionic constraints

Perovskite solar cells employing ~1.54 eV Pb-based absorbers with the composition $Cs_{0.05}FA_{0.90}MA_{0.05}PbI_3$ (+5 % $MAPbCl_3$) have demonstrated exceptional performance, exceeding 26 % efficiency in several reports (*88-89*). Here, control devices with ~600 nm absorbers (**Fig. 2c**) exhibited a PCE of ~23 % (**Fig.2d**), with $V_{OC} \approx 1.18$ V and FF > 83 %. However, the photocurrent density remained limited to ~23 mA cm^{-2}. Initial attempts to increase the absorber thickness by raising the precursor molarity to 1.8 M or modifying the spin-coating parameters led to film delamination upon annealing (**Fig. S26**). This behavior is attributed to increased mechanical stress between the perovskite and the hydrophobic Me-4PACz hole transport layer (*90*). Cross-sectional and top-view SEM confirmed that thicker one-step films developed wrinkled and rough morphologies, features commonly associated with stress-relief mechanisms in thick perovskite layers (see **Fig. S27-S32**).

Double cantilever beam fracture measurements show an increased fracture energy (G_c) for thicker one-step films deposited on Me-4PACz (**Fig. S33**), as the film thickness increases from 600 nm to 1000 nm. Under otherwise identical deposition conditions, such a thickness increase is expected to raise the driving force for delamination (G) by a factor of (1000/600) or ~67% based on the fact that G scales linearly with film thickness. However, G_c increases by only ~50%, from 0.4 to 0.6 J.m^{-2}, which is insufficient to compensate for the increased mechanical driving force. This imbalance provides a plausible explanation for the delamination observed in the thicker films (**Fig. S26**) for fracture tests of samples containing Me-4PACz, where the perovskite absorber completely delaminated from the Si substrate, indicating that the Me-4PACz/perovskite interface was the weakest layer in the device stack.

Alternative SAMs, including 2PACz, 4PADCB, and 4PABCz, partially mitigated delamination but did not recover device performance. For devices based on 4PADCB or 4PABCz, absorber thickening beyond 600 nm to ~1000 nm led to pronounced hysteresis and degraded performance without any net gain in J_{SC} (**Fig. S34**). SEM analysis confirmed that these thick one-step films still developed wrinkled and rough morphologies despite retaining large, columnar grains (Fig. S29-31). Such morphological changes could exacerbate ionic transport and recombination losses (*91-93*), offsetting the potential photocurrent gains from increased optical absorption. Similar observations have been reported for thick anti-solvent–processed middle-cells in perovskite triple-junction devices (*93)*.

To overcome these mechanical and morphological limitations, we developed a three-step deposition process (**Fig. S35**) that enables the fabrication of thick, low-bandgap perovskite absorbers without inducing wrinkling or delamination. Three-step-processed films preserve the smooth, mirror-like surface characteristic of thin one-step absorbers (**Fig. S27** and **S30**) while achieving substantially higher fracture energies on both Me-4PACz and 4PABCz. For three-step absorbers, perovskite delamination from the Si substrate is strongly suppressed relative to one-step films, indicating more robust interfacial bonding at the 4PABCz/perovskite interface (**Fig. S34**). Furthermore, only a portion of the perovskite remained attached to the Ag side after fracture, indicating that the 4PABCz/perovskite interface was no longer the weakest layer in the measurement stack. This observation suggests stronger bonding at the SAM/perovskite interface for 4PABCz, and the measured G_c of 1.04 ± 0.17 J·m^{-2} represents a lower bound of the 4PABCz/perovskite

interface adhesion. Moreover, three-step absorbers exhibit consistently higher adhesion on 4PABCz than on Me-4PACz, underscoring the coupled role of SAM chemistry and perovskite processing in determining interfacial mechanical stability. Optical microscopy and SEM confirm the formation of smooth, compact films with preserved interfacial integrity.

Despite these improvements to the adhesion of the bottom interface, the perovskite/C_{60} interface, along with the adjacent C_{60}/ALD-SnO_x interface, remains among the mechanically weakest regions of the device stack due to limited interfacial adhesion, warranting further interfacial engineering to enhance mechanical robustness and long-term stability (*94–96*). Recent studies by Poma *et al.* and Schutt *et al.* have demonstrated that incorporating alternative interlayers or modified electron-transport materials at this interface can substantially increase fracture energy, thereby improving mechanical durability (*95-96*).

Importantly, in contrast to thick one-step films, three-step absorbers maintain efficient charge extraction and do not exhibit elevated mobile ionic density (see **Fig. S36** for BACE measurements) (*91*, *92*). This conclusion is supported by transient surface photovoltage and bias-assisted charge-extraction measurements, which collectively reveal maintained mobile ionic density and efficient charge carrier dynamics characteristics in the three-step films (**Fig. S37**). Together, these results demonstrate that the three-step deposition strategy not only alleviates the mechanical stress inherent to conventional one-step antisolvent processing but also preserves the optoelectronic quality required for efficient middle-cell operation in perovskite-based triple-junction architectures.

Supplementary Note 8: Repeatability, process window, and trade-offs of the three-step absorbers

Compared to purely hybrid-processed absorbers, the three-step approach exhibits a broader and more forgiving process window. In our implementation, full or near-complete conversion of the evaporated PbI_2 layer is achieved over an organohalide concentration range of approximately **0.25–0.35 M**, within which device performance remains stable, while 0.25M results in minor PbI_2-excess which is not detrimental to performance but likely reduces long-term stability, facilitating autocatalytic degradation (*97-98*). In contrast, purely hybrid processing without an underlying one-step absorber requires tighter control, with an optimal concentration centered at **0.30 M**, outside of which conversion becomes incomplete or microstructural quality degrades (**Fig. S81**).

We attribute the broader processing window of the three-step route to the presence of the underlying one-step absorber, which acts as a reservoir of inorganic and organic halides. This reservoir partially buffers deviations in conversion chemistry and reduces sensitivity to small fluctuations in precursor concentration or local solvent dynamics. At the same time, one-step deposition remains intrinsically simpler and marginally more repeatable at the laboratory scale because it involves fewer sequential processing steps and therefore fewer sources of cumulative variability.

The dominant trade-off of the three-step approach arises under **under-converted conditions**, when organohalide concentration falls below the optimal window. In this regime, residual PbI_2 can persist at the buried interface, leading to increased series resistance, reduced fill factor, and more pronounced hysteresis. Fully converted or slightly under-converted three-step absorbers, however, do not exhibit systematic increases in series resistance or hysteresis relative to the one-step baseline, indicating that increased thickness does not impose transport limitations when conversion is complete. Residual PbI_2 in thicker interfacial regions is detrimental in this configuration, as it impedes carrier extraction and may accelerate degradation pathways reported in the literature.

Together, these results indicate that the three-step strategy combines an extended processing tolerance (0.25–0.35 M), while maintaining clearly identifiable and controllable failure modes when deviating from the optimal conversion window.

Supplementary Note 9: Bottom-cell architecture and surface texturing strategy

For silicon bottom-cell architecture, we employ a symmetric double-sided sub-micron-textured configuration, selected for its fabrication simplicity and compatibility with high device performance. Compared to asymmetric texturing strategies reported previously, this configuration enables streamlined processing while maintaining competitive optoelectronic characteristics. While slight increases in bottom-cell current can be achieved with asymmetric texturing, this can also enhance current mismatch between the bottom and middle cells, in some cases leading to a modest increase in fill factor. Importantly, the optical working principle of the middle reflector—namely, photon redistribution—is not intrinsically dependent on symmetric texturing and is expected to operate effectively under both symmetric and asymmetric configurations, although the precise current balance may differ due to modified back-reflection of silicon.

In our implementation, the a-Si(i)/a-Si(p) or nc-Si(p) passivation stack provides comparable surface passivation quality on both sub-micron and micron-scale textured silicon bottom cells. This behavior contrasts with prior reports suggesting sensitivity of passivation quality to texture dimensions (*99*). In our case, effective surface passivation is maintained without compromising optical or electrical performance across different texture scales (Fig. S55–S56).

To evaluate the influence of front-side texturing on triple-junction performance, we compared devices employing sub-micron pyramids (average height ≈260 nm) with those using micron-scale pyramids (average height ≈900 nm, with maxima up to ~2 µm). The micron-scale texture corresponds to the baseline configuration used for our double-side-textured bottom-cells, though it remains smaller than the ~5 µm pyramids commonly employed in industrial silicon photovoltaics. These comparisons enabled systematic assessment of light management and current balancing within the triple-junction architecture.

Supplementary Note: 10. Triple-junction integration and validation of developments

Initial triple-junction devices employed a front-flat architecture with a one-step middle-bandgap absorber and no middle reflector (Fig. S57). Incorporation of 4-HBA in the wide-bandgap absorber increased the average open-circuit voltage to 3.15 V (3.07 V control) and improved the average PCE to 25.5% (24% control), as shown in Fig. S58–S59. However, a significant current mismatch was observed between the wide-bandgap and middle-bandgap sub-cells (14.4 mA cm^{-2} vs. 9.8 mA cm^{-2}). To improve current matching, the top-cell bandgap was increased from 1.8 eV to ~1.9 eV by increasing the Br fraction to 50%, reducing excess current in the wide-bandgap cell.

Following validation of improved yield on sub-micron textures (Supplementary Note 9; Fig. S60), the one-step middle absorber was replaced with the three-step absorber. The thicker, lower-bandgap three-step absorber increased the middle-cell current density by ~0.5 mA cm^{-2}, resulting in an average absolute PCE improvement exceeding 1% (Fig. S59–S62). Comparisons across absorber thicknesses confirmed the consistent superiority of the three-step architecture.

Introduction of the SiO_x-np middle reflector on sub-micron textures further enhanced middle-cell absorption, enabling an MPP efficiency of 29.65% (Fig. S63). Optimization of the hole-transport layer

through incorporation of Me-4PACz with 4PADCB reduced series resistance (from ~16 to ~14 Ω·cm²), increasing the JV scan efficiency to 30.18% and MPP-derived efficiency to 30.1% (Fig. 3a; Fig. S64).

Finally, fine-tuning of top-cell thickness to optimize current matching (Fig. S65–S66) resulted in a stabilized triple-junction efficiency of 30.50% with J_{sc} of 12.45 mA cm^{-2} (Fig. 3a,b; Cell-2).

We note that mechanistic single-junction characterization was initially performed on 1.80 eV films during early process development, whereas the champion triple-junction employs a 1.90 eV top-cell absorber. Importantly, the reduction in bulk non-radiative losses induced by 4-HBA follows consistent trends across a range of perovskite bandgaps from 1.55 eV to 1.90 eV (Supplementary Note 5), supporting the transferability of the additive effect across halide compositions.

Supplementary Note 11: Comparison of reported stabilized efficiency procedures between in-house and certification

In-house measurements include reverse and forward JV scans performed sequentially until the cell performance stabilizes (typically within 1–5 JV cycles), followed by maximum power point (MPP) tracking for 300 s. The stabilized efficiency is reported as the average power output over the 300 s tracking period.

For independent certification at SIMIT, a standardized stabilization protocol was applied. MPP tracking was conducted for 300 s using a perturb-and-observe (P&O) method with a 1 s sampling interval. A device is considered stabilized when the rate of change in P_{max} during the final 60 s is below ±0.2% per minute, in accordance with IEC 60904-1-1:2020 practice. Only devices fulfilling this stabilization criterion are certified. The reported certified efficiencies correspond to the average P_{max} over the final 30 s of the tracking period. Our triple-junction devices satisfied the SIMIT stabilization requirements.

Hence, we note that minor differences exist between the in-house stabilization procedure and the certification protocol used for reporting stabilized efficiency, primarily in the definition of the stabilization criterion and the averaging window applied during MPP tracking.

Supplementary Note 12: Spectral mismatch factors and measurement uncertainty

According to IEC 60904-1-1:2017, the spectral matching factor Z_i for each junction should lie within 1.00 ± 0.03, while a tighter target range of ±0.01 is recommended when achievable. For our certified devices, the reported spectral mismatch factors were within ±0.015 of unity for all subcells, which is well within the IEC-required ±0.03 range and close to the recommended ±0.01 target.

The measurement uncertainty reported by SIMIT for the certified triple-junction devices is 3%. For context, recent certified perovskite–silicon tandem records listed in the NREL efficiency chart report measurement uncertainties ranging approximately from 0.9% to 3.3%. The 3% uncertainty reported by SIMIT, therefore, lies within the range of established international certification laboratories. Considering that triple-junction devices introduce an additional junction compared to perovskite–silicon tandems, thereby increasing spectral-matching and measurement complexity, this uncertainty level is consistent with state-of-the-art certification practice.

Supplementary Note 13: Origin of performance losses upon scaling to large-area triple-junction devices

The reduction in photovoltaic performance observed when scaling triple-junction solar cells from 1 cm^2 to 54 cm^2 arises primarily from passivation-related losses associated with industrially compatible design choices, with additional secondary contributions from optical shading and coating inhomogeneity (Fig. S74–S76).

The dominant loss mechanism is the open-circuit voltage and fill factor degradation. This originates from the transition to screen-printed metallization and industrial bottom-cell architectures. The use of an industrially relevant silicon bottom cell (minority carrier lifetime 3–4 ms at 5×10^{15} cm^{-3}, compared to 5–6 ms in laboratory-optimized devices) with a-Si-based selective contacts reduces open-circuit voltage compared to nc-Si-based reference architectures. In addition, screen-printed metallization, followed by curing at 130 °C in ambient for 10 minutes, likely induces partial degradation of top- and middle-cell passivation, contributing further to V_{OC} and FF losses.

In the middle sub-cell, reduced shunt resistance becomes significant in large-area devices. The JV characteristics of the 54 cm^2 devices exhibit leakage currents that artificially increase the apparent J_{SC} while substantially degrading FF. Evaluating the JV curve at the summed sub-cell open-circuit voltage ($V \approx V_{OC,bot} + V_{OC,top} \approx 2$ V) yields a corrected effective J_{SC} of approximately 10.8 mA cm^{-2}, which closely matches that of the 4 cm^2 device. This confirms that the current generation remains largely preserved and that FF degradation, rather than photocurrent loss, dominates the scaling penalty.

Secondary losses arise from optical and materials-related adjustments made for manufacturability. In the top cell, increased TCO thickness (40 nm ITO versus 25 nm IZrO) and reduced $PbCl_2$ and MACl content were introduced to improve process robustness and compatibility with screen printing. These modifications introduce an optical–electrical trade-off, slightly reducing J_{SC} while partially improving FF. Additional minor optical losses originate from increased finger shadowing, which rises from 1.5% in 1 cm^2 devices to 2.8% in 54 cm^2 devices. Because triple-junction devices operate at approximately 11 mA cm^{-2} (rather than ~20 mA cm^{-2} as in tandems), future optimization of the front-grid design can further reduce metallization shading.

Finally, spatial inhomogeneity in large-area perovskite coatings and the removal of hydrophobic Me-4PACz from the SAM (to improve wetting uniformity) increase interfacial recombination and reduce charge selectivity, contributing additional V_{OC} and FF penalties. Overall, the scaling-induced efficiency loss is predominantly transport- and resistance-limited, while optical and coating-related losses play secondary roles.

Importantly, the observed performance of large-area triple-junction devices is also influenced by limited statistical sampling and process maturity. While more than 200 devices were fabricated and optimized at the 1 cm^2 scale on 1-inch substrates, only 21 pixels across 3 wafers were realized for the 4 cm^2 architecture, and 6 devices for the 54 cm^2 architecture. This substantially lower device count reflects an earlier stage in the process learning curve, where variability, yield losses, and incomplete optimization are expected to have a more pronounced impact on average device performance.

Supplementary Note 14: Extended stability analysis and degradation mechanisms

Temperature-dependent operational stability of single-junction devices

Operational stability of unencapsulated wide-bandgap (WBG) single-junction devices (with and without 4-HBA, with initial average efficiency of 11.5% for the control and 12.4% for the target devices measured at 40°C) was evaluated under continuous maximum power point tracking (MPPT) in nitrogen. The device temperature was initially maintained at 40 °C for 1,675 h. After observing only minor degradation, the temperature was increased to 60 °C for 225 h and subsequently to 80 °C until the end of the 4,500 h test. This stepwise temperature ramp enabled the extraction of temperature-dependent degradation behavior (Fig. S77).

Both control and 4-HBA devices retained approximately 70% of their initial PCE after ~2,040 h. However, after ~2,250 h, control devices exhibited accelerated decay, leading to an S-shaped degradation profile. Such behavior is consistent with autocatalytic degradation pathways reported for halide perovskites, where once a critical density of degradation nuclei forms, the reaction rate increases nonlinearly. In contrast, 4-HBA-modified devices did not exhibit this transition and instead showed a more gradual decay.

After 4,500 h, control devices retained 24.3% of their initial PCE (17.9% average), whereas 4-HBA devices retained up to 39.5% (30% average). Optical images obtained after MPPT revealed more extensive formation of a PbI_2-rich yellow phase (or δ-phase) in control devices (Fig. S77), consistent with the higher Pb^0 content observed in XPS for untreated films. The bulk distribution of 4-HBA is therefore likely to delay the nucleation of thermally activated degradation pathways.

Damp heat stability of encapsulated triple-junction devices

Encapsulated triple-junction devices (glass/glass configuration, Fig. 4c) were subjected to damp heat testing at 85 °C and 85% relative humidity. Two high-performance devices ("Cell-4" and "Cell-5") were monitored for 500 h (Fig. 4d).

After 500 h, relative PCE losses were 13.8% and 22.9%, respectively. Optical inspection of the more strongly degraded device revealed the formation of a localized point-like defect within the active area (Fig. S78), consistent with a PbI_2-rich phase. The degradation remained spatially localized rather than catastrophic, suggesting that failure was driven by nucleation of isolated degradation centers rather than uniform bulk decomposition.

Outdoor performance monitoring

Two encapsulated triple-junction devices were deployed outdoors in Neuchâtel, Switzerland, from February 6 to June 3, 2025 (Fig. S79). The performance ratio (PR) was used to evaluate field stability.

"Cell-6" maintained its initial PR during the first two months, when ambient temperatures were relatively low (Fig. S80). As temperatures increased toward late spring, the PR gradually decreased to ~0.70. "Cell-7" began operation at a lower initial PR (~0.525) but maintained this value throughout the monitoring period despite increasing cell temperatures.

Table.S1 sums up the calculated binding energies. Structures showing adsorption of Pb^{2+} ions to HBA are given in Fig. 1 of the main text. We employ the Makov-Payne correction (*100, 101*) to handle long-range electrostatic interactions between periodic images. We found that the remaining errors closely follow a $1/L^3$ behavior (L being the box length), and we use this to extrapolate the adsorption energies to infinite L. Using the same extrapolation procedure, we have also investigated the adsorption of FA^+ and Cs^+ cations to HBA. The binding motive for Cs^+ is the same as for the case of Pb^{2+}, that is via formation of Cs-N bonds (not shown). FA^+ also prefers the same binding site, molecular structures are shown in **Fig.S6** (a)-(c). For interaction of a (neutral) $CsPbI_3$ molecule with HBA, the chosen 20 Å x 20 Å x 20 Å box is large enough to reduce the finite size error well below 0.1 eV. Binding energies are given with respect to isolated HBA and $CsPbI_3$ molecules. In the isolated $CsPbI_3$ molecule, Pb and Cs are coordinated via three shared I-bonds. $CsPbI_3$ can simply attach to HBA by occupying the aforementioned cationic binding site, forming a Cs-N bond. This is the most stable configuration for 3-HBA and 4-HBA, see **Fig.S6** (e) and (f). Such a binding motive exists also for 2-HBA as a metastable state. However, due to the spatial proximity of the NH_2 and OH groups in 2-HBA, $CsPbI_3$ can interact with both of them simultaneously, see **Fig.S6** (d) (notice also the rotation of the OH group). This slightly increases the adsorption energy compared to the other HBA positional isomers, see **Table.S1**.

Table.S1 Calculated binding energies for HBA interacting with Pb^{2+}, FA^+ and Cs^+ ions, as well as with a $CsPbI_3$ molecule.

	2-HBA	3-HBA	4-HBA
Pb^{2+}	6.8 eV	6.8 eV	6.8 eV
FA^+	1.7 eV	1.6 eV	1.6 eV
Cs^+	1.3 eV	1.2 eV	1.2 eV
$CsPbI_3$	1.2 eV	1.0 eV	0.9 eV

Numerical calculations based on equivalent circuit modeling

Table.S2

Input parameters and PV performance parameter results of numerical calculations.

Condition	Rs total (ohm.cm^2)	Top-cell current (mA/cm^2)	Middle-cell current (mA/cm^2)	Bottom-cell current (mA/cm^2)	V_{OC} (V)	J_{SC}(mA/cm^2)	FF (%)	PCE (%)
**Baseline**	13.5	12.75	11.00	17.00	3.110	11.018	86.23	29.55
**Rs + 1 ohm.cm^2**	14.5	12.75	11.00	17.00	3.110	11.018	85.90	29.44
**3-step absorbers**	14.5	12.75	11.75	16.25	3.110	11.767	84.60	30.98
**Middle-reflector + 3-step**	14.5	12.75	12.25	15.50	3.110	12.265	82.92	31.64
**Matched**	14.5	12.50	12.50	15.50	3.110	12.500	80.62	31.36

Table.S3

PV performance parameters of devices measured in Fraunhofer ISE and EPFL.

Cell number	Measured in -	V_{OC} (V)	FF (%)	J_{SC} (mA/cm^2)	PCE (%)
3052-8	Fraunhofer ISE	3.12	81.80	11.46	29.28
3052-8	EPFL	3.02	81.27	12.10	29.80
3052-9	Fraunhofer ISE	3.09	82.97	11.29	28.96
3052-9	EPFL	3.03	82.79	12.03	30.23
3054-1	Fraunhofer ISE	3.10	82.40	11.12	28.45
3054-1	EPFL	3.03	82.81	11.60	29.07
3054-5	Fraunhofer ISE	3.05	78.53	11.47	27.46
3054-5	EPFL	3.03	81.31	12.00	29.55
3054-6	Fraunhofer ISE	3.13	80.32	10.98	27.60
3054-6	EPFL	2.99	81.62	11.67	28.47
3054-7	Fraunhofer ISE	3.10	81.90	11.42	29.00
3054-7	EPFL	3.04	81.69	11.94	29.60
3061-2	Fraunhofer ISE	3.07	83.06	11.11	28.35
3061-2	EPFL	3.02	83.62	11.70	29.53
3061-6	Fraunhofer ISE	3.13	78.46	11.49	28.27
3061-6	EPFL	3.06	83.24	11.71	29.86
3061-9	Fraunhofer ISE	3.11	81.52	11.30	28.67
3061-9	EPFL	3.06	83.24	11.71	28.86
3115-8	Fraunhofer ISE	3.12	83.26	11.03	28.67
3115-8	EPFL	3.05	83.70	11.58	29.07

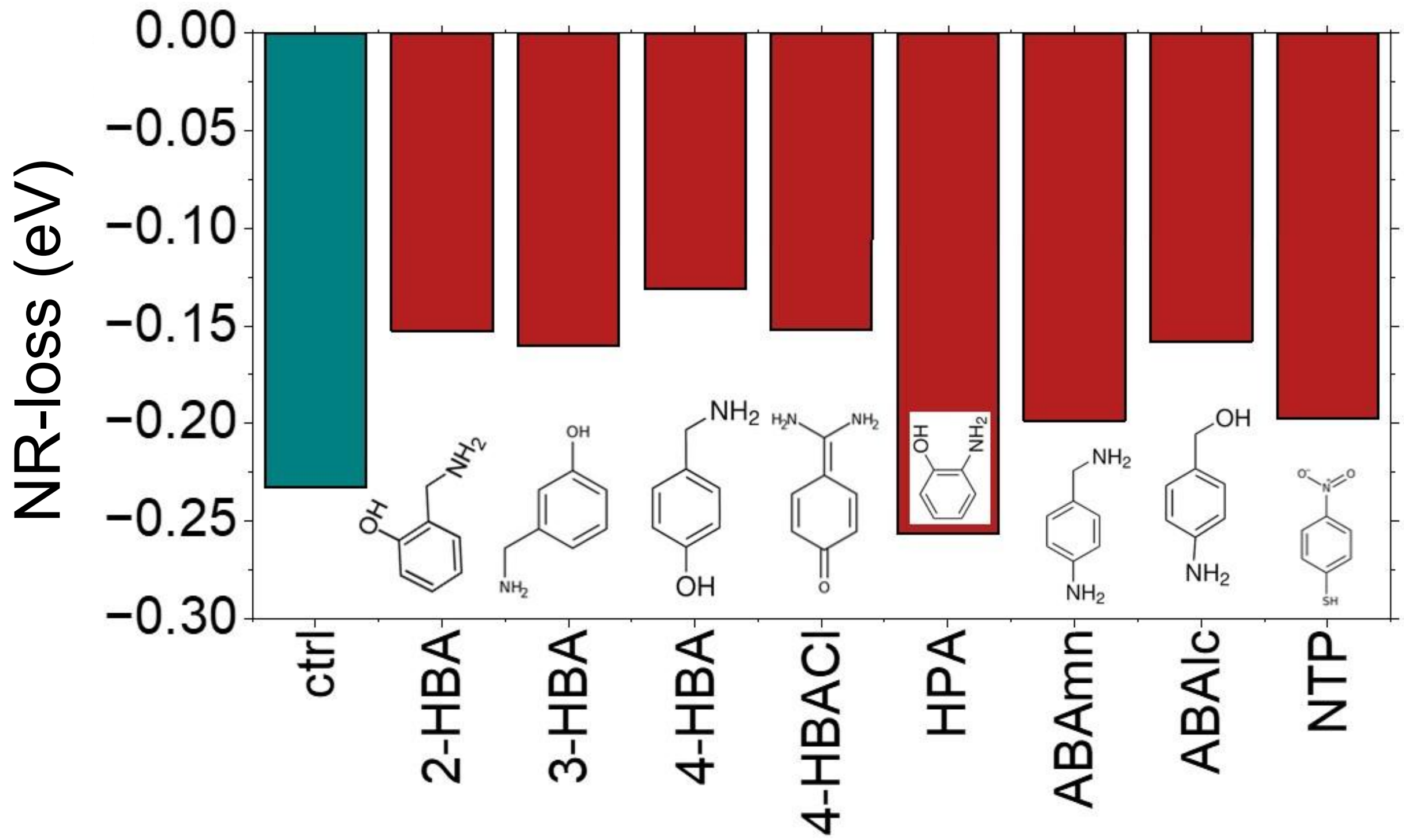

Figure S1. Non-radiative losses of the stack ITO/Me-4PACz/SiOx-np(0.1%)/perovskite with different additives.

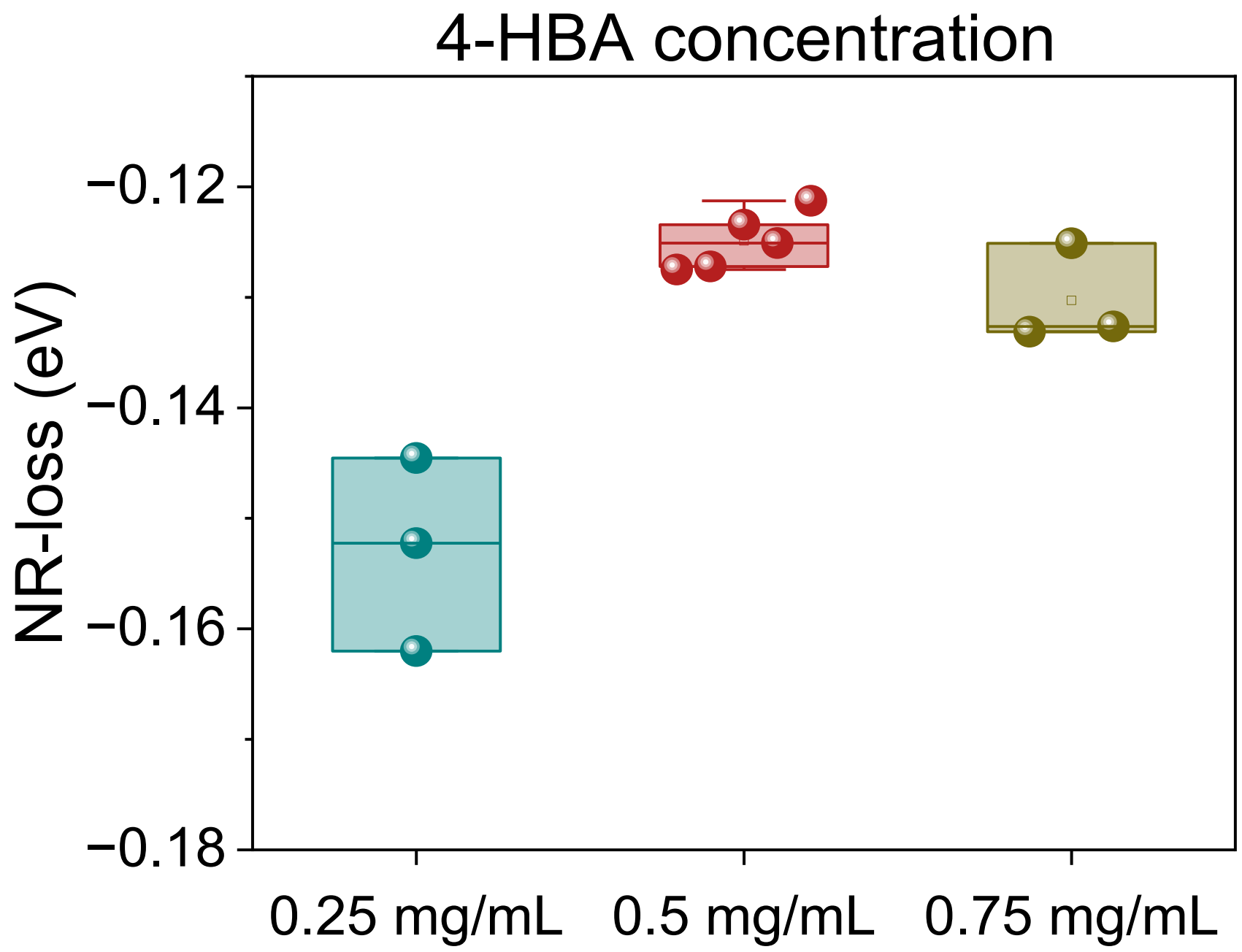

Figure S2. HBA-concentration optimization on half-cell stacks, ITO/Me-4PACz/SiOx-np(0.1%)/perovskite.

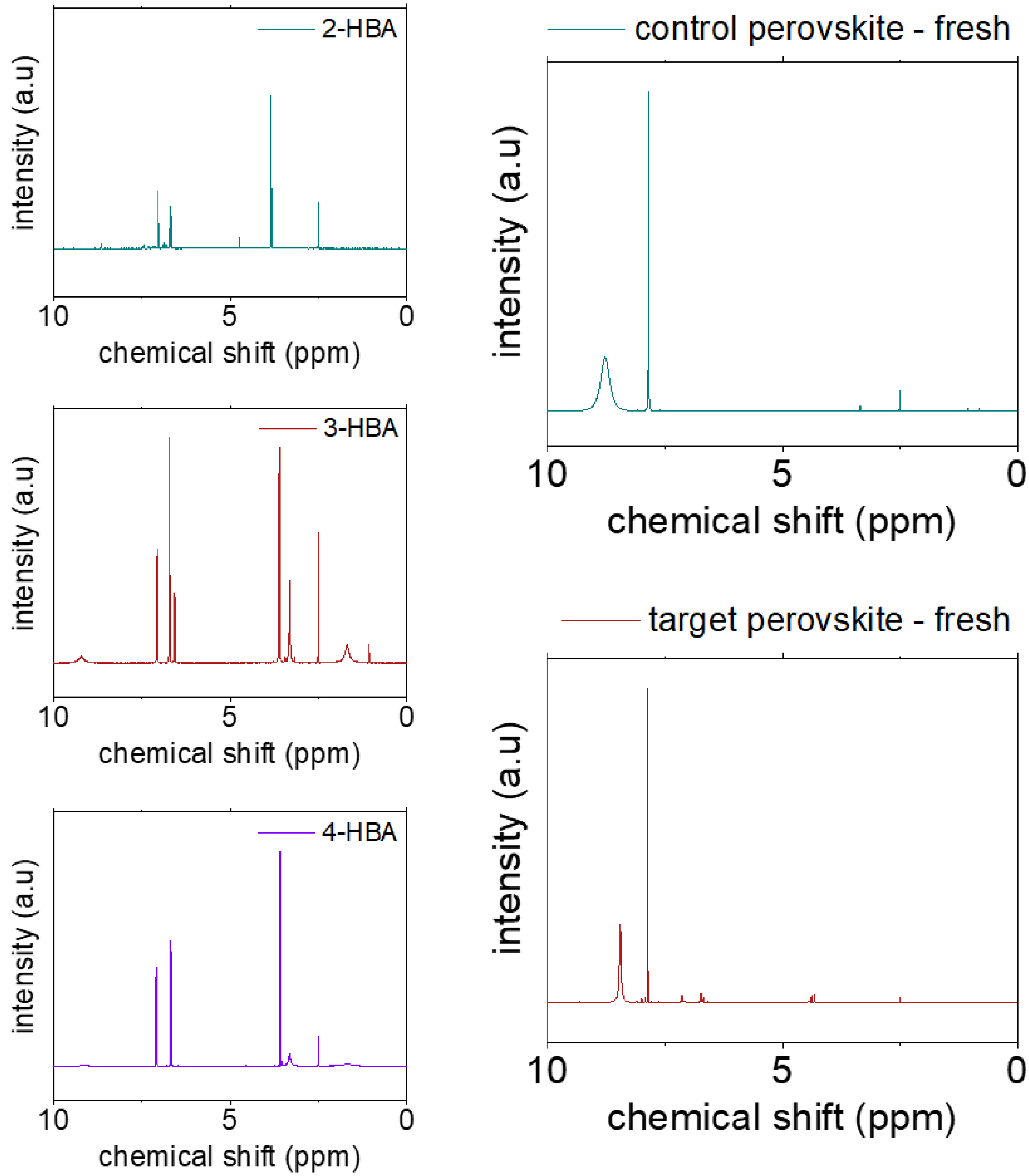

Figure S3. NMR spectra of HBA-derivatives, control and target perovskite (fresh).

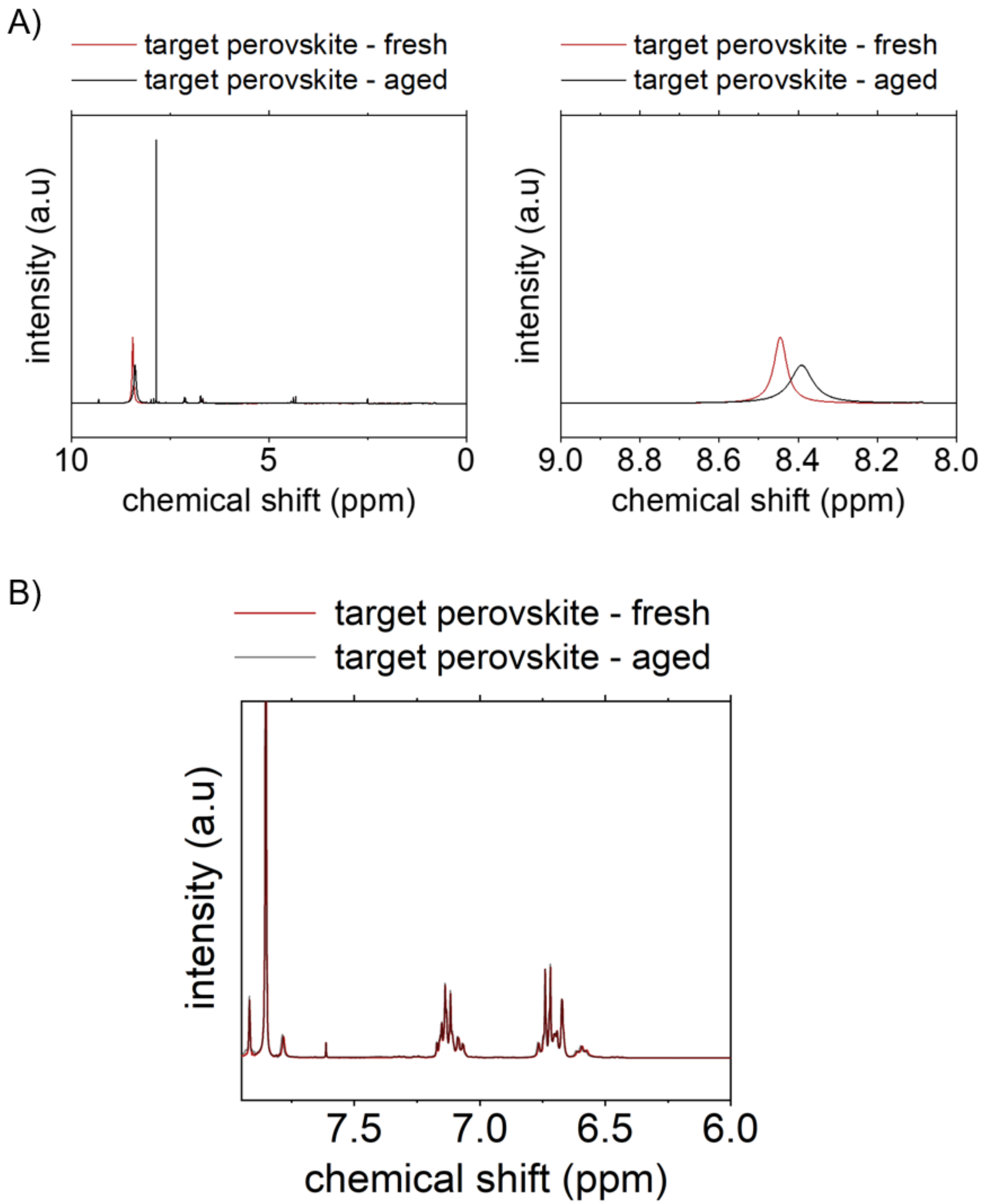

Figure S4. A) NMR spectrum of target perovskite (fresh and aged). Second graph with zoom-in to the peak at 8.45 ppm (-NH2 of FAI). B) NMR spectrum of target perovskite (fresh and aged), highlighting perfect overlap in the non-exchangeable aromatic peaks of HBAs.

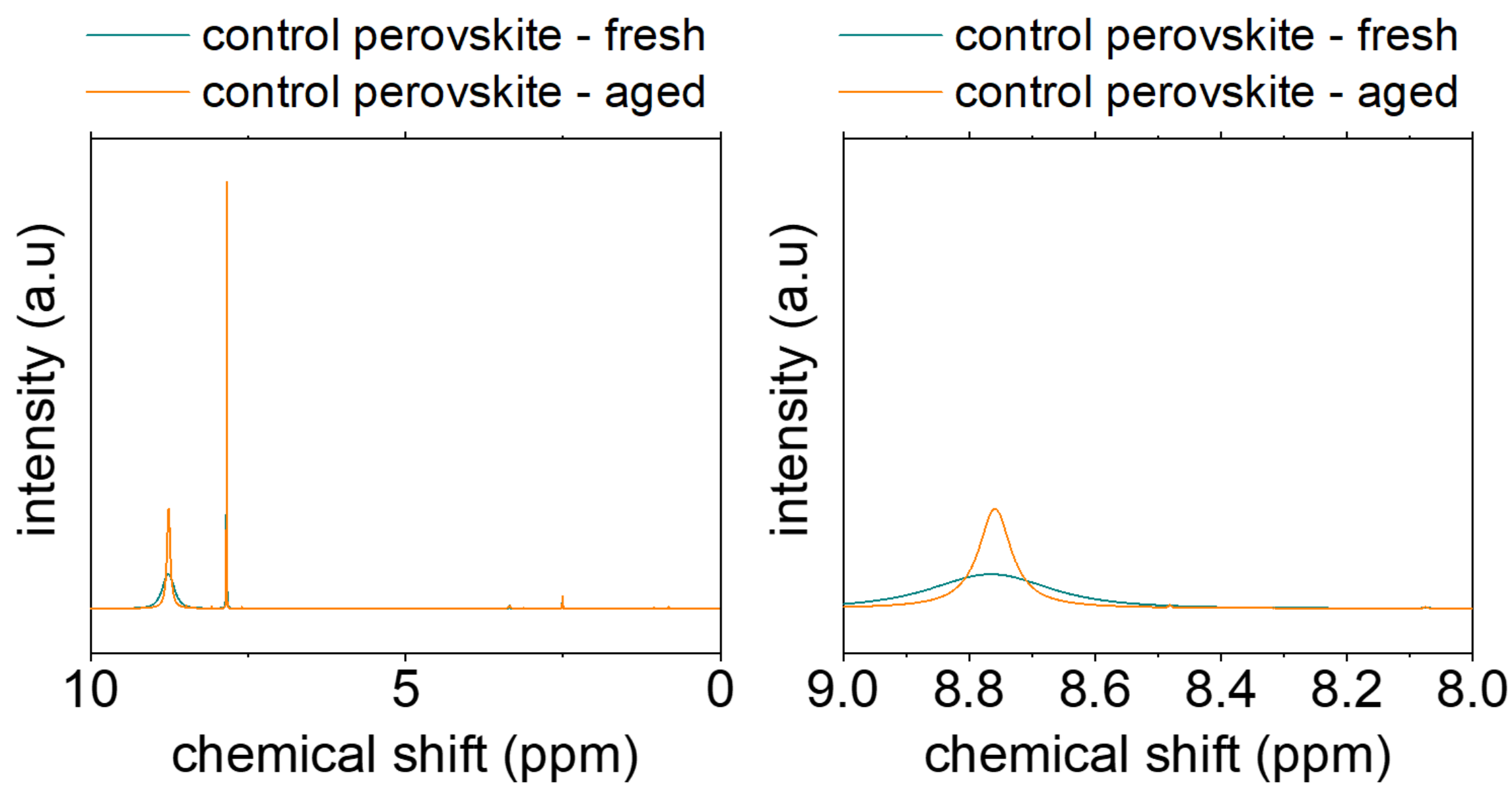

Figure S5. NMR spectrum of the control perovskite (fresh and aged). Second graph with zoom-in to the peak at 8.75 ppm (-NH2 of FAI).

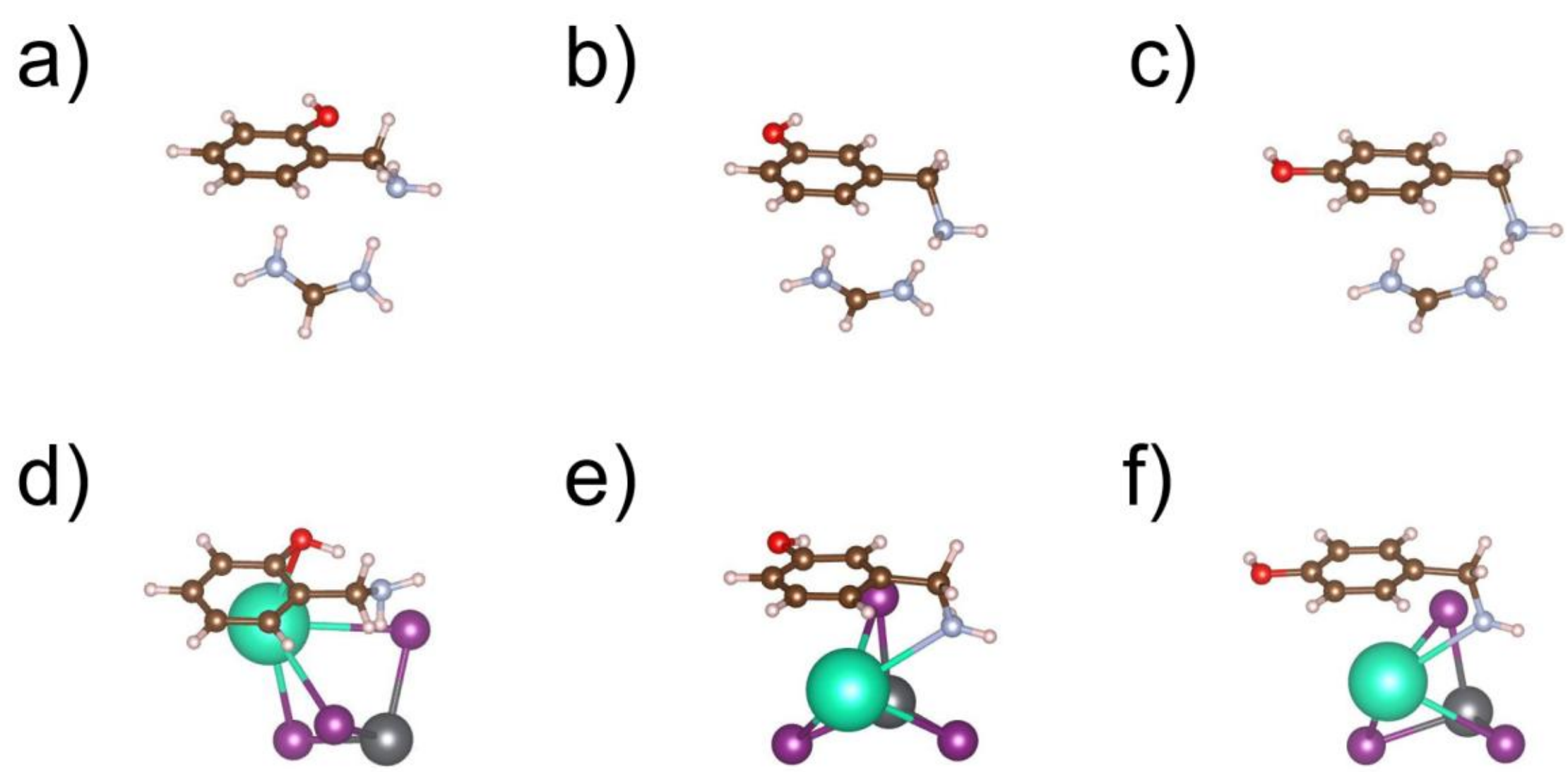

Figure S6. (a)-(c) Interaction of 2-3-4- HBA with a FA+ cation. (d)-(f) Interaction of 2-3-4-HBA with the model perovskite CsPbI3. Binding energies calculated with DFT are given in Table.S1. Carbon atoms are shown as brown spheres, hydrogen as white, nitrogen as blue, oxygen as red, lead as black, and cesium as green (*100*).

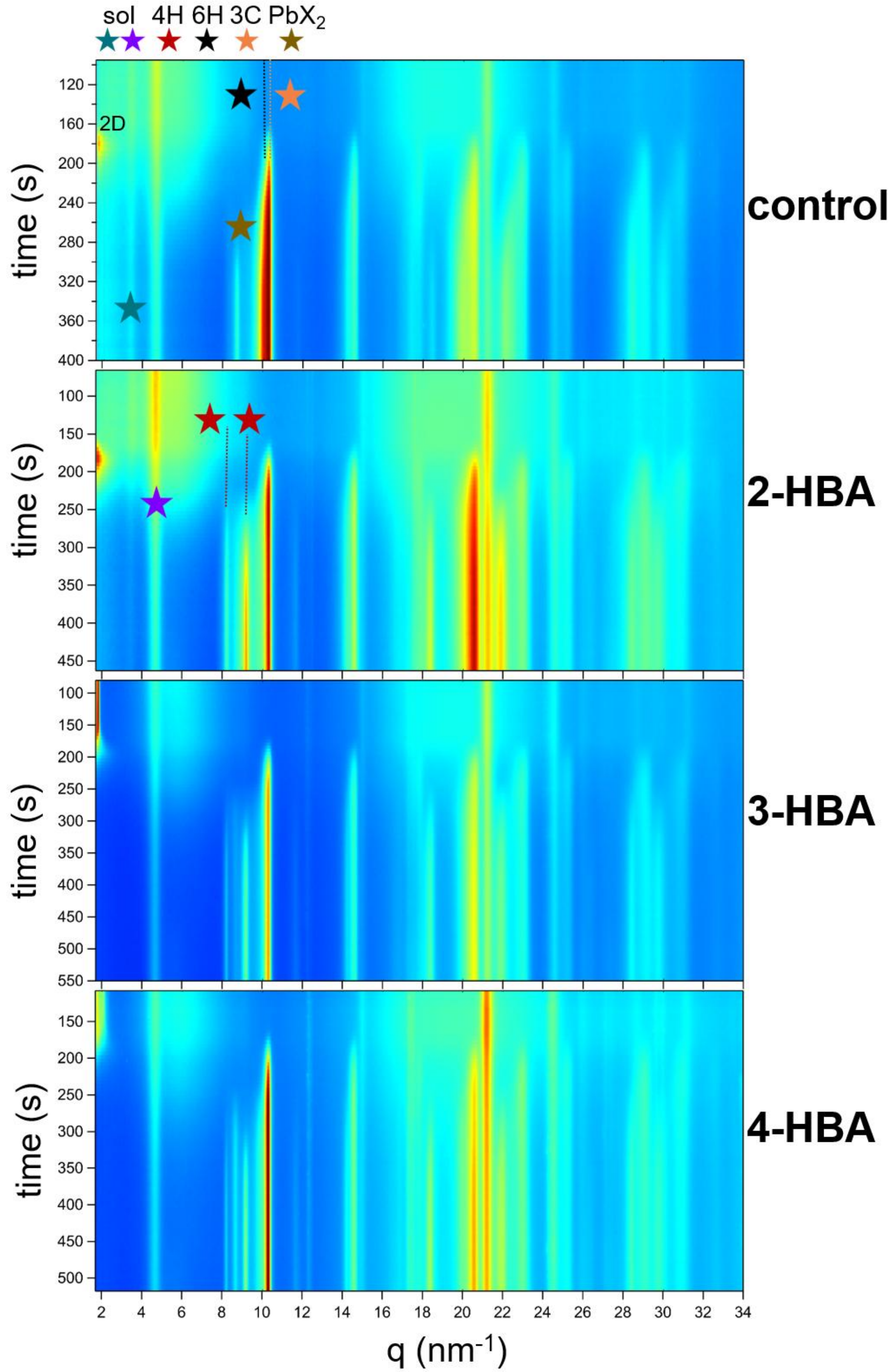

Figure S7. In-situ GIWAXS measurements during spinning.

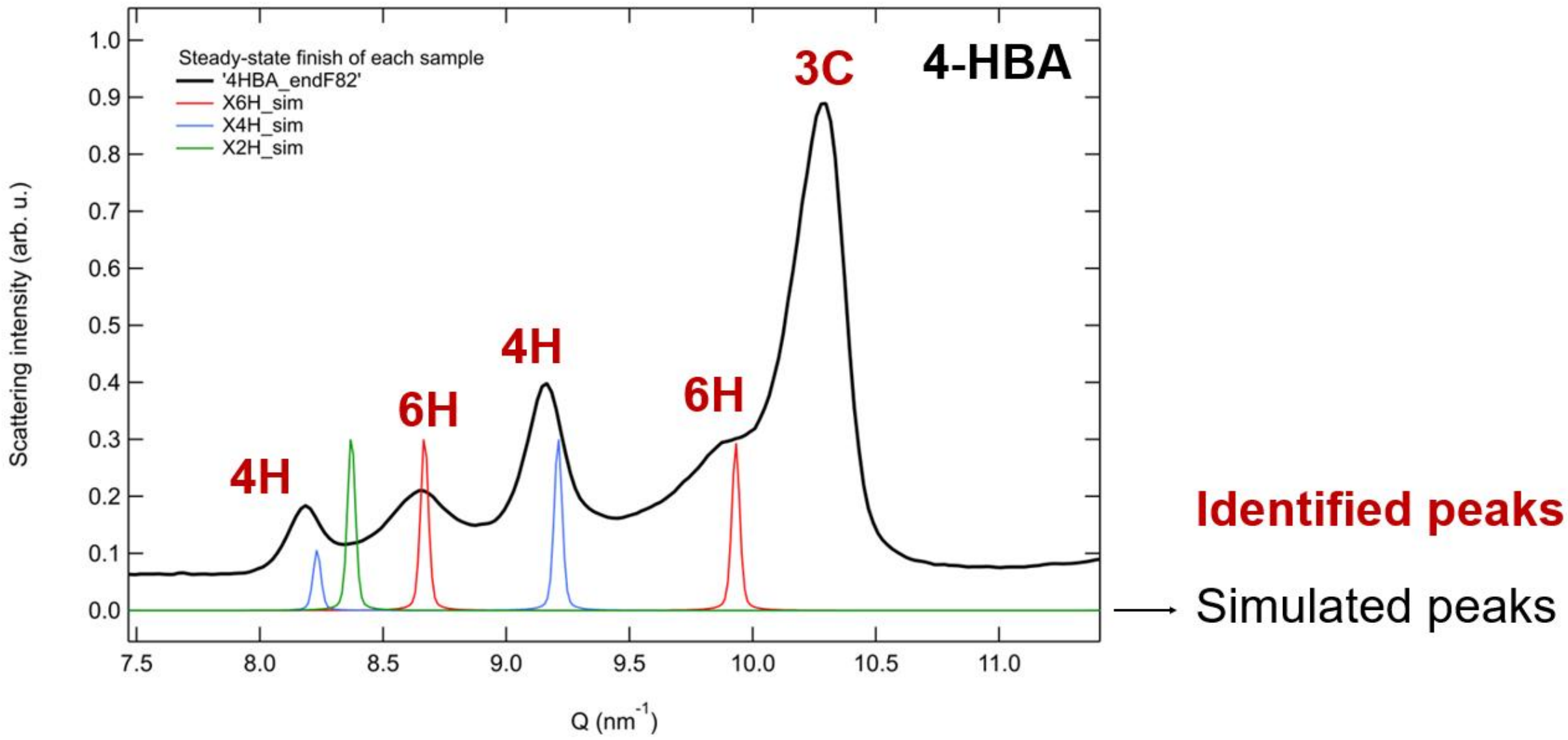

Figure S8. Simulated and identified peaks for the in-situ GIWAXS measurements.

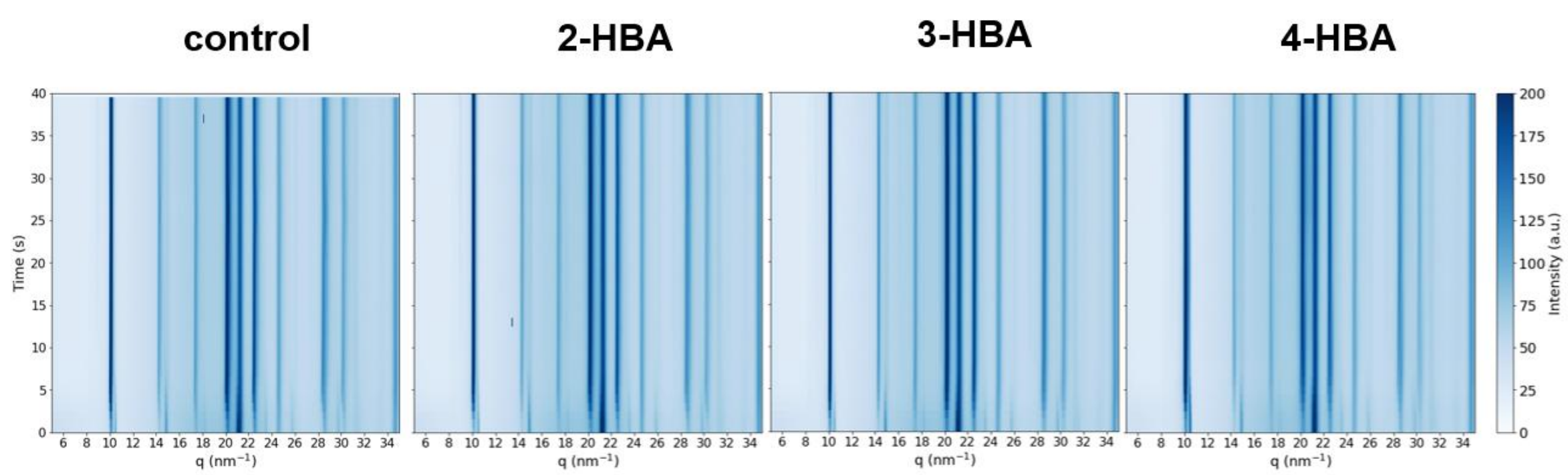

Figure S9. In-situ GIWAXS measurements during annealing.

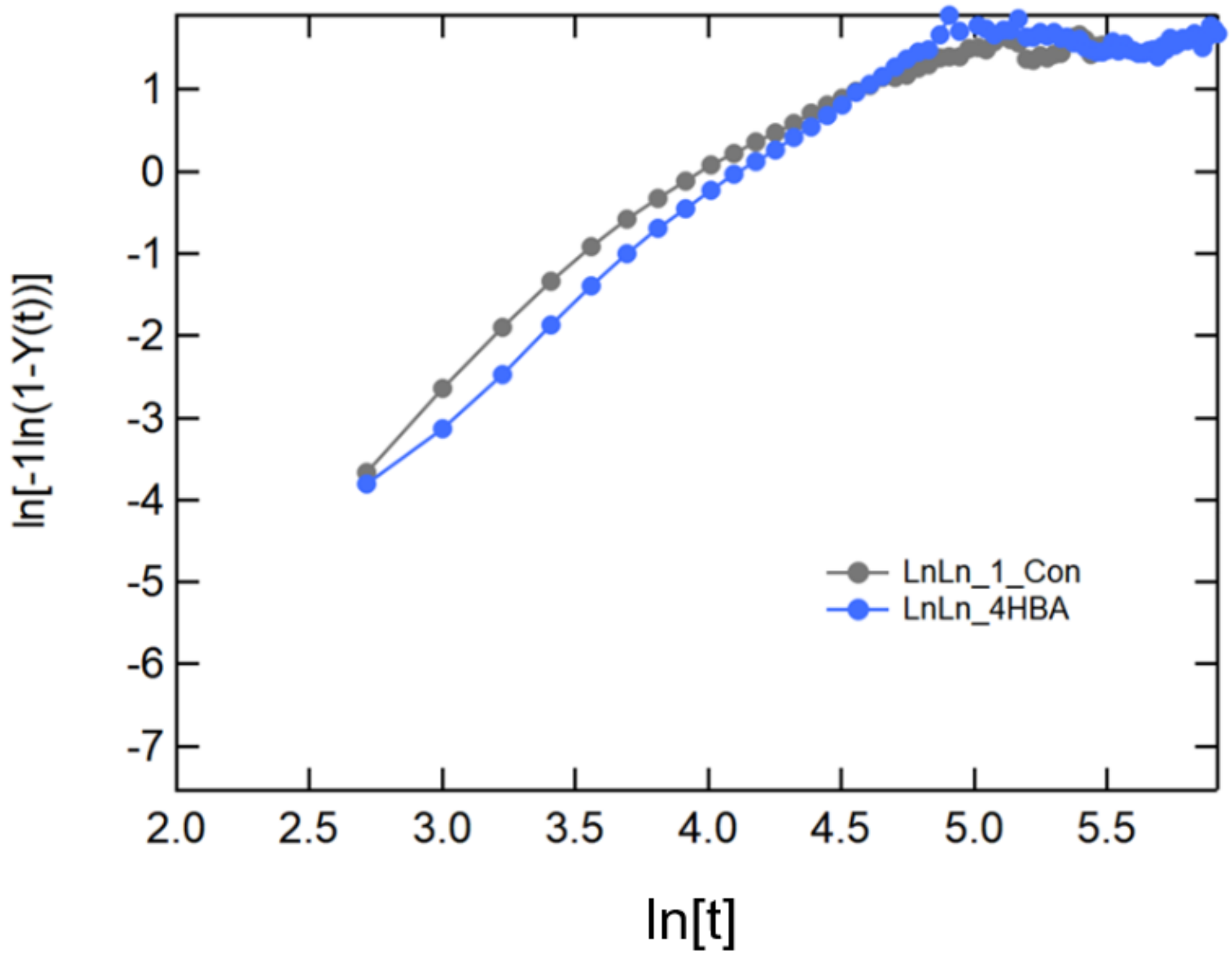

Figure S10. Avrami plots for control and 4-HBA extracted from **in-situ annealing** experiment.

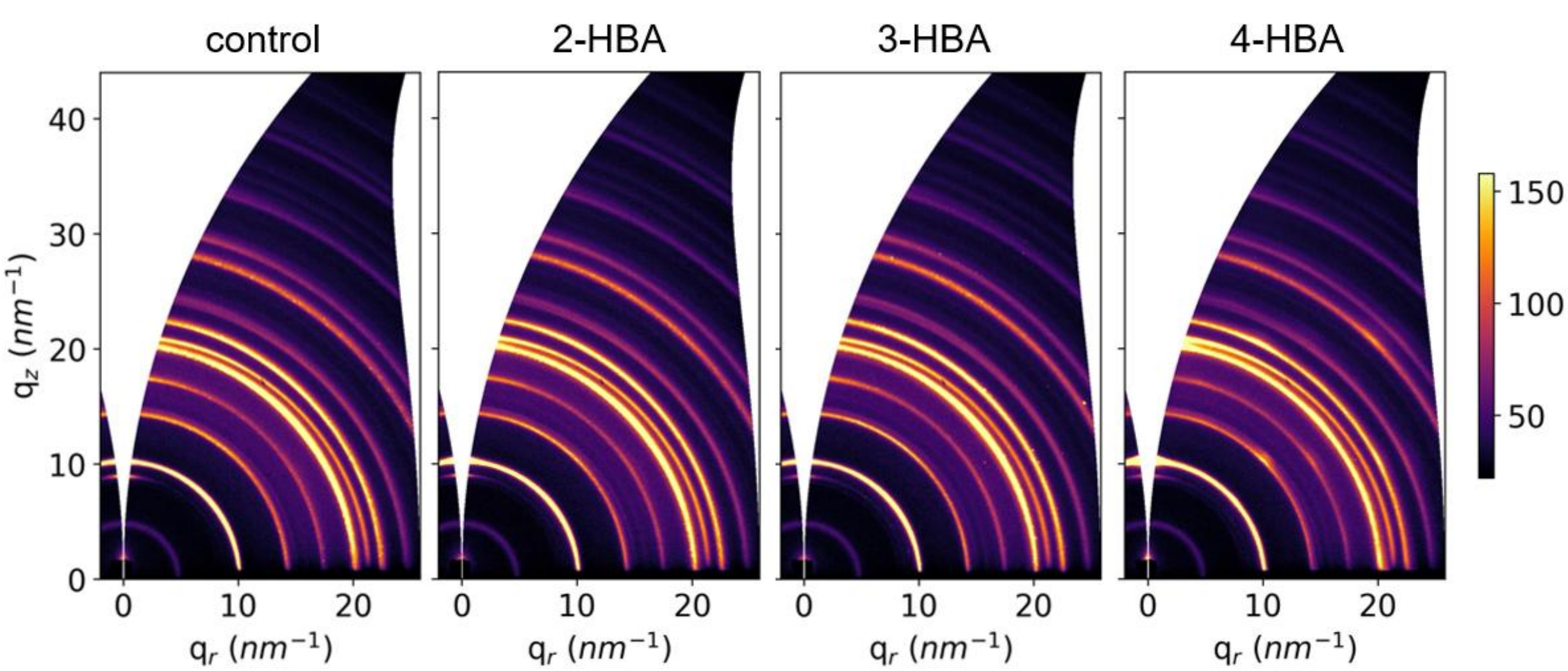

Figure S11. Ex-situ GIWAXS measurements after film formation.

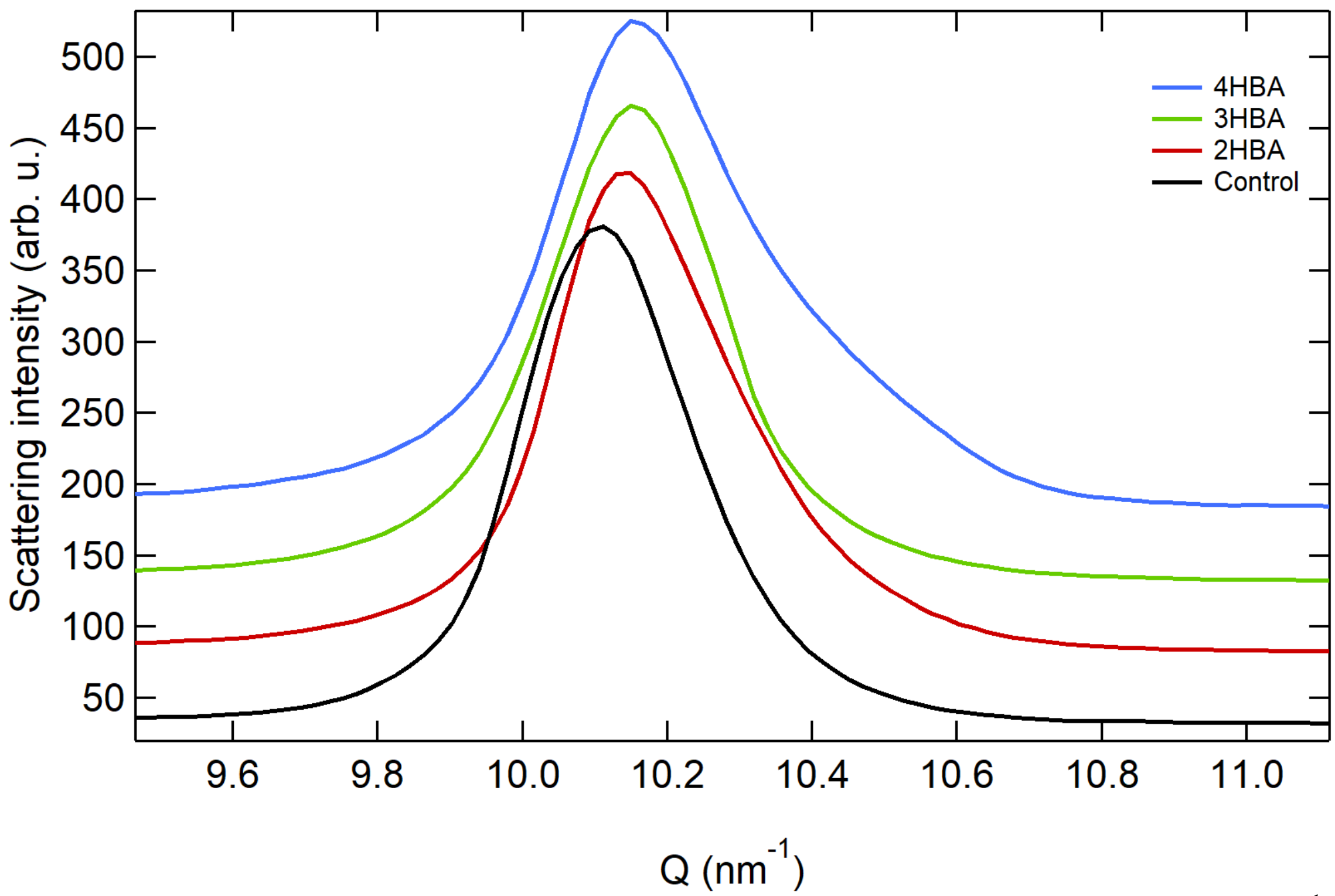

Figure S12. Ex-situ GIWAXS measurements, shift in the main perovskite peak at 10.1 nm^{-1}, indicates reduced unit cell volume. Ex-situ GIWAXS measurements, shift in the main perovskite peak at 10.1 nm^{-1}, indicates reduced unit cell volume.

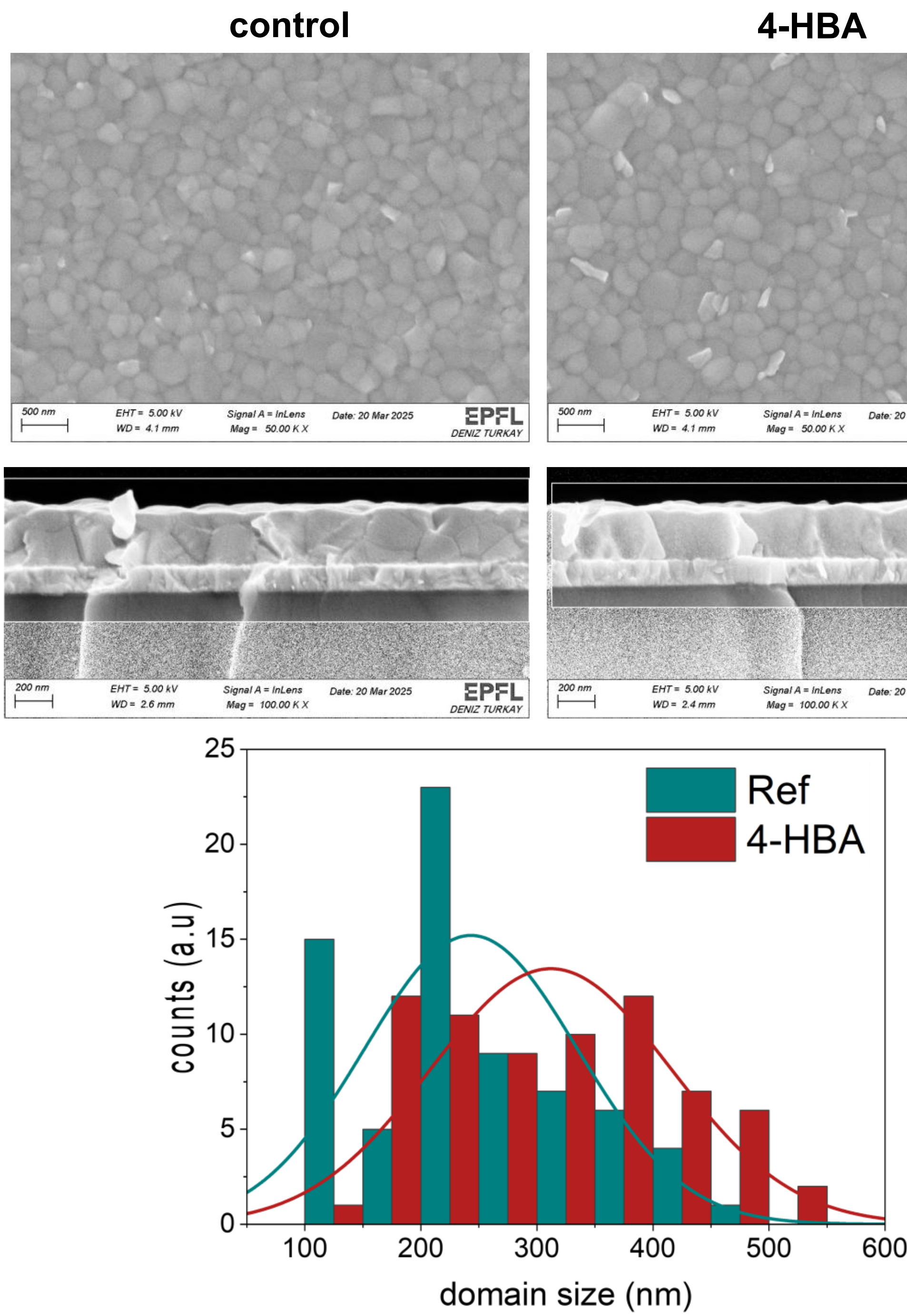

Figure S13. Top-view and cross-section SEM of ITO/perovskite samples, with and without 4-HBA additive. Domain size analysis from the top-view SEM.

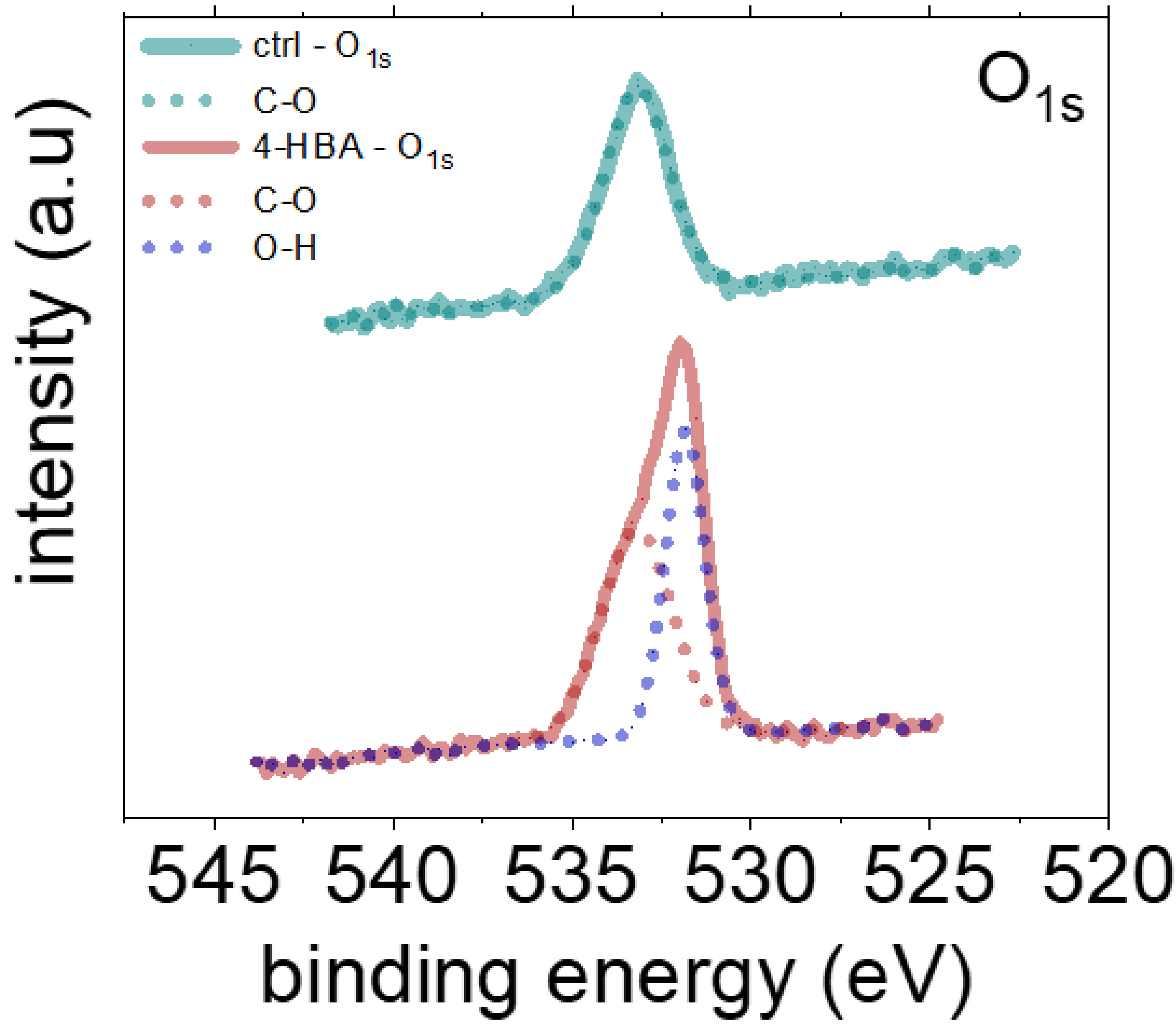

Figure S14. O_{1s} XPS spectrum of control and 4-HBA devices, the origin of C-O contribution is attained to atmospheric contamination.

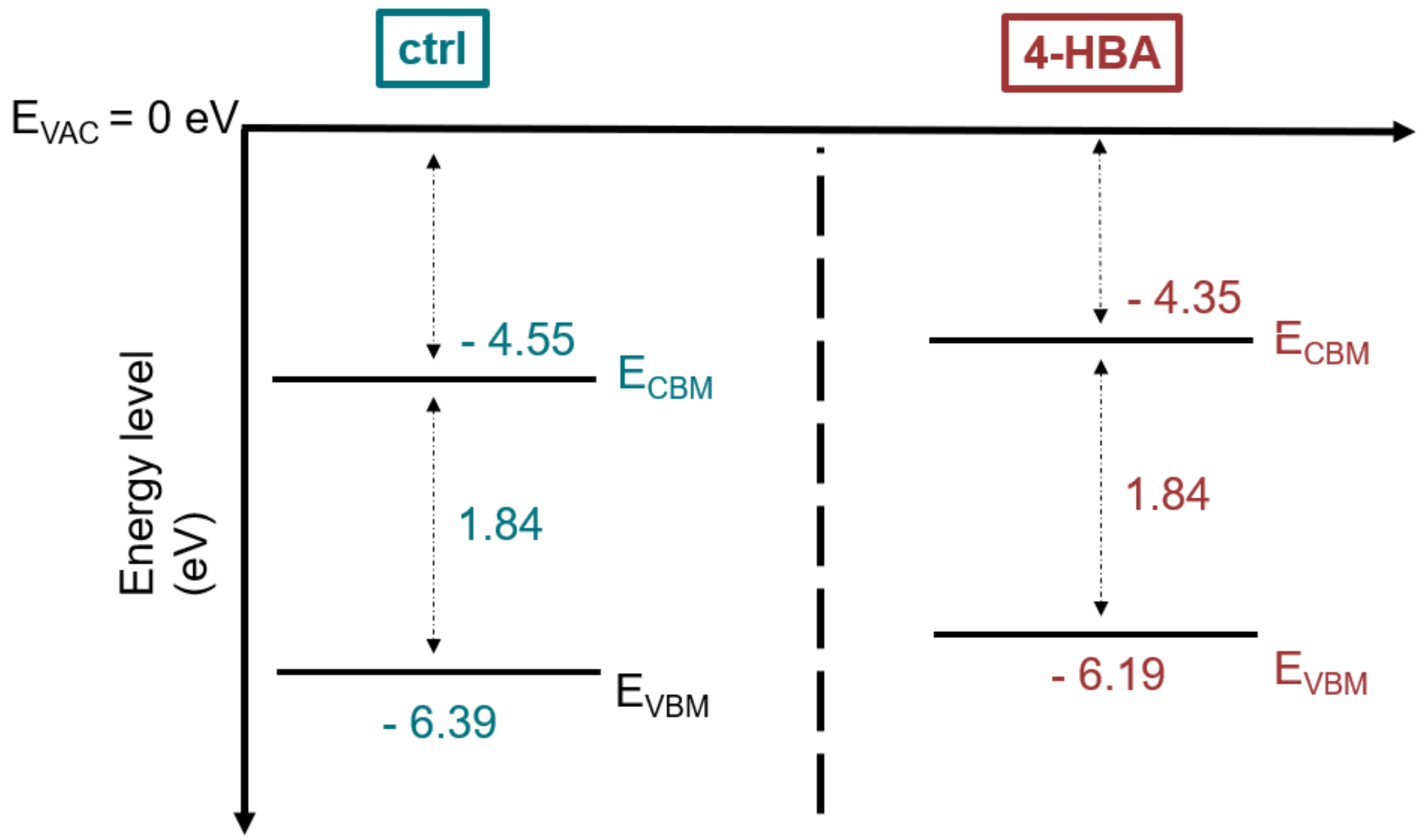

Figure S15. Changes in the band energetics with the 4-HBA additive.

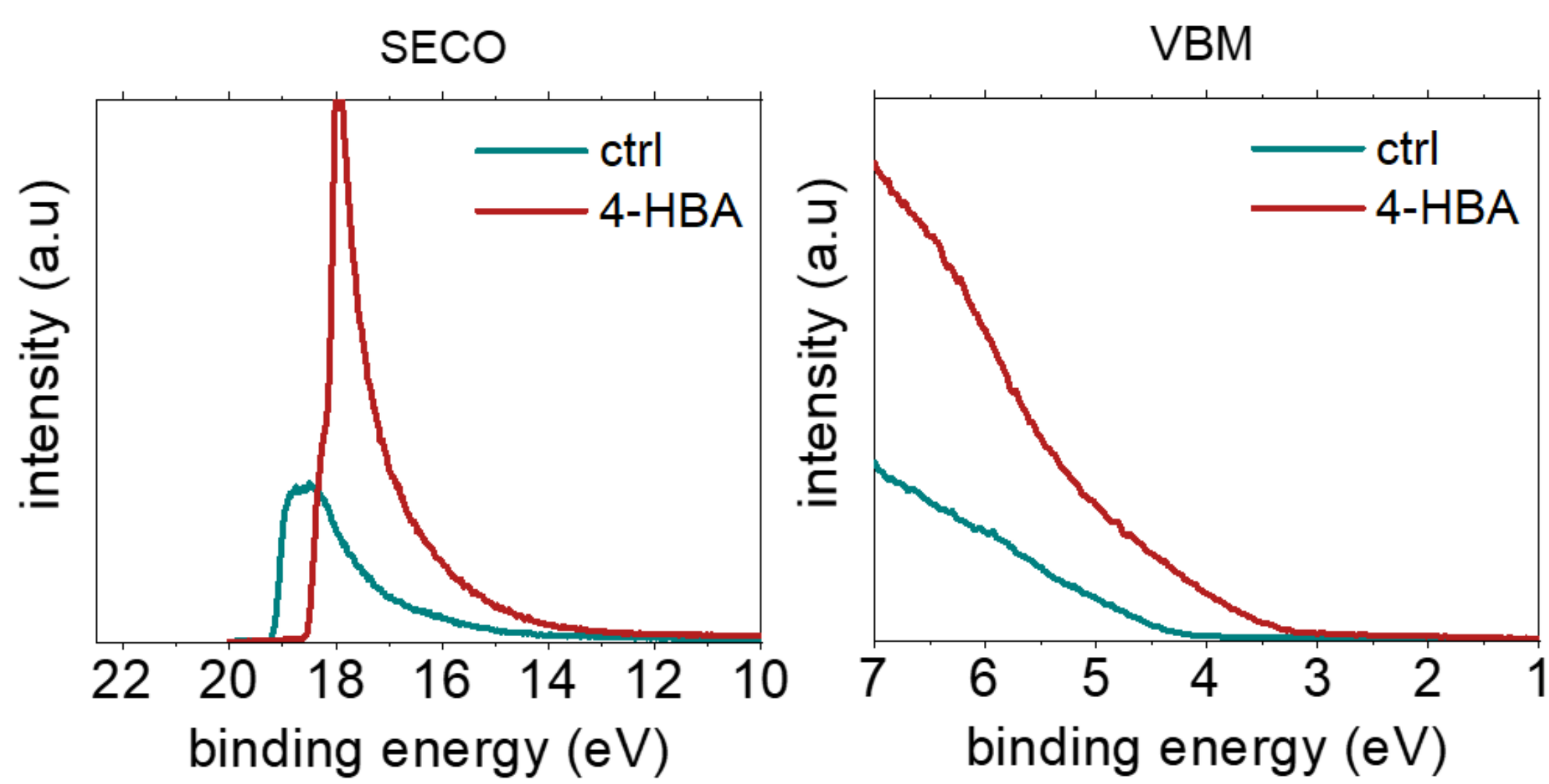

Figure S16. SECO and VBM of control and 4-HBA devices.

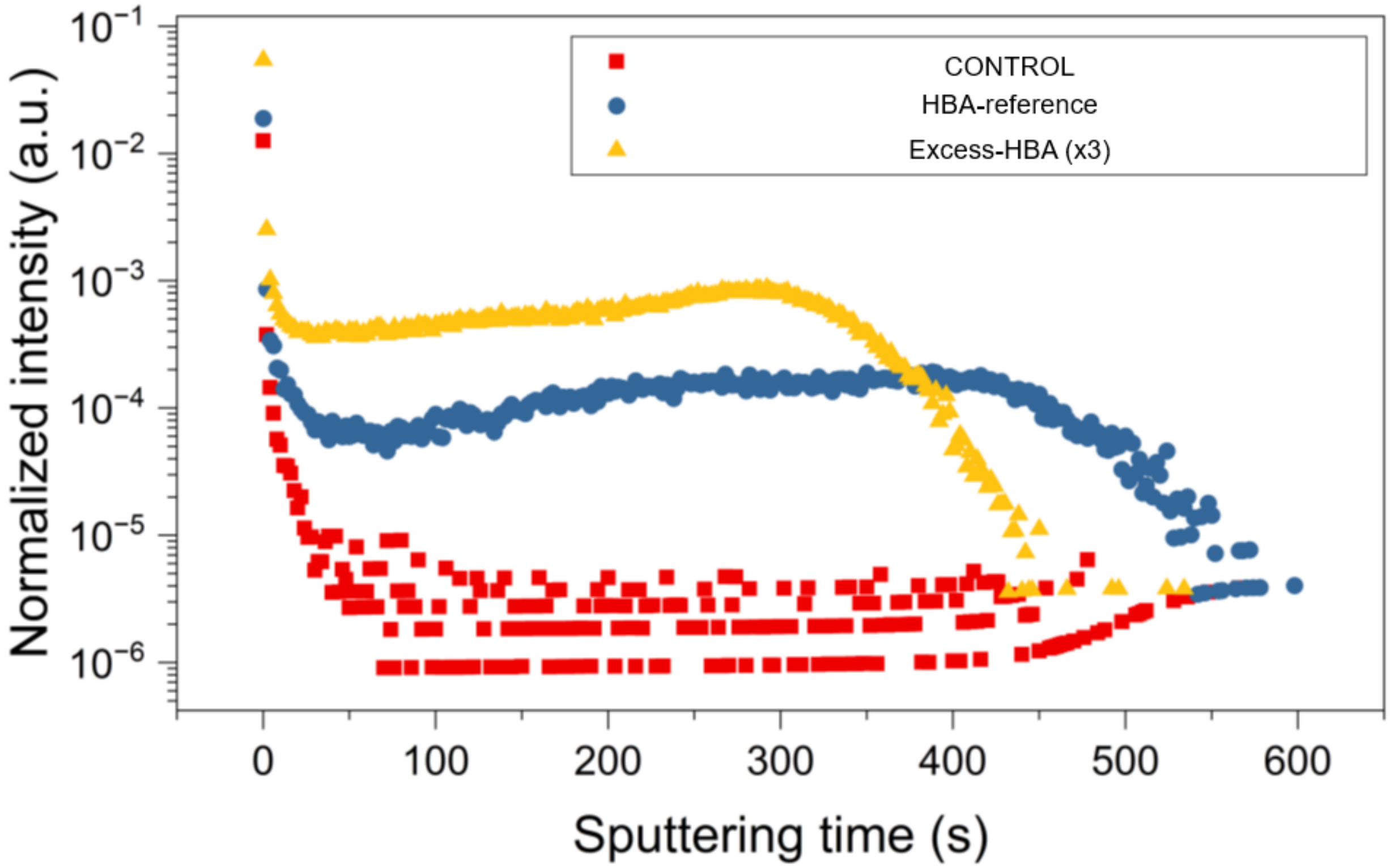

Figure S17. TOF-SIMS results for the HBA-peak.

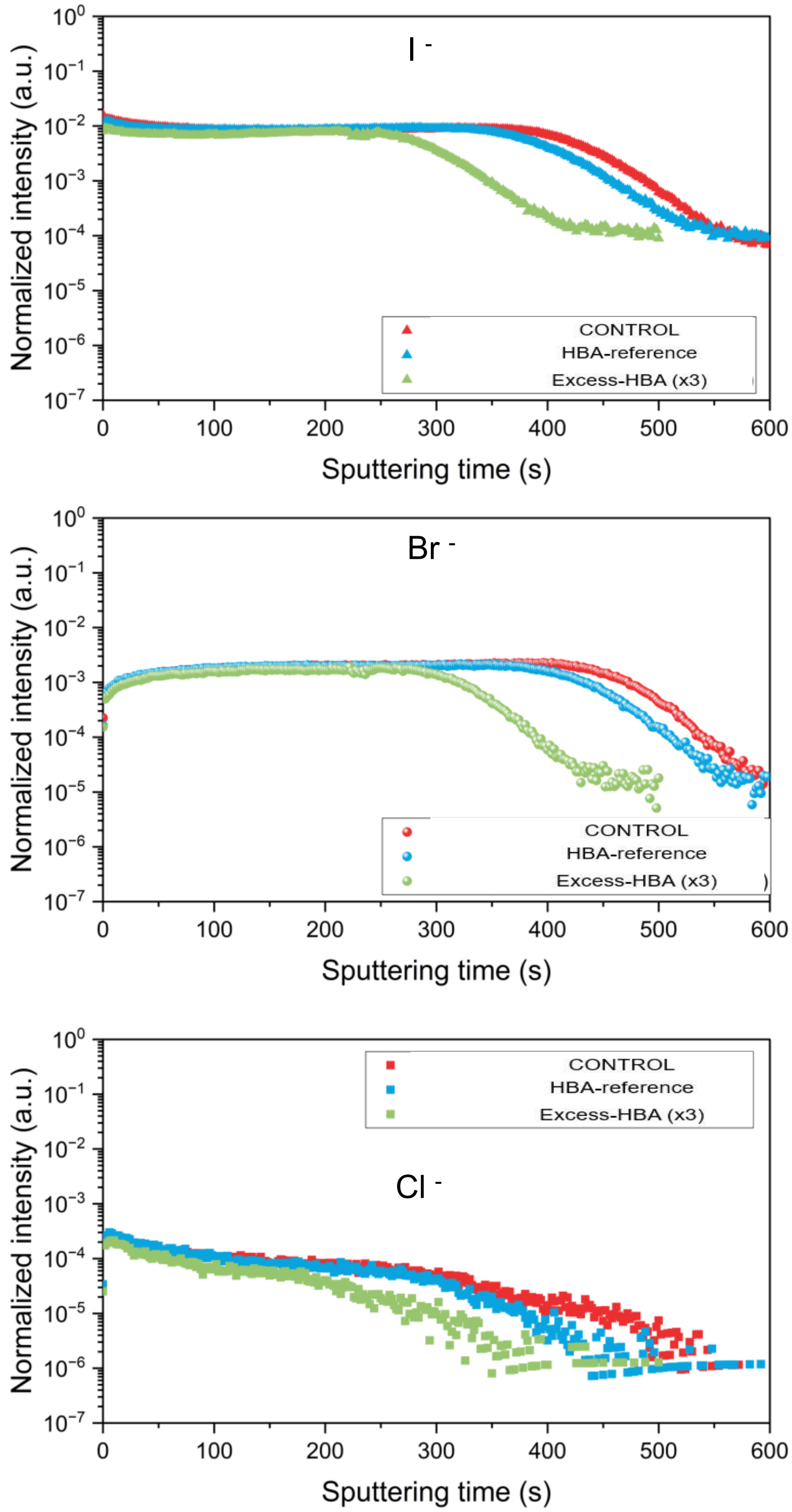

Figure S18. TOF-SIMS results for different halides. The faster fading of the signal with increasing HBA concentration is a result of dilution of the perovskite ink (and hence lower film thickness).

A)

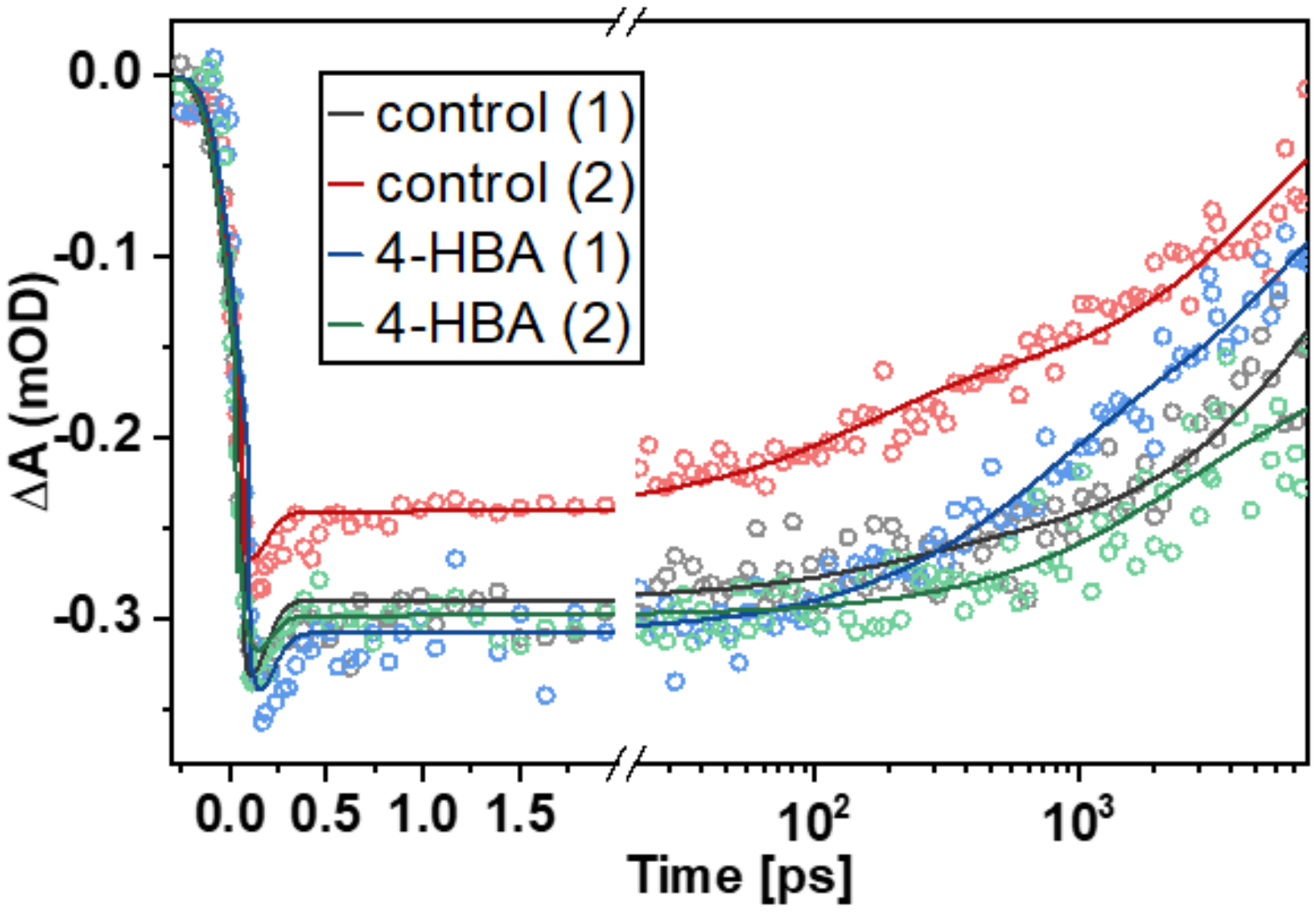

B)

Sample	t_1 (ps)	t_2 (ps)	t_3 (ns)
control	<0.15	205 ± 121	11.5 ± 1.2
control	<0.15	141 ± 33	5.4 ± 0.5
4-HBA	<0.15	612 ± 146	8.9 ± 1.4
4-HBA	<0.15	1862 ± 1426	46 ± 96

Figure S19 a) Transient absorption kinetic traces extracted from the ground state bleach minima at 678 nm for the different samples. b) Lifetimes extracted from a three-exponential decay function. We observe the trend of longer lifetimes for HBA samples.

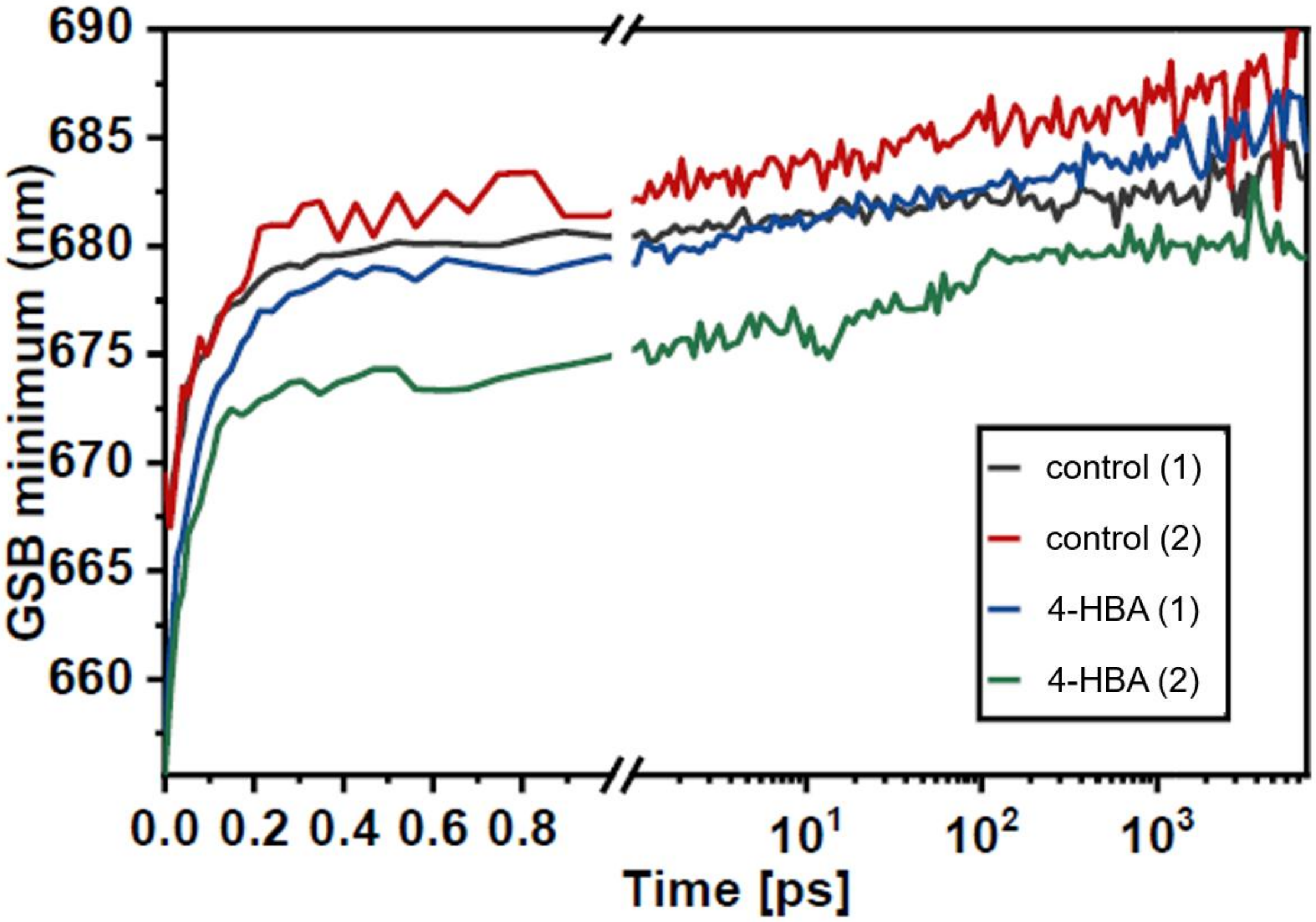

Figure S20. The wavelength position of the minimum of the ground state bleach as a function of delay time is shown, obtained from fitting a Gaussian function to the transient spectrum at every delay time after zero. In the first 400 fs, we can observe charge carrier thermalization, while at longer delay times, the shift may arise from phase segregation. The phase segregation is less pronounced in HBA samples.

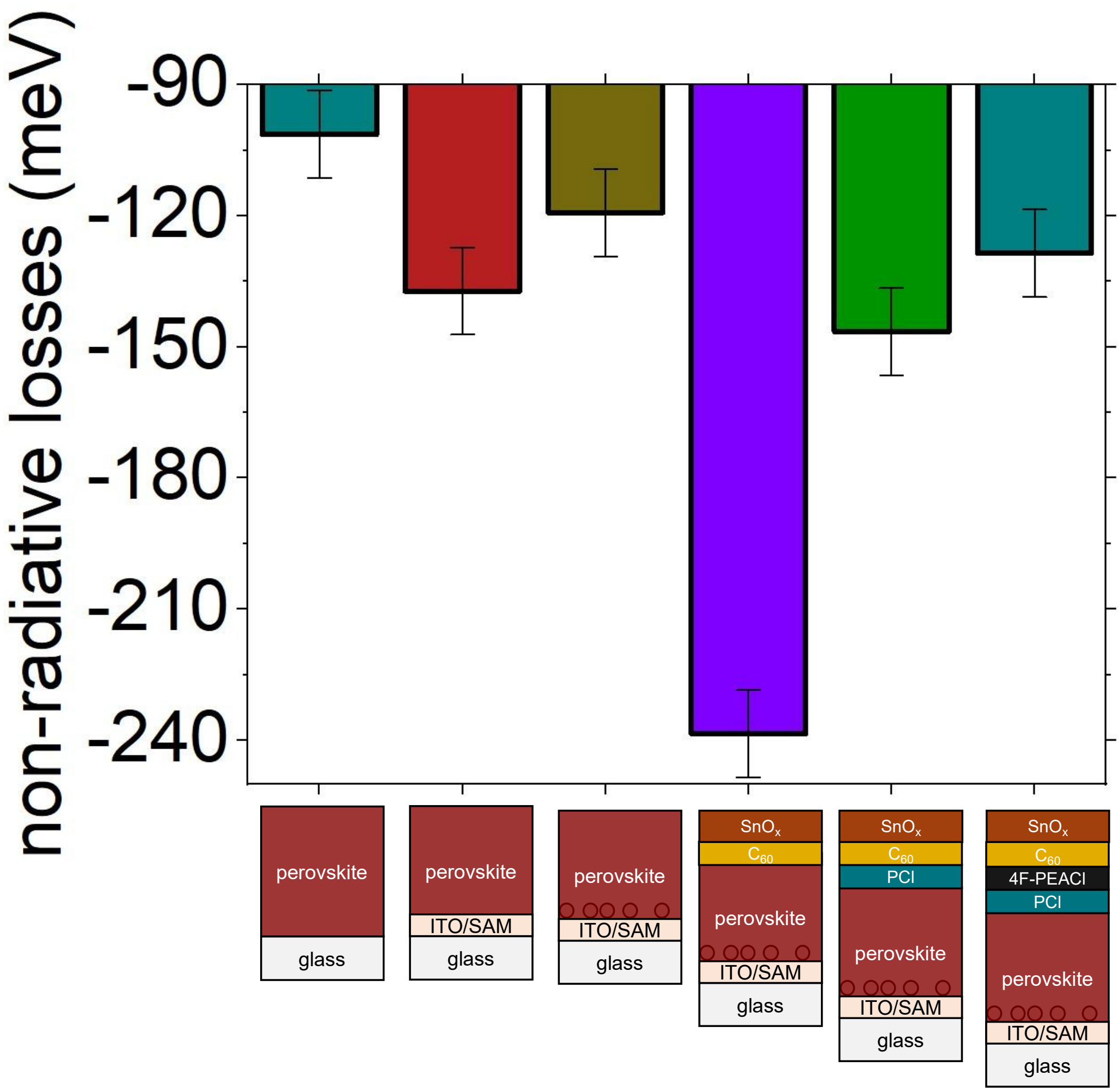

Figure S21. Non-radiative loss analysis of WBG top-cells.

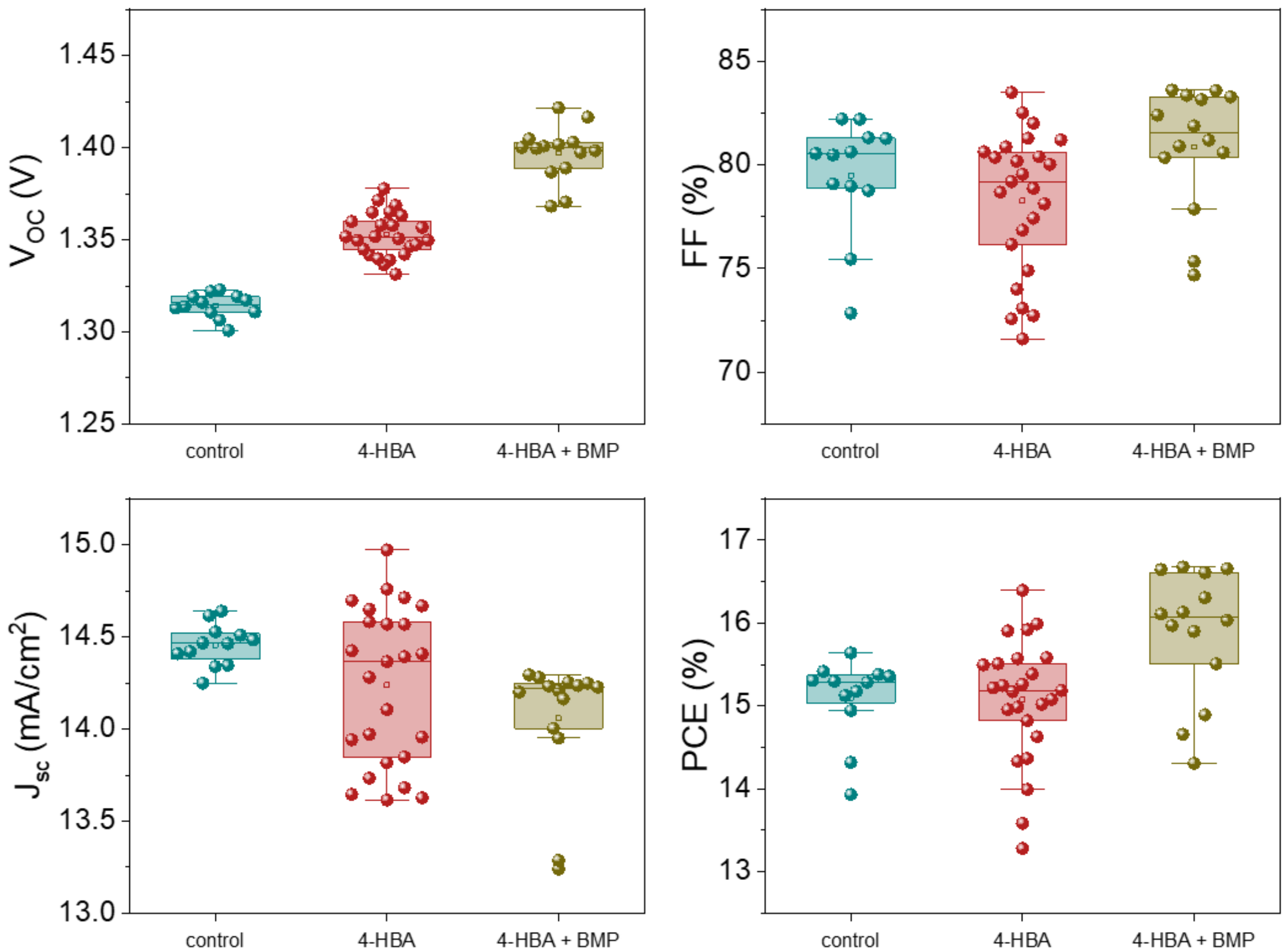

Figure S22. PV performance parameters of WBG single junction devices (control, 4-HBA and 4-HBA with bimolecular passivation - BMP).

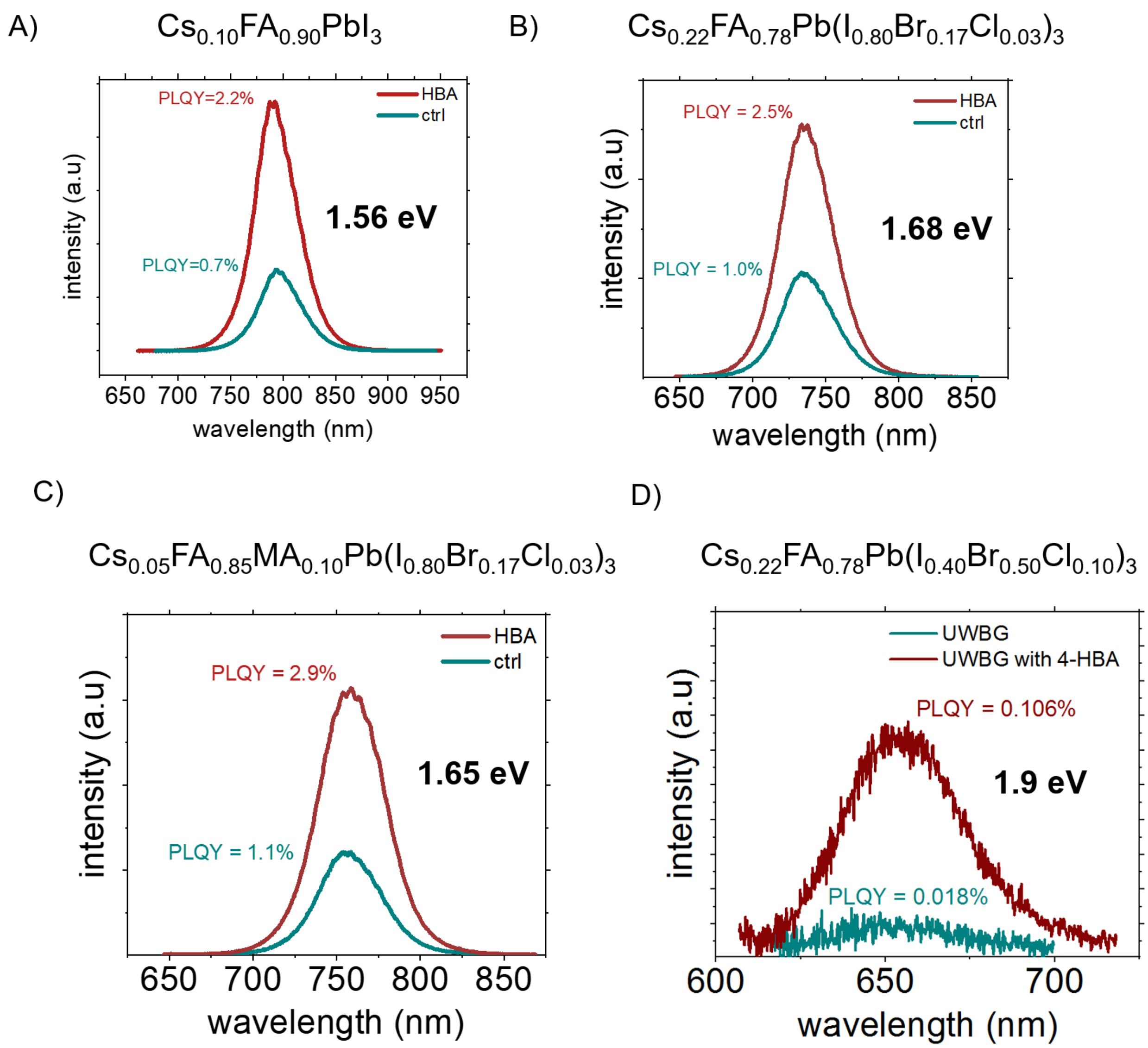

Figure S23. PLQY measurements of ITO-HTL-perovskite stacks with four different absorbers. Independent of composition, 4-HBA improves the PLQY.

4-HBA with $Cs_{0.10}FA_{0.90}PbI_3$

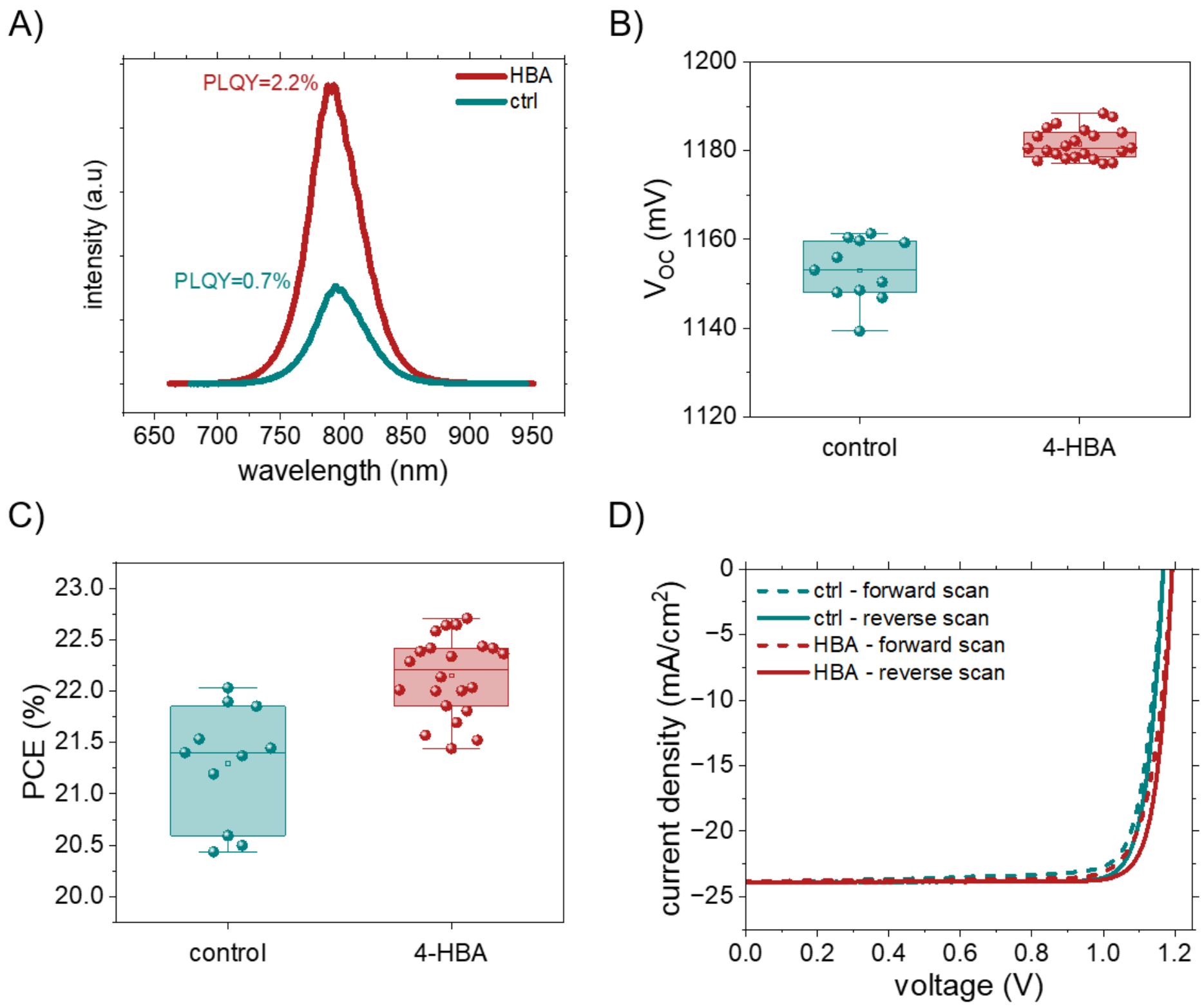

Figure S24. A) PLQY measurements of ITO-HTL-perovskite stack with and without 4-HBA with $CsFAPbI_3$ absorber. B) VOC, C) PCE and D) champion JV curves of single-junction perovskite solar cells with $CsFAPbI_3$ absorber with and without 4-HBA.

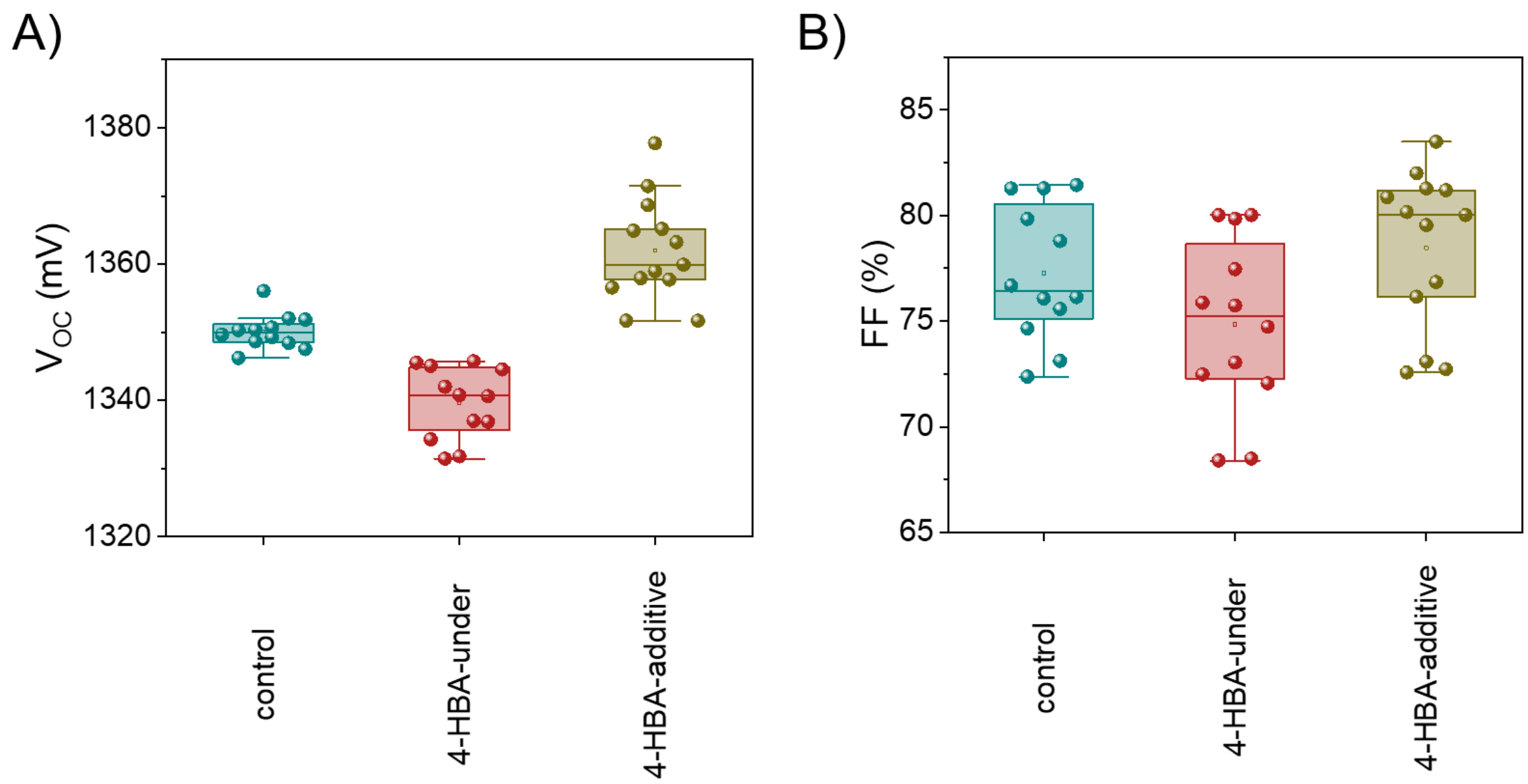

Figure S25. Device performance comparison, A) V_{OC}, B) FF of single-junction perovskite solar cells using WBG absorber, with 4-HBA additive, with 4-HBA surface treatment, and control.

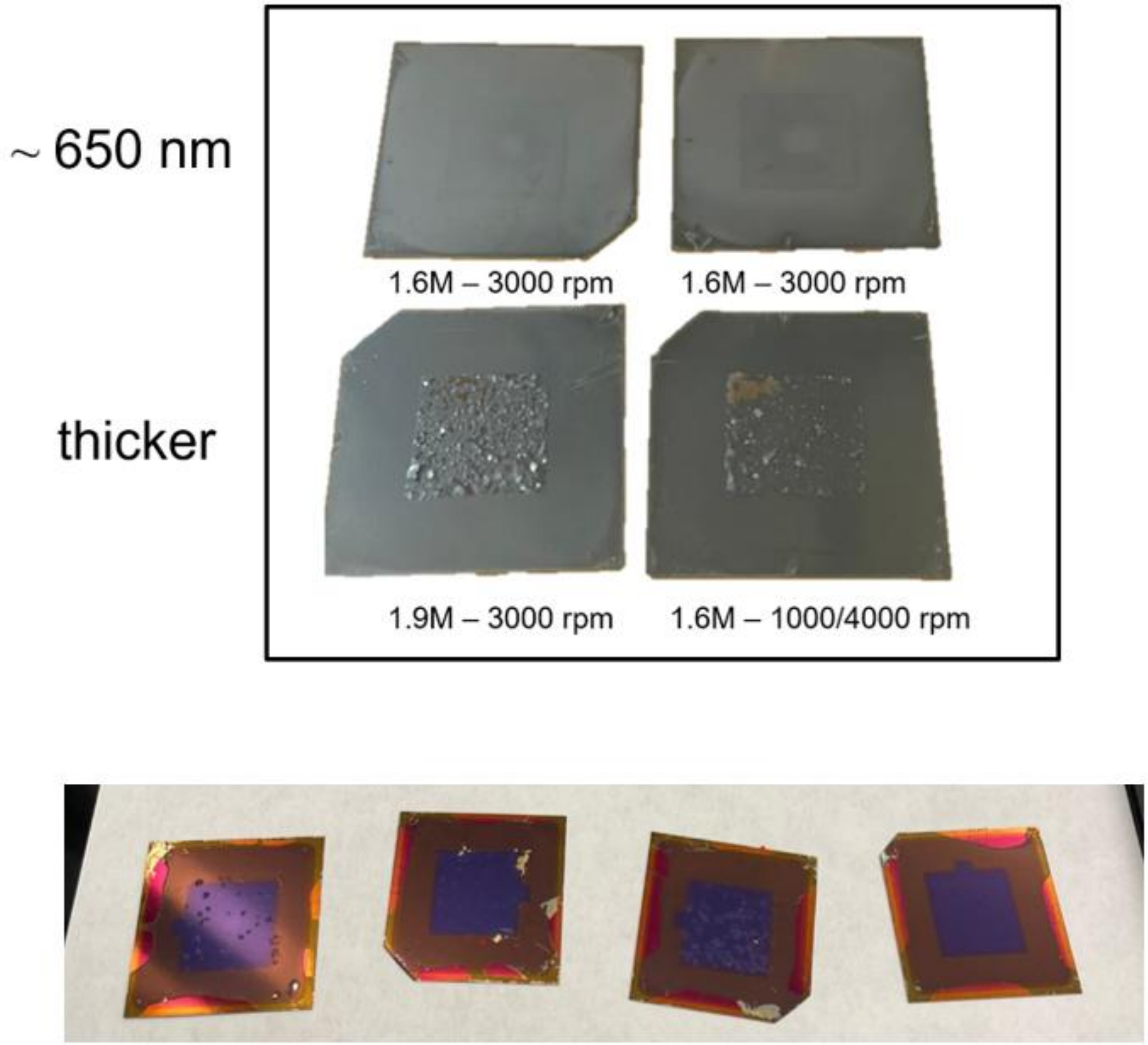

Figure S26. Top row: peeling off (delamination from the HTL side) during passivation processing, just before adding the C_{60} on the thick MBG absorber. Bottom row: Peeling off during top-cell SAM processing.

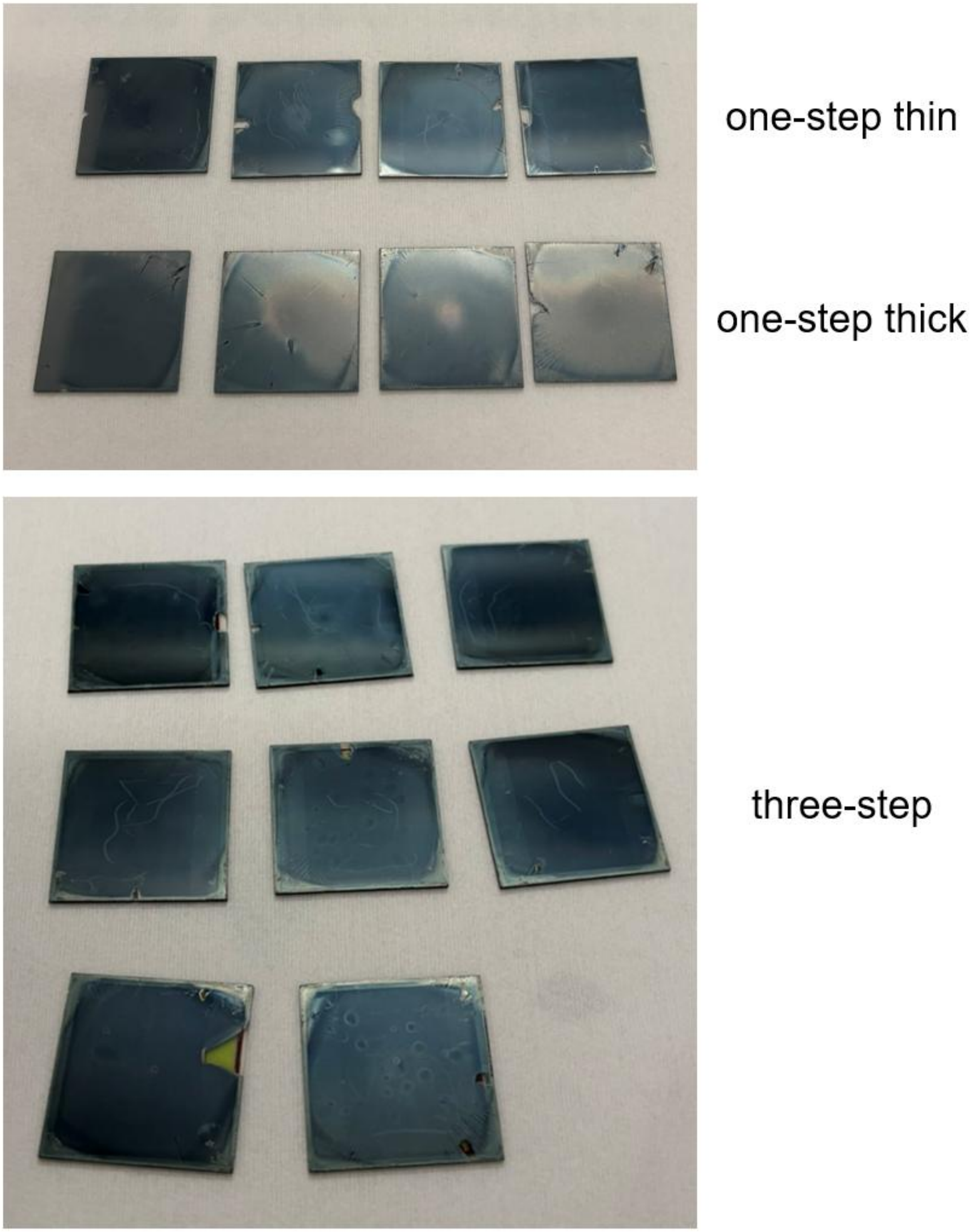

Figure S27. Optical images of one-step thin, one-step thick and three-step absorbers after the absorber fabrication on ITO-SAM. The rough morphology of the one-step thick absorbers is visible to the eye.

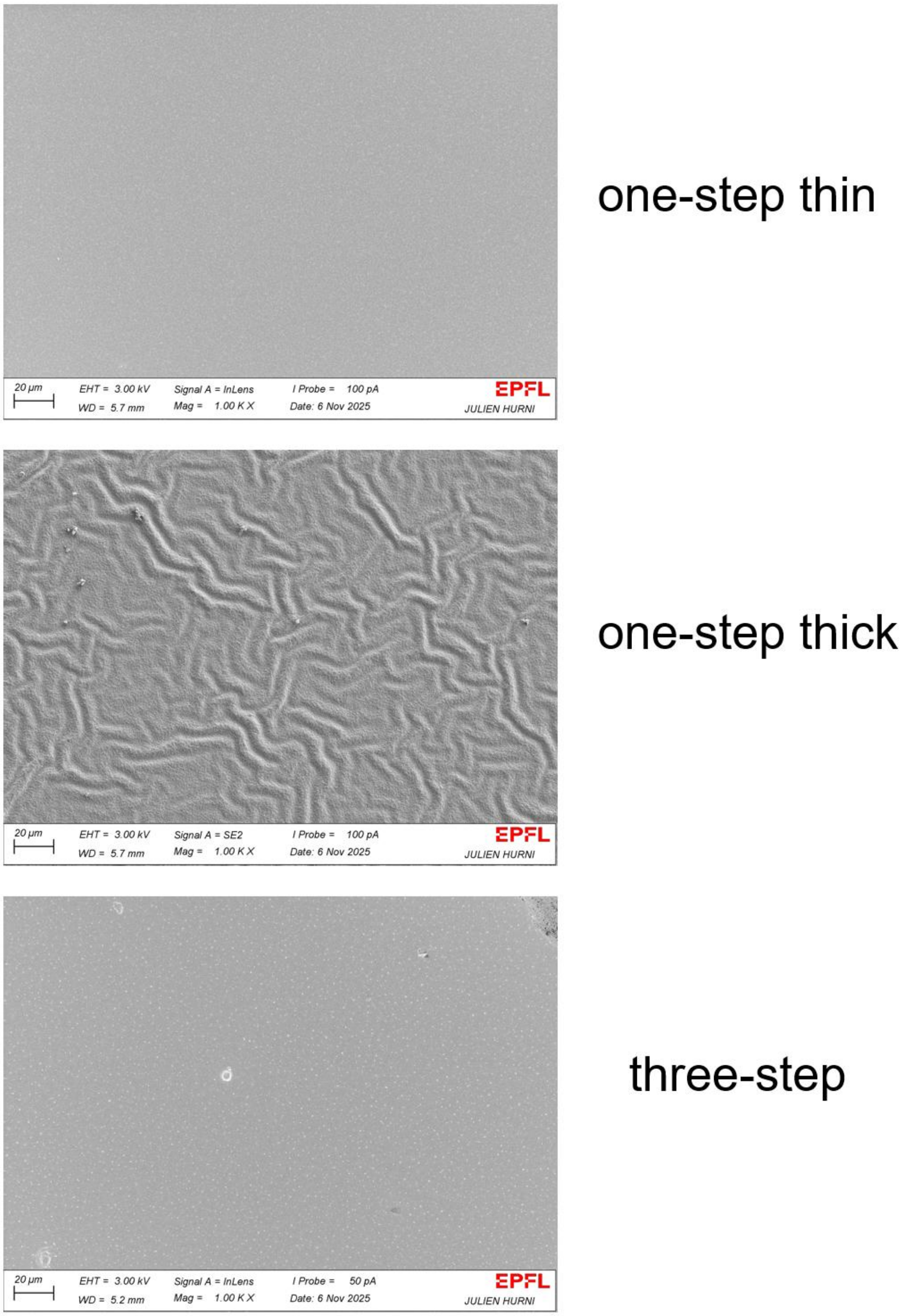

Figure S28. Top-view SEM of three different perovskite absorbers. The wrinkled morphology is observed specific to the one-step thick samples.

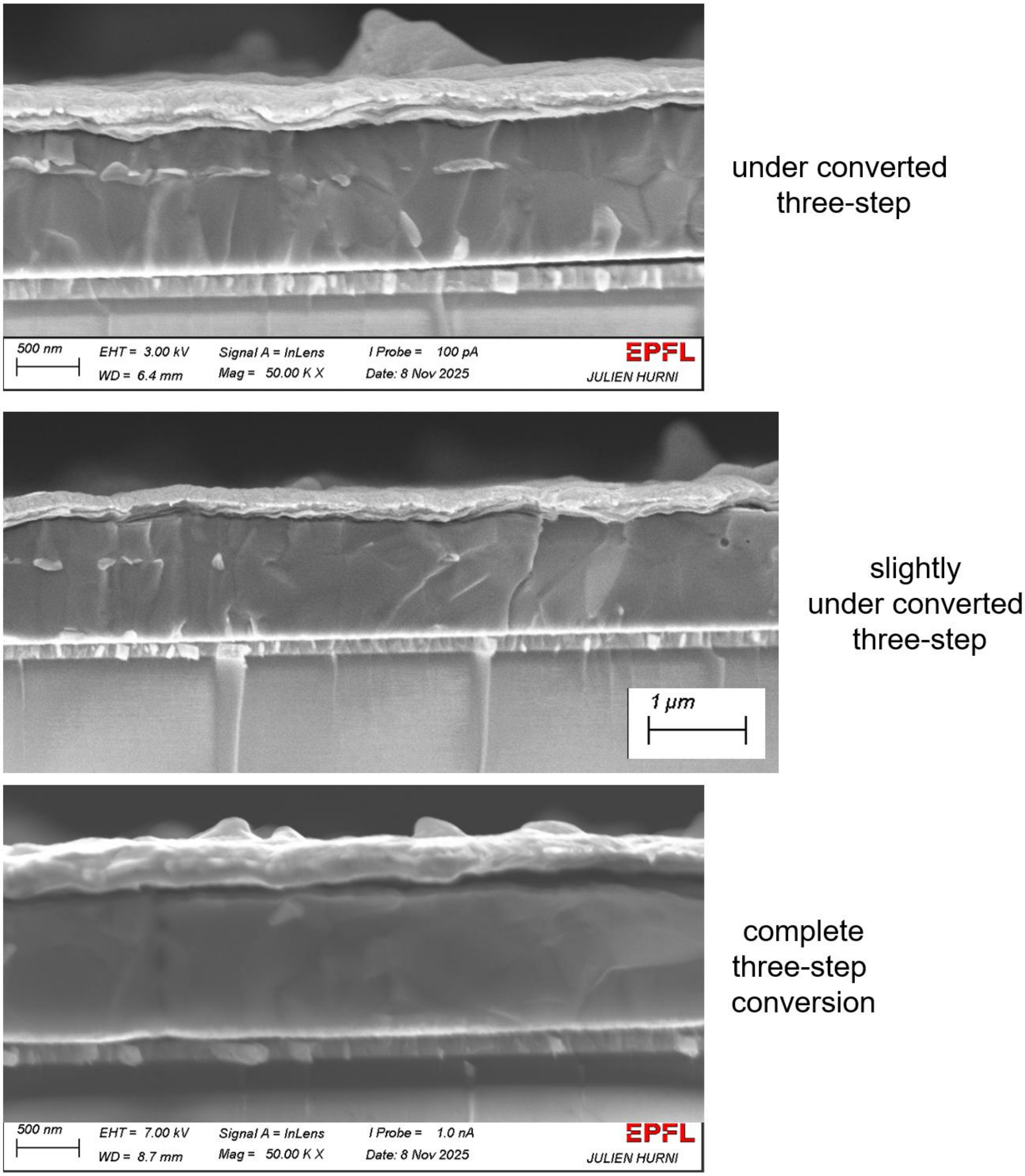

Figure S29. Optimization of the organohalide concentration of the three-step absorbers. Cross-section SEM of three-step perovskite absorbers with different degrees of conversion. PbI_2-residuals are not observed with the complete conversion.

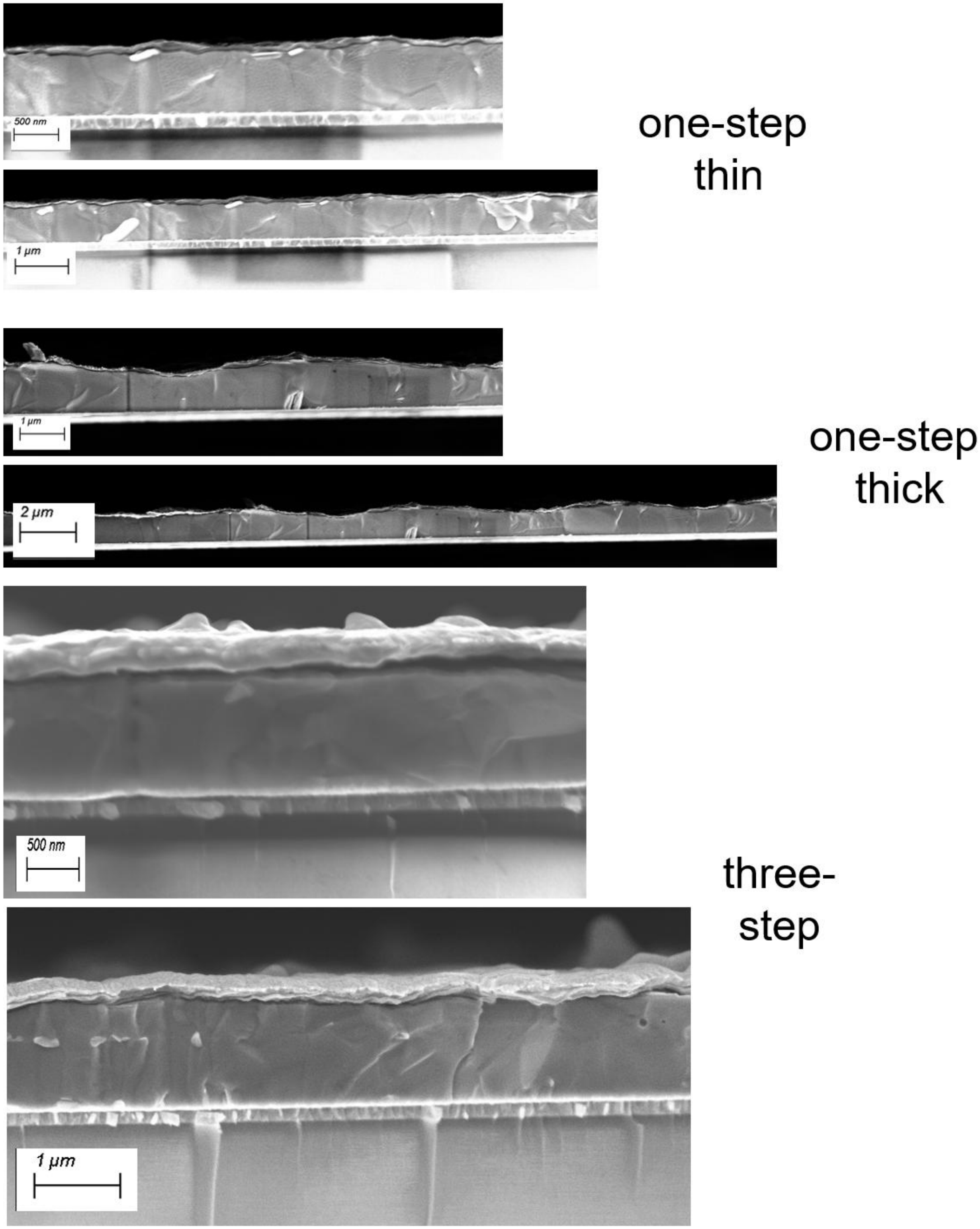

Figure S30. The surface waviness due to wrinkling is also visible in cross-section SEM. However, one-step thin and three-step absorbers preserve the smooth surface. One-step thick absorbers preserve the columnar thick domains similar to the one-step thin absorber.

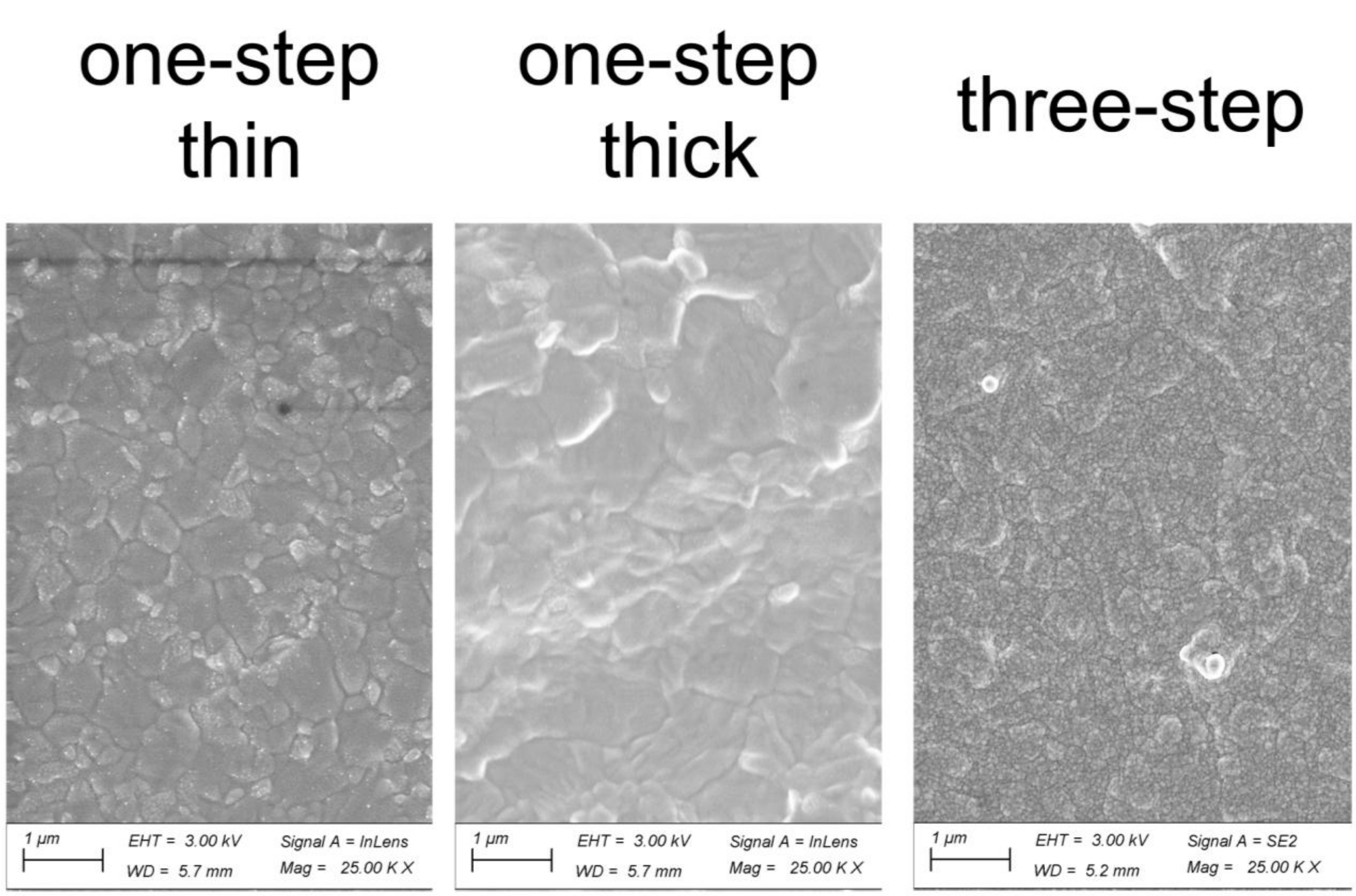

Figure S31. Top-view SEM imaging of ITO-HTL-perovskite-C60-SnO_x. The small islands represent ALD-SnOx layer, and the larger domains represent the perovskite microstructure. Each condition shows slightly different morphology.

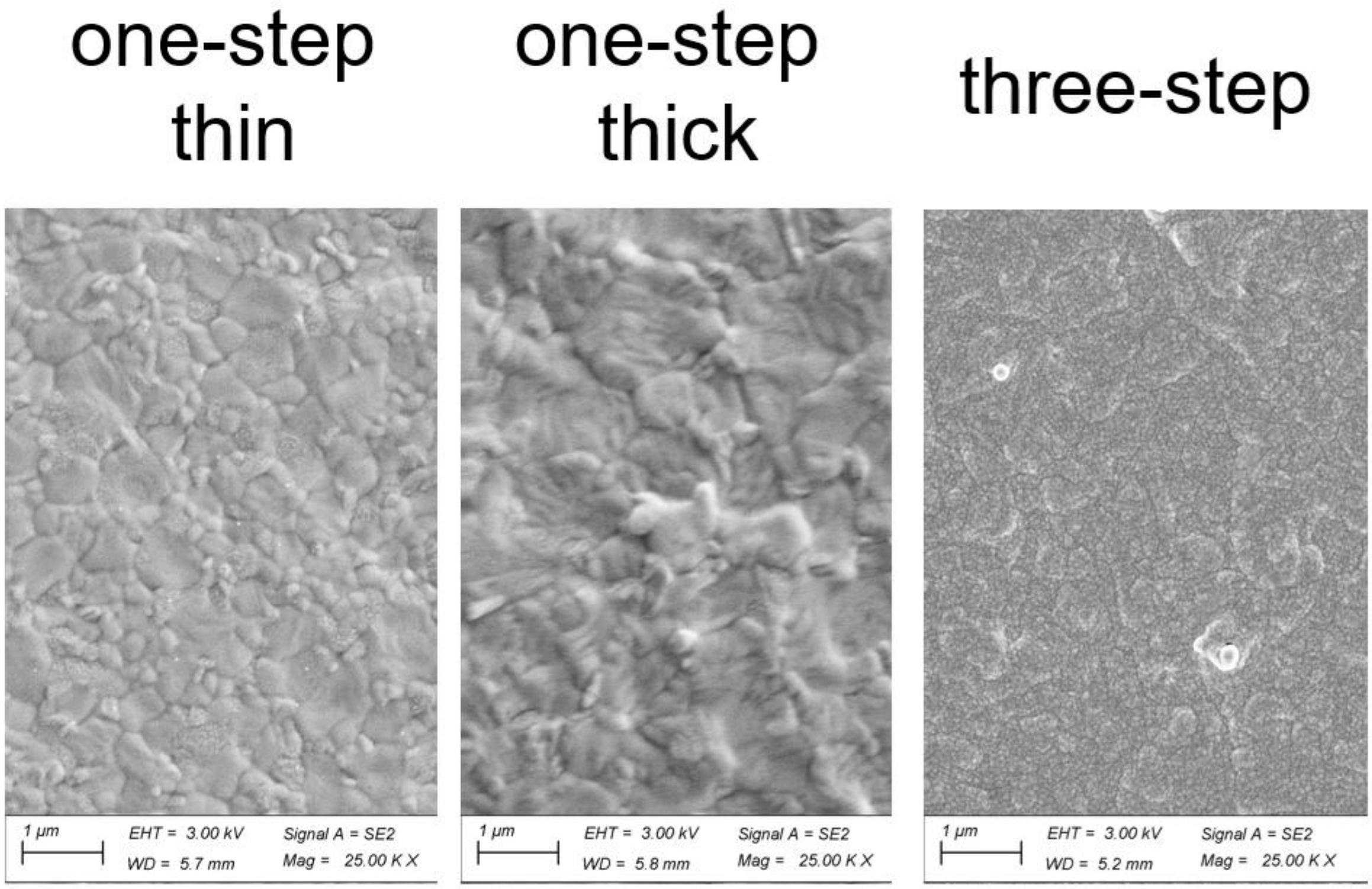

Figure S32. Top-view SEM images acquired using the secondary electron (SE2) detector to highlight surface roughness and morphological contrast. The one-step thick absorbers exhibit noticeably rougher surfaces.

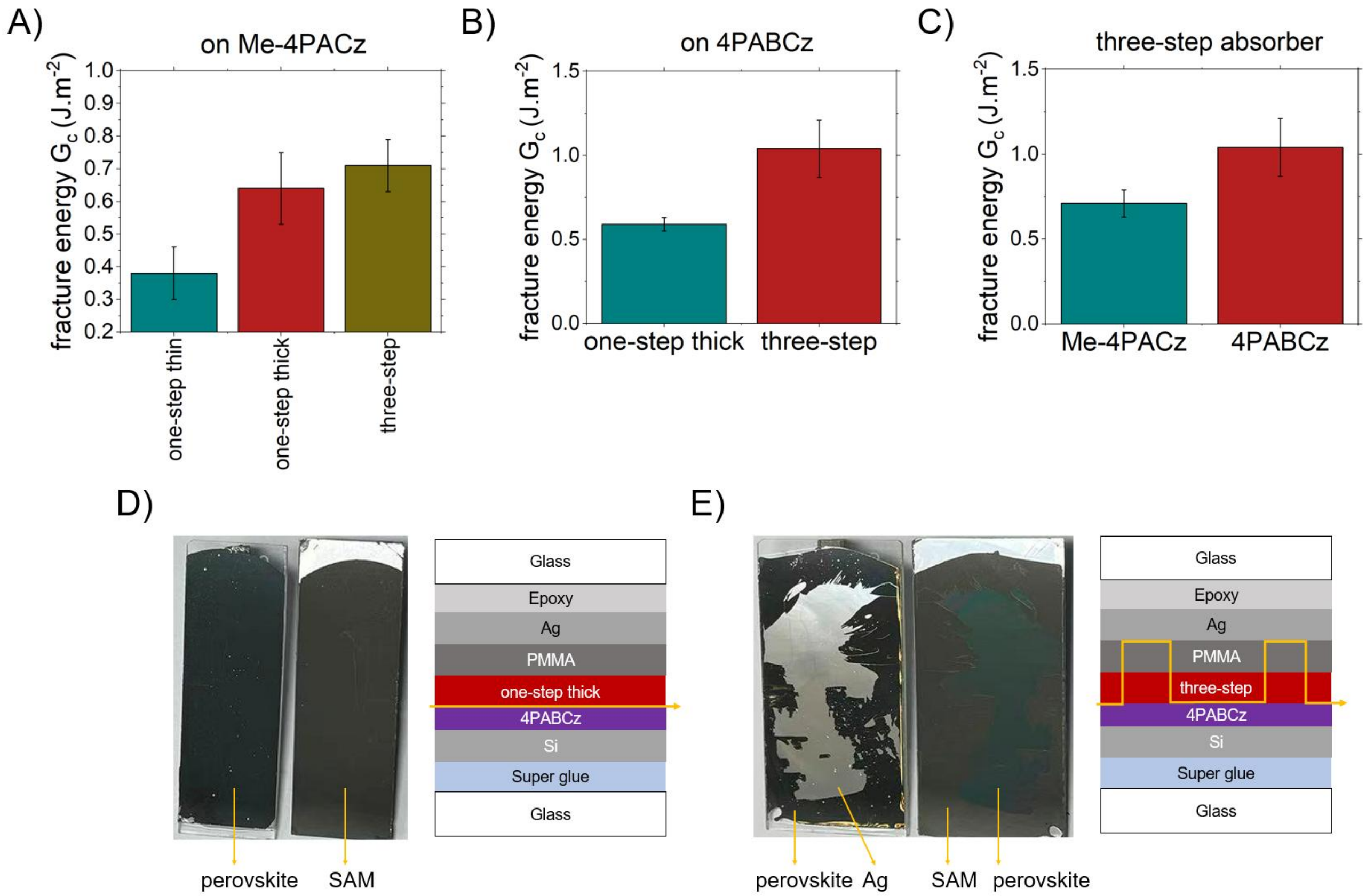

Figure S33. A) G_c of samples containing Me-4PACz with thin, thick, and three-step perovskite absorbers. (B) G_c of samples containing 4PABCz with one-step thick and three-step perovskite absorbers. (C) Comparison of G_c for three-step absorbers deposited on Me-4PACz and 4PABCz. (D, E) Images of fractured samples: (D) shows complete delamination at the 4PABCz/one-step thick perovskite interface in the one-step case, while (E) shows a meandering fracture path due to improved adhesion at the 4PABCz/three-step perovskite interface. Insets illustrate the stack structures of the measured samples with yellow arrows indicating the fracture path.

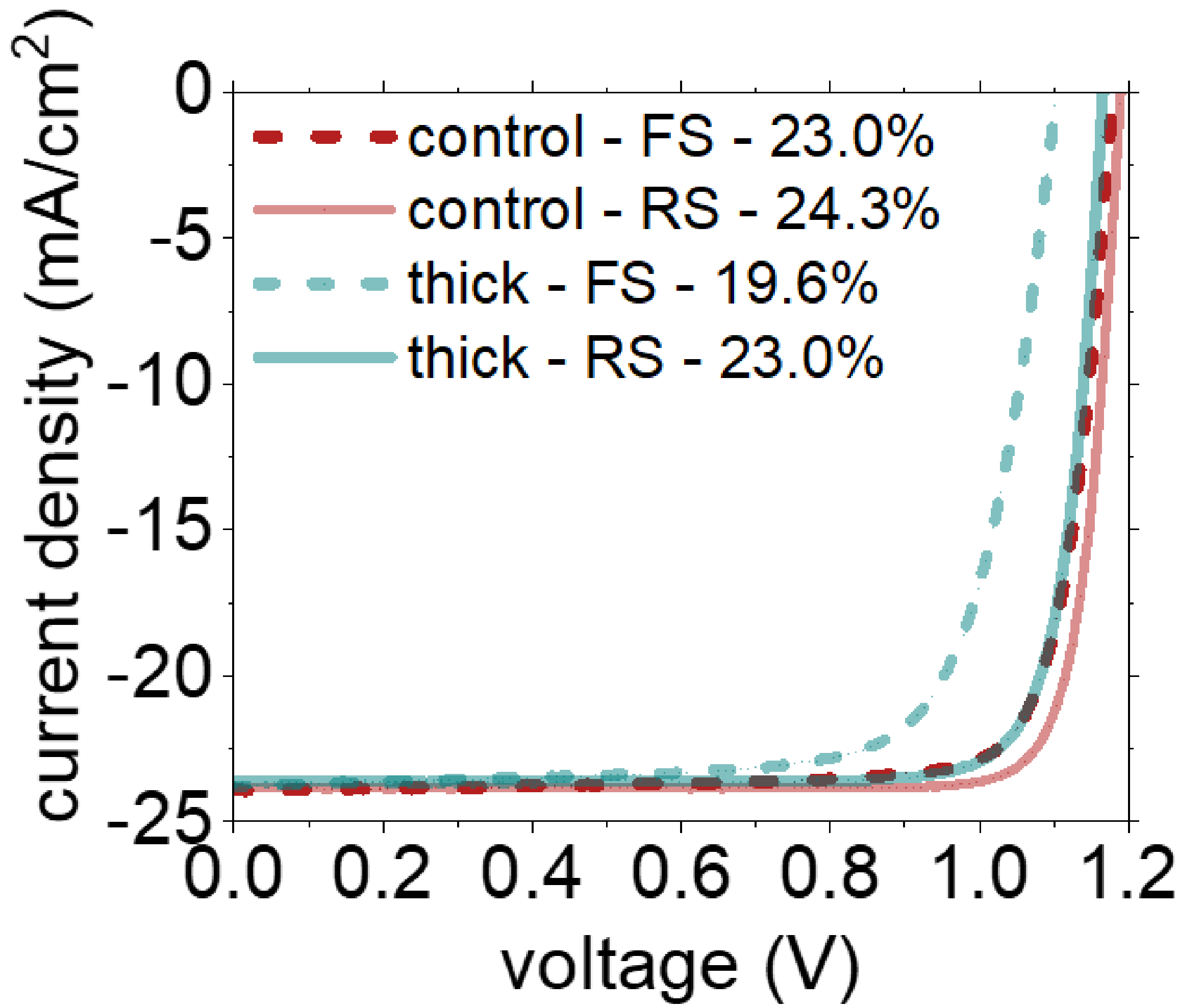

Figure S34. JV results of the champion single-junction perovskite solar cells with thin and thick one-step absorbers on 2PACz:4PADCB HTLs. Delamination is not observed on such an HTL-stack but severe hysteresis is present and J_{SC} decreases with increasing thickness likely due to enhanced ionic losses.

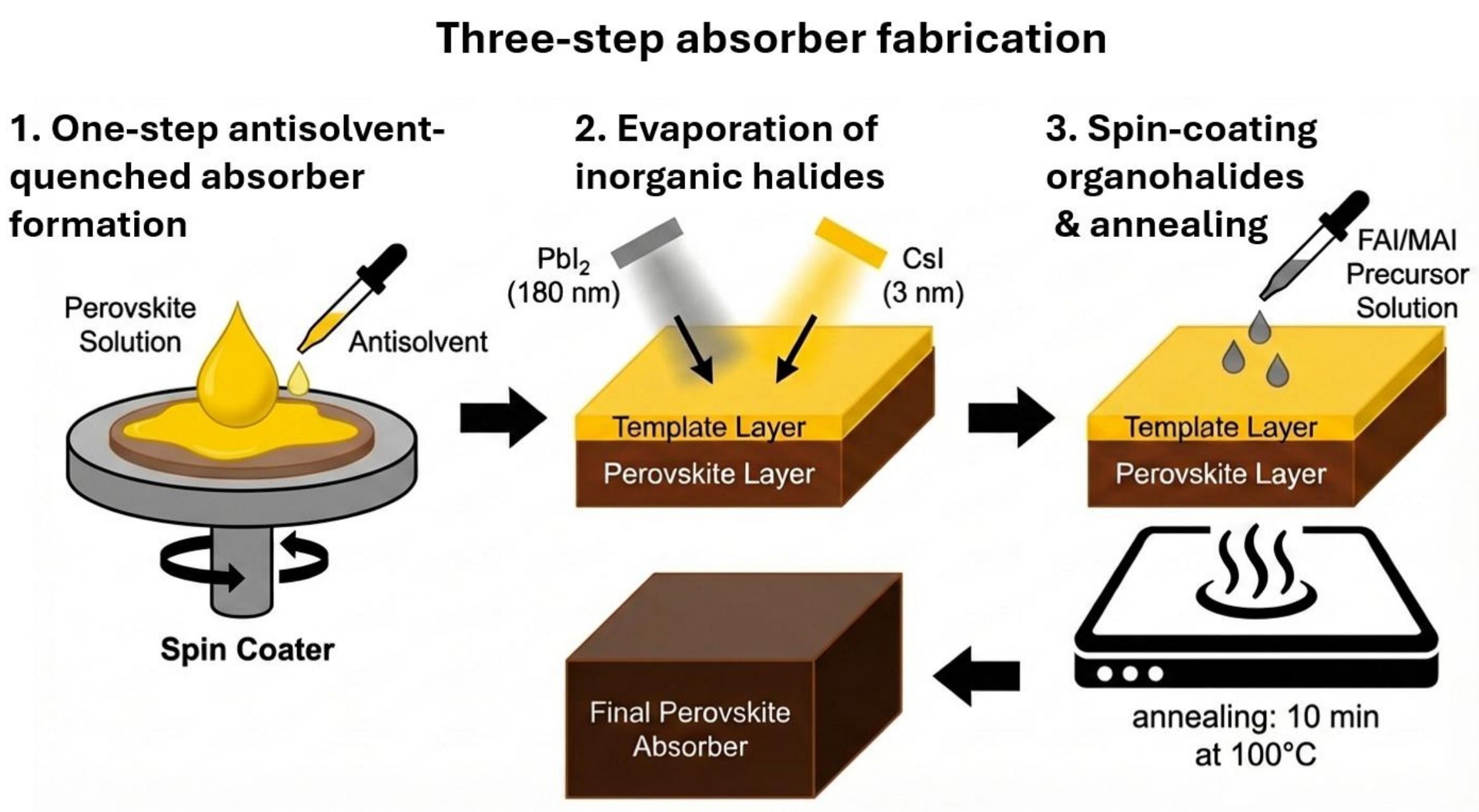

Figure S35. Fabrication procedure of three-step absorbers.

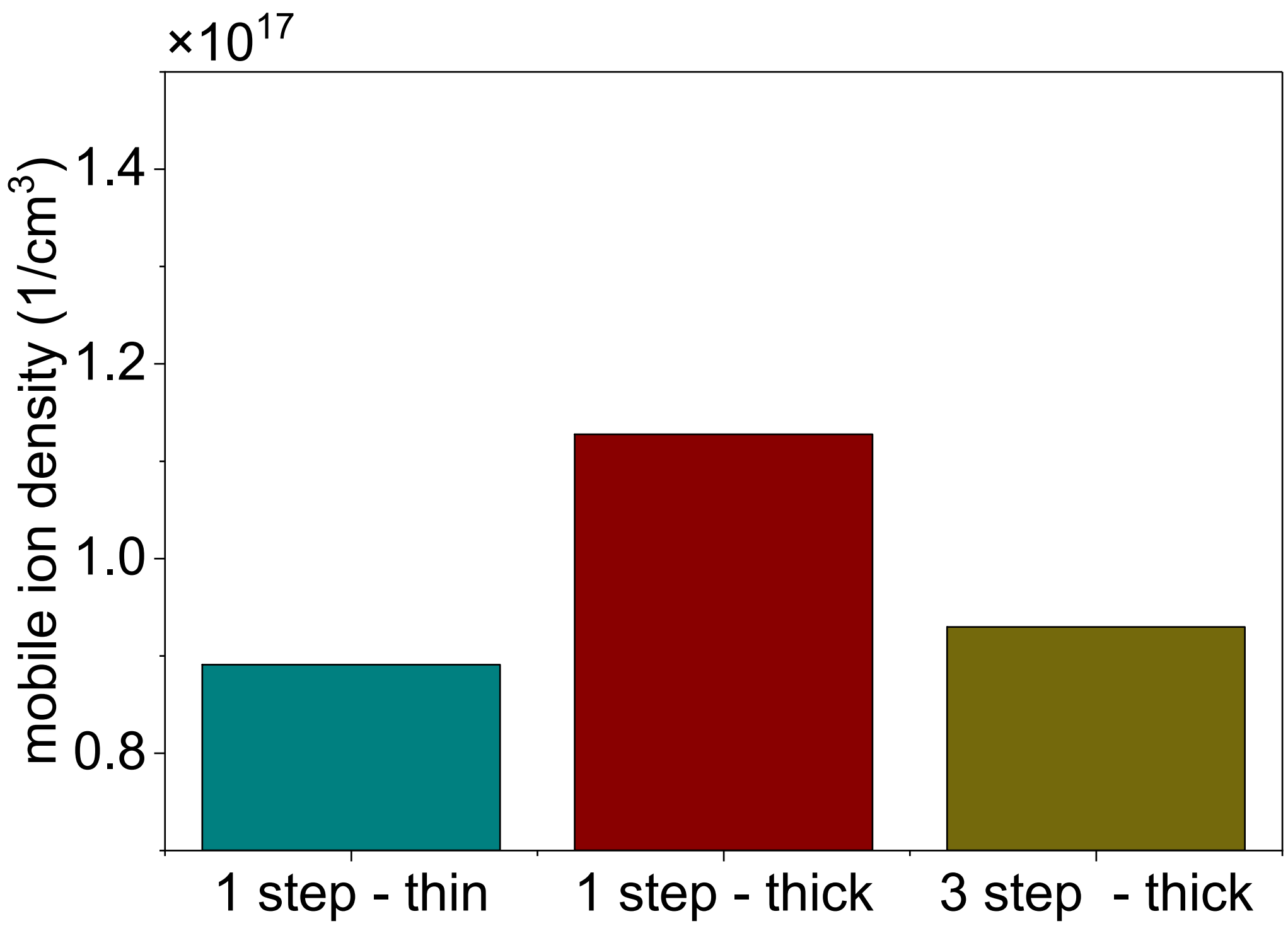

Figure S36. Mobile ion density extracted from BACE measurements. Although the mobile ion density increases for the one-step thick sample and remains relatively similar for the three-step sample, the overall increase is relevant but modest.

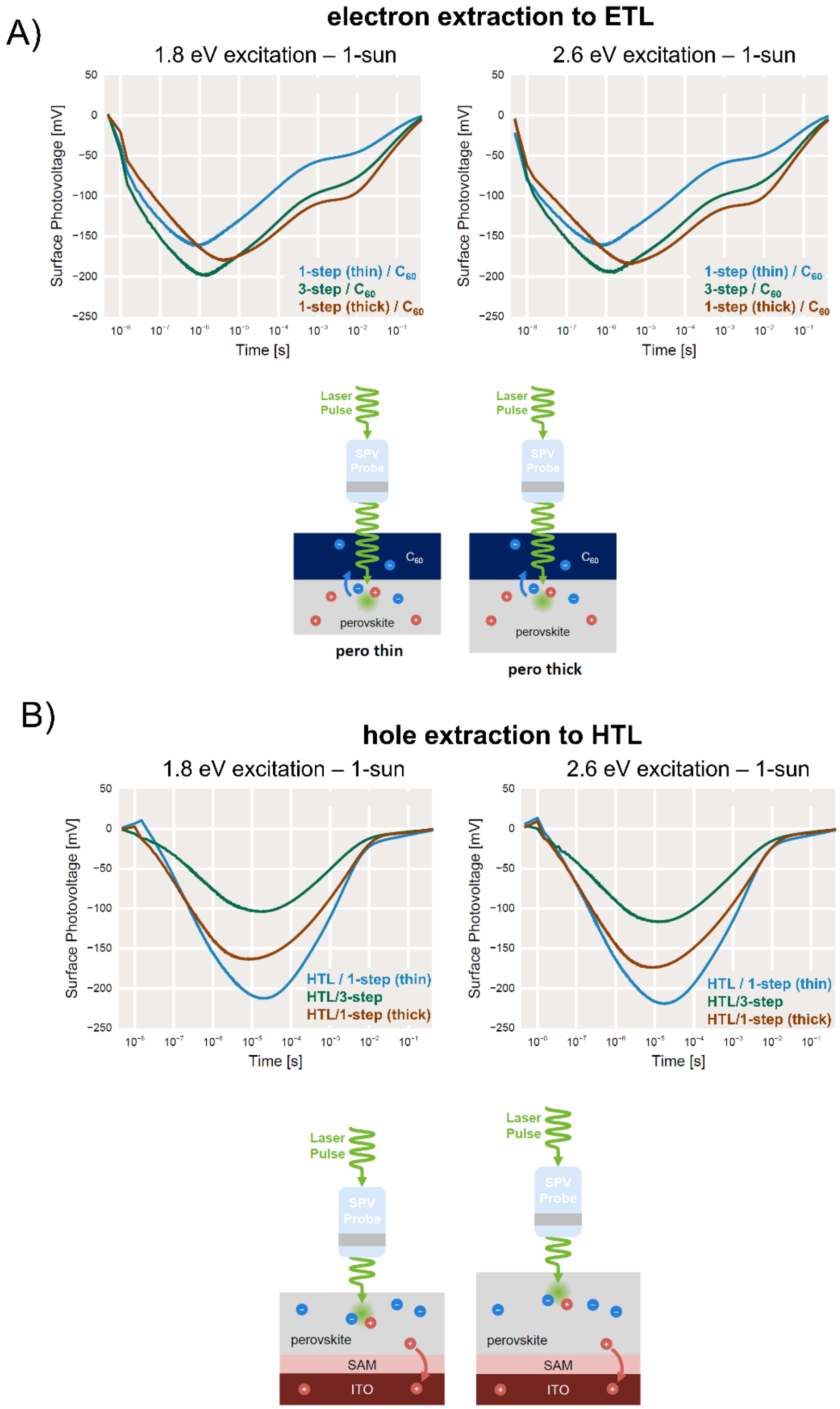

Figure S37. Transient SPV measurements. trSPV results show that the top interface to C_{60} improved for the 3-step deposition method (higher extraction velocity and amplitude), possibly due to higher absorption

(charge carrier density, which would be supported by higher J_{SC}) and lower non-radiative recombination, either due to better surface termination or crystallinity. SPV measurements show that 3-step deposition improves electron extraction but slightly reduces hole extraction. This reduction is most likely due to the increased film thickness, which forces holes to diffuse longer distances before reaching the hole-selective interface. Two opposing mechanisms emerge: (1) increased carrier density and enhanced hole extraction at the C_{60} interface due to the higher thickness, and (2) reduced hole extraction caused by the longer diffusion path. Given that carrier lifetimes are long relative to extraction times (for a similar bulk quality absorber (2% PLQY), we estimated diffusion lengths in the order of 3.7 µm in our previous work (*83*), the second effect is expected to be less detrimental. We also exclude alternative explanations such as the formation of specific hole traps or differing electron and hole diffusion lengths, because the PL intensity increases and the SPV transient shape remains essentially unchanged, with no accelerated decay (*102*).

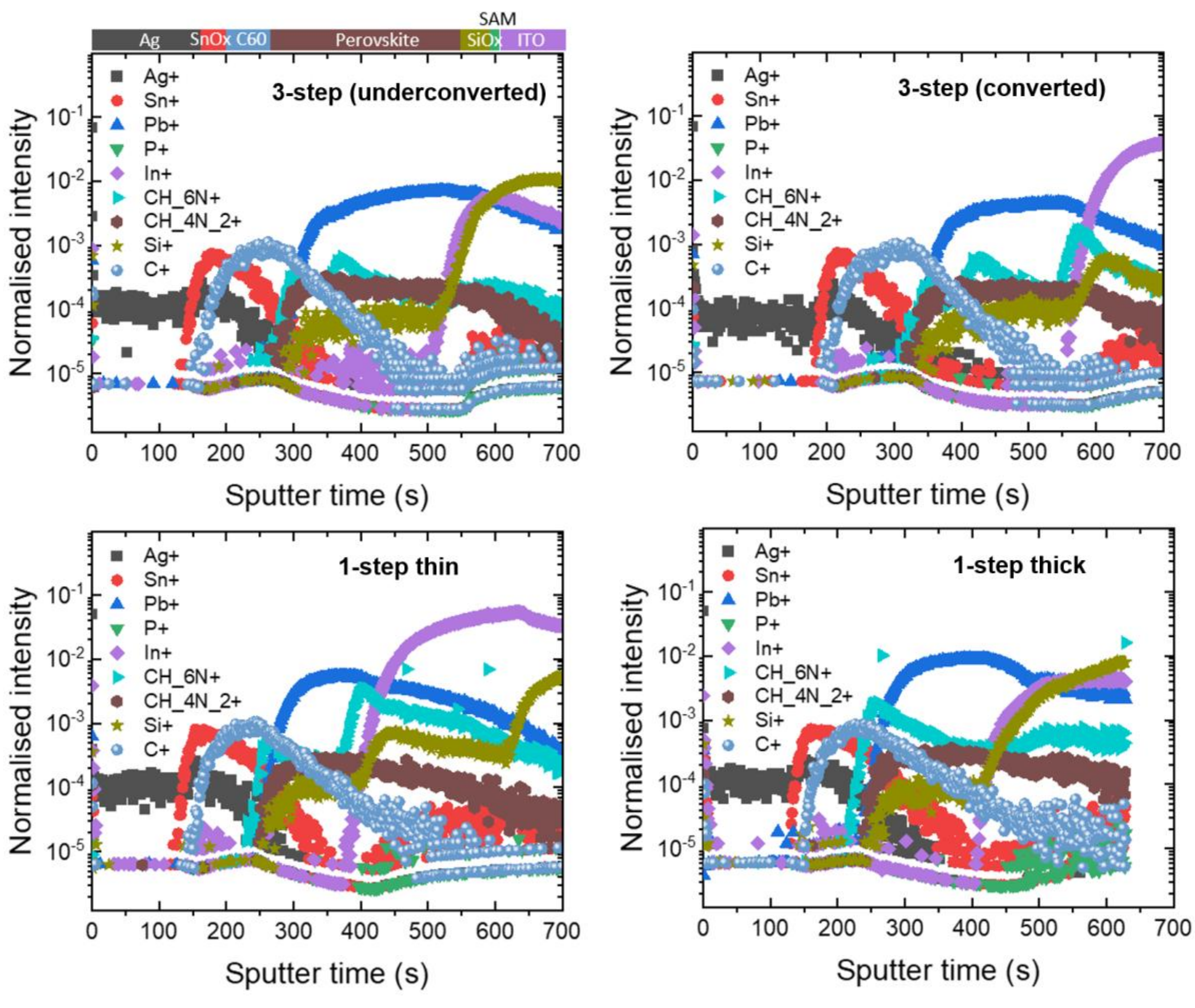

Figure S38. ToF-SIMS depth profiling of four different solar cells with four different absorbers. Due to the thicker Ag thin films on the 3-step converted device, the signals in that sample are shifted in the x-axis. Except for the underconverted 3-step sample that shows a gradient in the PbI- signal from top to bottom of the film, the other signals show relatively homogeneous distribution across the thickness.

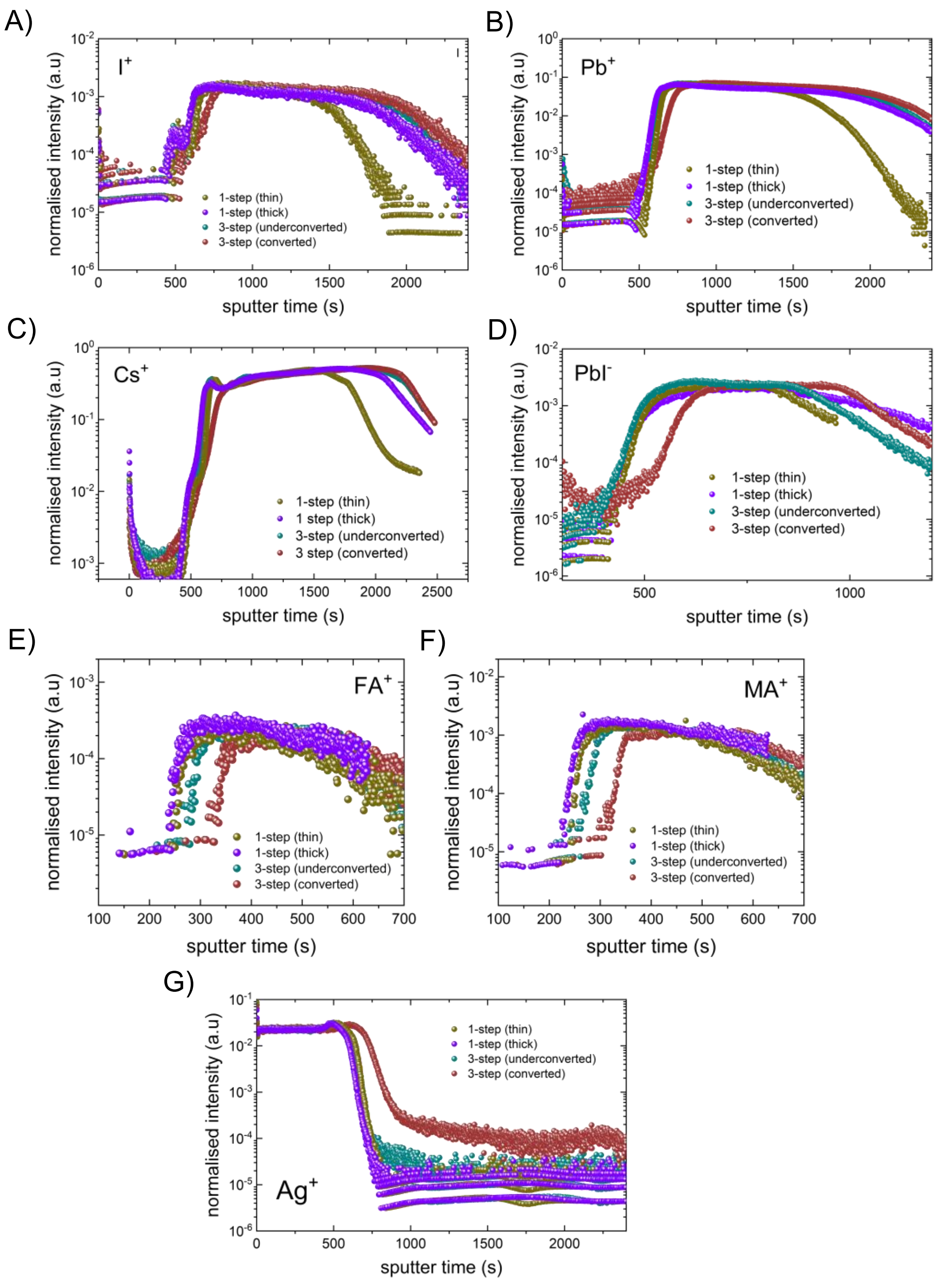

Figure S39. ToF-SIMS depth profiling of four different solar cells with four different absorbers. Due to the thicker Ag thin films on the 3-step converted device, the signals in that sample are shifted in the x-axis. Except the underconverted 3-step sample that shows a gradient in the PbI- signal from top and to bottom of the film, the other signals show relatively homogenous distribution across the thickness. We note that incomplete conversion of the hybrid absorber, most likely arising from an insufficient organohalide supply

and hereafter referred to as "underconverted", can lead to discontinuous PbI_2 residuals, consistent with previous reports on hybrid perovskite absorbers (*89*). Such residuals could, in principle, induce compositional inhomogeneity across the thickness of three-step–processed films (Fig.S40).

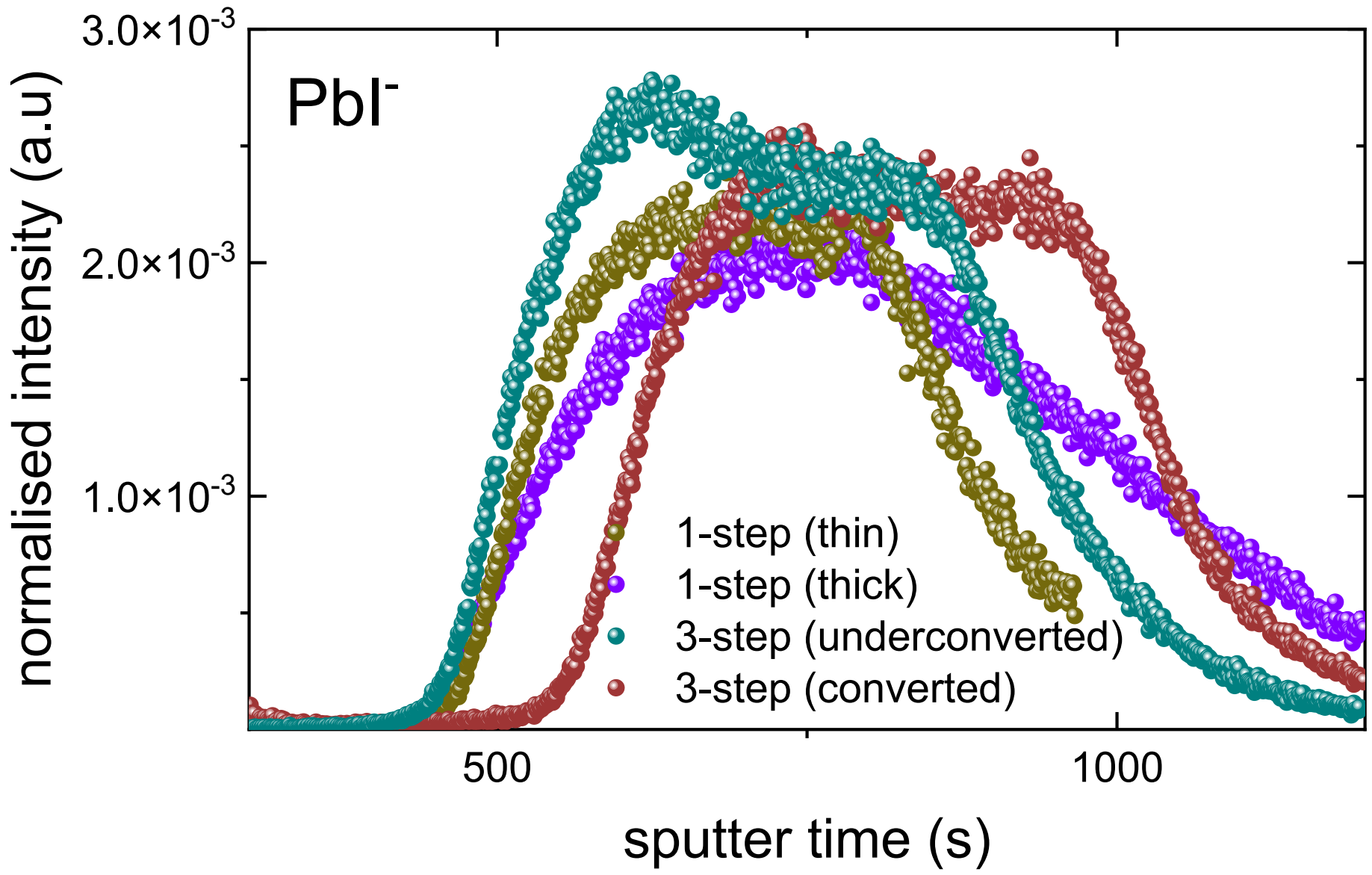

Figure S40. Linear scale PbI- peak to illustrate the differences. Investigation of other signals in linear scale did not alter any differentiable trend between samples. In contrast, the PbI^- signal exhibits a pronounced increase at the top of the under-converted three-step film, indicating localized excess PbI^- in this region, whereas the converted three-step films show a homogeneous PbI^- distribution consistent with the SEM observations (see Fig.S29-30).

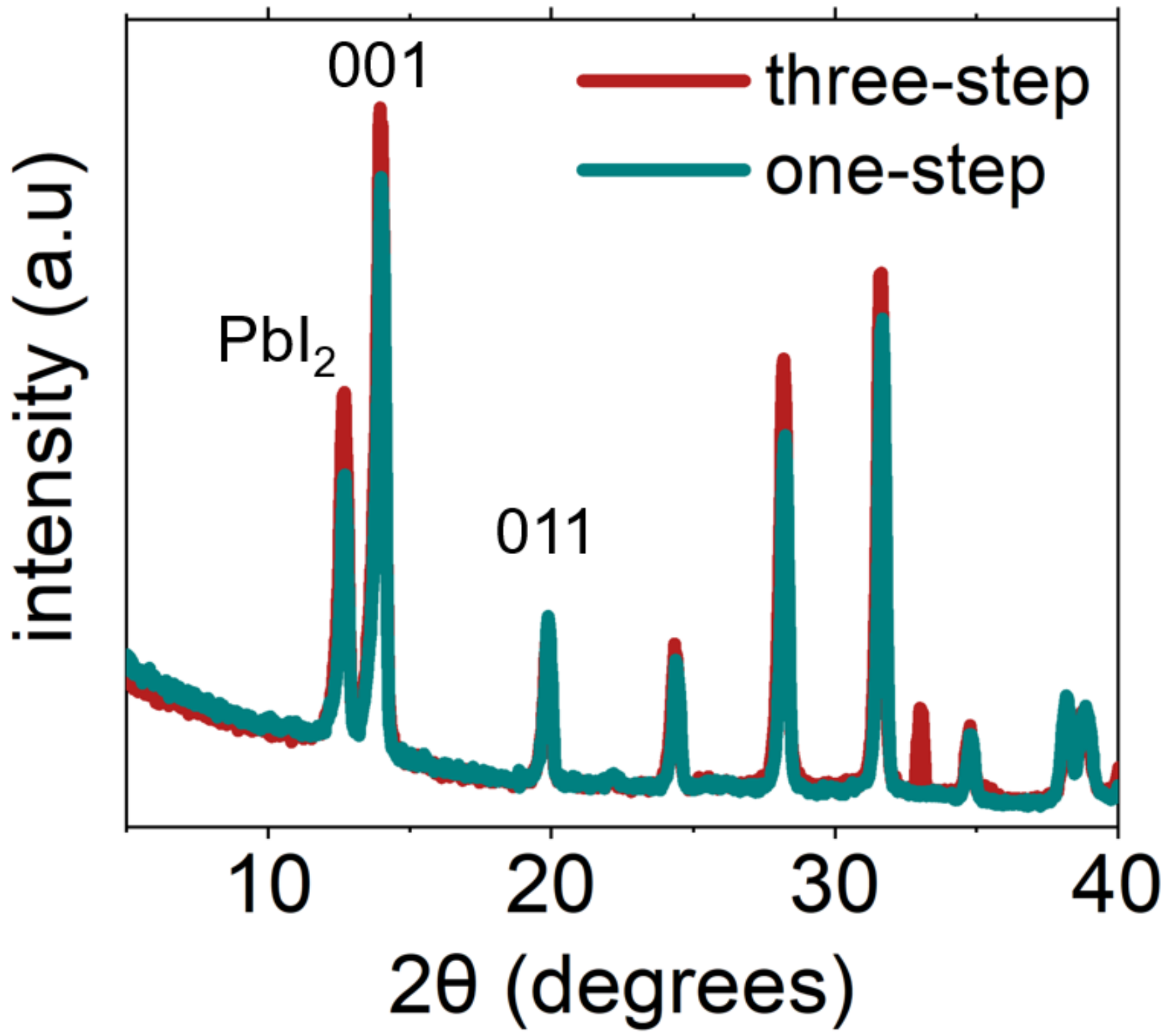

Figure S41. XRD of one-step and three-step absorbers.

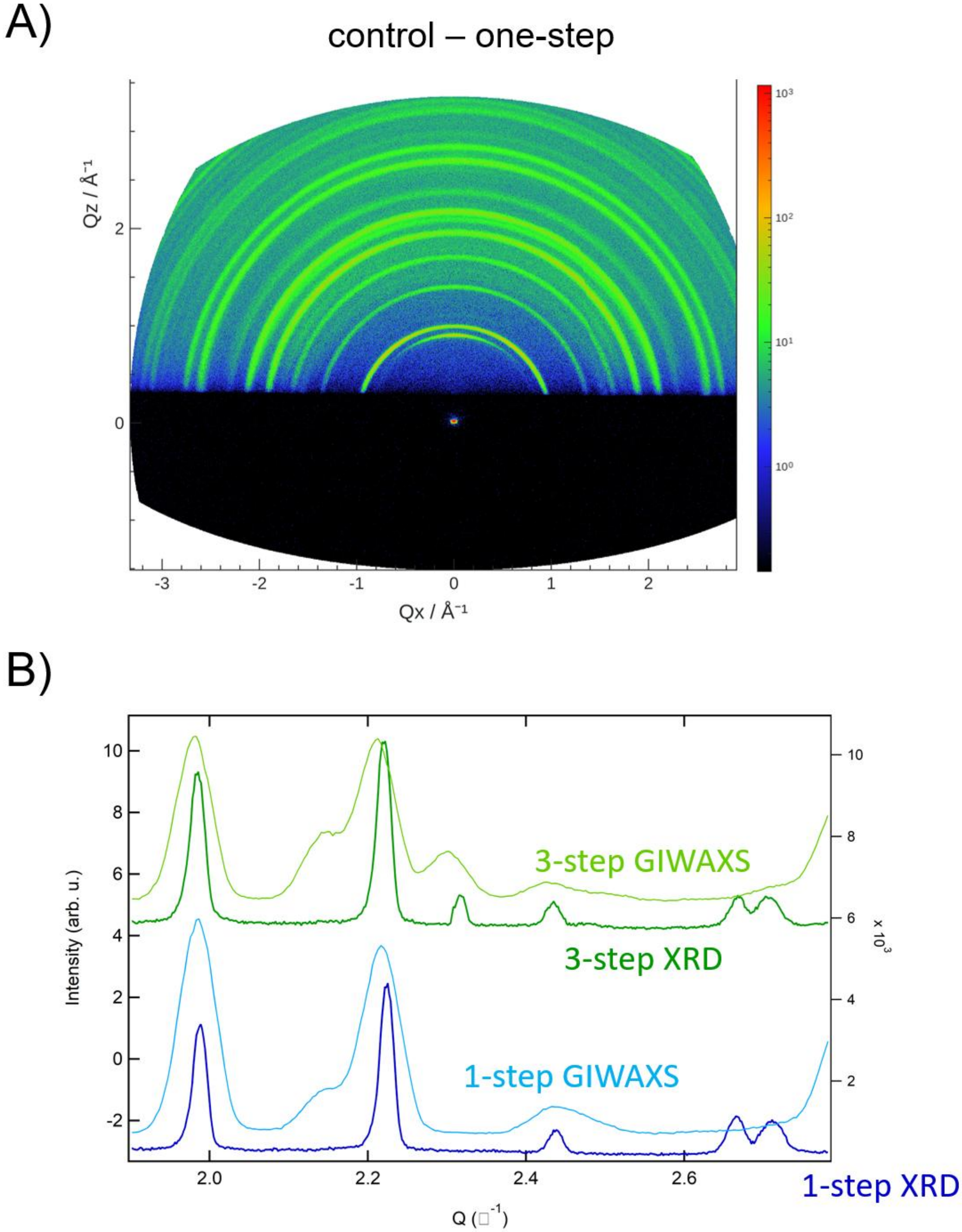

Figure S42. A) 2D GIWAXS measured at 4 degrees for the control one-step sample showing no preferential orientation. B) Comparison of 1D integration of GIWAXS and XRD measurements for control and target samples. GIWAXS measurements confirm the diffraction peak at $2\theta \approx 33°$ corresponds to $q \approx 2.3 A^{\circ -1}$ specific to three-step absorbers.

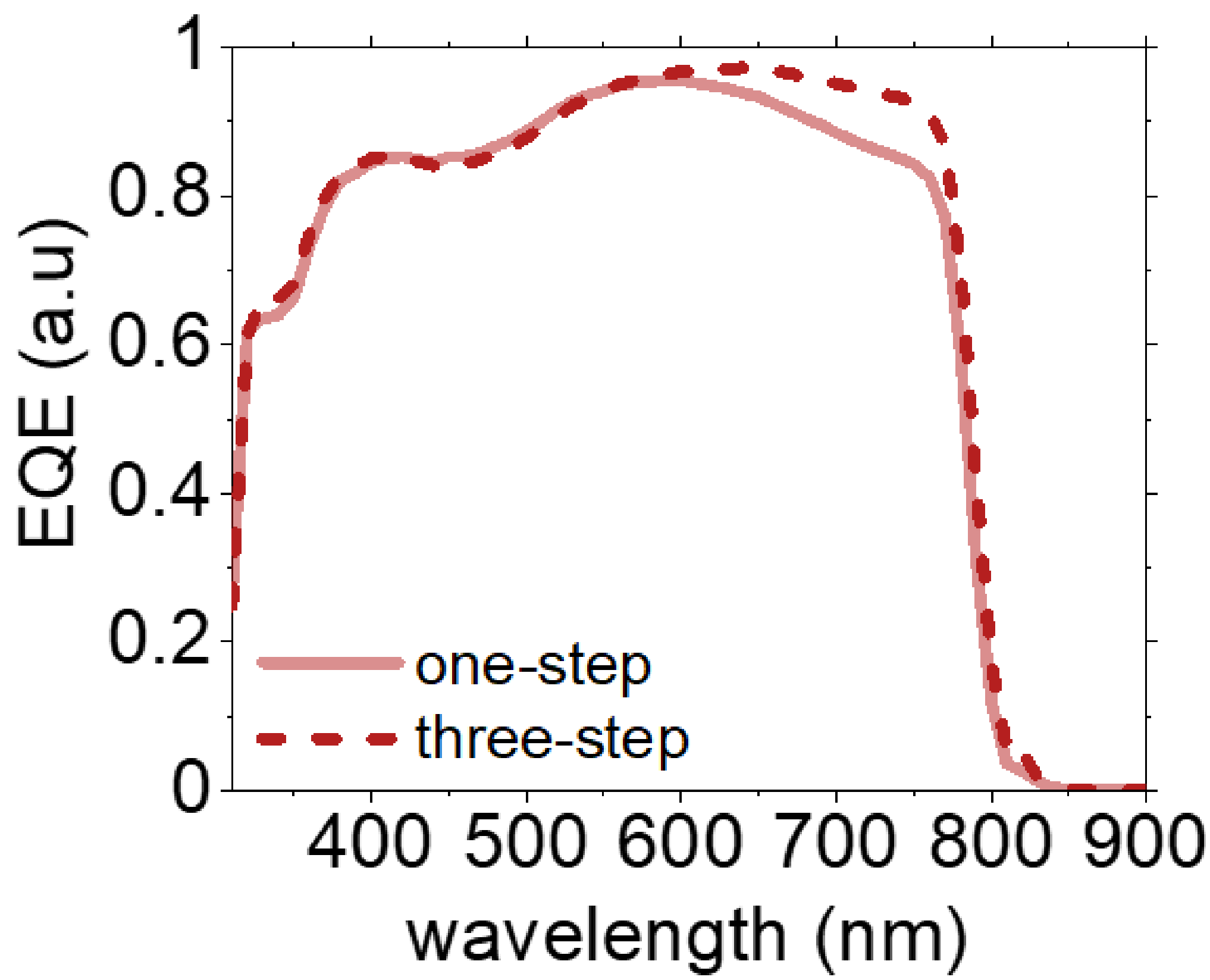

Figure S43. EQE comparison of one and three-step absorbers on ohmic-Si substrates.

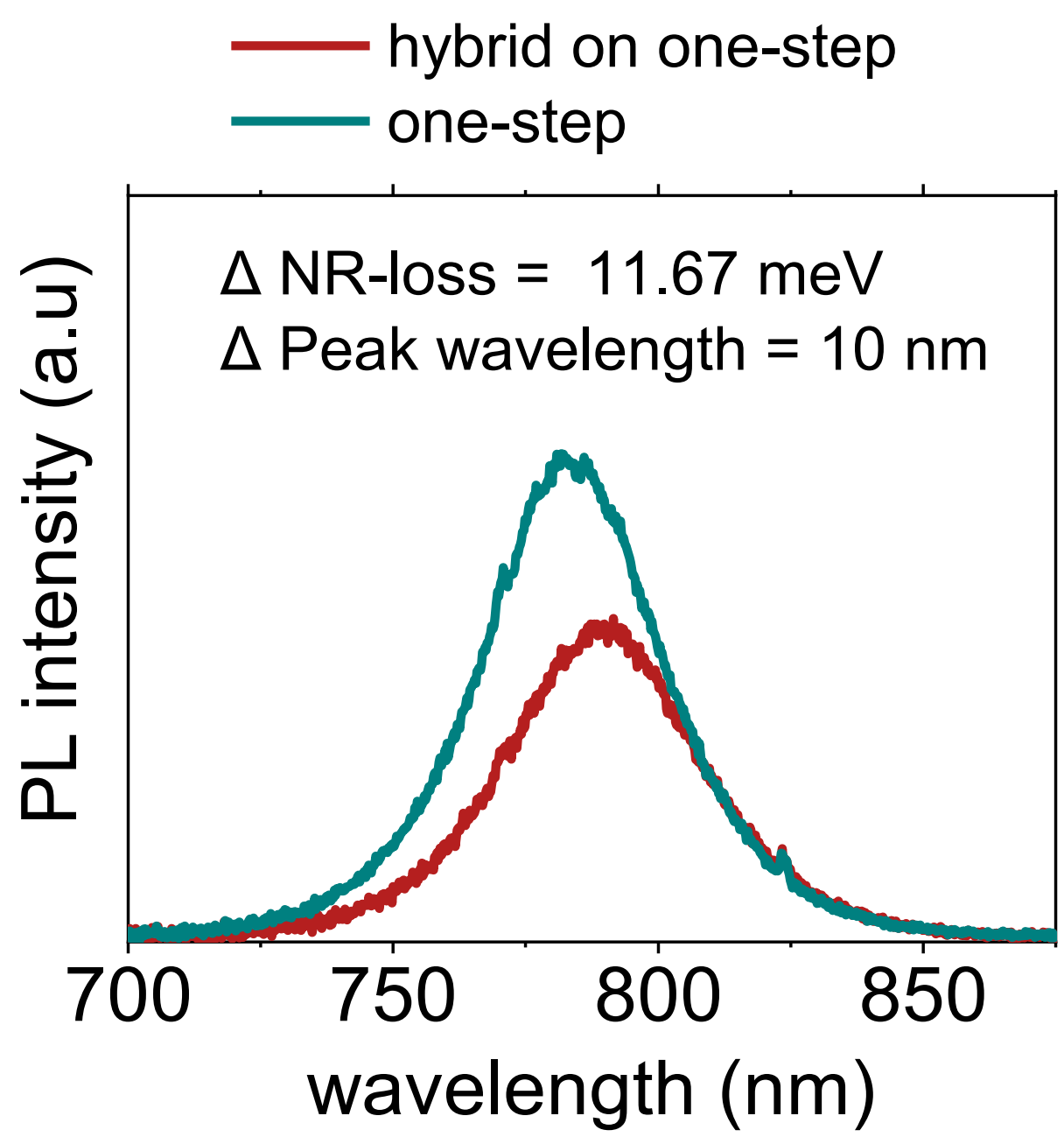

Figure S44. PLQY spectrum of the one-step and three-step (hybrid on one-step) absorbers.

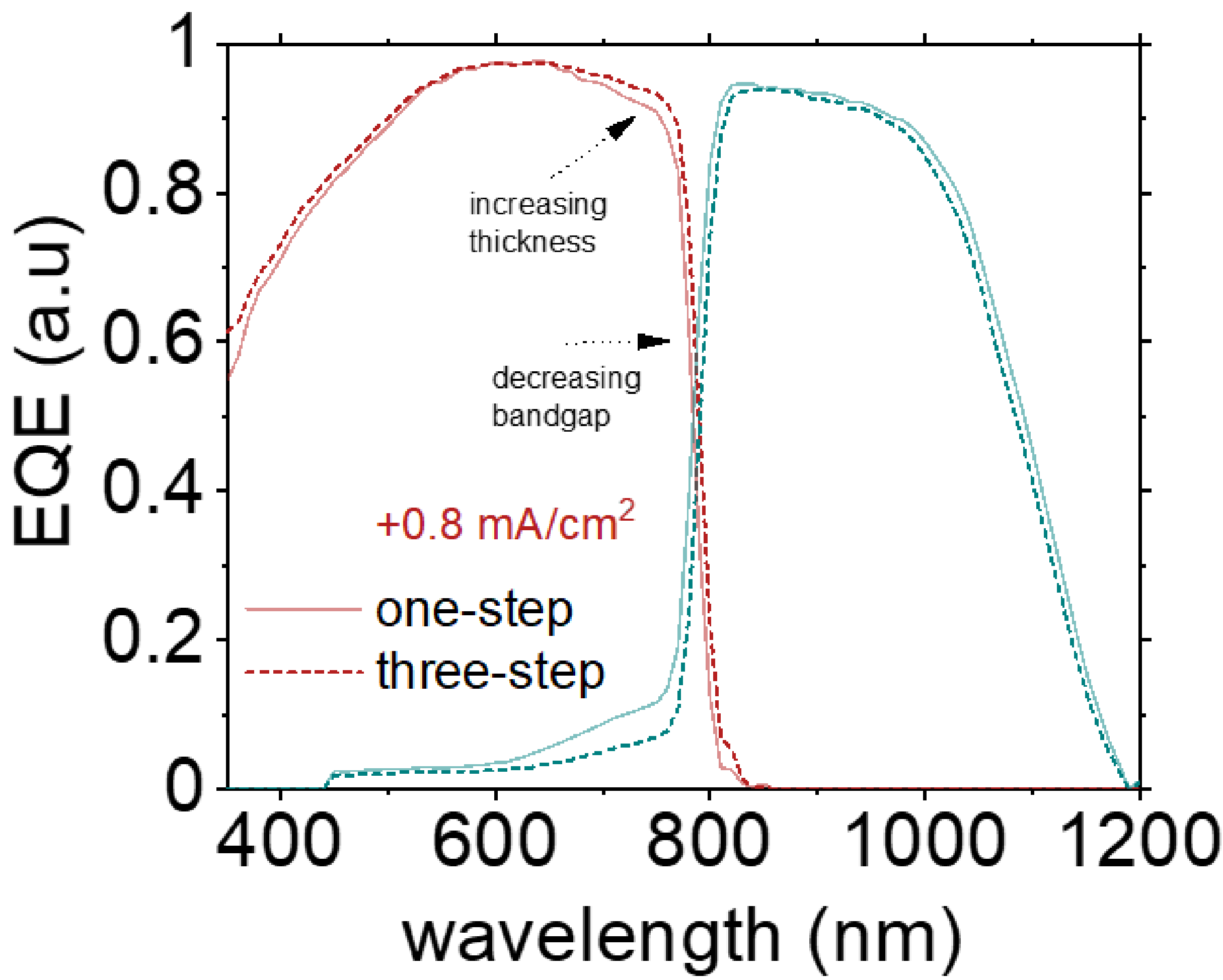

Figure S45. EQE of MBG-Si tandems with one-step and three-step (hybrid on one-step) absorbers.

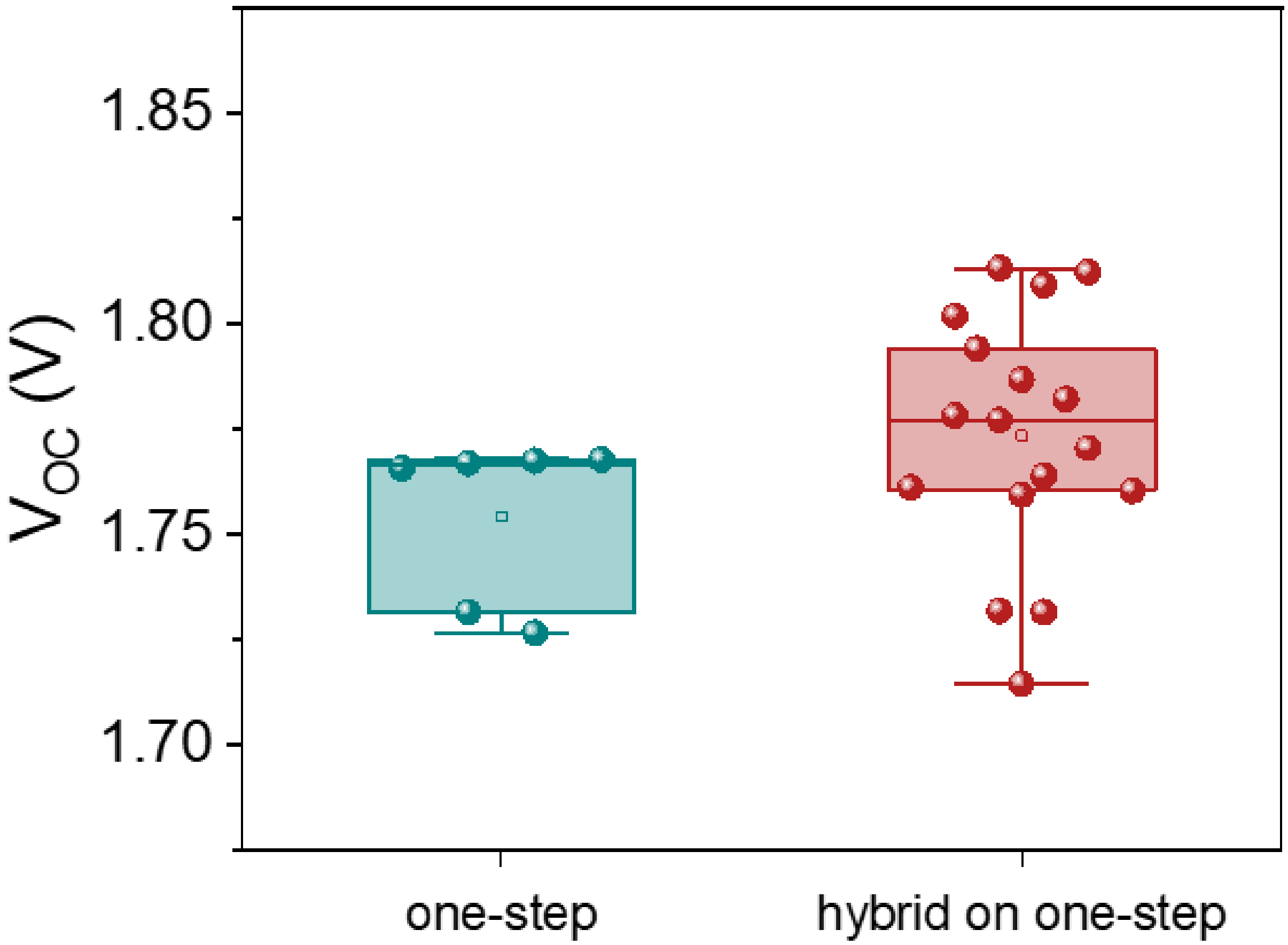

Figure S46. V_{OC} of MBG-Si tandems with one-step and hybrid on one-step absorbers.

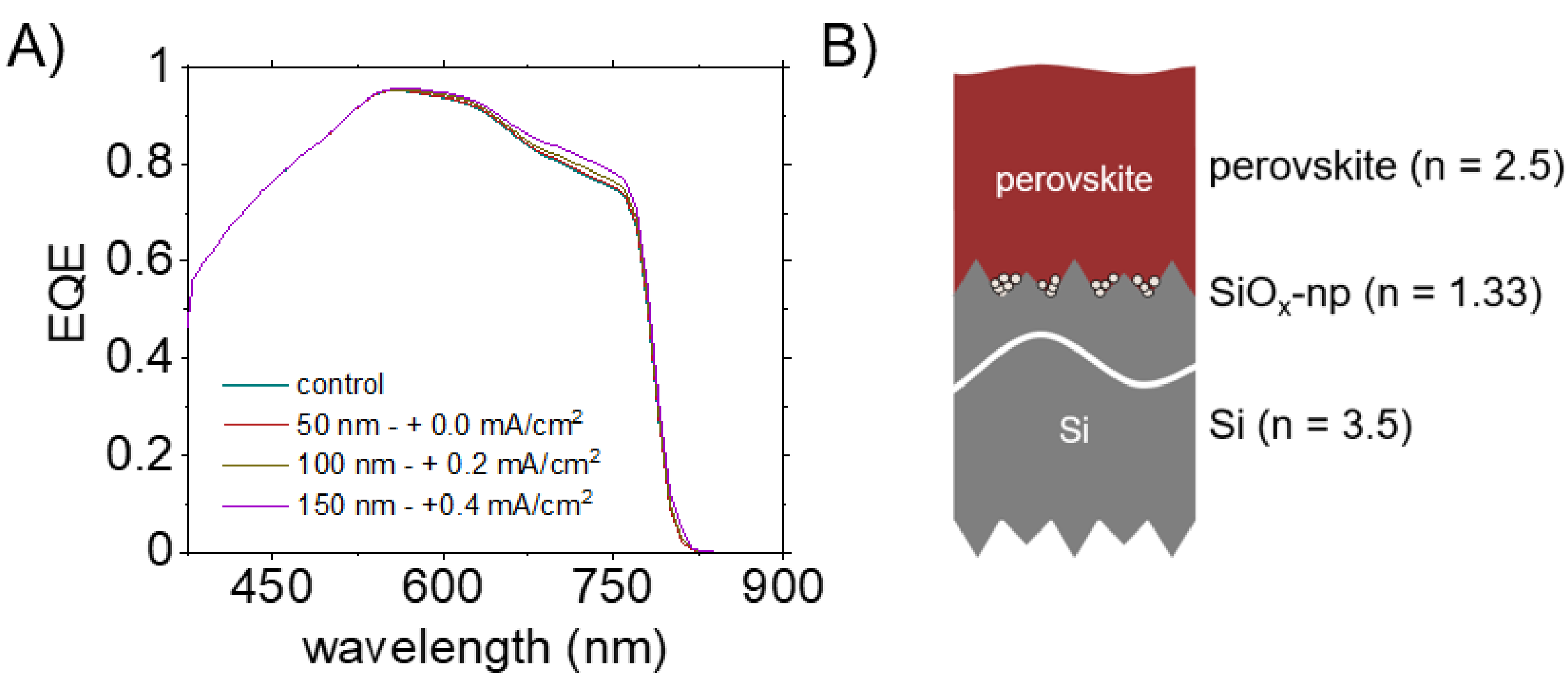

Figure S47. a) COMSOL simulation results for the middle-reflector with different filling ratios. b) Refractive index of perovskite, SiOx-np and Si at 550 nm.

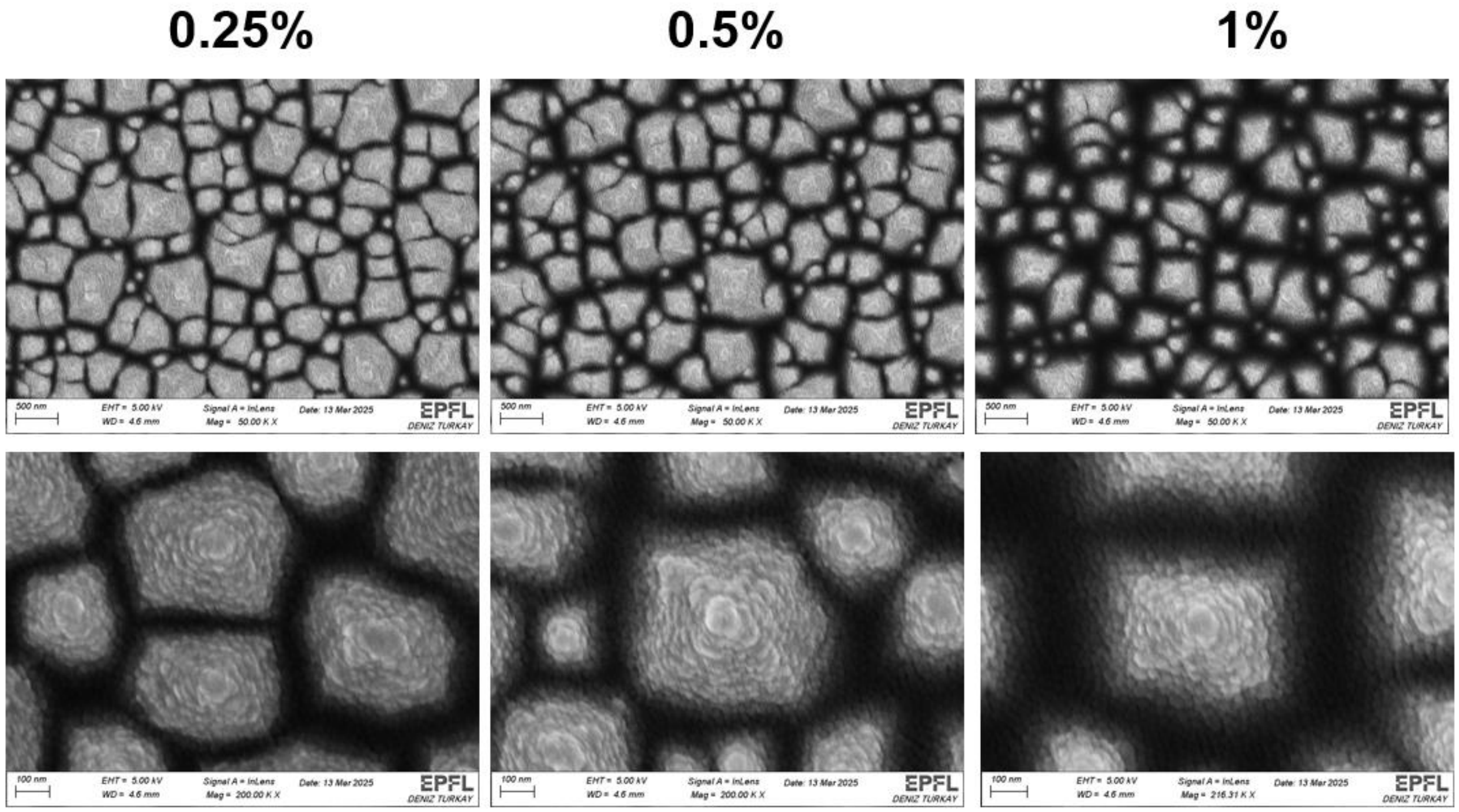

Figure S48. Top-view SEM images of different concentrations of middle-reflector (SiOx-np) on textured c-Si substrates.

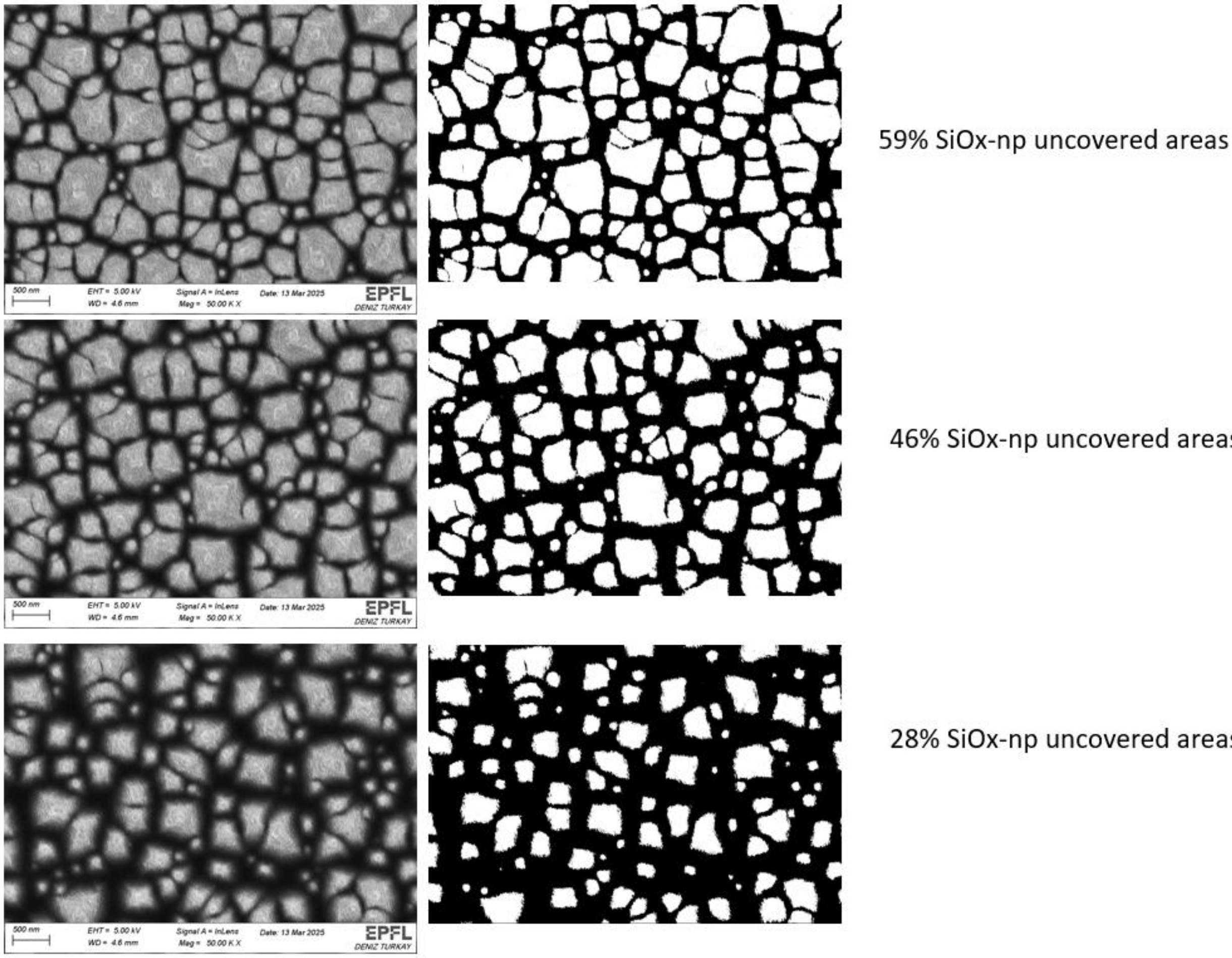

Figure S49. Image analysis of different concentrations of middle-reflector to quantify the SiO_x-np uncovered areas. Filling height (h) is calculated from the percentage of uncovered areas. The height of the pyramid tip (H) is taken as 260 nm.

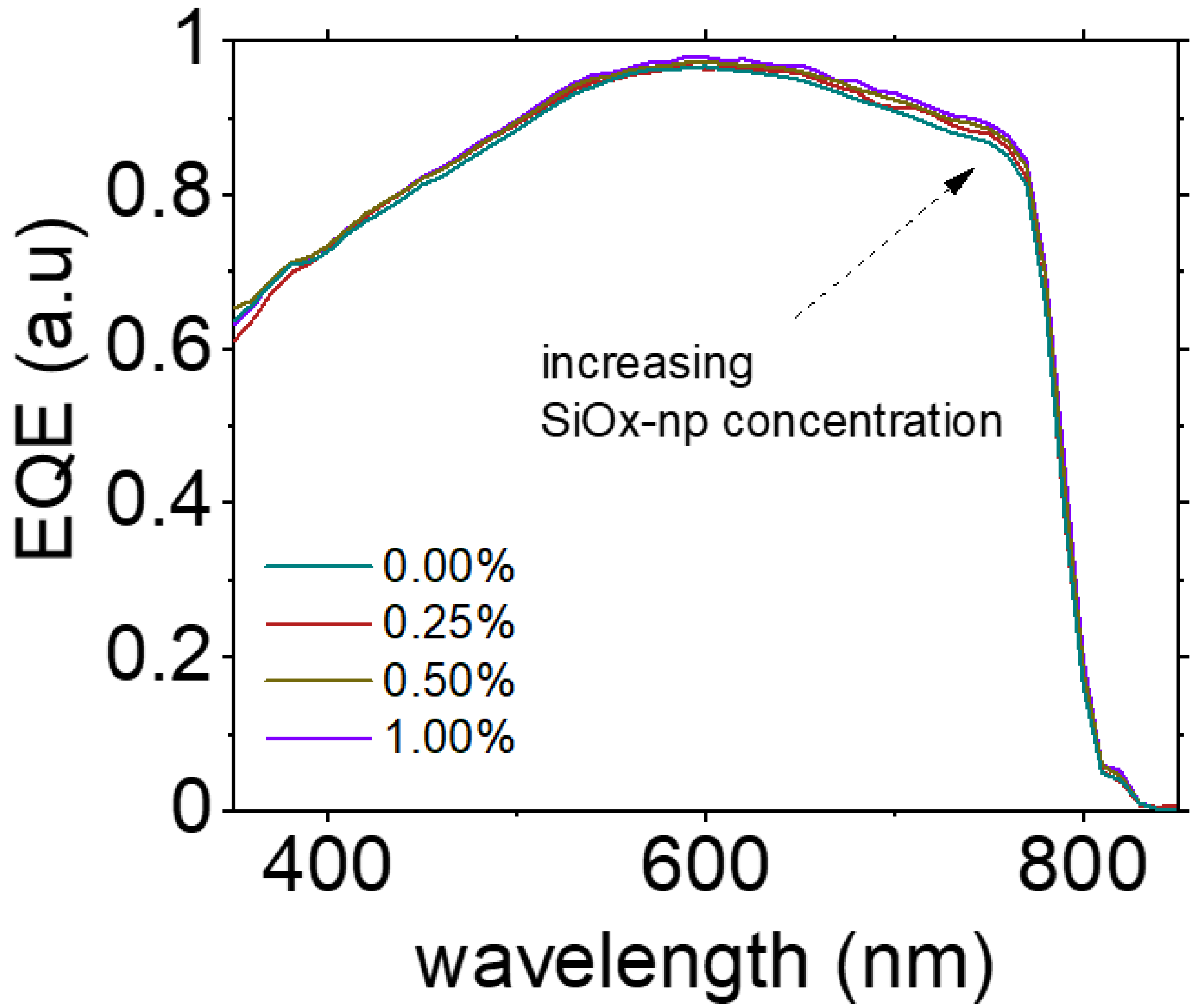

Figure S50. Middle-cell EQE results for MBG-Si tandems with different concentrations of middle-reflector.

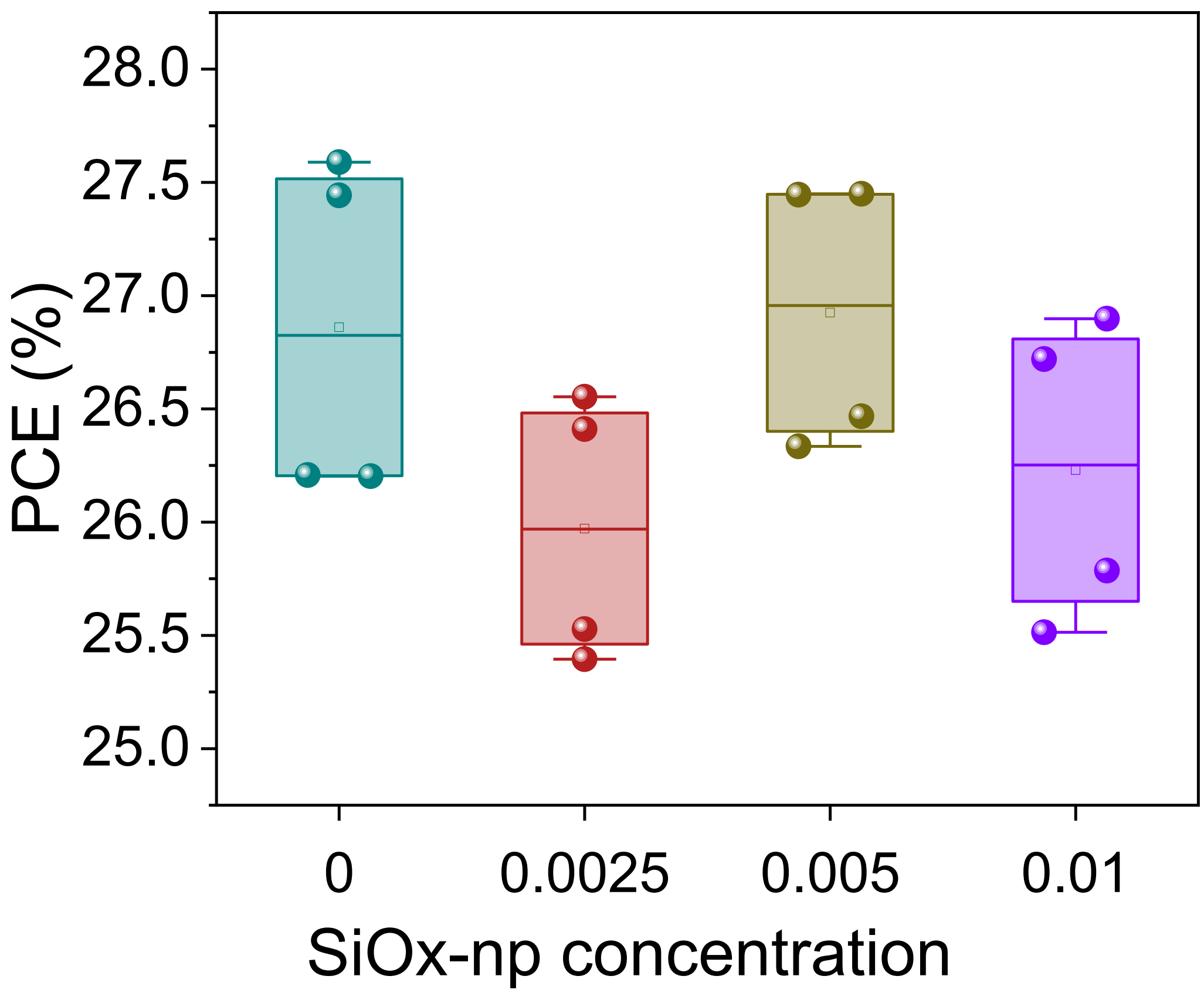

Figure S51. PCE of MBG-Si tandem devices with different concentrations of middle-reflector.

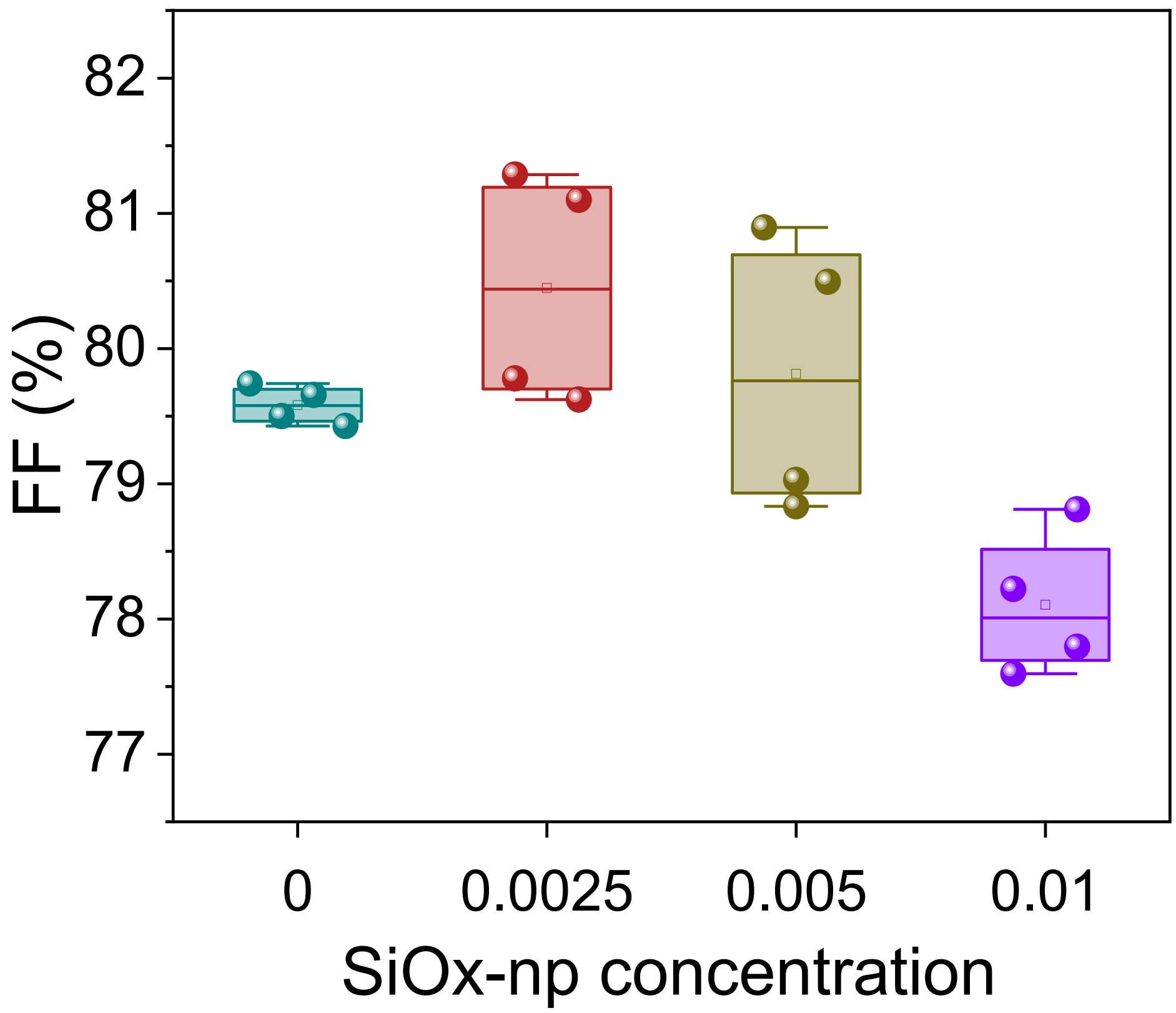

Figure S52. FF of MBG-Si tandems with different concentrations of middle-reflector.

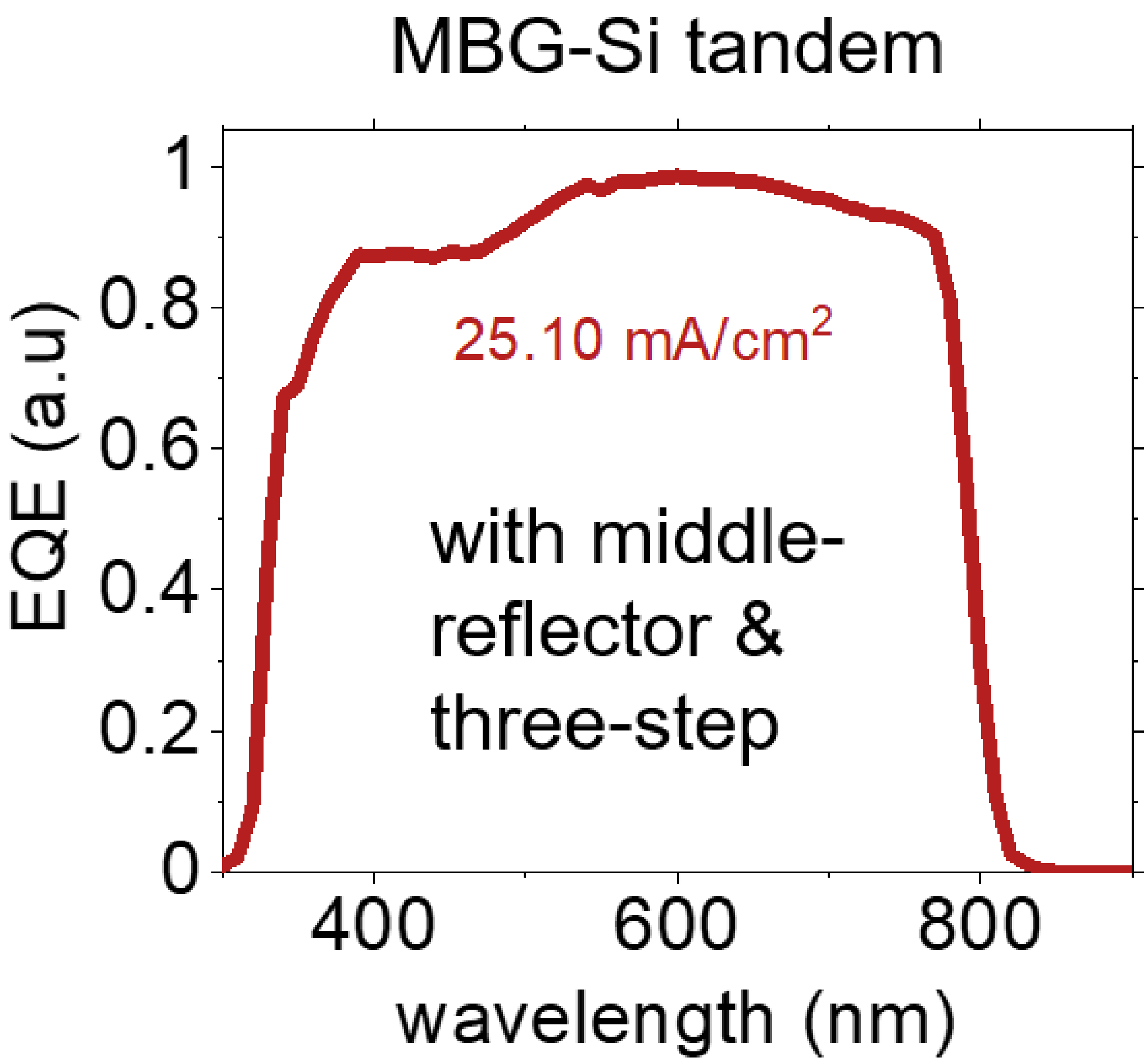

Figure S53. Champion EQE of the middle-cell in MBG-Si tandem with middle-reflector and three-step absorber integrated.

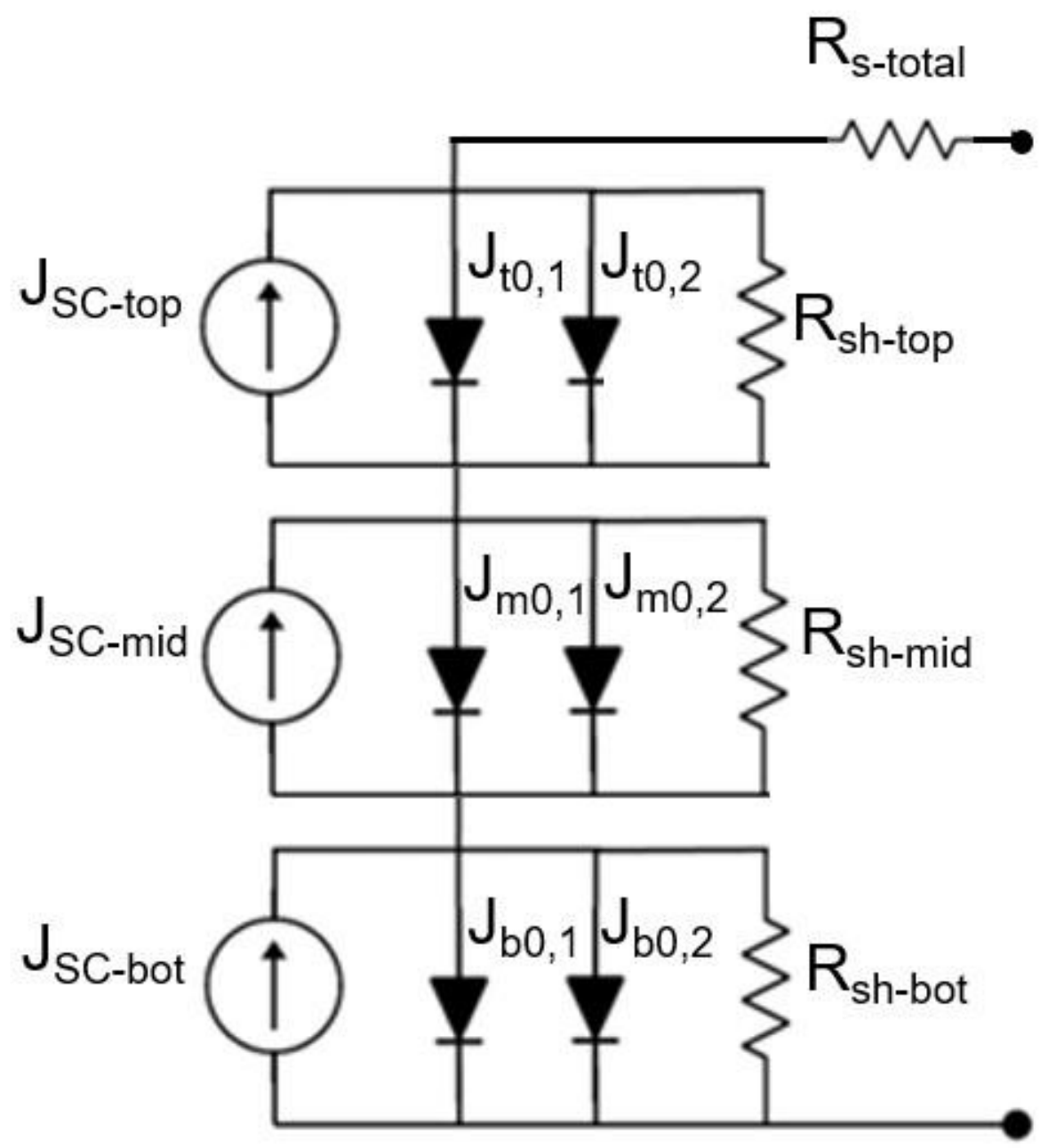

Rs-total (ohm.cm^2)	Rsh-top (ohm.cm^2)	Jt 0,1 (A/cm^2)	Jt 0,2 (A/cm^2)	Rsh-mid (ohm.cm^2)	Jm 0,1 (A/cm^2)	Jm 0,2 (A/cm^2)	Rsh-bot (ohm.cm^2)	Jb 0,1 (A/cm^2)	Jb 0,2 (A/cm^2)
13.5	2×10^{3}	5×10^{-21}	1×10^{-14}	1×10^{5}	5×10^{-18}	1×10^{-14}	5×10^{5}	3×10^{-14}	1×10^{-11}

Figure S54. 3J Equivalent circuit model and the parameters utilized for numerical calculations.

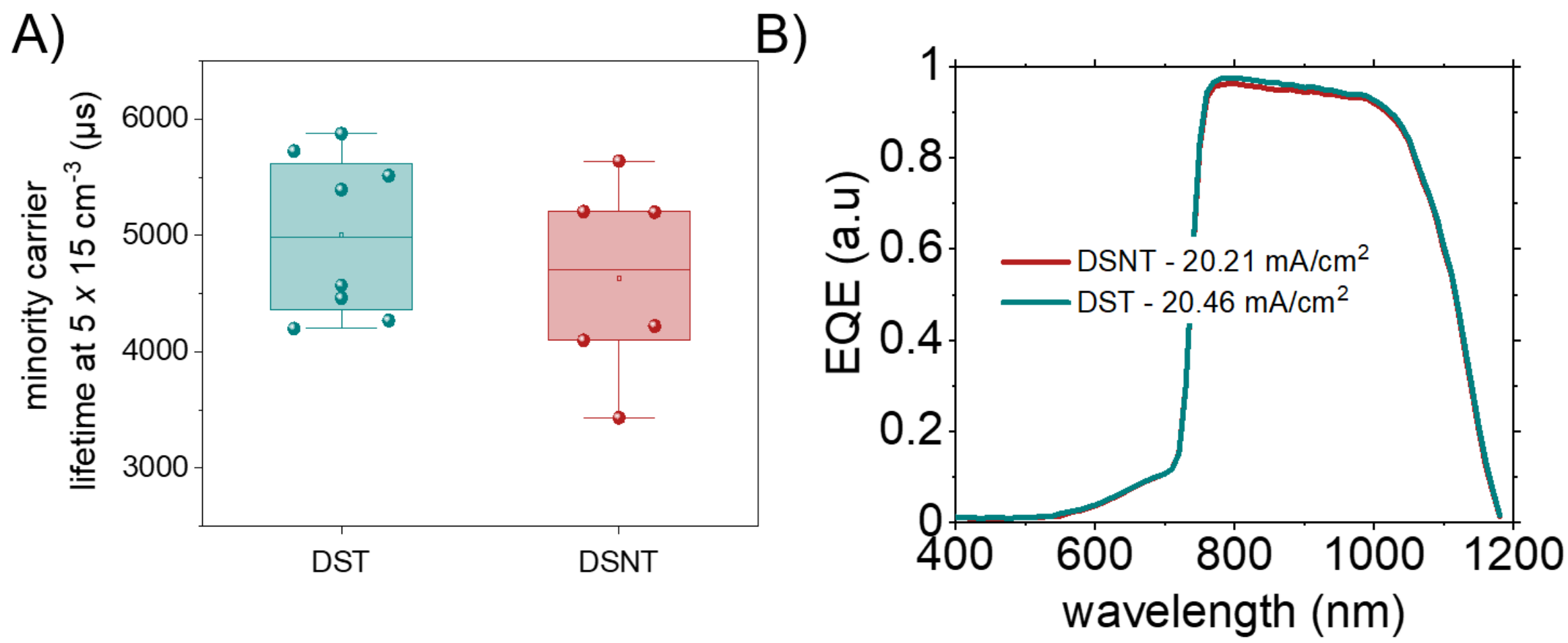

Figure S55. A) Minority carrier lifetime of double-side textured (DST) and double-side nanotextured (DSNT) bottom-cells after PECVD layers. B) Optical performance of DSNT and DST in a perovskite-Silicon tandem. Hence, optoelectronic performance of DST and DSNT bottom-cells are similar.

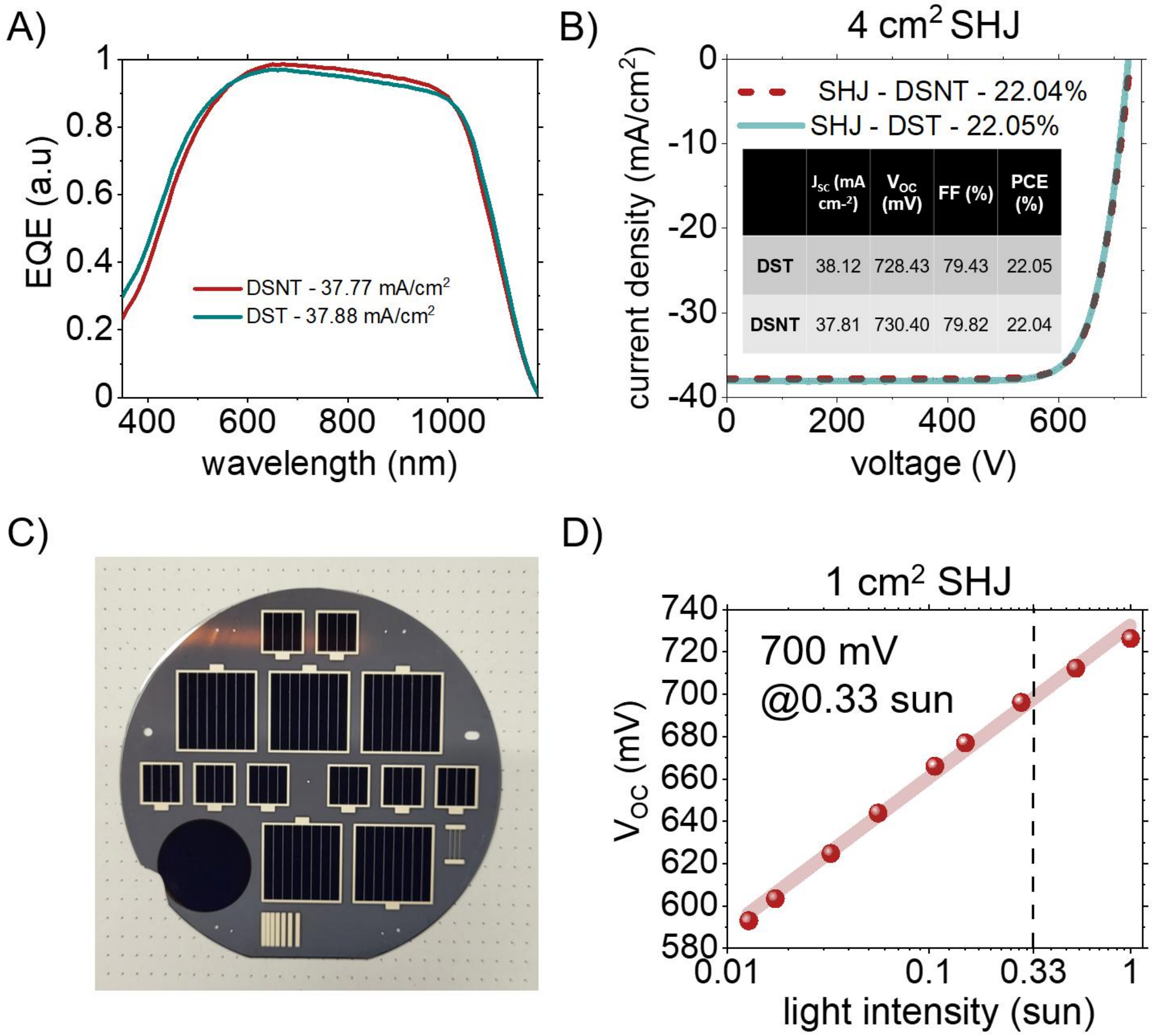

Figure S56. Single-junction c-Si solar cells with SHJ architecture with screen-printed electrodes on 4-inch wafers comparing DST and DSNT. A) EQE, B) JV of the champion devices, C) Optical image of the cell to show the metallization design composed of 1cm^2 and 4cm^2 cells, D) light intensity dependent V_{OC} measurements, highlighting the VOC relevant for 3Js at 0.33sun, 700 mV.

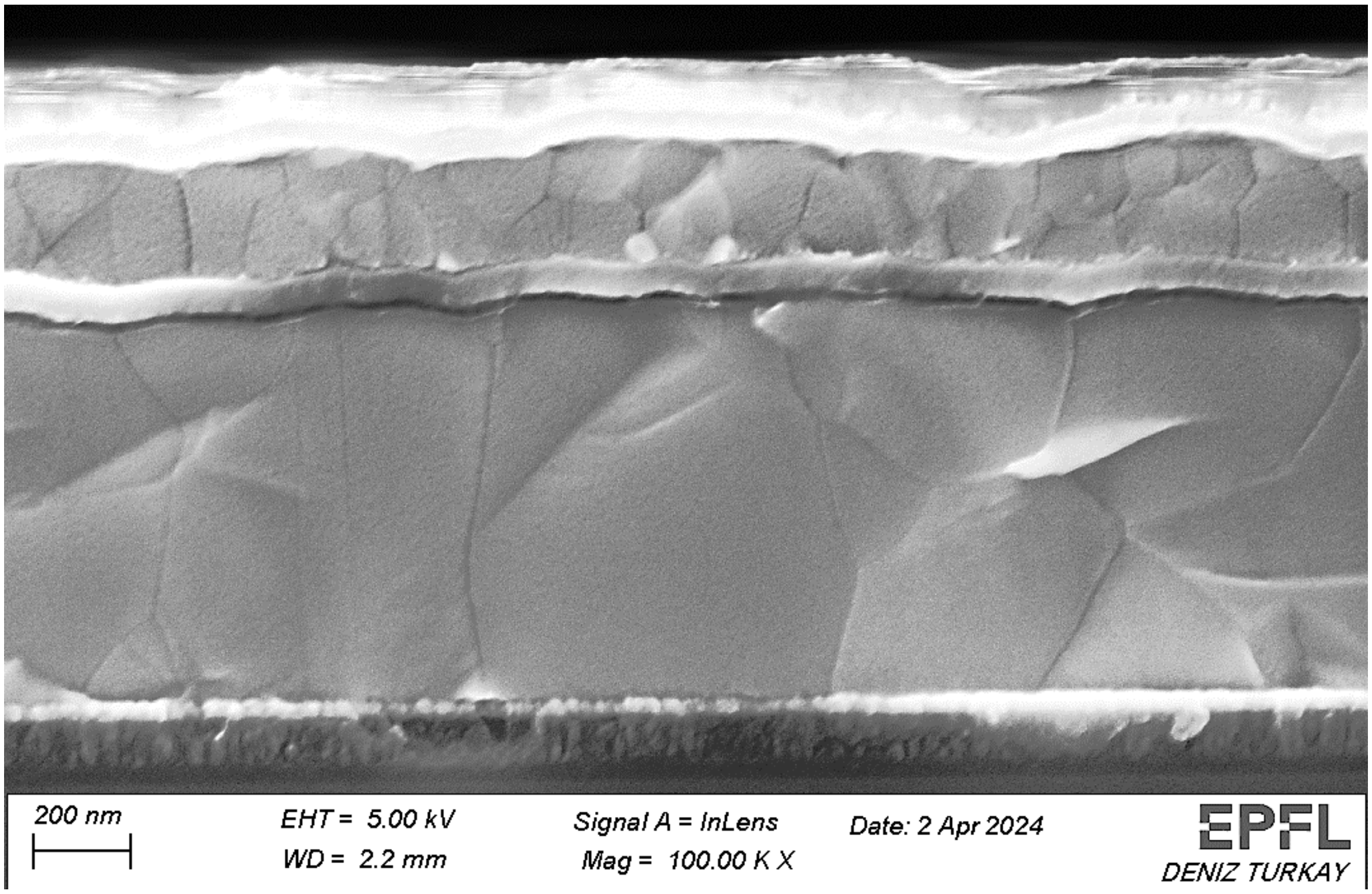

Figure S57. Cross-section SEM image of a triple-junction device on a front-flat Si.

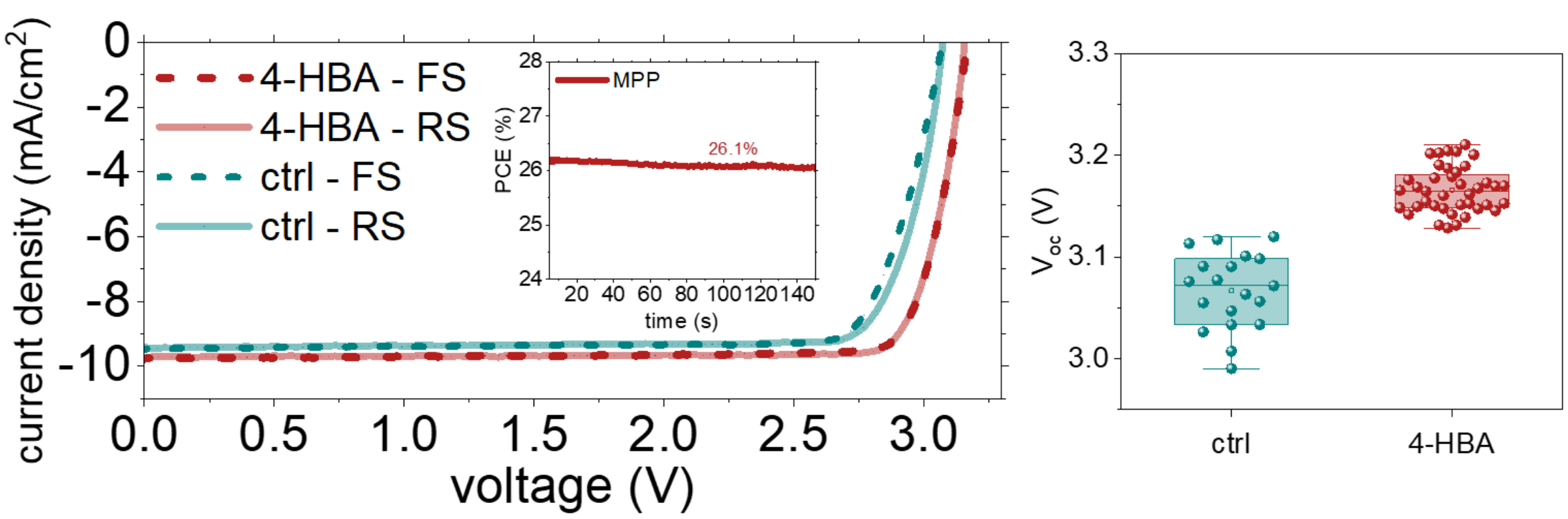

Figure S58. 3J device results with and without 4-HBA additive, prior to optical improvements in the middle-cell. Both reverse and forward scan included.

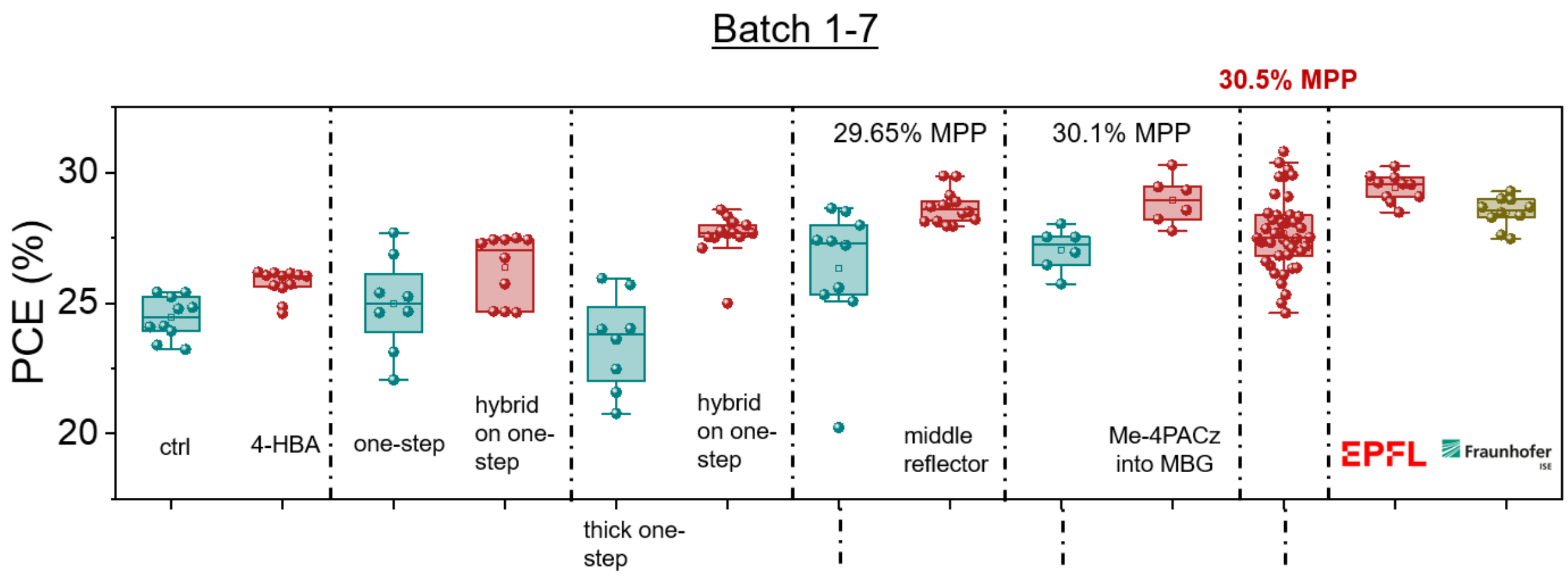

Figure S59. Device performance along 7 batches of fabrications with different conditions were tested. 1st batch is on front-flat Si bottom-cells, and the rest of the batches utilize double-side nanotextured bottom-cells.

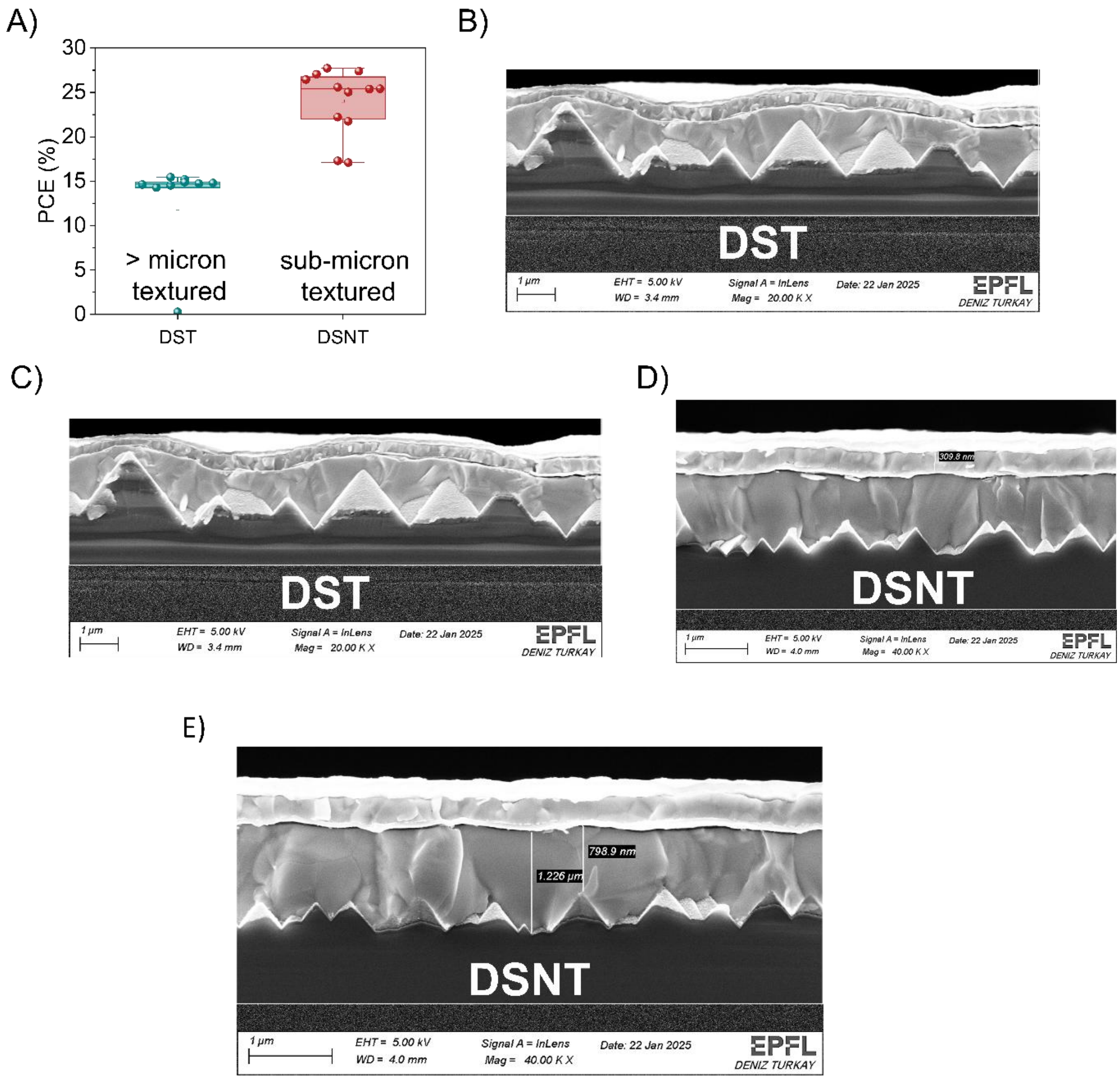

Figure S60. Comparison of triple-junctions on DST and DSNT bottom-cells. A) Performance, B-E) cross-section SEM images of the devices on corresponding bottom-cell. Due to the large pyramid thickness DST configuration suffers from morphological deformation and possible shunt pathways between junctions.

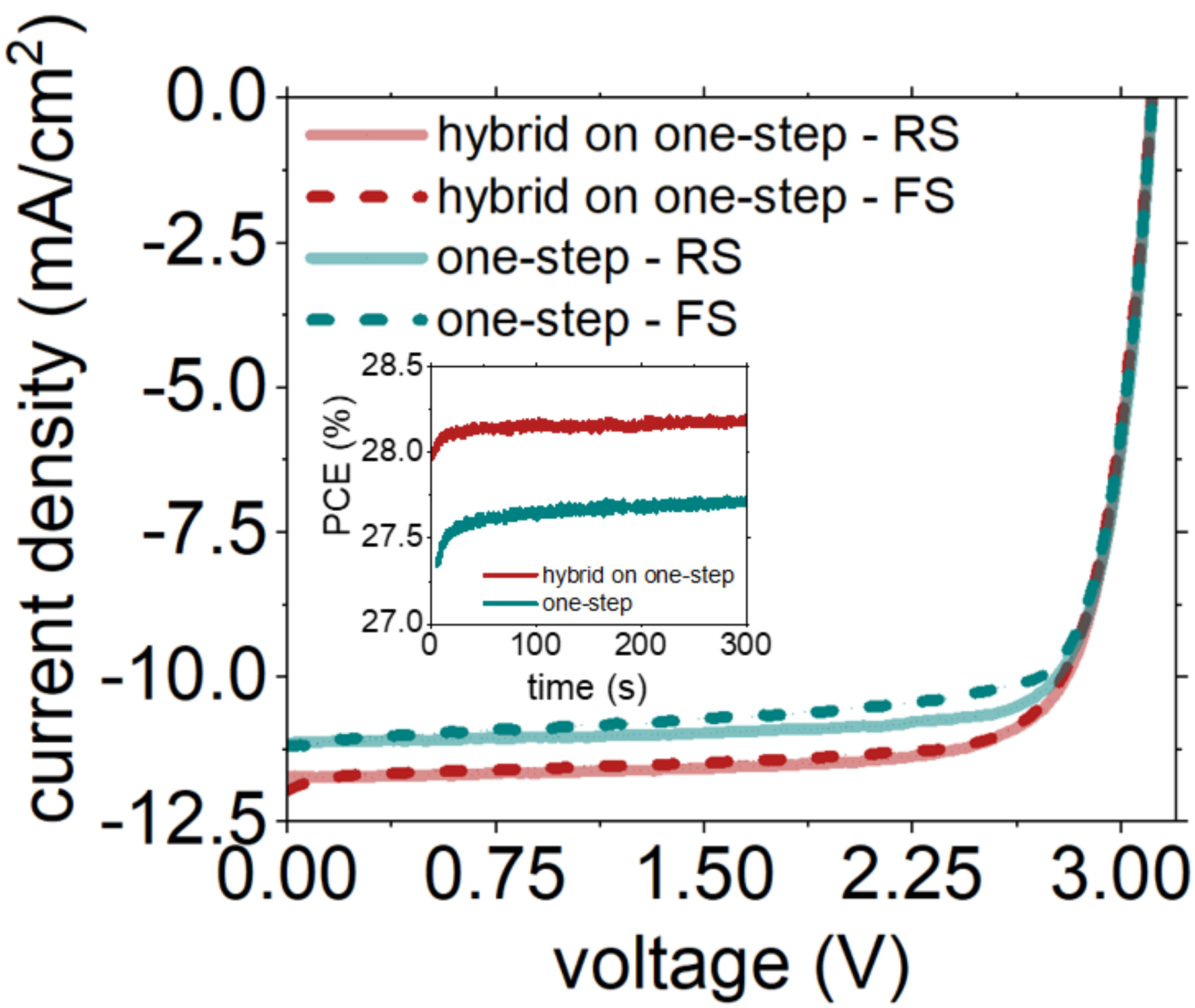

Figure S61. Reverse and forward scan curves of one-step and three absorber comparisons in Batch 2 (inset shows MPP).

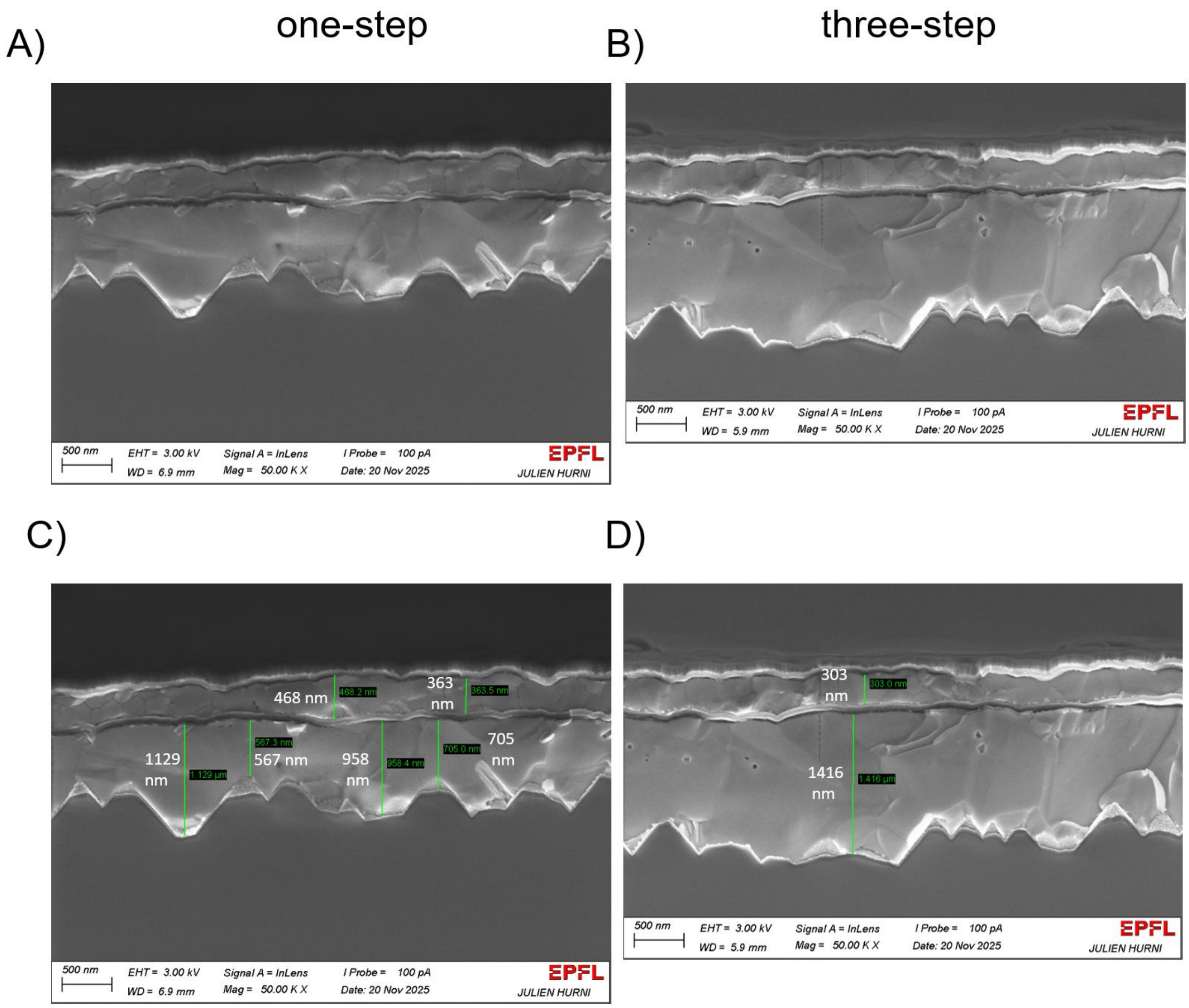

Figure S62. Cross-section SEM comparison of one-step and three-step films. In converted three-step films, residual PbI_2 is not observed. Absorber thickness measured from the valley increases from 1129 nm (one-step) to 1416 nm (three-step absorber). Top-cell thickness is measured between 303 to 468 nm.

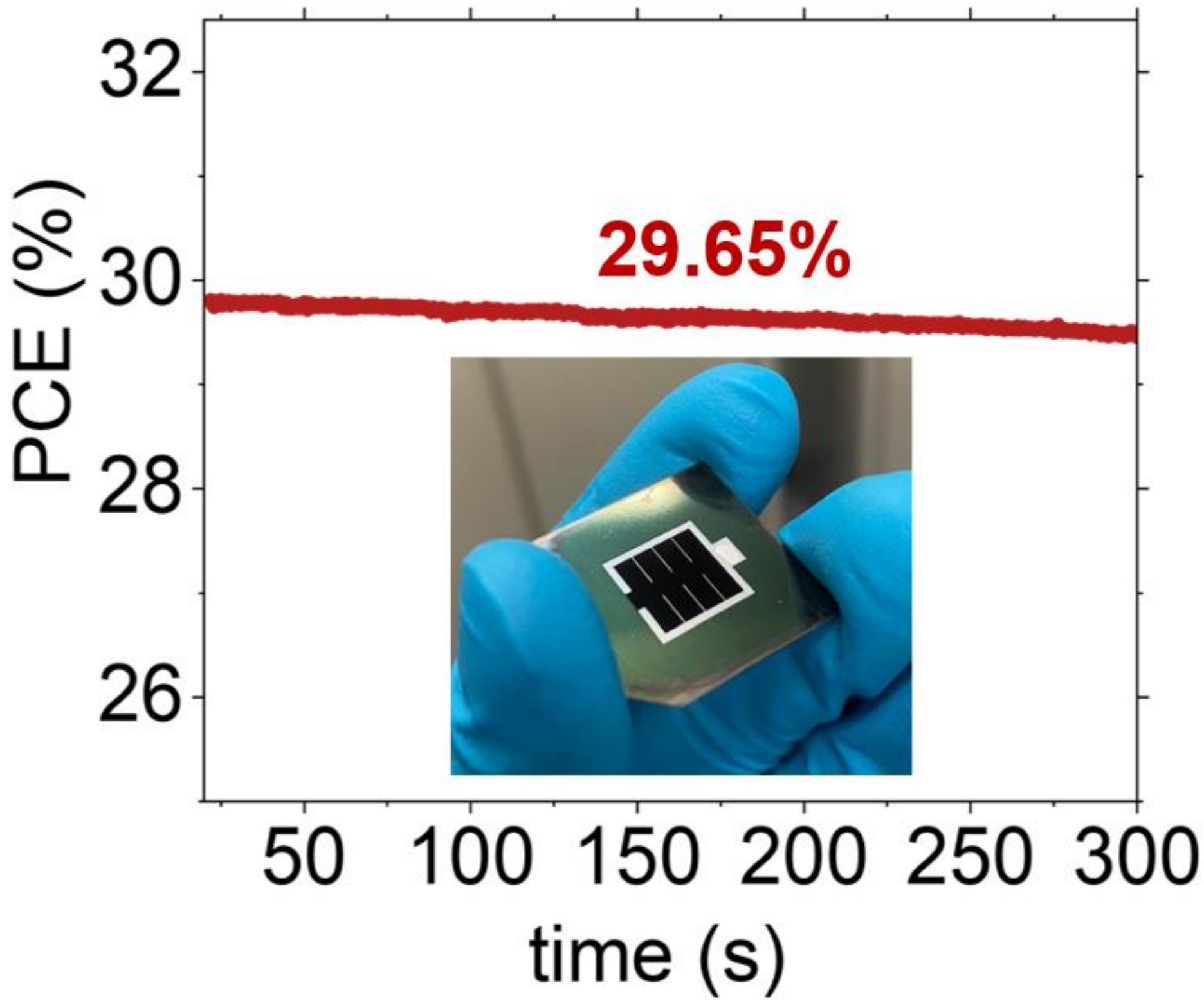

Figure S63. MPP tracking for the champion cell from batch 4.

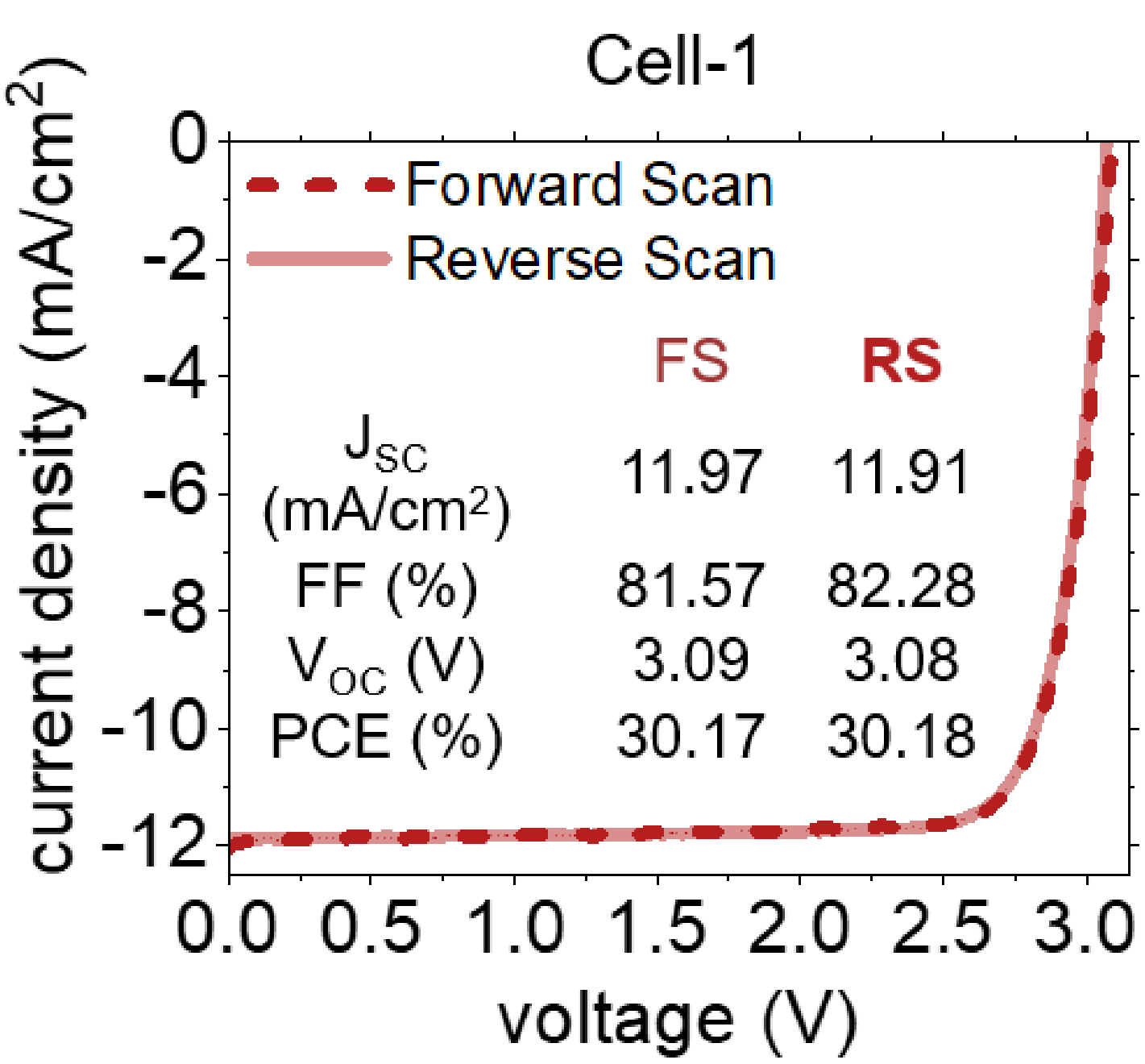

Figure S64. Reverse and forward JV curve of Cell-1.

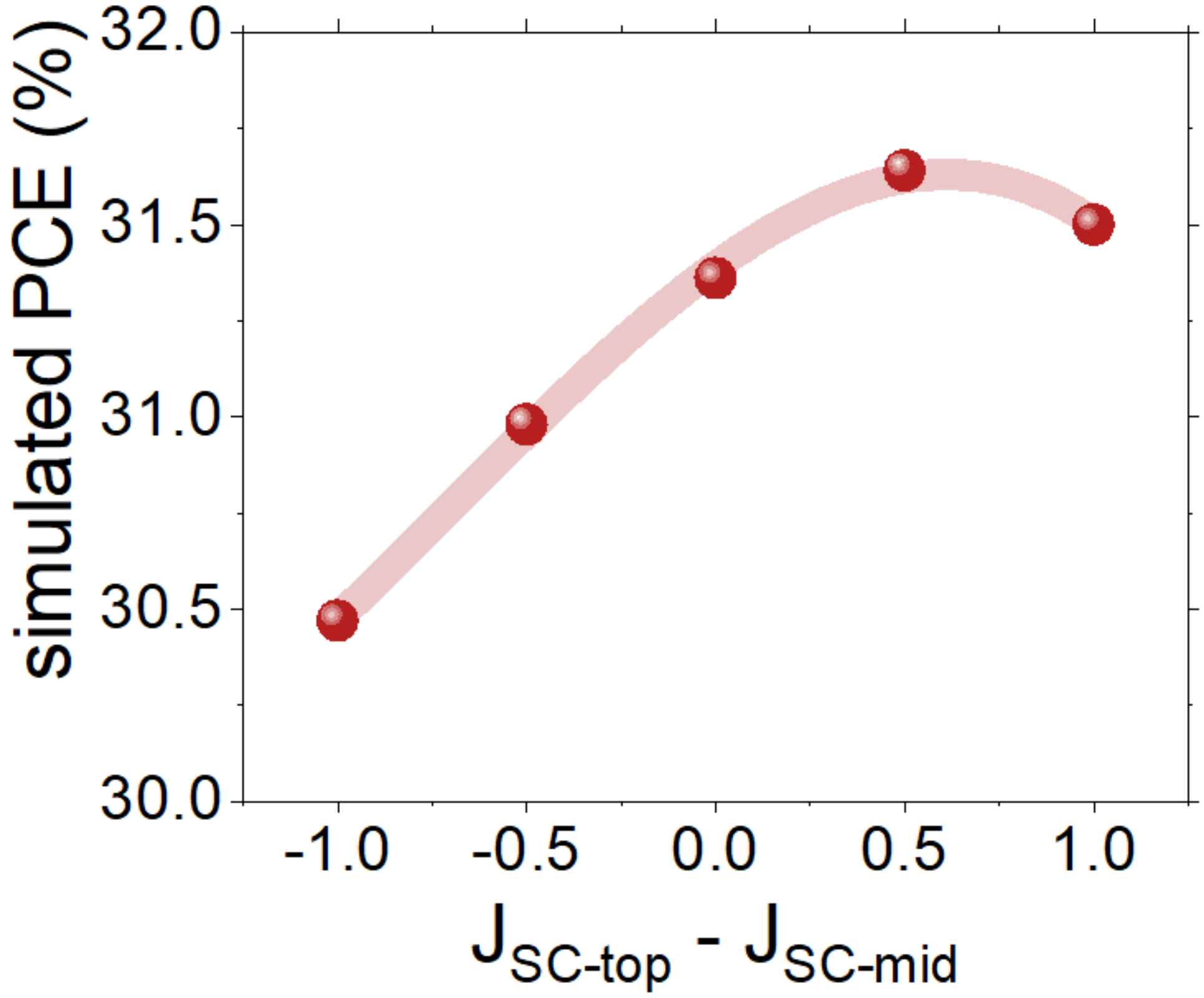

Figure S65. Simulated PCE from circuit modeling with changing mismatch conditions between the top and middle-cell. Optimum performance is achieved at 0.5 mA/cm^2 middle limiting scenario, due to the low shunt resistance of the top-cell compared to the middle-cell. As such, shunt resistance asymmetry increases, and optimum performance points shift more towards the middle-cell.

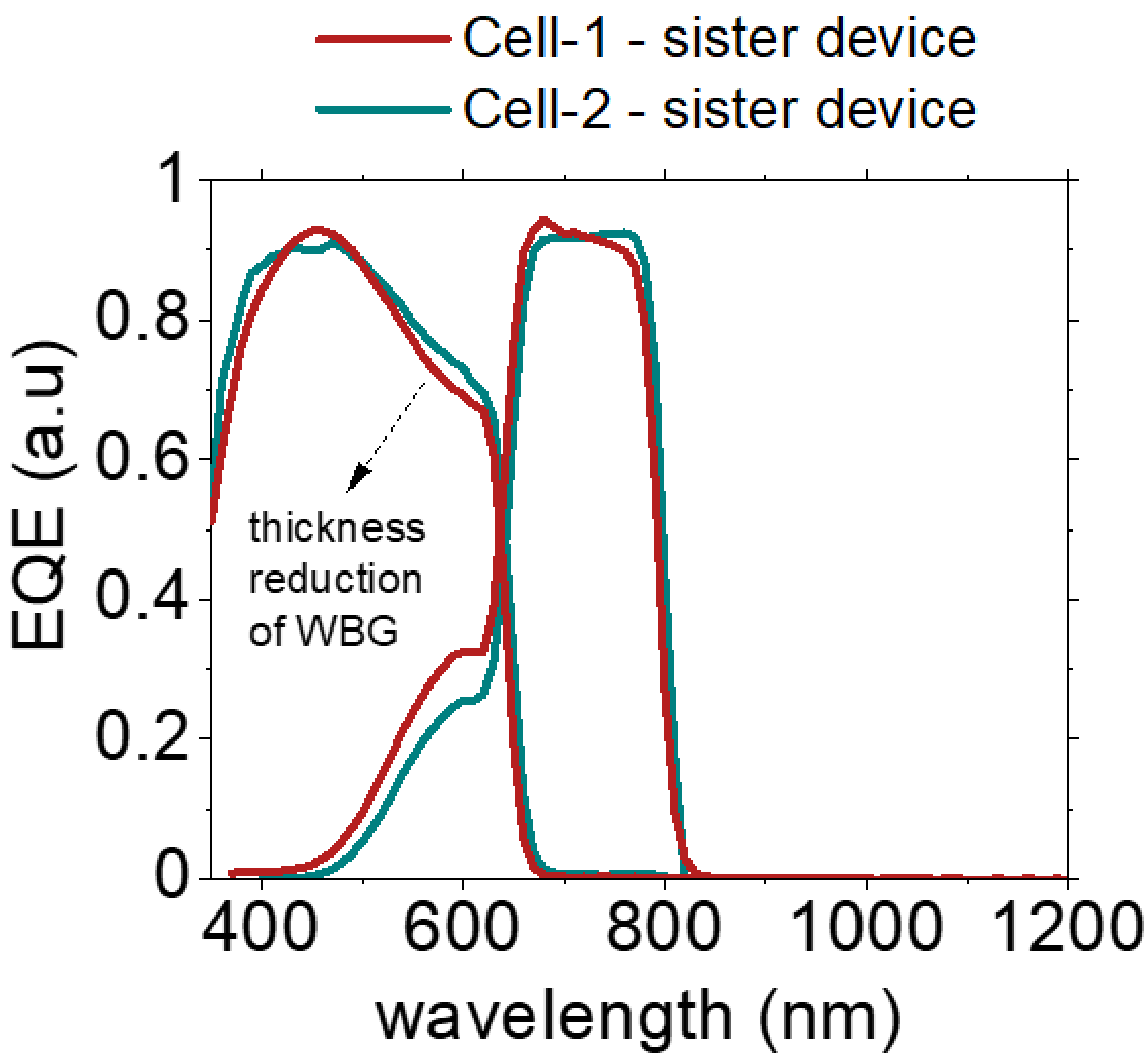

Figure S66. Representative EQE of top and middle-cell for triple-junctions from Batch 5 (same batch as Cell-1) and Batch 6 (same batch as Cell-2), showing decreasing thickness of the top-cell to improve current matching.

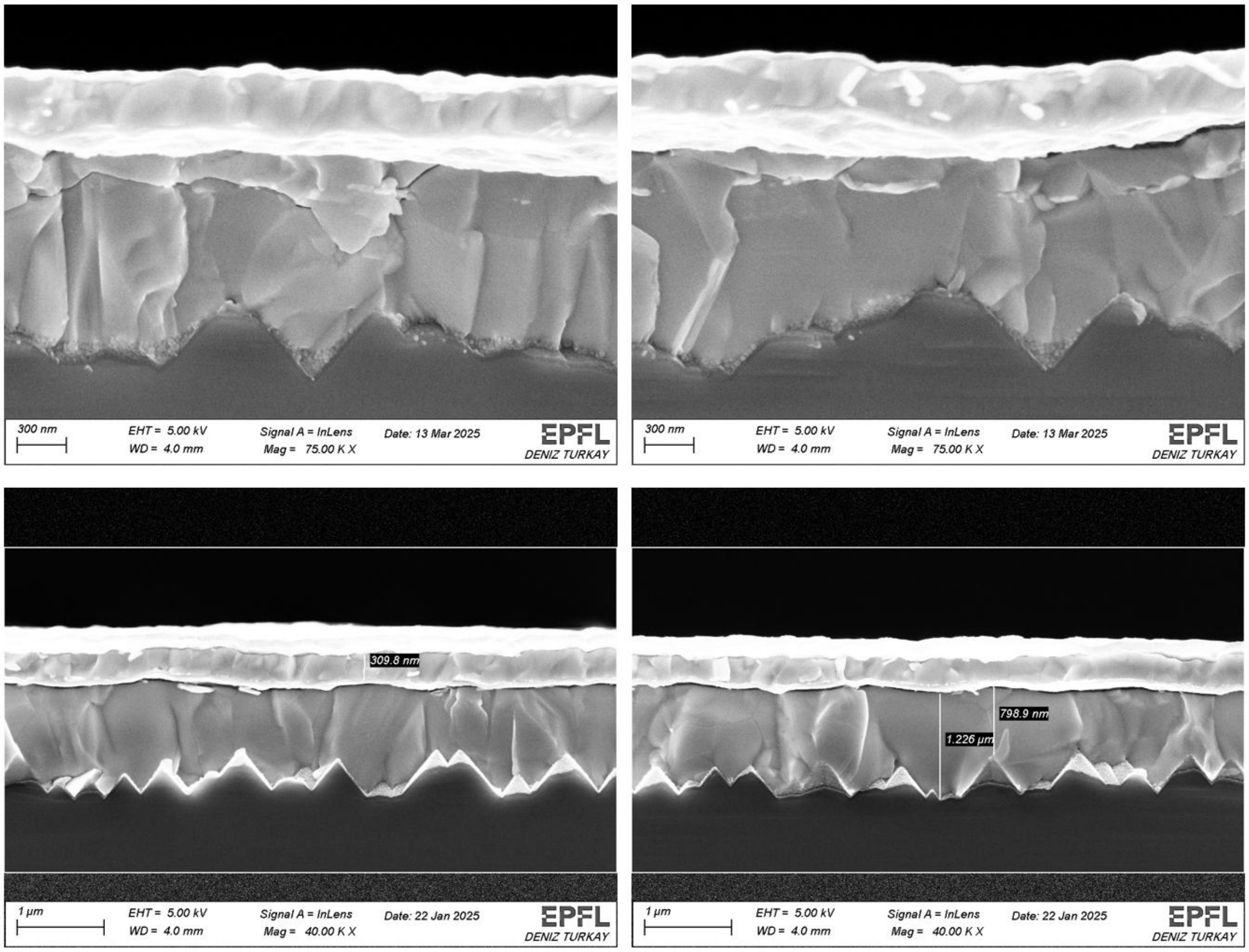

Figure S67. Cross-sectional SEM images of triple-junction devices fabricated on DSNT wafers. **Top row:** Devices employing underconverted three-step absorbers, in which residual PbI_2 within the middle-cell absorber is clearly observed as bright contrast features. The middle reflector, composed of accumulated SiO_x-np at the valleys of the textured Si surface, is also visible. **Bottom row:** Devices incorporating one-step absorbers, exhibiting a more homogeneous middle-cell absorber without detectable residual PbI_2.

We note that despite the visible presence of PbI_2 residuals in this batch of devices, all high-energy photons ($\lambda < 600$ nm) are absorbed in the top subcell. As a consequence, the PbI_2 is not directly exposed to high-energy illumination, which could otherwise (i) contribute to parasitic absorption losses and (ii) accelerate photo-induced degradation pathways. Accordingly, no initial performance deterioration is observed. However, we consider it likely that residual PbI_2 could negatively affect long-term operational stability, particularly under extended thermal and electrical stress, and therefore regard its presence as undesirable from a durability perspective.

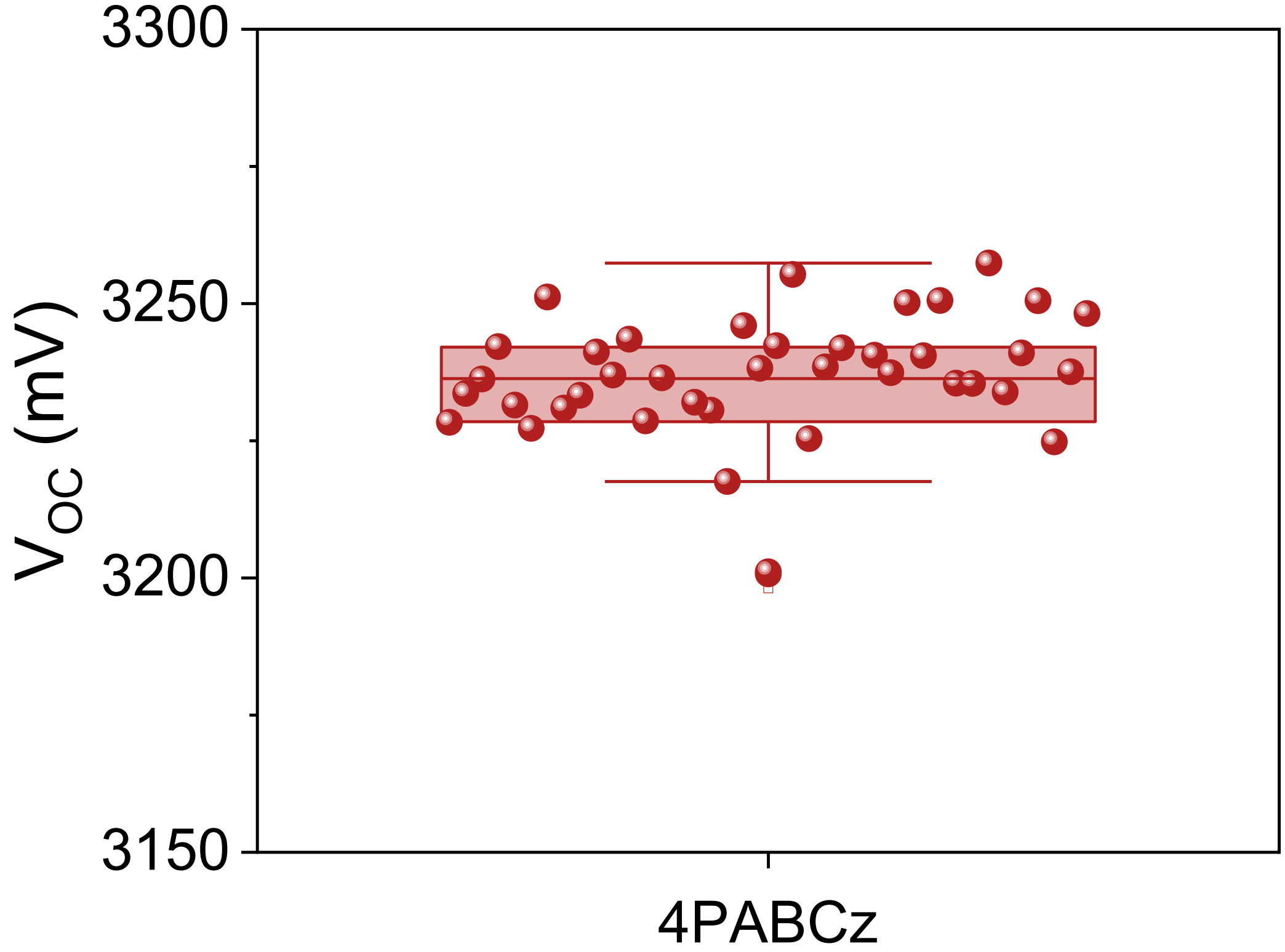

Figure S68. V_{OC} statistics from the champion batch of triple-junction devices with 4PABCz SAM.

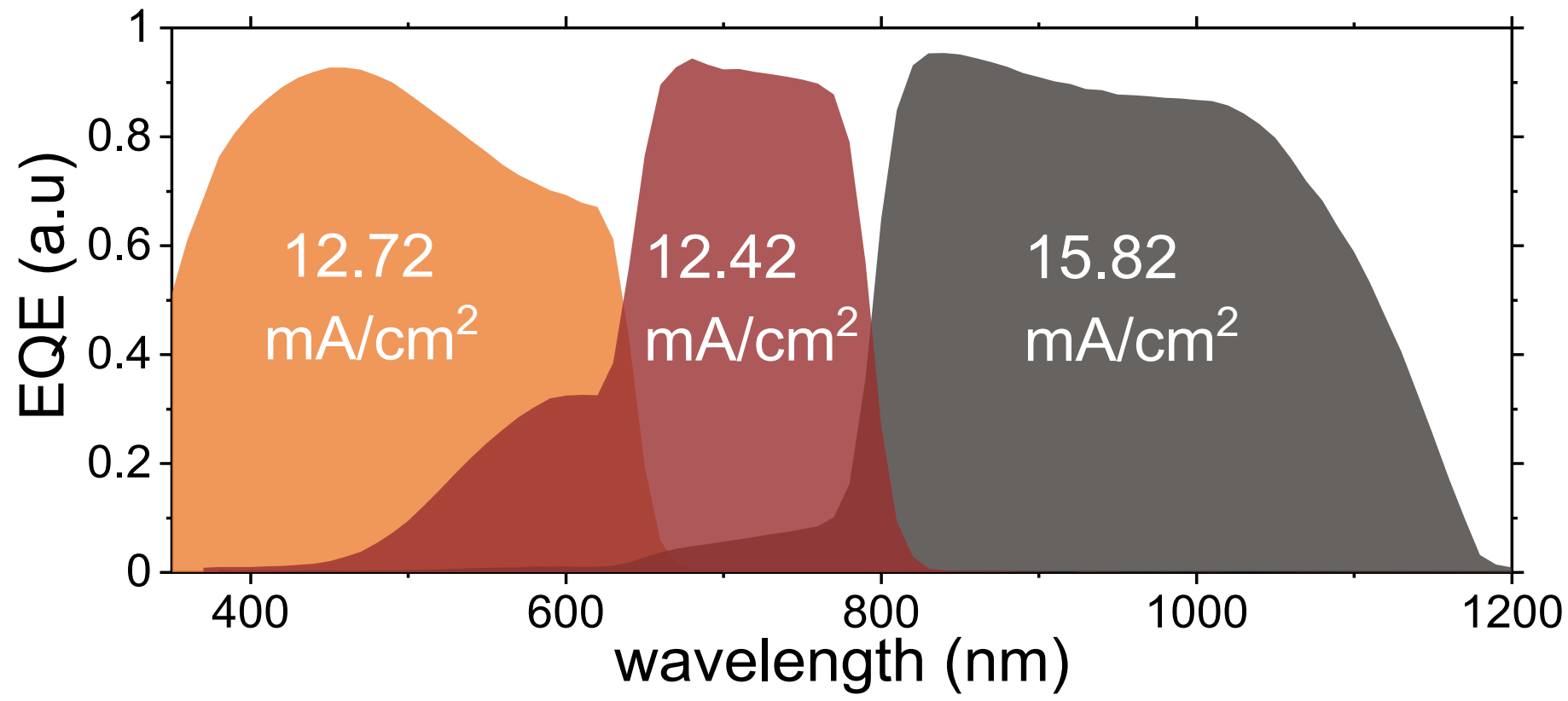

Figure S69. EQE and integrated current densities. The integrated values are reported for completeness and in line with common practice in the perovskite multi-junction literature. However, absolute EQE determination in monolithic multi-junction devices is intrinsically non-trivial. Accurate sub-cell EQE measurement requires selective bias illumination and applied voltage to approximate short-circuit conditions of the targeted junction (*103*, *104*). In series-connected architectures, the individual sub-cell operating point cannot be independently accessed, and the measured EQE amplitude can therefore be influenced by bias-dependent artifacts, non-ideal parallel resistance, luminescent coupling effects (*105*).

In perovskite-containing tandems and triple-junctions, additional dynamic effects may arise from bias-light intensity and measurement conditions, which can modify the apparent EQE amplitude (*104*). While the relative EQE shape remains reliable for the spectral adjustment of solar simulators (*106)*, absolute current densities obtained from EQE integration may not uniquely represent the true current-matching condition within the device. These considerations are well established in the III–V multi-junction community and are equally relevant for perovskite–silicon multijunctions. Accordingly, the integrated values reported here should be interpreted with appropriate caution.

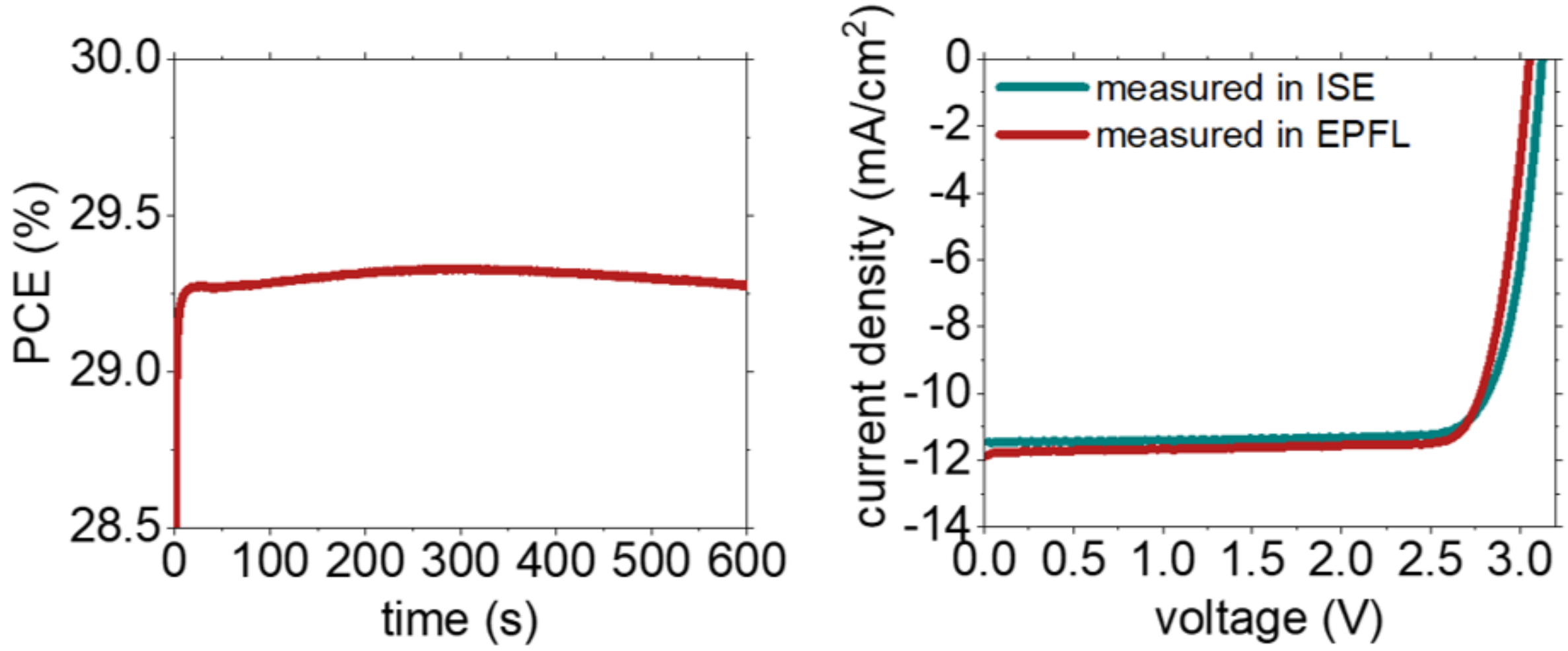

Figure S70. PCE tracking (at fixed voltage) of the champion device measured in Fraunhofer ISE and the comparison of JVs of the same device measured in ISE and EPFL.

Test and Calibration Center of New Energy Device and Module,
Shanghai Institute of Microsystem and Information Technology,
Chinese Academy of Sciences (SIMIT)

中国认可 国际互认 检测 TESTING CNAS L8490

Measurement Report

Report No. 26TR010902

**Client Name**	EPFL PV-Lab & CSEM
**Client Address**	Neuchâtel Switzerland
**Sample**	Perovskite/perovskite/silicon triple-junction solar cell
**Manufacturer**	EPFL PV-Lab & CSEM
**Measurement Date**	9th January, 2026

**Performed by:**	Qiang Shi *Qiang shi*	**Date:** 09/01/2026
**Reviewed by:**	Wenjie Zhao *Wenjie Zhao*	**Date:** 09/01/2026
**Approved by:**	Zhengxin Liu *Zhengxin Liu*	**Date:** 09/01/2026

Address: No.235 Chengbei Road, Jiading, Shanghai — **Post Code:** 201800
E-mail: solarcell@mail.sim.ac.cn — **Tel:** +86-021-69976905

The measurement report without signature and seal are not valid.
This report shall not be reproduced, except in full, without the approval of SIMIT.

**Sample Information**	
Sample Type	Perovskite/perovskite/silicon triple-junction solar cell
Serial No.	3J-3
Lab Internal No.	26010901-2#
Measurement Item	I-V characteristic
Measurement Environment	(24.5±2.0) °C, (38.7±5.0) %R.H

**Measurement of I-V characteristic**	
**Reference cell**	PVM1121, mono-Si, WPVS, calibrated by NIM (Certificate No. GXgf2025-01762)
**Calibration Value/Date of Calibration for Reference cell**	143.3 mA/ May. 2025
**Measurement Conditions**	Standard Test Condition (STC): AM1.5, 1000W/m^2, 25°C
**Measurement Equipment/ Date of Calibration**	AAA Steady State Solar Simulator (YSS-T155-2M) / Oct.2025<br>IV test system (ADCMT 6246) / June. 2025<br>Measuring Microscope (MF-B2017C) / July.2025<br>SR Measurement System (CEP-25ML-CAS) / May.2025
**Measurement Method**	The solar simulator was adjusted to ensure the spectral mismatch factor for each sub-cell remained within 1.000±0.015. Irradiance was set to 1 Sun based on the reference cell calibration value and spectral mismatch factor of the current-limiting junction.<br>A bidirectional logarithmic voltage sweep, from open circuit voltage (Voc) to short circuit current (Isc) and back, was performed within a single flash in accordance with IEC 60904-1:2020.<br>MPP-Tracking was conducted for 300s using the P&O method, with a sampling interval of 1 s. It was considered to have reached steady-state when the rate of change in Pmax during the final 60 seconds was less than ±0.2%/min. The reported results correspond to the average values over the last 30 seconds of the tracking period.
**Measurement Uncertainty**	Area: 1.0%(k=2); Isc: 2.6%(k=2); Voc: 1.0%(k=2);<br>Pmax: 2.9%(k=2); Eff: 3.0%(k=2).

Report No. 26TR010902

====Measurement Results ====

	Forward Scan (Isc to Voc)	Reverse Scan (Voc to Isc)	MPP-Tracking
Area		0.9743 cm²	
Isc	11.377 mA	11.373 mA	/
Voc	3.214 V	3.207 V	/
Pmax	29.430 mW	29.654 mW	29.247 mW
Ipm	10.709 mA	10.713 mA	10.728 mA
Vpm	2.748 V	2.768 V	2.726 V
FF	80.49 %	81.31 %	/
Eff	30.21 %	30.44 %	30.02 %

- Spectral mismatch correction was performed for the current-limiting junction.
- Designated illumination area defined by a thin mask was measured by measuring microscope.
- MPPT results correspond to the average values over the last 30 seconds after steady-state was reached.
- Test results listed in this measurement report refer exclusively to the mentioned measured sample.
- The results apply only at the time of the test, and do not imply future performance.

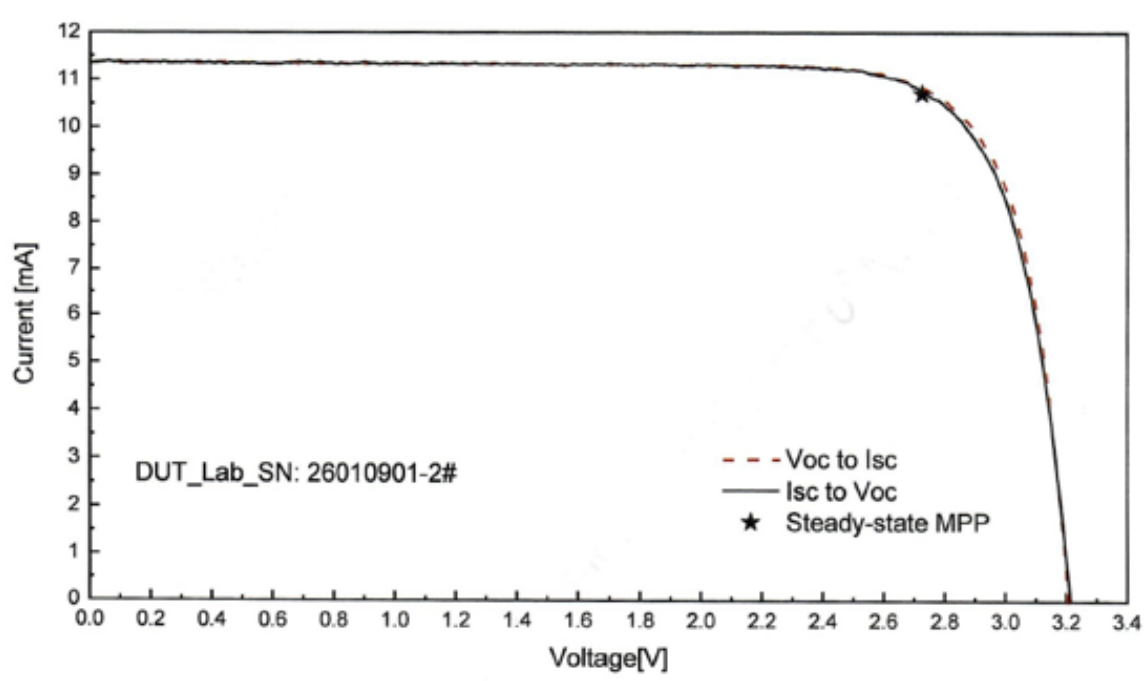

Fig.1 I-V curves of the measured sample

3 / 4

Report No. 26TR010902

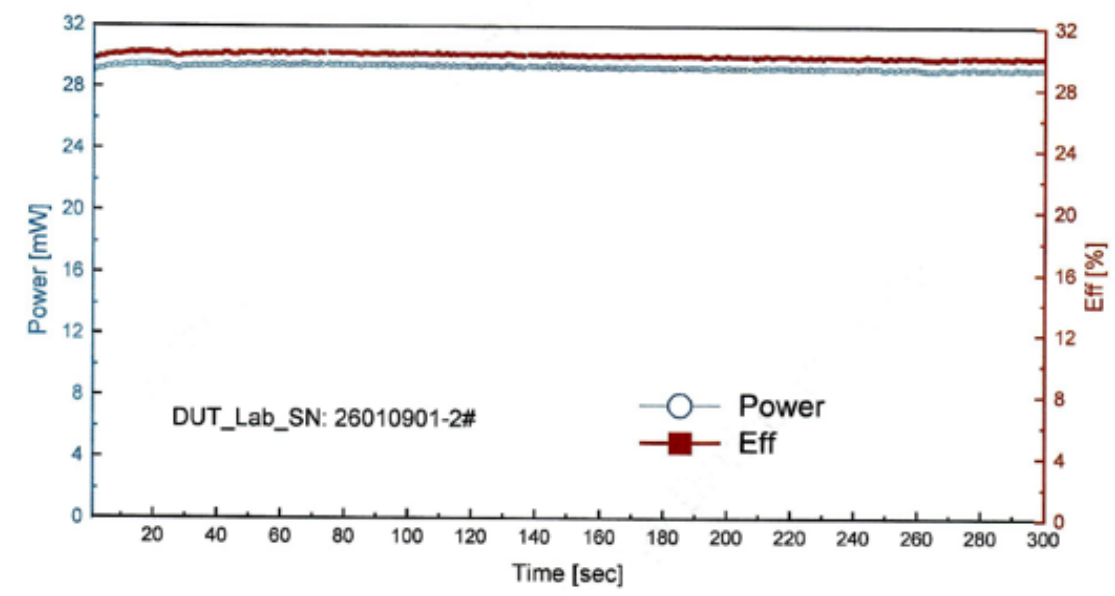

Fig.2 Steady-state maximum power output of the measured sample

-------End of Report-------

Figure S71. Certified device performance of the champion device (*JV* and steady state) from Shanghai Institute of Microsystem and Information Technology (SIMIT).

Test and Calibration Center of New Energy Device and Module,
Shanghai Institute of Microsystem and Information Technology,
Chinese Academy of Sciences (SIMIT)

中国认可
国际互认
检测
TESTING
CNAS L8490

Measurement Report

Report No. 26TR010901

**Client Name**	EPFL PV-Lab & CSEM
**Client Address**	Neuchâtel Switzerland
**Sample**	Perovskite/perovskite/silicon triple-junction solar cell
**Manufacturer**	EPFL PV-Lab & CSEM
**Measurement Date**	9th January, 2026

**Performed by:**	Qiang Shi *Qiang Shi*	**Date:** 09/01/2026
**Reviewed by:**	Wenjie Zhao *Wenjie Zhao*	**Date:** 09/01/2026
**Approved by:**	Zhengxin Liu *Zhengxin Liu*	**Date:** 09/01/2026

Address: No.235 Chengbei Road, Jiading, Shanghai **Post Code:** 201800
E-mail: solarcell@mail.sim.ac.cn **Tel:** +86-021-69976905

The measurement report without signature and seal are not valid.
This report shall not be reproduced, except in full, without the approval of SIMIT.

1 / 4

Report No. 26TR010901

**Sample Information**	
Sample Type	Perovskite/perovskite/silicon triple-junction solar cell
Serial No.	3J-2
Lab Internal No.	26010901-1#
Measurement Item	I-V characteristic
Measurement Environment	(24.5±2.0) °C, (38.7±5.0) %R.H

**Measurement of I-V characteristic**	
**Reference cell**	PVM1121, mono-Si, WPVS, calibrated by NIM (Certificate No. GXgf2025-01762)
**Calibration Value/Date of Calibration for Reference cell**	143.3 mA/ May. 2025
**Measurement Conditions**	Standard Test Condition (STC): AM1.5, 1000W/m², 25°C
**Measurement Equipment/ Date of Calibration**	AAA Steady State Solar Simulator (YSS-T155-2M) / Oct.2025<br>IV test system (ADCMT 6246) / June. 2025<br>Measuring Microscope (MF-B2017C) / July.2025<br>SR Measurement System (CEP-25ML-CAS) / May.2025
**Measurement Method**	The solar simulator was adjusted to ensure the spectral mismatch factor for each sub-cell remained within 1.000±0.015. Irradiance was set to 1 Sun based on the reference cell calibration value and spectral mismatch factor of the current-limiting junction.<br>A bidirectional logarithmic voltage sweep, from open circuit voltage (Voc) to short circuit current (Isc) and back, was performed within a single flash in accordance with IEC 60904-1:2020.<br>MPP-Tracking was conducted for 300s using the P&O method, with a sampling interval of 1 s. It was considered to have reached steady-state when the rate of change in Pmax during the final 60 seconds was less than ±0.2%/min. The reported results correspond to the average values over the last 30 seconds of the tracking period.
**Measurement Uncertainty**	Area: 1.0%(k=2); Isc: 2.6%(k=2); Voc: 1.0%(k=2);<br>Pmax: 2.9%(k=2); Eff: 3.0%(k=2).

2 / 4

Report No. 26TR010901

====Measurement Results ====

	Forward Scan (Isc to Voc)	Reverse Scan (Voc to Isc)	MPP-Tracking
Area		0.9743 cm²	
Isc	11.385 mA	11.388 mA	/
Voc	3.215 V	3.201 V	/
Pmax	29.220 mW	29.406 mW	28.993 mW
Ipm	10.694 mA	10.712 mA	10.690 mA
Vpm	2.732 V	2.745 V	2.712 V
FF	79.82 %	80.66 %	/
Eff	29.99 %	30.18 %	29.76 %

- Spectral mismatch correction was performed for the current-limiting junction.
- Designated illumination area defined by a thin mask was measured by measuring microscope.
- MPPT results correspond to the average values over the last 30 seconds after steady-state was reached.
- Test results listed in this measurement report refer exclusively to the mentioned measured sample.
- The results apply only at the time of the test, and do not imply future performance.

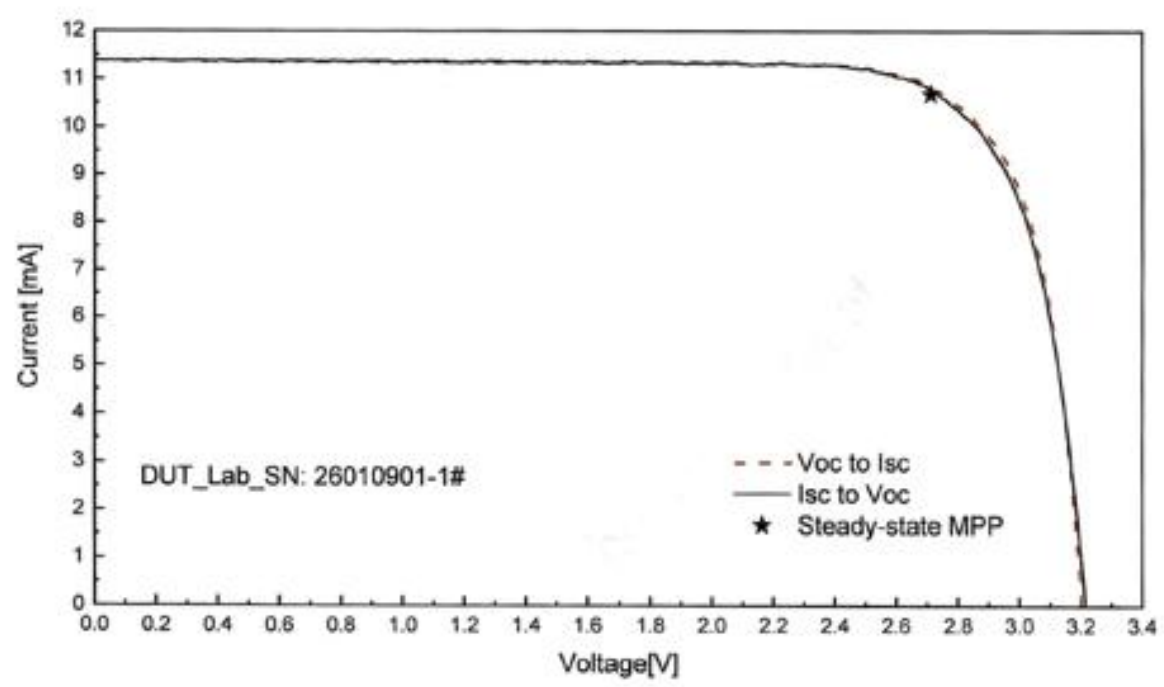

Fig.1 I-V curves of the measured sample

3 / 4

Report No. 26TR010901

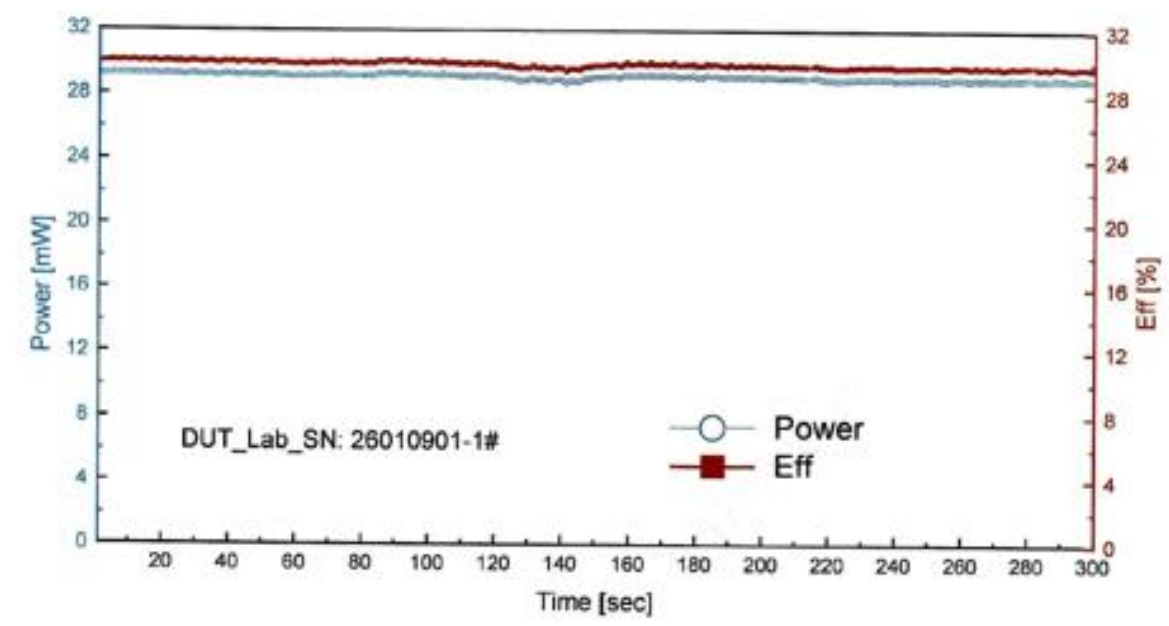

Fig.2 Steady-state maximum power output of the measured sample

-------End of Report-------

Figure S72. Certified device performance of the second-best device (*JV* and steady state) from SIMIT.

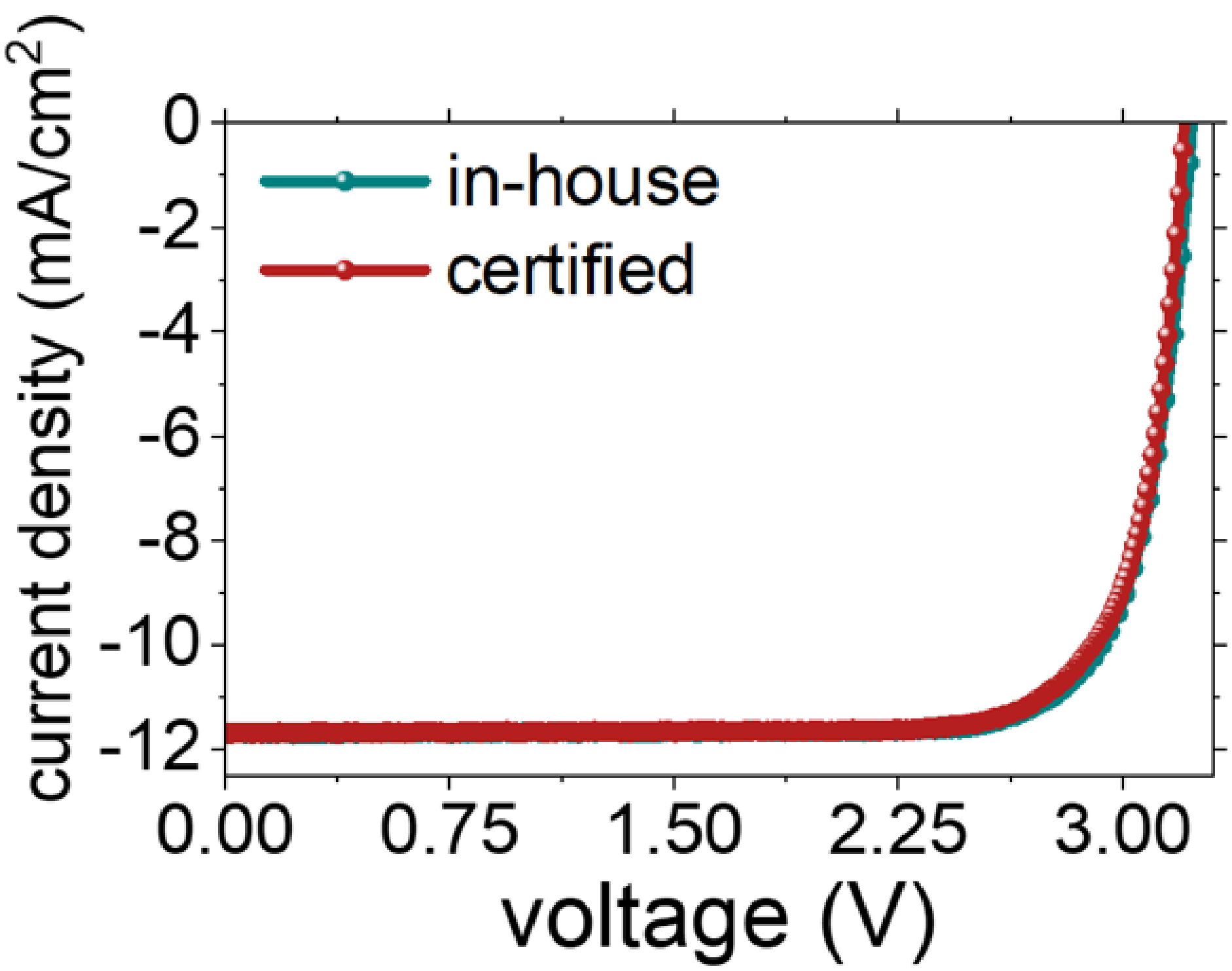

	in-house	certified
J_{SC} (mA/cm²)	11.69	11.67
FF (%)	80.45	81.31
V_{OC} (V)	3.233	3.207
PCE (%)	**30.41**	**30.44**
MPP (%)	**30.10**	**30.02**

Figure S73. Comparison of the *JV* curves of the certified solar cell (Cell-3) measured in-house prior to shipment and the independently certified *JV* curve.

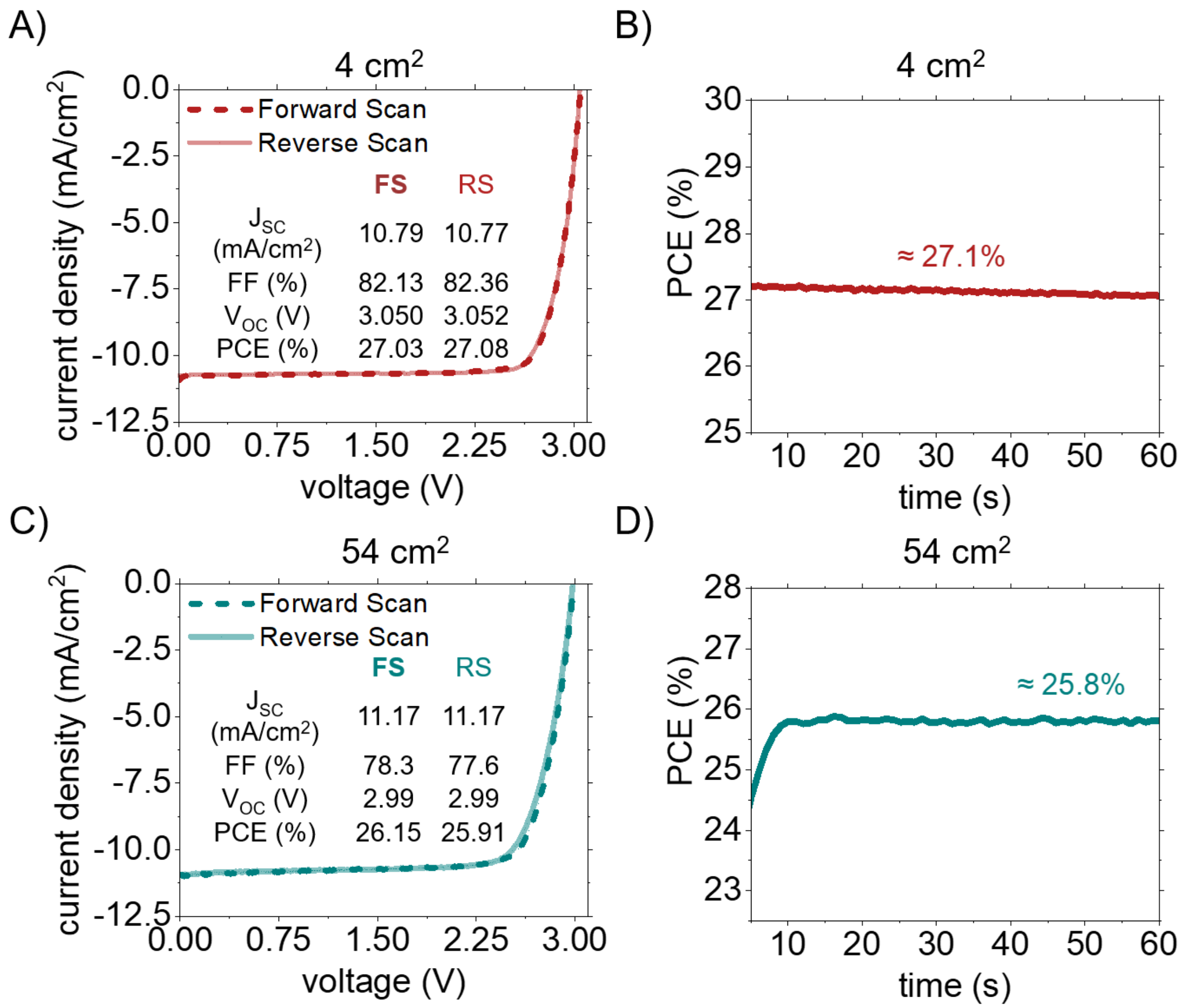

Figure S74. Champion large-area devices fabricated by processing 4-inch wafers. A) *JV* curves, B) MPP for the champion 4 cm^2, C) *JV* curves, D) MPP for champion 54 cm^2 triple-junctions.

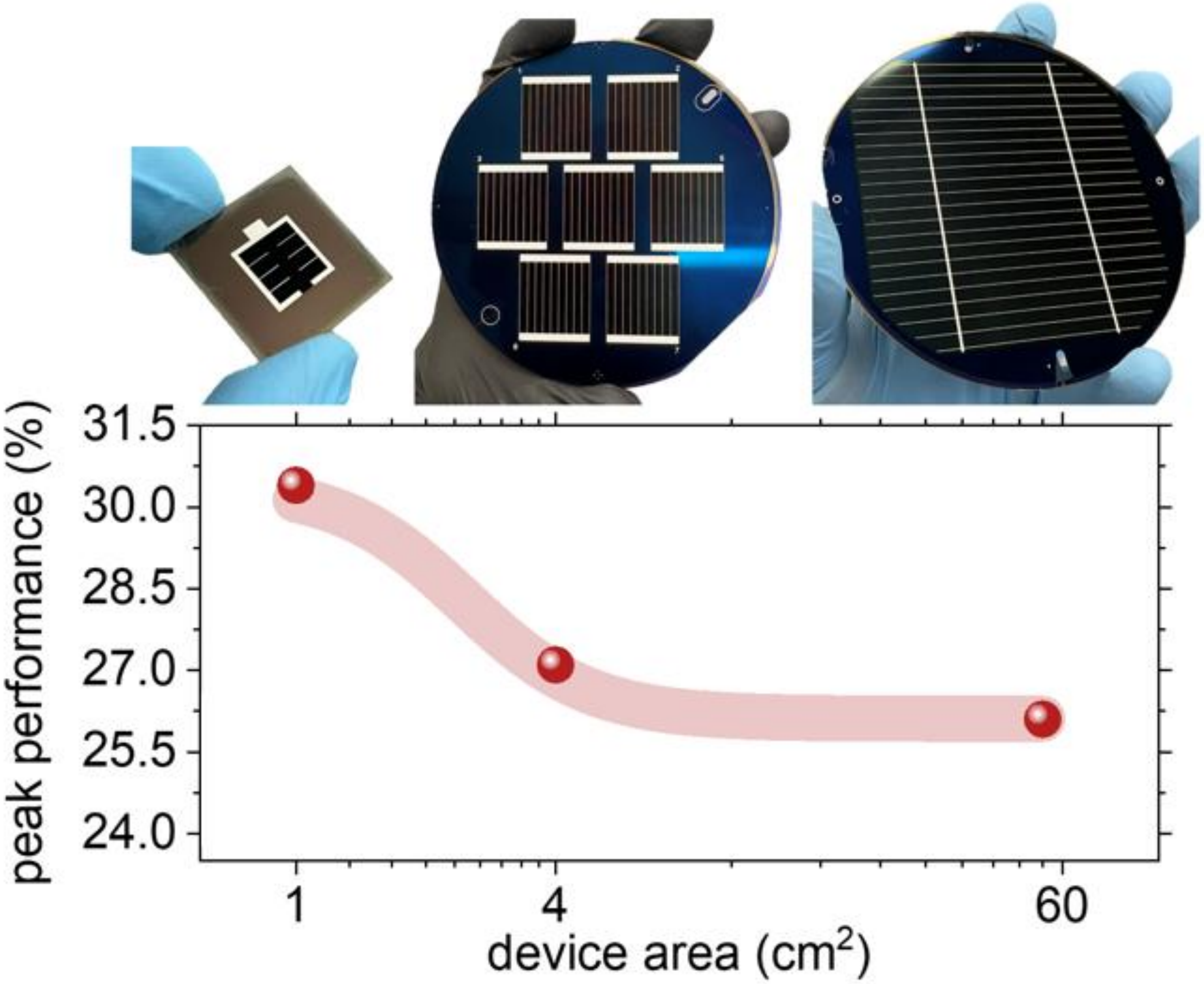

Figure S75. Peak reverse scan efficiency for 1 (certified from SIMIT), 4 and 54 cm^2 triple-junctions.

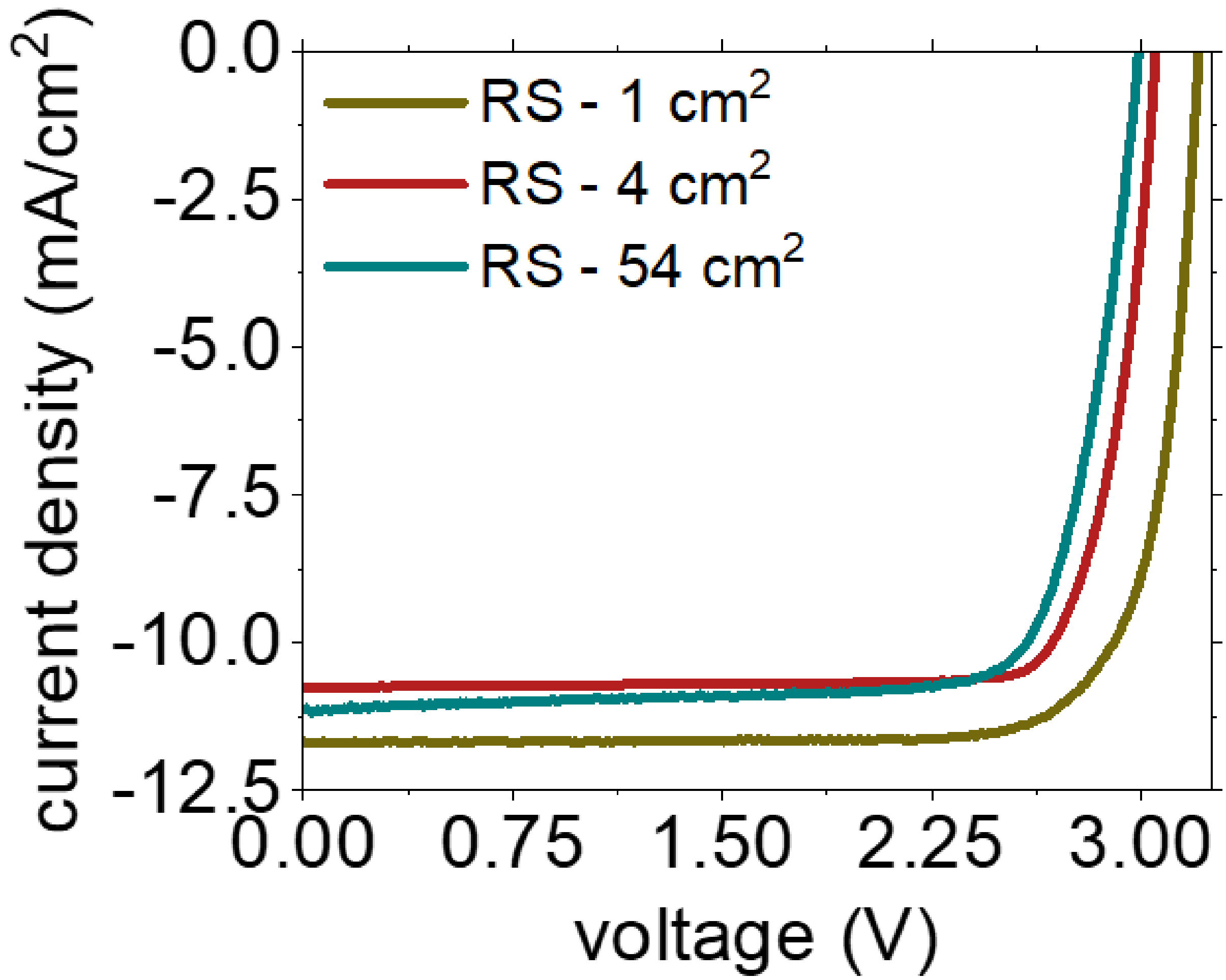

Figure S76. Champion reverse scan *JV* curves for devices with different active areas. The performance losses observed upon scaling to larger areas are discussed in detail in Supplementary Note 13.

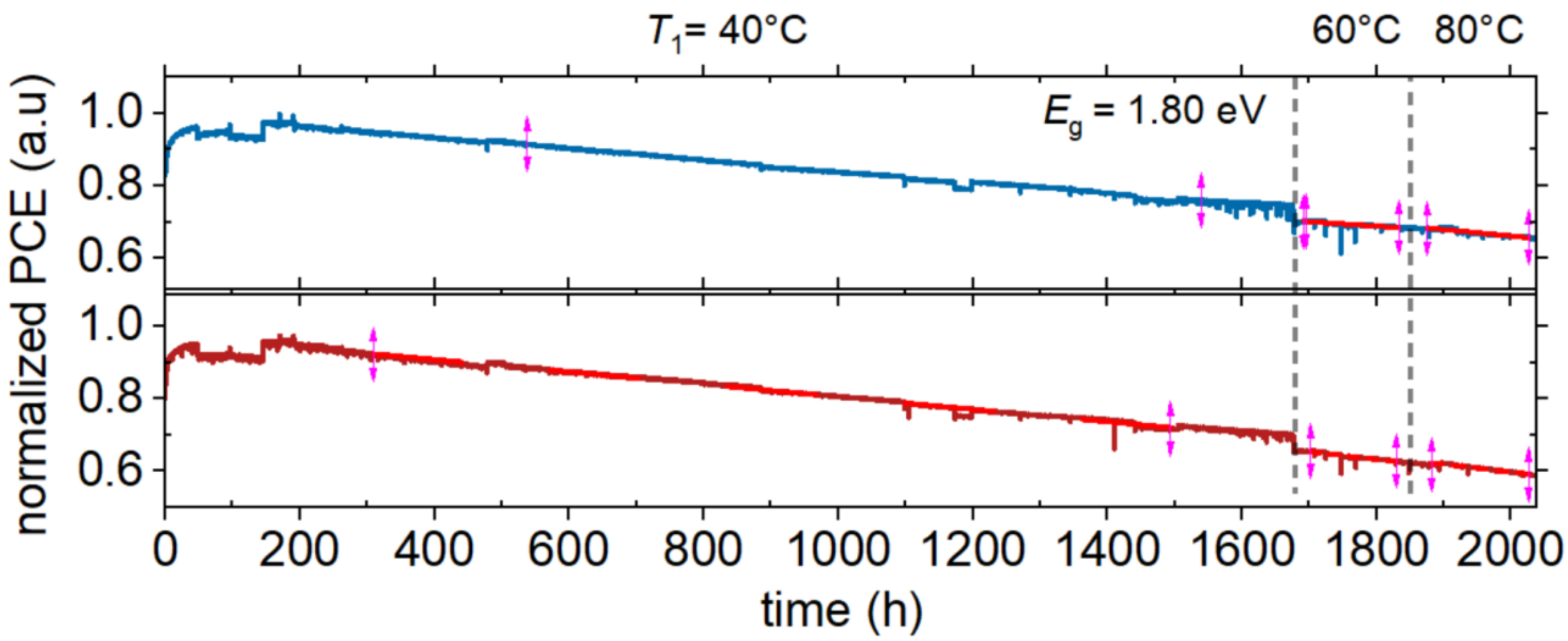

Figure S77. MPP tracking of single-junction devices with WBG absorber, control (blue) and 4-HBA (red). The fit regions to extract the degradation rate.

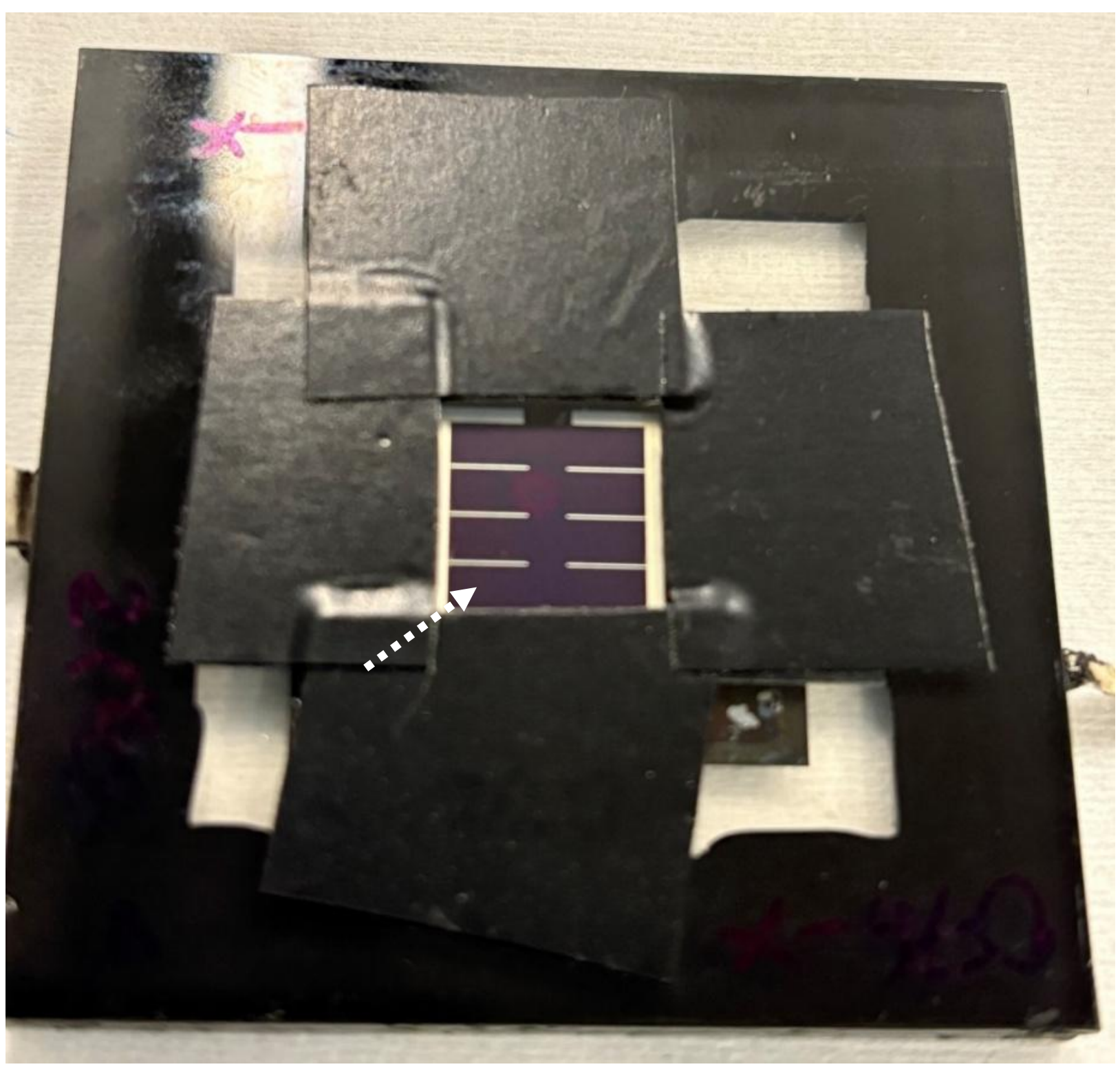

Figure S78. Image of the encapsulated 3J device after 500 h of DH testing that lost 22.9 $\%_{rel}$ PCE. In the middle of the sample, yellow point formation due to degradation is observed.

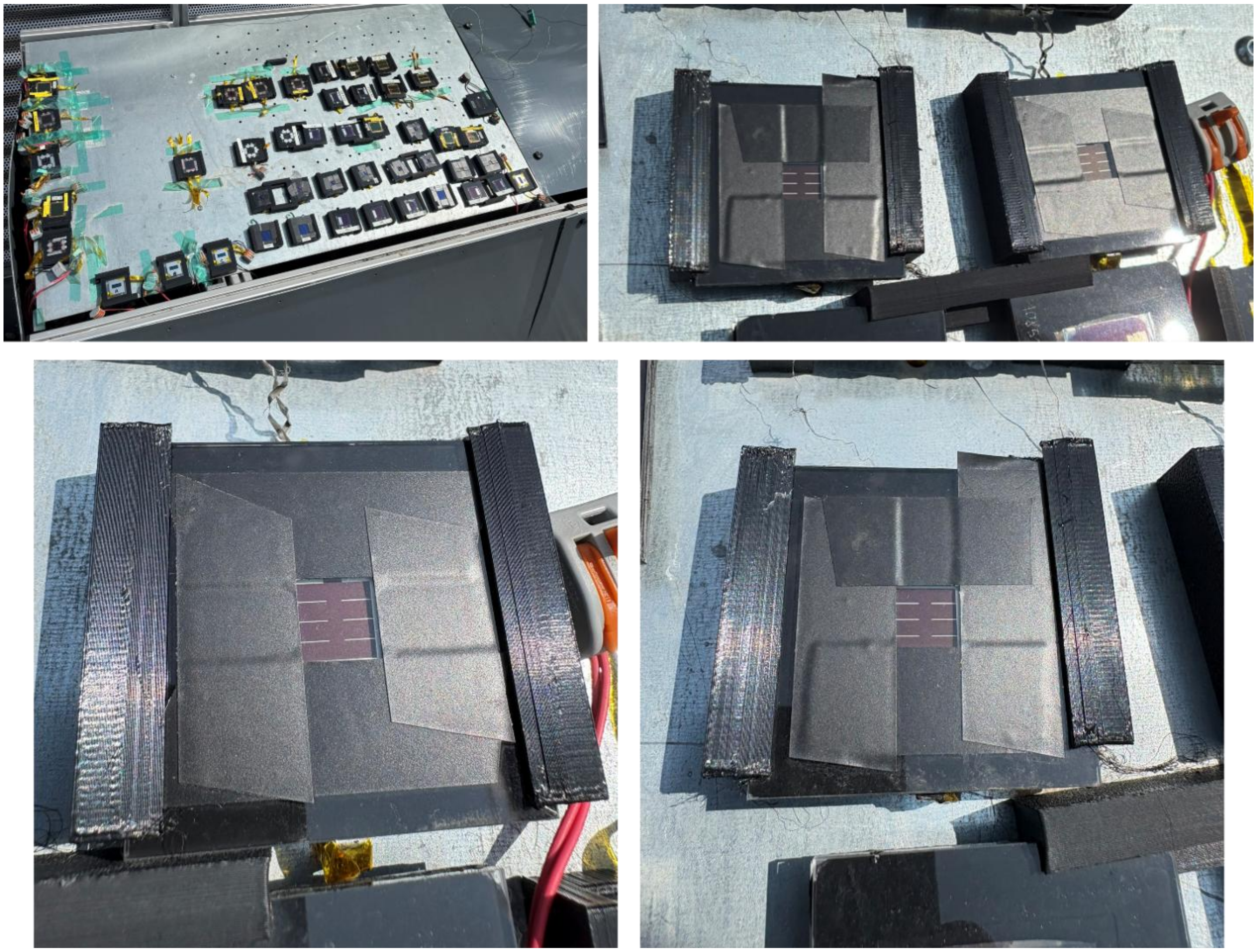

Figure S79. Images of the outdoor monitoring setup and tracked cells taken on the 6th of June, 2025. No visible degradation is observed in the 3J devices.

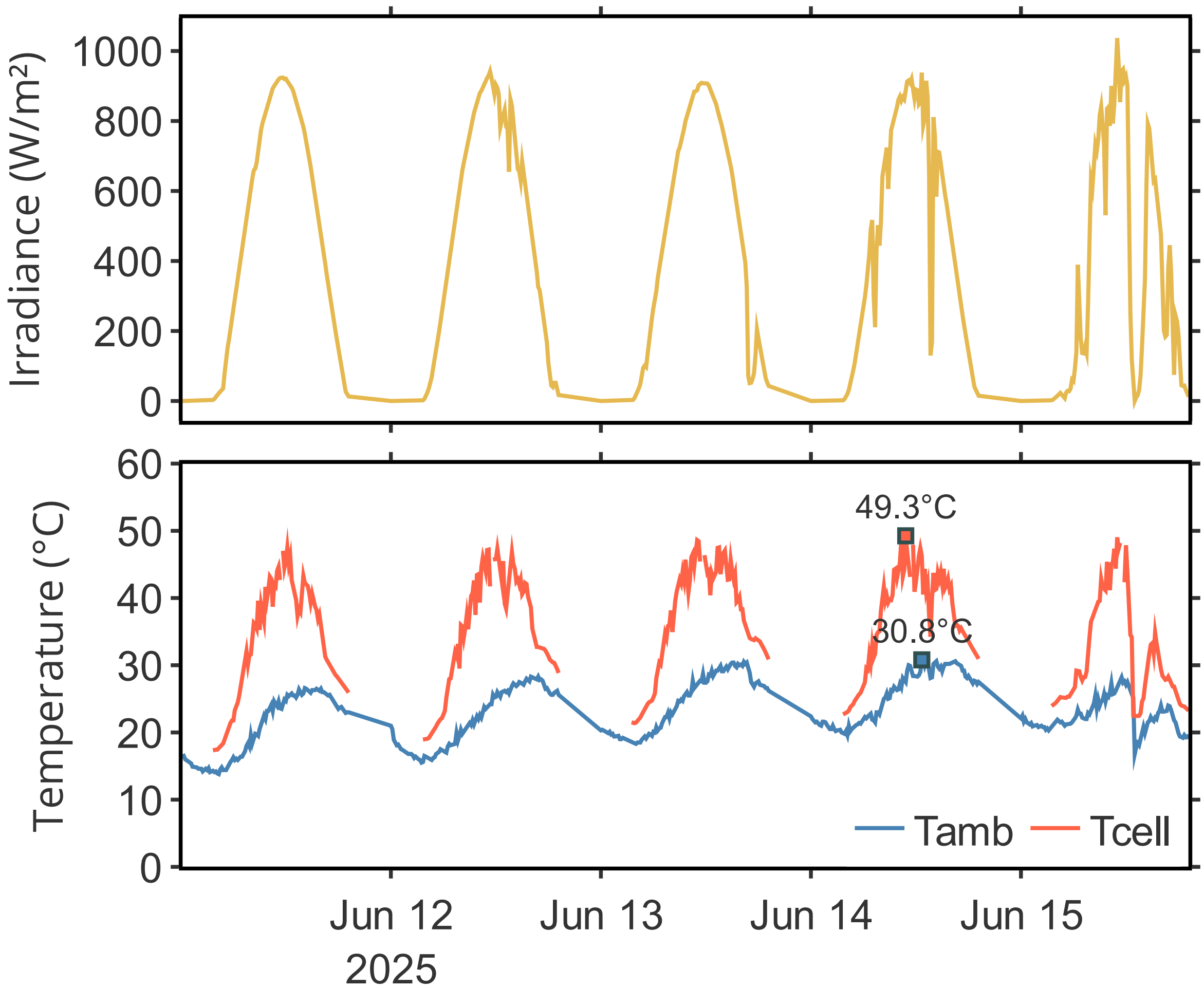

Figure S80. Measured cell temperature using a thermocouple behind a sister-cell and ambient temperature. Before 29 May, no data was recorded for the cell temperature due to technical difficulty. Cell temperature is ~19 °C higher than ambient temperature at peak irradiation.

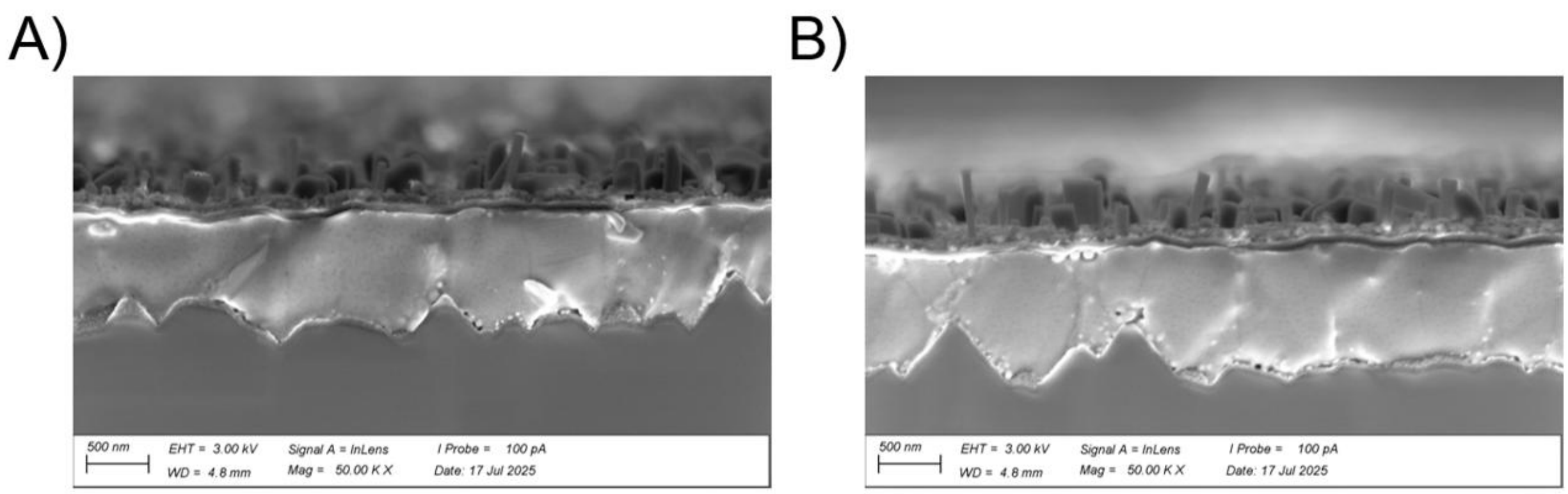

Figure S71. Perovskite–silicon tandem devices incorporating a NaF antireflection coating were processed during the summer (July, 2025), when relative humidity reached up to 60%. Owing to the hygroscopic ature of NaF, the coating microstructure deformed under these conditions, leading to the formation of vertically elongated, “skyscraper”-like features.

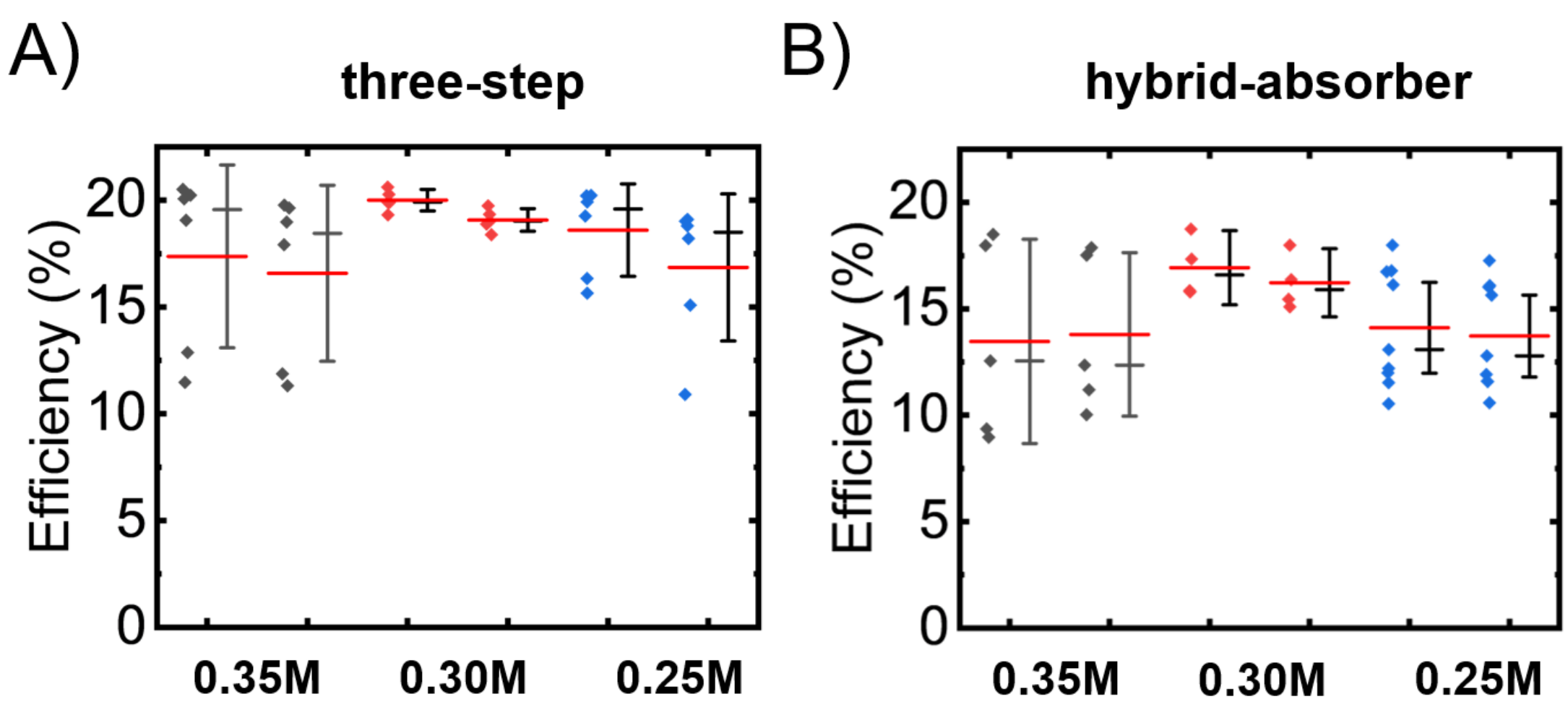

Figure S82. Comparison of organohalide concentration processing window for a) three-step and b) hybrid perovskite absorbers.

References

76. J. A. Steele, E. Solano, D. Hardy, D. Dayton, D. Ladd, K. White, P. Chen, J. Hou, H. Huang, R. A. Saha, L. Wang, F. Gao, J. Hofkens, M. B. J. Roeffaers, D. Chernyshov, M. F. Toney, How to GIWAXS: Grazing Incidence Wide Angle X-Ray Scattering Applied to Metal Halide Perovskite Thin Films. *Adv. Energy Mater.* **13** (2023).
77. P. Gratia, I. Zimmermann, P. Schouwink, J. H. Yum, J. N. Audinot, K. Sivula, T. Wirtz, M. K. Nazeeruddin, The Many Faces of Mixed Ion Perovskites: Unraveling and Understanding the Crystallization Process. *ACS Energy Lett.* **2**, 2686–2693 (2017).
78. S. Zeiske, O. J. Sandberg, N. Zarrabi, C. M. Wolff, M. Raoufi, F. Peña-Camargo, E. Gutierrez-Partida, P. Meredith, M. Stolterfoht, A. Armin, Static Disorder in Lead Halide Perovskites. *Journal of Physical Chemistry Letters* **13**, 7280–7285 (2022).
79. M. Othman, Q. Jeangros, D. A. Jacobs, M. H. Futscher, S. Zeiske, A. Armin, A. Jaffrès, A. G. Kuba, D. Chernyshov, S. Jenatsch, S. Züfle, B. Ruhstaller, S. Tabean, T. Wirtz, S. Eswara, J. Zhao, T. J. Savenije, C. Ballif, C. M. Wolff, A. Hessler-Wyser, Alleviating nanostructural phase impurities enhances the optoelectronic properties, device performance and stability of cesium-formamidinium metal-halide perovskites. *Energy Environ. Sci.* **17**, 3832–3847 (2024).
80. D. Turkay, K. Artuk, X.-Y. Chin, D. A. Jacobs, S.-J. Moon, A. Walter, M. Mensi, G. Andreatta, N. Blondiaux, H. Lai, F. Fu, M. Boccard, Q. Jeangros, C. M. Wolff, C. Ballif, Synergetic substrate and additive engineering for over 30%-efficient perovskite-Si tandem solar cells. *Joule*, doi: 10.1016/j.joule.2024.04.015 (2024).
81. C. Liu, Y. Yang, H. Chen, J. Xu, A. Liu, A. S. R Bati, H. Zhu, L. Grater, S. Sudhakar Hadke, C. Huang, V. K. Sangwan, T. Cai, D. Shin, L. X. Chen, M. C. Hersam, C. A. Mirkin, B. Chen, M. G. Kanatzidis, E. H. Sargent, "Bimolecularly passivated interface enables efficient and stable inverted perovskite solar cells;" https://www.science.org.
82. Z. Liu, H. Li, Z. Chu, R. Xia, J. Wen, Y. Mo, H. Zhu, H. Luo, X. Zheng, Z. Huang, X. Luo, B. Wang, X. Zhang, G. Yang, Z. Feng, Y. Chen, W. Kong, J. Gao, H. Tan, Reducing Perovskite/C60 Interface Losses via Sequential Interface Engineering for Efficient Perovskite/Silicon Tandem Solar Cell. *Advanced Materials* **36** (2024).
83. K. Artuk, A. Oranskaia, D. Turkay, F. Saenz, M. D. Mensi, M. De Bastiani, A. F. Castro-Méndez, J. Hurni, C. Allebé, M. Othman, L. Champault, A. G. Kuba, A. Levtchenko, D. A. Jacobs, J. B. Puel, D. Ory, F. Lang, A. Hessler-Wyser, U. Schwingenschlogl, Q. Jeangros, C. Ballif, C. M. Wolff, 60 cm2 perovskite-silicon tandem solar cells with an efficiency of 28.9% by homogeneous passivation. *Nature Communications* **16** (2025).
84. R. Azmi, D. S. Utomo, B. Vishal, S. Zhumagali, P. Dally, A. M. Risqi, A. Prasetio, E. Ugur, F. Cao, I. F. Imran, A. A. Said, A. R. Pininti, A. S. Subbiah, E. Aydin, C. Xiao, S. Il Seok, S. De Wolf, Double-side 2D/3D heterojunctions for inverted perovskite solar cells. *Nature* **628**, 93–98 (2024).
85. R. Lin, Y. Wang, Q. Lu, B. Tang, J. Li, H. Gao, Y. Gao, H. Li, C. Ding, J. Wen, P. Wu, C. Liu, S. Zhao, K. Xiao, Z. Liu, C. Ma, Y. Deng, L. Li, F. Fan, H. Tan, All-perovskite tandem solar cells with 3D/3D bilayer perovskite heterojunction. *Nature* **620**, 994–1000 (2023).
86. A. Ullah, B. Vishal, A. Razzaq, A. A. Said, S. Kralj, M. Morales-Masis, S. De Wolf, Improved Crystallinity and Efficiency in Perovskite Solar Cells through Layered Hybrid Deposition. *ACS Energy Lett.*, 30–33 (2024).
87. O. Er-raji, M. A. A. Mahmoud, O. Fischer, D. Bogachuk, B. P. Kore, O. Schultz-Wittmann, M. Bivour, M. Hermle, M. C. Schubert, J. Borchert, S. W. Glunz, P. S. C. Schulze, Tailoring perovskite crystallization and interfacial passivation in efficient, fully textured perovskite silicon tandem solar cells. *Joule*, doi: 10.1016/j.joule.2024.06.018 (2024).
88. Y. Yang, H. Chen, C. Liu, J. Xu, C. Huang, C. D. Malliakas, H. Wan, A. S. R Bati, Z. Wang, R. P. Reynolds, I. W. Gilley, S. Kitade, T. E. Wiggins, S. Zeiske, S. Suragtkhuu, M. Batmunkh, L. X.

Chen, B. Chen, M. G. Kanatzidis, E. H. Sargent, “Amidination of ligands for chemical and field-effect passivation stabilizes perovskite solar cells;” https://www.science.org.
89. C. Liu, Y. Yang, H. Chen, J. Xu, A. Liu, A. S. R Bati, H. Zhu, L. Grater, S. Sudhakar Hadke, C. Huang, V. K. Sangwan, T. Cai, D. Shin, L. X. Chen, M. C. Hersam, C. A. Mirkin, B. Chen, M. G. Kanatzidis, E. H. Sargent, “Bimolecularly passivated interface enables efficient and stable inverted perovskite solar cells;” https://www.science.org.
90. S. Liu, J. Li, W. Xiao, R. Chen, Z. Sun, Y. Zhang, X. Lei, S. Hu, M. Kober-Czerny, J. Wang, F. Ren, Q. Zhou, H. Raza, Y. Gao, Y. Ji, S. Li, H. Li, L. Qiu, W. Huang, Y. Zhao, B. Xu, Z. Liu, H. J. Snaith, N. G. Park, W. Chen, Buried interface molecular hybrid for inverted perovskite solar cells. *Nature* **632**, 536–542 (2024).
91. J. Thiesbrummel, V. M. Le Corre, F. Peña-Camargo, L. Perdigón-Toro, F. Lang, F. Yang, M. Grischek, E. Gutierrez-Partida, J. Warby, M. D. Farrar, S. Mahesh, P. Caprioglio, S. Albrecht, D. Neher, H. J. Snaith, M. Stolterfoht, Universal Current Losses in Perovskite Solar Cells Due to Mobile Ions. *Adv. Energy Mater.* **11** (2021).
92. J. Thiesbrummel, S. Shah, E. Gutierrez-Partida, F. Zu, F. Peña-Camargo, S. Zeiske, J. Diekmann, F. Ye, K. P. Peters, K. O. Brinkmann, P. Caprioglio, A. Dasgupta, S. Seo, F. A. Adeleye, J. Warby, Q. Jeangros, F. Lang, S. Zhang, S. Albrecht, T. Riedl, A. Armin, D. Neher, N. Koch, Y. Wu, V. M. Le Corre, H. Snaith, M. Stolterfoht, Ion-induced field screening as a dominant factor in perovskite solar cell operational stability. *Nat. Energy*, doi: 10.1038/s41560-024-01487-w (2024).
93. H. Hu, S. X. An, Y. Li, S. Orooji, R. Singh, F. Schackmar, F. Laufer, Q. Jin, T. Feeney, A. Diercks, F. Gota, S. Moghadamzadeh, T. Pan, M. Rienäcker, R. Peibst, B. A. Nejand, U. W. Paetzold, Triple-junction perovskite-perovskite-silicon solar cells with power conversion efficiency of 24.4%. *Energy Environ. Sci.* **17**, 2800–2814 (2024).
94. M. De Bastiani, G. Armaroli, R. Jalmood, L. Ferlauto, X. Li, R. Tao, G. T. Harrison, M. K. Eswaran, R. Azmi, M. Babics, A. S. Subbiah, E. Aydin, T. G. Allen, C. Combe, T. Cramer, D. Baran, U. Schwingenschlögl, G. Lubineau, D. Cavalcoli, S. De Wolf, Mechanical Reliability of Fullerene/Tin Oxide Interfaces in Monolithic Perovskite/Silicon Tandem Cells. *ACS Energy Lett.* **7**, 827–833 (2022).
95. K. Schutt, M. Davis, M. Li, S. A. Johnson, D. Martinez, J. Titus, T. Leijtens, B. Martin, M. D. McGehee, S. R. Marder, N. Rolston, J. M. Luther, Toward Fullerene-Free PIN Perovskite Solar Cells. *Anal. Chem.* **10**, 6307–6317 (2025).
96. M. R. S. Poma, Y. Zhang, M. Li, K. Mao, R. A. DeCrescent, S. Barlow, N. Rolston, S. R. Marder, M. D. McGehee, Incorporating a naphthalene diimide polymer into a fullerene electron-transport layer to improve the fracture energy of perovskite solar cells. *EES Solar*, doi: 10.1039/D5EL00194C (2026).
97. H. Wang, Z. Wang, Z. Qu, Z. Zhang, K. Meng, G. Chen, T. J. Jacobsson, J. You, J. Luo, Spatial PbI2 distribution impacting stability of perovskite solar cells. *Journal of Energy Chemistry* **105**, 446–453 (2025).
98. G. Tumen-Ulzii, C. Qin, D. Klotz, M. R. Leyden, P. Wang, M. Auffray, T. Fujihara, T. Matsushima, J. W. Lee, S. J. Lee, Y. Yang, C. Adachi, Detrimental Effect of Unreacted PbI2 on the Long-Term Stability of Perovskite Solar Cells. *Advanced Materials* **32**, 1–7 (2020).
99. Jiang Liu, Yongcai He, Lei Ding *et al.* Perovskite/silicon tandem solar cells with bilayer interface passivation. *Nature* **635**, 596–603 (2024).
100. G. Makov, M. C. Payne, Periodic boundary conditions in *ab initio* calculations. *Phys. Rev. B* **51**, 4014–4022 (1995).
101. K. Momma, F. Izumi, *VESTA 3* for three-dimensional visualization of crystal, volumetric and morphology data. *J. Appl. Crystallogr.* **44**, 1272–1276 (2011).
102. A. Musiienko, F. Yang, T. W. Gries, C. Frasca, D. Friedrich, A. Al-Ashouri, E. Sağlamkaya, F. Lang, D. Kojda, Y. T. Huang, V. Stacchini, R. L. Z. Hoye, M. Ahmadi, A. Kanak, A. Abate, Resolving electron and hole transport properties in semiconductor materials by constant light-induced magneto transport. *Nat. Commun.* **15** (2024).

103. J. Metzdorf, “Calibration of solar cells. responsivity method” (1987); https://doi.org/10.1364/OA_License_v1#VOR.
104. M. Meusel, R. Adelhelm, F. Dimroth, A. W. Bett, W. Warta, Spectral mismatch correction and spectrometric characterization of monolithic III-V multi-junction solar cells. *Progress in Photovoltaics: Research and Applications* **10**, 243–255 (2002).
105. M. Heydarian, A. J. Bett, C. Messmer, J. Aulich, O. Fischer, M. Heydarian, Y. Gupta, P. S. C. Schulze, J. Borchert, F. Schindler, M. C. Schubert, S. W. Glunz, Impact of Perovskite Subcell Breakdown on the Performance of Perovskite/Perovskite/Silicon Triple-Junction Solar Cells. *Solar RRL* **8** (2024).
106. D. Chojniak, M. Schachtner, S. K. Reichmuth, A. J. Bett, M. Rauer, J. Hohl-Ebinger, A. Schmid, G. Siefer, S. W. Glunz, A precise method for the spectral adjustment of LED and multi-light source solar simulators. *Progress in Photovoltaics: Research and Applications* **32**, 372–389 (2024).
