# Supplementary material for: Triple-junction solar cells with improved carrier and photon management
Source: Nature. 2026 Mar 17;653(8113):90–7. doi: 10.1038/s41586-026-10385-y (PMC13658016; doi:10.1038/s41586-026-10385-y)
Supplement: Supplementary file 2 [file 41586_2026_10385_MOESM2_ESM.pdf]

nature portfolio

Peer Review File

Triple-junction solar cells with improved carrier and photon management

Corresponding Author: Dr Kerem Artuk

Any redactions in this file are there to maintain patient confidentiality, the confidentiality of unpublished data, or to remove third-party material.

This file contains all reviewer reports in order by version, followed by all author rebuttals in order by version.

Attachments originally included by the reviewers as part of their assessment can be found at the end of this file.

Version 0:

Reviewer comments:

Referee #2

(Remarks to the Author)
This manuscript addresses two primary limitations in perovskite–silicon triple-junction solar cells: (i) the low open-circuit voltage of the wide-bandgap top cell and (ii) the current density constraint of the middle-bandgap subcell. The authors employ additive engineering to regulate crystallization kinetics and reduce bulk recombination in wide-bandgap perovskites, implement a three-step deposition method to fabricate thicker middle-bandgap absorbers, and utilize SiO_x nanoparticle-based back reflection to enhance photocurrent. As a result, the authors claim that the device achieves a steady-state efficiency of 30.5%. While this efficiency seems to exceed the previous certified record of 27.6% (Nature, 628, 306–312, 2024), the manuscript does not provide any formal certification reports—precluding apple-to-apple comparison.

In addition, the scientific findings primarily consolidates well-established knowledge (such as additive-assisted crystallization, absorber thickness optimization, and nanoparticle-mediated light management) rather than introducing any new concepts. Too many perovskite papers simply use some additives (whether random or well-known), achieve high efficiency, and then make up a story to get published. This is not how the field works, especially for an extremely high-impact journal like Nature. As the most authoritative scientific journal, Nature has the commitment to publishing work that not only reports the record efficiency but also focuses more on scientific concepts capable of inspiring and advancing the perovskite field.

Thus, the authors should seriously and clearly articulate how their work advances beyond existing studies and emphasize its unique scientific contribution. Major concerns are as follows:

1. Additive engineering is a well-established approach for controlling crystallization kinetics and enhancing film quality. Recent work by De Wolf et al. (Nature, 628, 93–98, 2024) demonstrated the efficacy of 4-hydroxybenzylamine in this context, which is EXACTLY the SAME in this work. Compared to previous studies, this manuscript offers no new insights into additive selection or mechanistic understanding. The authors are encouraged to provide original perspectives on the rationale for material selection and nucleation–crystallization dynamics to strengthen its academic impact. It is very important the authors need to conduct an in-depth investigation into whether there is an universal scientific principle for this additive that other researchers can learn from regarding how this additive affects wide-bandgap perovskites.

2. In wide-bandgap perovskites ($E_g > 1.9$ eV) used as top cells in triple-junction configurations, halide phase segregation remains a significant challenge. The authors apply conventional additive engineering to regulate crystallization and chemical coordination to passivate defects, thereby reducing non-radiative recombination and improving open-circuit voltage. However, these techniques and related scientific questions are extensively reported. The manuscript lacks an insight into the mechanism for suppressing phase segregation or a generalizable strategy to address this issue, limiting its contribution to advancing the field.

3. Increasing absorber thickness to enhance light harvesting via three-step deposition is a common method. For instance,

Tan et al. and Li et al. (Nature, 620, 994–1000, 2023; eScience, 100449, 2025) reported similar methods with notable efficiency gains. Given the similarities, thickness optimization alone does not establish novelty. The authors should explore distinctive effects on interface evolution, phase stability, or carrier dynamics to differentiate their work from existing literature.

4. The use of SiO_x nanospheres to enhance light harvesting on textured silicon surfaces was recently reported (Nature Communications, 16, 7331, 2025). However, the insulating nature of SiO_x raises concerns about the trade-off between improved optical reflection and potential reductions in electrical contact or transmittance, which could compromise the overall optical design. The manuscript should clarify how this strategy integrates into the device's optoelectronic framework and provide data on these trade-offs, beyond focusing solely on photocurrent enhancement.

5. Although efficiency measurements were conducted by Fraunhofer ISE and EPFL, the current manuscripts lack formal certification reports from authoritative institutions. In high-impact journals, key efficiency claims typically include such certification (e.g., Nature, 635, 596–603, 2024; Nature, 623, 313–318, 2023). It is COMPULSORY to include FORMAL certified reports to ensure the reliability and global credibility of their findings.

6.This manuscript still lacks an important result regarding device stability, namely the operational stability of triple-junction tandem solar cell devices, which should be evaluated through maximum power point tracking, rather than only focusing on the operational stability of WBG solar cells.

Some other technical issues also require attention, for example:

7.The rigor of the insitu GIWAXS analysis presented in Figure 1c during the annealing of wide-bandgap perovskites is questionable. According to established literature (Nature Energy, 9, 536-547, 2024; Nature, 624, 557-563, 2023), the characteristic q-value for the 3C cubic perovskite phase is approximately 1.0 nm^{-1}, while those for the 2H, 4H, and 6H phases are approximately 8.6 nm^{-1}, 8.4 nm^{-1}, and 9.3 nm^{-1}, respectively. However, the q-values assigned to these phases in the manuscript deviate significantly from these benchmarks. More critically, the formation of the 3C phase was not observed in the experiments. The authors should provide a reasonable explanation for these discrepancies.

8.In Figure 2D, the current density of single-junction MBG devices is consistently below 24 mA/cm^2, regardless of whether a one-step or three-step method is used. This value is strikingly low compared to the field benchmarks: for 1.52 eV perovskite materials, current densities routinely approach 26 mA/cm^2, with averages exceeding 25 mA/cm^2 (refs: Nature, 632, 301-306 (2024); Nature, 632, 536-542 (2024); Nat Commun 16, 753 (2025); Adv. Mater., 37, 2505087 (2025)). The authors must provide a detailed explanation for this significant underperformance relative to established standards.

9.In Figure 2H, the increased reflection from the SiOx-np layer in the Perovskite/SiOx-np/Si structure is likely to be reabsorbed by the thick middle-bandgap perovskite layer. Therefore, comparing the reflectance spectra of Perovskite/SiOx-np/Si with that of Perovskite/Si is inaccurate. It would be more convincing to directly compare the reflectance spectra of SiOx-np/Si and bare Si.

In general, this manuscript could be reconsidered if the following two things are further conducted: 1) Efficiency is certified by a third-party authority; 2) The scientific findings are reorganized to provide in-depth insights into why this work holds significant scientific importance for triple-junction perovskite/silicon tandem solar cells.

Referee #3

(Remarks to the Author)
I co-reviewed this manuscript with one of the reviewers who provided the listed reports.

Referee #4

(Remarks to the Author)
The manuscript entitled "Perovskite-perovskite-silicon triple junction solar cells with improved carrier and photon management," with Ref #: "2025-07-17350", by authors "Artuk et al." is a research article on the demonstration of a monolithic (series-connected) perovskite-perovskite-silicon triple-junction solar cell. The authors address two key challenges, one which is the shortcomings of the open-circuit voltage (Voc) of the wide-bandgap top-cell and the other is the limited current density (due to absorber thickness) in the middle-cell. For tackling the top-cell, the authors use additive engineering via 4-hydroxybenzylamine (4-HBA), an organic compound with the molecular formula C_7H_9NO. They show that 4-HBA improves the perovskite crystallization dynamics and suppresses bulk recombination, which helped achieve impressive open-circuit voltages up to 1.405 V. The authors use many high-level characterization techniques to confirm the benefit of the additive 4-HBA, such as in-situ grazing incidence wide-angle x-ray diffraction (GIWAX), X-ray photoelectron spectroscopy (XPS), Time-of-flight secondary ion mass spectrometry (ToF-SIMs), high-dynamic-range external quantum efficiency (HDR EQE), and more. For the middle-cell, they developed a somewhat complex three-step deposition process in order to achieve a substantially "thick" perovskite absorber based roughly on the Cs0.05FA0.9MA0.05PbI3 formulation. The method to achieve the increased thickness required (1) standard one-step spin-coated antisolvent deposition, (2) thermal co-evaporation of the inorganic halide template of PbI2 and CsI, and (3) final spin-coating of FAI:MAI in isopropanol (including annealing steps). A third innovation that the authors demonstrate is the filling of the textured pyramid valleys with reflective SiOx (given the sharp change in refractive index), which helps with the insufficient absorption in the middle-cell. This idea was used back in µc/a-Si tandem and triple-junction solar cells many years ago to enhance top and middle-cell current density.

The fabrication of perovskite–perovskite–silicon triple-junction solar cells is an exceptionally challenging endeavour, demanding not only a high-level of technical complexity in terms of materials processing and device integration, but also substantial investment of time, resources, and experimental precision to achieve reproducible and high-performance devices. The authors produce an impressive record efficiency for the perovskite-perovskite-silicon triple-junction solar cell of 30.5%. The current record is actually 28.7% published only recently by Xu et al. in Nature Materials on the 23 September 2025 (see Xu et al., Stabilized perovskite phases enabling efficient perovskite/perovskite/silicon triple-junction solar cells. Nat. Mater., 1–8, 2025. doi: 10.1038/s41563-025-02367-8). The device demonstrated by the authors here shows a notable efficiency improvement of 1.8%, achieved by effectively addressing key challenges through the three innovations outlined above. These key innovations would be very welcome and of great interest and timely to the perovskite-based multi-junction solar cell field, which has seen a surge in popularity given the potential for industrial mass-scale commercialization.

Overall, the manuscript is suitable for publication in “Nature”, after some key revisions. Please find the detailed review below:

Main Concerns:

1. One of the key concerns is with section on “Hybrid on one-step (three-step) absorbers”. The authors have not made it clear why they could not create a thicker middle-cell absorber with simply the one-step antisolvent or gas-quenching techniques and why they have to resort to such a complex three-step deposition process? The authors simply mention in Supplementary Note 1, that “Initial attempts to increase the absorber thickness by adjusting the solution molarity and spin-coating parameters resulted in delamination of the absorber upon annealing, as shown in Fig. S22. This issue arose due to increased mechanical stress and weak adhesion between the perovskite and the hydrophobic Me-4PACz hole transport layer (22).” The authors should first provide some context and discussion as to why thick perovskite films >1µm, such as $FAPbI_3$, cannot simply be deposited by a one-step process. Is there a primarily limitation with the deposition of thicker perovskite films using one-step techniques? For example, is it because of solubility limitations or is it because of issues with crystallisation, film morphology or phase issues? Are there any relevant examples of this in the literature?

2. It is opportune that the three-step technique the authors propose, with the co-evaporation followed by sequential solution processing of the FAI:MAI in isopropanol do not remove the underlying one-step deposited perovskite. However, there are still a few questions to be asked here. Especially if this is the first time this three-step technique has been demonstrated by the authors. For example, what does the homogeneity of the middle-cell perovskite look like? Some cross-sectional SEM with EDX/EDS and/or ToF-SIMs analysis across this layer would be valuable here (single-junctions or simply layers on substrates is sufficient). Does the three-step process end up producing some sort of graded bandgap perovskite absorber (see Ergen et al., Graded bandgap perovskite solar cells. Nat. Mater., 16, 522–525, 2017. doi: 10.1038/nmat4795)? Furthermore, could there be a limitation with the charge extraction given limited carrier diffusion lengths in the actual perovskite absorber layer going to thicker films?

3. The authors should provide some discussion (including references) with the use of sub-micron textured pyramids. There have been many publications in the literature for the use of such the sub-micron textured pyramids to be compatible with solution-processed perovskites. It would seem highly appropriate here given that current-matching due to the limitations of the middle-cell thickness is a key aspect in this work. How does the sub-micron textured pyramid heights and the way it reflects/absorbs the incoming light differ to that for standard industrial heterojunction silicon solar cells with ~5 µm features (for example, see Chen et al., Blade-Coated Perovskites on Textured Silicon for 26%-Efficient Monolithic Perovskite/Silicon Tandem Solar Cells. Joule, 4(4), 850–864, 2020. doi: 10.1016/j.joule.2020.01.008 and Li et al., Nanoscale Size Control of Si Pyramid Texture for Perovskite/Si Tandem Solar Cells Enabling Solution-Based Perovskite Top-Cell Fabrication and Improved Si Bottom-Cell Response. Adv. Mater. Interfaces, 10(35), 2300504, 2023. doi: 10.1002/admi.202300504)?

Other concerns and corrections:

Page 2, Line 62: It is unclear when the authors state: "Perovskite-silicon tandem solar cells have surpassed III-V tandem technology, achieving 34.8% efficiency, approaching the theoretical limit of 42.2% (1, 2)." Is not the record for III-V on silicon 36.1% (triple-junction) and the record for perovskite-silicon 34.85% (dual-junction)? It would be clearer to make the claim that this is for "dual-junction" and find the correct reference here, it seems to be the GaAsP/Si record from OSU/UNSW/SolAero of 23.4% from 2020. Perhaps it would make more sense to simply state that this is the "dual-junction record" with no need to list the 23.4% (III-V researchers probably do not target dual-junction III-V on Si as it is not particularly attractive to do so given efficiencies are higher for triple-junctions.

Page 2, Line 71: There is actually a recent newly published efficiency record for triple-junction perovskite-perovskite-silicon solar cells (see Xu et al. Stabilized perovskite phases enabling efficient perovskite/perovskite/silicon triple-junction solar cells. Nat. Mater., 1–8, 2025. doi: 10.1038/s41563-025-02367-8).

Page 2, Line 80: For context, the authors could also mention some other ways the field has tried to try to reduce Voc losses in wide-bandgap perovskites especially for perovskite-based multi-junction solar cells. For example, use of Rb^+ (Wang et al. Suppressed phase segregation for triple-junction perovskite solar cells. Nature, 618, 74–79, 2023. doi: 10.1038/s41586-023-06006-7 and Zheng et el. Strain-induced rubidium incorporation into wide-bandgap perovskites reduces photovoltage loss. Science, 388(6742), 88–95, 2025. doi: 10.1126/science.adt3417), propane-1,3-diammonium iodide (Wang et al. Halide homogenization for low energy loss in 2-eV-bandgap perovskites and increased efficiency in all-perovskite triple-junction solar cells. Nat. Energy, 9, 70–80, 2024. doi: 10.1038/s41560-023-01406-5), or triple-cation triple halide (Heydarian et al.

Minimizing Open-Circuit Voltage Losses in Perovskite/Perovskite/Silicon Triple-Junction Solar Cell with Optimized Top Cell. Solar RRL, 9(3), 2400645, 2025. doi: 10.1002/solr.202400645).

Page 2, Line 89: "Moreover, a middle-reflector composed of low refractive index SiOx-nanoparticles (SiOx-np) accumulating in the valleys of submicron-textured silicon between the middle- and bottom-cell further enhances the middle-cell current density." Did the authors examine the interface between the SiOx and the perovskite with SAM in between? For example, does the interface show or demonstrate an improvement or loss to the ITO in terms of quasi-fermi level splitting (QFLS) analysis?

Page 3, Line 120: The term 'stereoisomers' may not be entirely accurate in this context. 2-HBA, 3-HBA, and 4-HBA are positional (constitutional) isomers, differing by the position of the hydroxyl group on the aromatic ring. Strictly speaking, stereoisomers of hydroxybenzylamine would only arise if chirality were introduced at the benzylic carbon or under particular structural constraints. Perhaps the authors might consider clarifying this wording to avoid potential confusion.

Pg, 3, Line 122: "…stereoisomers, 4-HBA provided the highest passivation quality, reducing non-radiative losses to ~ 130 meV." Chemically, was there a reason why this is the case between 2-, 3-, and 4-HBA?

Page 6, Line 230. Have the author quantified the benefit of having reduced surface area between the perovskite and hole transport layer by using the SiOx. Is significant reduction in surface recombination seen as a result? Have the authors reasonably quantified this?

Page 6, Line 238. The authors state, "without affecting Jsc". However, from Figure S21, it looks as though the Jsc decreases slightly with the BMP? Is that simply a smaller spread in the samples that were tested? Would "without drastically affecting Jsc" be more suitable here?

Figure 2A) caption: "... reverse scan JV curves..." otherwise "... reverse scan JV curve of the wide-bandgap..."

Figure 2C) and Figure 2F) Scale bar values could be shown directly onto the SEM image for better clarity.

Page 7, Line 248. The order of the references should be Randi et al. then Lin et al. (17, 31)

Page 7, Line 266. Weak or 'forbidden' reflections in FA-based perovskites have been shown to originate from subtle octahedral tilting, a structural distortion that is easily overlooked in conventional XRD but crucially suppresses the transition to hexagonal 4H/6H polytypes. Doherty et al. provided clear evidence of this tilt-stabilized noncubic phase, directly linking such faint peaks to enhanced phase stability (see Doherty et al. Stabilized tilted-octahedra halide perovskites inhibit local formation of performance-limiting phases. Science, 374(6575), 1598–1605, 2021. doi: 10.1126/science.abl4890).

Page 9, Figure 3C). Why have the authors opted to use NaF as the front anti-reflection layer as opposed to the more common MgF2 or LiF?

Page 9, Figure 3F). Integrated Jsc values for each of the top, middle, and bottom-cells are required here. Nearly all publications in perovskite-based multi-junction solar cells show these values.

Page 10, Line 356. The authors mention that Si bottom-cell performs on average rather poorly (iVOC = 0.679V) with detrimental low PL areas. Could the authors provide some PV parameters for stand-alone single-junction silicon heterojunction bottom cells that were used to make the triple-junction devices in this work? Perhaps as an additional figure or table in the Supplementary materials? At the very least provide a reference(s) to silicon hetero-junction solar cells made in the past by the same research institute. Higher Voc can be expected not only from reduced processing steps and handling challenges highlighted by the authors, but also from the increased light capture that results when the two additional middle- and top-cell perovskite absorbers are absent in single-junctions. Having single-junction silicon heterojunction solar cell data used in this work would provide a strong basis for comparison and understanding of upper device efficiency limits.

Supplementary Information, page 3: "…SiOx-np solution in Ethanol (for WBG) and 0.1% SiOx-np for MBG in EtOH (20 nm diameter)…" Is Ethanol not the same as EtOH?

Supplementary Information, page 3: "For the BMP in WBG, 0.4 mg/mL…," this should read "For the bimolecular passivation (BMP) in WBG, 0.4 mg/mL…"

Supplementary Information, page 4: On the description of the double-side nanotextured devices, are both the front-side and rear-side the same texturing process? That is, are they both the same sub-micron textured pyramids? If so, why did the authors not consider the standard texturing process (~5 µm) for the rear side and sub-micron texturing for the front side of the silicon heterojunction solar cell? For example, see Jia et al. Efficient perovskite/silicon tandem with asymmetric self-assembly molecule. Nature, 644, 912–919, 2025. doi: 10.1038/s41586-025-09333-z.

Supplementary Information, page 4: "…is deposited at 200°C (45. …" ???

Supplementary Information, page 5: What does CsFA-TH stand for? Could the authors please provide the full term in the unabbreviated form?

Supplementary Information, page 5: "…coordinated via three shared I-bonds)." Seems to be a hanging ")" there.

Supplementary Information, page 6: "1.000 W/m2" to "1,000 W/m2"

Supplementary Information, page 6: Isn't ~0.50 V/s very fast for a typical scan rate? Why have the author chosen this scan rate? Later on in the paragraph, the scan rate for the ~1 cm2 is 0.1 V/s. Why the variation here?

Supplementary Information, page 6: "Pv-tools" should be "PV-tools"

Supplementary Information, page 7: "… June 3, 2025. sGlobal horizontal…" Please correct.

Supplementary Information, page 9: "linkam" should be capitalized, "Linkam".

Supplementary Information, page 21: For Figure S13, is there a colour or symbol differentiation between the C-O and O-H?

Supplementary Information, page 25. For Figure S18-S19, it is not clear what the difference is between the samples simply with reference to the names 32R, 72R, 42HBA, and 92HBA. There does not seem to be an explanation anywhere in the text.

Overall, this manuscript presents high-quality research that makes a timely and substantial contribution to the rapidly advancing field of perovskite-based multi-junction solar cells. The authors report a record-efficiency perovskite–perovskite–silicon triple-junction device by addressing wide-bandgap Voc losses, middle-cell thickness limitations, and optical management through filling of SiOx in textured interfaces. The work demonstrates both originality and technical innovation, and its findings would be of interest to the readership of Nature.

Regarding certification, while independent testing is normally expected for record devices, the authors reasonably justify its absence. They note that two leading institutes could not certify triple-junctions and a third had a long delay, and instead worked with Fraunhofer ISE to validate results against EPFL using an LED-based simulator. This provides a credible basis for confidence in the reported metrics.

Some aspects, however, need further strengthening before publication. The three-step deposition method for the middle-cell absorber should be studied more carefully through cross-sectional characterization (e.g., SEM with EDX/EDS or ToF-SIMS) to confirm uniformity in thick perovskite films. Further discussion is also needed on the limitations of one-step deposition for >1 µm absorbers and on the role of sub-micron nano-textures compared to standard micron-scale silicon texturing, to place the work more firmly within the broader literature.

Version 1:

Reviewer comments:

Referee #2

(Remarks to the Author)
The authors have provided a comprehensive response to the previous comments and have supplemented the study with independent certification reports from SIMIT. However, as previously noted in comments #1, #3, and #4, the use of the 4-hydroxybenzylamine additive, the three-step deposition strategy, and the SiOx nanoparticle reflector are well-documented in the existing literature. Therefore, referring to these as 'innovations' in the manuscript abstract is inaccurate. It appears that the current work is largely a composite of established methods; while the improvement in efficiency for the perovskite-perovskite-silicon triple-junction solar cells is notable, the revised manuscript still lacks new scientific insights. Frankly, the work reads more like a technological process report than a scientific study. We reiterate the view that 'too many perovskite papers simply use some additives (whether random or well-known), achieve high efficiency, and then construct a narrative to get published.' Given the lack of novelty and the data issues, we do not recommend the acceptance of this manuscript.
Major concerns are as follows:
1. Regarding the responses to comments 1–4#, I must emphasize that although the revised manuscript shows minor differences from prior literature—concerning the additive, the three-step perovskite deposition method, and the SiOx nanoparticle reflector—such marginal variations fail to establish the novelty and scientific significance required for work in the field of triple-junction solar cells.
2. There is a clear logical contradiction regarding the scientific problem statement in the revised manuscript. The authors state in the Introduction that 'wide-bandgap (WBG, >1.80 eV) perovskite absorbers suffer from elevated non-radiative losses and require high bromide content, which leads to phase segregation, reducing stability, and increasing non-radiative recombination…' Subsequently, however, they claim that 'In perovskite-based, additive and compositional engineering have been demonstrated to address this (former) challenge.'
If additive engineering has already resolved these challenges, what is the significance of the extensive study on the 4-HBA additive in this work? Furthermore, where is the scientific innovation? These self-contradictory statements are one of the primary reasons I consider the novelty of this manuscript to be insufficient."
3. Regarding the analysis of the 4-HBA additive engineering, although the authors attempted in their response to distinguish bulk doping from previously reported surface-phase reconstruction strategies, their exposition of the core passivation mechanism remains confined to the chemical coordination between the amino group and Pb^{2+}. The authors also acknowledge similarities in the mechanistic discussion of chemical passivation with that the previously reported work by De

Wolf et al. This study has not sufficiently demonstrated the unique advantages of introducing molecules into the bulk phase. The innovation is thus confined to the adjustment of “Pb passivation at different sites” rather than representing a breakthrough in understanding the passivation mechanism.

4. The application of SiOx nanoparticles on textured silicon substrates has been reported on multiple occasions (Joule, 8, 1735-1753, 2024; Nature Communications, 16, 7331, 2025). Specifically, the Nature Communications study explicitly elucidated the utilization of nanoparticles for optical anti-reflection and light management, which significantly overlaps with the mechanism of action of SiOx in this study. In the revised manuscript, the authors continue to focus primarily on anti-reflection properties, failing to provide novel scientific insights beyond existing understanding.

5. The artificial compilation of key data in the revised manuscript is unacceptable for a journal of Nature’s standing. For example, the triple-junction device relies on a 50% Br content (1.9 eV), while the single-junction analysis is limited to 30% Br (1.8 eV). Because the compositions are not identical, the challenges faced by these two bandgaps are not on the same level! It is clear that the authors are cherry-picking data to fit a predetermined narrative, failing to provide a targeted solution for the top wide-bandgap perovskite in the triple-junction device.

6. Is SIMIT qualified to certify the efficiency of triple-junction tandem solar cells? The accuracy of tandem solar cell efficiency measurements is critically dependent on the spectral match of the solar simulator. What is the spectral mismatch factor of the YSS-T155-2M simulator? Does it satisfy the standard requirements for testing the efficiency of triple-junction tandem solar cells?

7. Regarding the responses to comment #8, the explanation for the current density of the single-junction MBG devices remains unconvincing. Numerous publications (Nat. Energy, 10, 1371-1381, 2025; Nat. Photonics, 20, 55-62, 2026; Angew. Chem. Int. Ed., 64, e202505876, 2025) have demonstrated that devices (~1.52 eV) based on the same substrate can achieve significantly higher currents than those in this study. Particularly within the triple-junction device system addressed in this study, single-junction current densities exceeding 26 mA cm^{-2} have been achieved (Nature Materials, 2025, DOI: 10.1038/s41563-025-02367-8).

In the revised manuscript, the perovskite film thickness exceeds 1 μm in both the planar SEM (Figure 2c) and the textured SEM (Figure 2c). For an intermediate-bandgap perovskite, a current density of only 23.76 or 23.36 mA/cm^2 is clearly defies common sense in this research field! Most importantly, the EQE (Figure 2g and 2i) shows a significant drop in the 600–800 nm range, which is markedly inconsistent with the claimed film thickness. The authors are requested to verify the consistency of these data.

Therefore, these data in the revised manuscript clearly do not support the authors’ claim: “To overcome the current density limitations in the middle cell, a three-step perovskite deposition strategy enables the formation of thick…(Line 97-98)”.

8. The presentation logic for PV performance in Figure 3 requires adjustment to more clearly highlight the innovative contributions of this study. It is recommended that Cell-1, Cell-2, and Cell-3 in Figure 3 be replaced with independent optimization control groups corresponding specifically to the innovations of this study. Specifically, please present data for 3-junctions optimized with: (1) 4-HBA additive, (2) the three-step deposition strategy, (3) SiOx nanoparticle reflectors, rather than devices with varied SAMs. This will allow for a visual isolation of the independent contribution of each specific innovation to device performance, avoiding confusion caused by variations in other factors.

9. Figure 2h and 2i in the revised manuscript display the reflection and absorption of the middle-bandgap perovskite layer. The data reveal that following SiOx-np optimization, both reflection and absorption of this layer increase simultaneously-a phenomenon that contravenes fundamental physical principles. Furthermore, the authors assert that SiOx-np, functioning as an intermediate reflective layer, reflects photons with wavelengths >775 nm back into the middle cell while reducing transmission to the bottom cell. This claim is directly contradicted by the data presented in Figure R3b.

10. The wide-bandgap perovskite in the three-junction device compound primarily consists of FA^+ and Br^- ions, yet the authors' theoretical calculations are based on the $CsPbI_3$ model. Given the significant discrepancy between this model and the actual material composition, the authors are requested to clarify the specific rationale and justification for selecting this particular model.

11. In Figure 4a, why is the initial value of the normalized PCE not 1? The retention rates labeled on the right y-axis (24.5% and 39.5%) are easily confused with the PCE values.

12. The authors expanded the effective active area of the triple-junction device from 1 cm^2 to 54 cm^2 and provided an analysis of performance losses in Supplementary Note 2. However, the analysis appears overly broad, lacking a clear logical prioritization of the primary and secondary loss mechanisms.

13. The authors utilized non-radiative recombination energy loss (NR-loss) analysis for molecular screening (Figures S1 and S2). However, both the main text and supplementary materials lack detailed descriptions of the key testing and calculation procedures.

14. The data and figure processing lack rigor. For example: The current layout and clarity of Figures 4b and 4c do not meet the publication standards of a high-quality journal. The authors are requested to readjust the layout and improve the image resolution to enable accurate interpretation by readers.

Referee #3

(Remarks to the Author)
I co-reviewed this manuscript with one of the reviewers who provided the listed reports.

Referee #4

(Remarks to the Author)
The manuscript entitled “30%-efficient perovskite-perovskite-silicon triple-junction solar cells with improved carrier and photon management” with Ref # 2025-07-17350, by Artuk et al., reports monolithic (series-connected) perovskite-perovskite-Si triple-junction photovoltaics reaching certified percentage conversion efficiencies (PCEs) at or above 30%. The study targets two central performance bottlenecks specifically in triple-junction perovskite-perovskite-Si triple-junction solar cells:

(i) open-circuit voltage (Voc) limitations in the wide-bandgap (WBG) top sub-cell, and (ii) photocurrent limitation in the middle-bandgap (MBG) perovskite sub-cell due to constraints on absorber thickness and related transport and stability penalties. The authors propose a co-ordinated "carrier and photon management" approach: a non-volatile additive (4-hydroxybenzylamine, 4-HBA) to regulate WBG crystallization and passivate defects, then a 3-step/hybrid deposition method to form thick MBG absorbers while retaining extraction and suppressing detrimental mobile ion effects, and a special optical "middle reflector" based on low refractive index SiOx nanoparticles accumulating in textured silicon valleys to redistribute photons toward the MBG sub-cell. Importantly, the revised submission includes third-party certification from the Shanghai Institute of Microsystem and Information Technology (SIMIT) and expanded mechanistic evidence, as stated in detail in the rebuttal from the authors.

Overall, the revised manuscript has been substantially strengthened compared with the prior version, particularly through: (1) independent certification of >30% efficiency and steady-state reporting and (2) reorganization and added characterization intended to sharpen mechanistic claims. The work sits at an appropriate point in the perovskite multi-junction PV field: triple-junction solar cell demonstrations are increasingly expected to justify not only record metrics but also a clear, transferable step forward in the progression toward higher PCEs. The present study is close to that standard and is likely suitable for publication in Nature after some further minor revisions below are addressed:

General Comments and Concerns:

Firstly, thank you to the authors for making the necessary revisions to the manuscript. The clarity and detail have been improved, especially with the contributions from the additional authors. The revised manuscript now highlights the mechanistic role of 4-HBA and of the optical middle reflector. This is the correct overall direction, however, the text would benefit from minor revisions to more clearly present the work as a coherent and broadly applicable framework.

The main text should clearly separate general, transferable mechanisms from device-specific optimizations. In particular, the authors should state which effects of 4-HBA are expected to be broadly applicable (for example, its influence on defect formation and recombination pathways) and which performance gains depend on specific architectural choices, such as the SAM stack or surface texturing.

This is not a request for additional experiments, but for clearer explanation. The manuscript would benefit from one short paragraph that explicitly links the key experiments to the main claims. For example, the authors could briefly state which measurements support improved crystallization and which support defect passivation, as well as which support reduced recombination, and clarify which alternative explanations were excluded. Although the rebuttal notes the addition of PL, device measurements, ToF-SIMS, transient SPV, fracture energy, and BACE data, the manuscript should ideally present these results as parts of a single coherent story, rather than as separate pieces of supporting evidence.

Secondly, the certification and stabilized efficiency reporting are a major strength, but reporting clarity can be improved. Given that independent certification is a central for record devices, and the revised submission now includes SIMIT certification reports and comparisons with in-house curves (Figs. S70–S72) and additional inter-lab comparisons (Fraunhofer ISE) for sub-30% devices. Although, this is a strong response to prior review requests, the main text should ensure that the reader cannot misinterpret which numbers are (i) in-house reverse scan, (ii) in-house stabilized MPP, (iii) certified reverse scan, and (iv) certified steady-state. The manuscript does provide these categories but given the prominence of the "30%" headline, it would be beneficial to present them in a compact, explicit manner in the Results, with consistent terminology and matching device IDs.

The reviewer appreciates the authors' detailed discussion in the rebuttal regarding the limitations of integrating measured EQEs in monolithic multi-junction devices, particularly in the presence of bias-dependent effects, luminescent coupling, and non-ideal sub-cell behavior. These considerations are well established in the III–V community and are indeed relevant for perovskite–silicon tandems and triple-junctions, as correctly described. The concern about potential misinterpretation of current matching based on absolute EQE integration is therefore well-taken. That said, it would still be valuable for readers to have access to estimated short-circuit current densities for the individual top, middle, and bottom sub-cells, even if these values are not presented in the main figure and are accompanied by appropriate caveats. One possible compromise would be to report integrated EQE-derived current densities for representative devices in the Supplementary Information, together with a short Supplementary Note explicitly outlining the known limitations, potential artifacts, and measurement sensitivities. This would preserve methodological rigour while still aligning with common practice in the perovskite multi-junction literature, where such estimates are routinely reported to facilitate comparison and interpretation.

Minor Concerns:

The authors could add a short statement clarifying whether the certified steady-state measurement conditions (illumination spectrum, temperature control, preconditioning, stabilization time) are fully matched to the in-house stabilized protocol, or if not, what differences exist.

Ensure the certified steady-state values are given equal prominence to reverse scans, especially since the work emphasizes stabilized efficiencies.

For the middle-cell three-step/hybrid deposition, there is outstanding performance, but reproducibility and trade-offs should be explicit (possibly list some pros and cons). The manuscript reports that a champion device reached reverse-scan efficiency of 31.15% with stabilized efficiency 30.9%, and batch statistics of 22 devices average 29.65%. This is impressive

and suggests good repeatability at the device level. However, the three-step MBG deposition concept, while clearly effective, raises questions that should be addressed more directly:

• For example, is the process window tight or broad? The text implies robust performance but does not clearly communicate yield in terms of how many wafers/batches were attempted and how representative the champion batch is.

• What is the primary trade-off? For example, does increased thickness introduce higher series resistance, more pronounced hysteresis, or new failure modes under damp heat and outdoor conditions?

• The Supplementary Information indicates that residual PbI2 can appear in “under-converted” three-step absorbers (Fig. S67), while one-step absorbers can look more homogeneous in some cross-sections, which could confuse readers if not carefully explained. The authors should ensure the message is unambiguous for when and why residual PbI2 is observed, whether it is beneficial, neutral, or detrimental, and how it correlates with device-to-device spread.

• For the stability discussions, there is good progress, but could the authors align the narrative with realistic deployment expectations? The Supplementary Figures include damp heat testing (noting 22.9% relative PCE loss after 500 h for an encapsulated 3J device) and outdoor monitoring setup images with no visible degradation on a specific date. This is useful, but the stability story would benefit from providing a succinct “stability summary” in the main text describing the test conditions (temperature, RH, encapsulation type, MPP tracking versus periodic JV, and duration). Even if the detailed plots remain in the Supplementary Information, the reader needs a coherent takeaway in the main paper (1-2 sentences). No new experiments are necessarily required, but the stability claims must be carefully framed to avoid overstating “stability” based on limited windows.

• In Supplementary Note 1, it may be premature to describe the Me-4PACz/perovskite interface as the “weakest layer” in the device stack unless fracture energy measurements have been performed for all relevant interfaces throughout the full stack. In the broader perovskite–silicon tandem literature, the perovskite/C60 interface is more frequently identified as one of the mechanically weakest interfaces when assessed by fracture energy. The authors may wish to consider recent work by Poma et al., EES Solar (accepted February 2026), “Incorporating a Naphthalene Diimide Polymer into a Fullerene Electron-Transport Layer to Improve the Fracture Energy of Perovskite Solar Cells,” which directly addresses this issue (although on the electron transport layer side). This reference may also be relevant to the discussion in the main text (Page 8, Lines 327–333). Including for stability as C60 is often the weakest link.

Minor Concerns and Suggestions:

• The rebuttal explains why symmetric double-sided nanotexturing was used and why asymmetric texturing adds processing complexity. This is a reasonable response, however, in the manuscript, the authors could consider adding one sentence clarifying whether the present optical gains are expected to persist under asymmetric industrial texturing approaches.

• A short paragraph in the Discussion could help clarify what stability milestones remain for perovskite-perovskite-Si triple-junctions (for example, damp heat duration expectations) and where this work currently stands, without overclaiming (more for future work and directions) (optional).

• Page 10, Line 414: When the authors refer to overall efficiency gain of 0.66%abs, it’s not clear from looking at Table S2. Can the authors be more explicit with what numbers they are referring to please?

• Several multi-panel figures are quite compact. If allowable within the Nature journal format, the authors could opt to use slightly larger axis labels or a simplified legend would help with readability.

• Page 13, Figure 4(E). The PR could be explained in detail, as it’s not really a common metric in the PV field.

• Page 9, JV and EQE in Fraunhofer ISE: In the context of the spectral response and current-matching discussion in the Supplementary Information, it would be appropriate to also cite the earlier work by Meusel et al. on spectral response measurements and measurement artifacts in monolithic triple-junction devices (Prog. Photovolt. Res. Appl. 11, 499–514 (2003)). This paper complements Ref. 28 and provides useful historical context for spectral response artifacts in series-connected multijunction solar cells.

Grammatical Errors and Corrections:

Main Article:

Page 1, Line 64: “nanoparticles accumulated in the front valleys...” to “nanoparticles that accumulate in the front valleys...”

Page 2, Line 86: “...among the sub-cells; ...” standardize “sub-cells” vs “subcells” (both appear later). Or just pick one that, but please keep consistent. Similarly, for “middle-cell” throughout the text.

Page 2, Line 89: “...perovskite-Si-based triple junctions...”, again standardize to “triple-junctions” to match the majority usage.

Page 2, Line 95: “...through a coordinated carrier and photon management strategy.” consider hyphenating “carrier- and photon-management strategy”.

Page 2, Line 101: “SiOx-nanoparticles (SiOx-np) ...” please standardize terminology to one preferred form (e.g., “SiOx-np” thereafter, and avoid switching back to “SiOx nanoparticles” later).

Page 7, Line 296:
Current: “...constrained to thicknesses below ~ 800 nm...” remove the space after the ~, so “~800 nm”.

Page 10, Line 399: “Fig. 47(b)” should be “Fig. S49(b)”?

Page 10, Line 435-438: “To decrease such a mismatch in the further experiments, the top-cell bandgap was increased to ~1.9 eV from 1.8 eV by increasing the Br fraction to 50%. To decrease such a mismatch in the further experiments, the top-cell bandgap was increased to ~1.9 eV from 1.8 eV by increasing the Br fraction to 50%.” This sentence has been repeated twice!

Page 12, Line 486: “(reverse scan efficiency of 27.10%) (15).” remove the double space before the reference: “...27.10%) (15).”

Page 12, Line 491: “The iVoc of the three-step middle-cell...” please standardize “iVoc” to “iVOC” to match usage in Lines 487–489 and Line 493.

In general, there are many hyphenation inconsistencies, such as subcell vs sub-cell, middle-cell, triple-junction, SiOx-nanoparticles (SiOx-np), etc. Please check carefully and keep consistent throughout the manuscript. Also, please ensure consistent use of Ethanol versus EtOH, PV-tools capitalization, etc. The rebuttal indicates these were fixed, but could the authors please do a final check.

Supplementary Materials:

Figure S37. Text is too small. Please resize for readability.

Figure S41. For the XRD figure, please consider labelling the individual peaks. E.g. (001), (011), PbI2, etc.

Figure S62. Text is too small and colour choice makes readability difficult. Please consider improving this.

After careful consideration, the revised manuscript is suitable for publication in Nature pending minor revisions. The authors have convincingly addressed the major points raised in the initial review, particularly through independent third-party certification above 30% (including steady-state values) and through expanded experimental and mechanistic analysis supporting the wide-bandgap additive strategy, the three-step middle-bandgap absorber approach, and the optical middle reflector.

The remaining comments primarily concern clarity and presentation. In particular, the manuscript would benefit from clearer performance reporting, more explicit distinction between general mechanisms and device-specific optimizations, and some general (big picture) discussion of reproducibility, trade-offs, and stability. Addressing these points should be straightforward and would further strengthen the manuscript.

Open Access This Peer Review File is licensed under a Creative Commons Attribution 4.0 International License, which permits use, sharing, adaptation, distribution and reproduction in any medium or format, as long as you give appropriate credit to the original author(s) and the source, provide a link to the Creative Commons license, and indicate if changes were made.
In cases where reviewers are anonymous, credit should be given to 'Anonymous Referee' and the source.
The images or other third party material in this Peer Review File are included in the article's Creative Commons license, unless indicated otherwise in a credit line to the material. If material is not included in the article's Creative Commons license and your intended use is not permitted by statutory regulation or exceeds the permitted use, you will need to obtain permission directly from the copyright holder.
To view a copy of this license, visit https://creativecommons.org/licenses/by/4.0/

We sincerely thank all reviewers for their thoughtful and constructive comments, which have significantly improved the quality and clarity of our manuscript. Our detailed responses to each remark are provided below in blue. During revision, we have refined the manuscript title to ensure consistency with the independently certified device performance.

We would also like to inform the editor and reviewers that, following additional contributions during the revision process, several contributors have been added to the author list in accordance with their substantial contributions:

1. Joël Spitznagel fabricated numerous high-quality bottom-cells utilized during this work in EPFL PV-Lab.
2. Jonathan S. Austin contributed new ToF-SIMS measurements carried out at Empa.
3. Andrés-Felipe Castro-Méndez and Felix Lang performed the BACE measurements at Potsdam University.
4. Thomas W. Gries, Siddha Hill, and Artem Musiienko, conducted the transient SPV measurements at HZB.
5. Muzhi Li and Nicholas Rolston carried out the fracture energy measurements and analyzed them at Arizona State University.
6. Cheng Liu supported the certification process at Northwestern University.
7. Jun Zhao supported the certification process at CSEM.

We believe these additions appropriately reflect the newly included data and ensure proper acknowledgment of all contributors' efforts.

Reviewer 2&3

This manuscript addresses two primary limitations in perovskite–silicon triple-junction solar cells: (i) the low open-circuit voltage of the wide-bandgap top cell and (ii) the current density constraint of the middle-bandgap subcell. The authors employ additive engineering to regulate crystallization kinetics and reduce bulk recombination in wide-bandgap perovskites, implement a three-step deposition method to fabricate thicker middle-bandgap absorbers, and utilize SiO_x nanoparticle-based back reflection to enhance photocurrent. As a result, the authors claim that the device achieves a steady-state efficiency of 30.5%. While this efficiency seems to exceed the previous certified record of 27.6% (Nature, 628, 306–312, 2024), the manuscript does not provide any formal certification reports—precluding apple-to-apple comparison.

In addition, the scientific findings primarily consolidates well-established knowledge (such as additive-assisted crystallization, absorber thickness optimization, and nanoparticle-mediated light management) rather than introducing any new concepts. Too many perovskite papers simply use some additives (whether random or well-known), achieve high efficiency, and then make up a story to get published. This is not how the field works, especially for an extremely high-impact journal like Nature. As the most authoritative scientific journal, Nature has the commitment to publishing work that not only reports the record efficiency but also focuses more on scientific concepts capable of inspiring and advancing the perovskite field.

We sincerely thank the reviewers for highlighting several important points. We have carefully addressed their concerns and revised the manuscript accordingly to improve its overall quality.

Thus, the authors should seriously and clearly articulate how their work advances beyond existing studies and emphasize its unique scientific contribution. Major concerns are as follows:

1. Additive engineering is a well-established approach for controlling crystallization kinetics and enhancing film quality. Recent work by De Wolf et al. (Nature, 628, 93–98, 2024) demonstrated the efficacy of 4-hydroxybenzylamine in this context, which is EXACTLY the SAME in this work. Compared to previous studies, this manuscript offers no new insights into additive selection or mechanistic understanding. The authors are encouraged to provide original perspectives on the rationale for material selection and nucleation–crystallization dynamics to strengthen its academic impact. It is very important the authors need to conduct an in-depth investigation into whether there is an universal scientific principle for this additive that other researchers can learn from regarding how this additive affects wide-bandgap perovskites.

We thank the reviewer for this valuable comment and for highlighting the importance of clarifying the novelty of our additive strategy relative to the recent work by De Wolf *et al.* (*Nature* 628, 93–98, 2024). Whereas previous studies employed 4-HBA as an interfacial modifier to form quasi-2D surface layers, we demonstrate that the same molecule can act as a bulk crystallization regulator, establishing a new additive design paradigm based on transient precursor coordination rather than surface reconstruction.

While that study employed 4-hydroxybenzylamine (4-HBA) or its salt as a surface-applied treatment to form quasi-2D perovskite layers at the top and bottom interfaces, our approach is fundamentally different in both purpose and mechanism. In our work, 4-HBA is incorporated directly into the precursor ink, where it functions as a crystallization modulator and defect passivator rather than as an interfacial agent. This bulk additive engineering strategy enables 4-HBA to be distributed throughout the perovskite film, rather than being confined to interfaces, thereby enhancing defect passivation and optimizing energy level alignment at the HTL-perovskite interface. Importantly, no evidence of 2D perovskite formation is observed, confirming that 4-HBA does not induce phase reconstruction but instead modulates the crystallization dynamics of the 3D perovskite lattice.

Building on atomistic insights from DFT, molecular-level information from NMR, crystallization dynamics from in-situ GIWAXS, and ex-situ chemical and optoelectronic analyses, we establish a comprehensive mechanism by which 4-HBA governs nucleation dynamics and defect chemistry in the perovskite absorbers. Unlike additives that form new hybrid complexes within the ink, 4-HBA interacts with perovskite precursors without generating distinct molecular phases (*Science* 366, 7, 49–753 (2019) and S*cience* 385,533-538(2024)). This stable coordination maintains ink stability (Fig.S4) and preserves the native ionic equilibrium, ensuring compatibility across diverse perovskite formulations. DFT reveals strong Pb^{2+} binding via the amine group, which transiently stabilizes precursor adducts and retards crystallization, consistent with in-situ GIWAXS results. Moderated crystallization promotes lattice reorganization, yielding larger, well-oriented domains with reduced boundary density and fewer defects. ToF-SIMS confirms 4-HBA distribution throughout the film, in contrast to volatile or interface-accumulating additives (*Joule* 8, 999–1008 (2024), *J Am Chem Soc* 145, 5920–5929 (2023), *Nat Commun* 16, 9894 (2025)). The bulk distribution further enhances chemical passivation by extending beyond the top and bottom interfaces, and optimizes energy alignment at the HTL-perovskite interface (see Fig.S25 for comparison of bottom surface treatment vs. additive). Across four different compositions, 4-HBA consistently suppresses non-radiative losses (see Fig.S23 and Fig.1(b)). Collectively, 4-HBA offers a universal, composition-tolerant route to modulate crystallization and defect formation, coupling solution-phase control with solid-state passivation to enhance film quality and device performance.

Overall, although the underlying defect chemistry shares some conceptual similarity with the surface passivation strategy reported by De Wolf et al., the physical distribution, functional role, and mechanistic effect of 4-HBA in our work are distinct. We clarify these distinctions in the revised manuscript and expand the mechanistic discussion to emphasize how the multifunctional nature of 4-

HBA provides a generalizable framework for molecular modulation of crystallization and defect passivation in wide-bandgap perovskites.

We added the following paragraph on the detailed mechanistic function of 4-HBA and the following supplementary figures as a result of new experiments (Fig.S23, 24, 25).

"Building on atomistic insights from DFT, molecular-level information from NMR, crystallization dynamics from in-situ GIWAXS, and ex-situ chemical and optoelectronic analyses, we establish a comprehensive mechanism by which 4-HBA governs nucleation dynamics and defect chemistry in the perovskite absorbers. Unlike additives that form new hybrid complexes within the ink, 4-HBA interacts with perovskite precursors without generating distinct molecular phases (***24***). This stable coordination maintains ink stability (Fig.S4) and preserves the native ionic equilibrium, ensuring compatibility across diverse perovskite formulations. DFT reveals strong Pb^{2+} binding via the amine group, which transiently stabilizes precursor adducts and retards crystallization, consistent with in-situ GIWAXS results. Moderated crystallization promotes lattice reorganization, yielding larger, well-oriented domains with reduced boundary density and fewer defects. ToF-SIMS confirms 4-HBA distribution throughout the film, in contrast to volatile or interface-accumulating additives (***18*, *35–37***). The bulk distribution further enhances chemical passivation by extending beyond the top and bottom interfaces, and optimizes energy alignment at the HTL-perovskite interface (see **Fig.S25** for comparison of bottom surface treatment vs. additive). Across four different compositions, 4-HBA consistently suppresses non-radiative losses (see **Fig.S23** and **Fig.1(b)**). Collectively, 4-HBA offers a universal, composition-tolerant route to modulate crystallization and defect formation, coupling solution-phase control with solid-state passivation to enhance film quality and device performance."

2. In wide-bandgap perovskites (Eg > 1.9 eV) used as top cells in triple-junction configurations, halide phase segregation remains a significant challenge. The authors apply conventional additive engineering to regulate crystallization and chemical coordination to passivate defects, thereby reducing non-radiative recombination and improving open-circuit voltage. However, these techniques and related scientific questions are extensively reported. The manuscript lacks an insight into the mechanism for suppressing phase segregation or a generalizable strategy to address this issue, limiting its contribution to advancing the field.

Rather than targeting halide phase segregation directly, this work focuses on suppressing the dominant efficiency- and stability-limiting pathways under operating conditions. High-resolution EQE and transient absorption measurements show that the addition of 4-HBA mitigates phase segregation to some extent, although residual segregation remains detectable. Importantly, wide-bandgap single-junction devices exhibit remarkable operational stability at elevated temperatures (Fig.4a–b), even when phase-segregation signatures emerge in photoluminescence measurements after short illumination times. The incorporation of 4-HBA further enhances this stability by delaying or suppressing yellow-phase formation. These observations indicate that device degradation is governed primarily by decomposition into PbI_2-rich or δ-phase structures, rather than by halide segregation itself.

3. Increasing absorber thickness to enhance light harvesting via three-step deposition is a common method. For instance, Tan et al. and Li et al. (Nature, 620, 994–1000, 2023; eScience, 100449, 2025) reported similar methods with notable efficiency gains. Given the similarities, thickness optimization alone does not establish novelty. The authors should explore distinctive effects on interface evolution, phase stability, or carrier dynamics to differentiate their work from existing literature.

We thank the reviewer for raising this point. Prior work (e.g., Tan et al. - *Nature*, 620, 994–1000, 2023) used hybrid deposition atop a solution-processed perovskite to form a 3D/3D bilayer with a ~50 nm hybrid layer that primarily modifies interfacial properties and improves energy level alignment with fullerene electron transport layer. In contrast, our objective is to simultaneously increase the absorber thickness and reduce the absorber bandgap while preserving microstructural integrity. Attempts to

achieve this solely by modifying ink chemistry or deposition conditions degraded device performance, which is consistent with why state-of-the-art single-junction perovskites typically retain similar solution-processing conditions to our absorber (adapted from *Science* 384, 189–193 (2024) and *Science* 382, 810–815 (2023)). The origin of such performance degradation is identified as differences in morphology (e.g., formation of wrinkles due to excess mechanical stress in thick absorbers) and enhanced ionic losses.

We identify the origin of this degradation as mechanical and morphological instability in thick one-step antisolvent-processed films (see Supplementary Note 1). SEM and optical microscopy reveal that such films develop pronounced wrinkling and surface roughness despite retaining large, columnar grains. These features introduce excess mechanical stress, promote ionic transport, and exacerbate non-radiative recombination losses, effects that offset the expected photocurrent gains from increased optical absorption. Similar limitations have been reported for thick middle cells in perovskite triple-junction devices, underscoring the generality of this challenge.

To overcome these constraints, we developed a three-step deposition strategy that fundamentally alters interface evolution, adhesion, and carrier dynamics. Unlike thick one-step films, three-step absorbers maintain smooth, mirror-like surfaces comparable to thin absorbers, while enabling substantially thicker and lower-bandgap films without wrinkling or delamination. Fracture-energy measurements show significantly enhanced adhesion at the perovskite/SAM interface, particularly for 4PABCz, where the measured critical energy release rate ($G_c = 1.04 \pm 0.17$ J m^{-2}) represents a lower bound for the true interfacial adhesion strength. Notably, fracture no longer localizes at the SAM/perovskite interface, indicating that this interface is no longer the mechanically weakest layer in the stack (see Fig.S33).

Importantly, these mechanical improvements, that ensure lateral homogeneity, are directly coupled to preserved optoelectronic quality. In contrast to thick one-step absorbers, three-step films maintain efficient charge extraction and do not exhibit elevated mobile ionic density. This is confirmed by transient surface photovoltage (tr-SPV) measurements, which show enhanced extraction velocity and amplitude at the perovskite/C_{60} interface (see Fig.S37), as well as by bias-assisted charge extraction measurements, which reveal stable mobile ion densities. These results demonstrate that the three-step process suppresses stress-induced ionic losses while preserving fast carrier dynamics and high fill factors.

Moreover, hybrid deposition in our approach enables controlled compositional engineering: the deposited layer is effectively $FAPbI_3$-rich, allowing a deliberate and homogeneous bandgap reduction of the underlying absorber ($\approx$1.53 eV $\rightarrow$ $\approx$1.52 eV) without compromising film integrity. This controlled bandgap tuning, combined with enhanced adhesion and preserved microstructure, is not achievable in thick one-step absorbers and is distinct from previously reported hybrid bilayer approaches.

These results demonstrate that mechanical integrity and ionic stability, rather than optical thickness alone, constitute the fundamental limitation of thick perovskite absorbers, an insight that extends beyond triple-junction architectures. In summary, our three-step strategy represents a coordinated optimization of thickness, composition, interface adhesion, microstructure, and carrier dynamics, rather than a simple thickness increase. By eliminating wrinkling, suppressing ionic losses, strengthening SAM–perovskite bonding, and preserving efficient charge extraction, this approach enables thick, low-bandgap absorbers that deliver higher JSC without sacrificing FF, thereby addressing a critical bottleneck for efficient middle-cell operation in perovskite-based triple-junction solar cells.

4. The use of SiO_x nanospheres to enhance light harvesting on textured silicon surfaces was recently reported (Nature Communications, 16, 7331, 2025). However, the insulating nature of SiO_x raises concerns about the trade-off between improved optical reflection and potential reductions in electrical contact or transmittance, which could compromise the overall optical design. The manuscript should

clarify how this strategy integrates into the device's optoelectronic framework and provide data on these trade-offs, beyond focusing solely on photocurrent enhancement.

We thank the reviewer for raising this point. We have previously demonstrated the use of SiO_x nanoparticles (SiO_x-np) in perovskite–Si tandems as a back reflector at the rear of the c-Si bottom cell (Solar RRL 9, 2400704 (2025)), and SiO_x-np have already been demonstrated as an intermediate reflector in µc-Si/a-Si tandems (*Nano Lett.* 2014, 14, 9, 5085–5091).

Owing to the refractive-index contrast at a wavelength of ~800 nm, n(SiO_x-np) ≈ 1.33 and n(perovskite) ≈ 2.5, the perovskite-SiO_x-np interface exhibits enhanced Fresnel reflection. In this spectral range utilized as a back-reflector, light reaching the rear is predominantly IR, so the layer functions as an IR reflector that increases the optical path length and thus absorption in c-Si.

In the present work, we transpose this concept to an intermediate reflector on the front side of the c-Si bottom cell, analogous to nc-SiO_x or SiO_x-np intermediate reflectors long used in micromorph triple-junctions (*Phys. Status Solidi (c)* 9, 2145–2148 (2012), and *Nano Lett.* 14, 5085–5091 (2014)).

SiO_x-np placed at the perovskite/SiO_x-np/c-Si interface reflects photons >775 nm back into the middle cell, enhancing band-edge absorption. As expected, there are trade-offs. First, the IR light transmitted into the bottom cell decreases, causing 0.71 mA cm^{-2} loss in the bottom-cell (from the EQE of the middle-band-gap, MBG–Si tandem; Fig.2I). Second, the reduced ITO/SAM/perovskite contact area introduces additional series resistance ($\Delta R_s \approx 1\ \Omega \cdot cm^2$), lowering FF (Fig.S52). However, the MBG cell gains 0.52 mA cm^{-2} (see Fig.S50), and our equivalent-circuit analysis together with device data shows that this gain offsets the losses, yielding a net PCE increase (Table S2 and Fig.S59).

Regarding the recent report in *Nature Communications* 16, 7331 (2025) by Zhang *et al.*, we have concerns about the optical interpretation of "SiO_x-np." Introducing a low-index interlayer between the perovskite and Si sub-cells should increase reflectance at $\lambda \gtrsim$ 750–800 nm, thereby reducing NIR/IR transmission into the bottom cell. In contrast, Zhang *et al.* report reduced reflectance at this interface. The results in the paper are based on the assumption that the refractive index of SiO_x nanoparticles is ≈2.7, citing Opt. Express 26, A750–A760 (2018), which focuses on nanocrystalline SiO_x films rather than SiO_x-np. While nc-SiO_x thin films can indeed exhibit n ≈ 2-3, nanoparticulate SiO_x networks are materially different and typically have much lower effective refractive index (≈1.3–1.5) (*Nano Lett.* 14, 5085–5091 (2014)). We have clarified in the manuscript that our SiO_x-np layer is a low-index intermediate reflector, and we updated the measured reflectance at the perovskite/SiO_x-np/c-Si interface (see Fig.2h).

5. Although efficiency measurements were conducted by Fraunhofer ISE and EPFL, the current manuscripts lack formal certification reports from authoritative institutions. In high-impact journals, key efficiency claims typically include such certification (e.g., Nature, 635, 596–603, 2024; Nature, 623, 313–318, 2023). It is COMPULSORY to include FORMAL certified reports to ensure the reliability and global credibility of their findings.

We sincerely thank the reviewer for highlighting this important point regarding independent validation. Our group has a strong and well-established record of working with top-tier certification institutes. To date, we have reported six independently certified results published in *Science*, *Joule*, *Advanced Materials*, and *Nature Communications*. These certifications underscore our longstanding commitment to rigorous, transparent, and globally credible performance reporting.

For the present work, owing to the limited availability of formal certification slots at these institutes before the submission, we conducted third-party measurements at Fraunhofer ISE. Fraunhofer ISE has decades of experience in the characterization of III–V multijunction solar cells and operates a state-of-

the-art LED-based solar simulator that provides the precise spectral control required for accurate triple-junction device characterization. Importantly, Fraunhofer ISE routinely cross-checks their dual-junction measurements against those from CalLab Cells and consistently reports excellent agreement.

Furthermore, in response to the reviewers' request, we have obtained two formal independent certifications from Shanghai Institute of Microsystem and Information Technology (SIMIT), which are now included in the revised manuscript (Fig. S70-S71). The devices were independently certified with power conversion efficiencies of 30.44% and 30.18% from reverse JV scans, and 30.02% and 29.76% under steady-state conditions, in excellent agreement with our in-house measurements, differing by only ~0.1 percentage points in absolute efficiency (see Fig. S72 for a direct comparison). These results represent a significant improvement of 3.34% absolute power conversion efficiency compared to the previously highest certified reverse-scan efficiency of 27.10% again certified in SIMIT (Nature 628, 306–312, 2024).

6.This manuscript still lacks an important result regarding device stability, namely the operational stability of triple-junction tandem solar cell devices, which should be evaluated through maximum power point tracking, rather than only focusing on the operational stability of WBG solar cells.

Our indoor accelerated aging setup, equipped with five LED light sources (Cicci Research), enables reliable testing of single-junction and tandem devices. However, operational stability in monolithic triple-junction solar cells is inherently challenging to assess under indoor conditions, owing to the stringent spectral requirements. While our calibrated indoor MPPT setup allows controlled accelerated aging of single-junction and tandem devices, it is not spectrally optimized for triple-junction architectures. We therefore evaluate the operational stability of triple-junction devices under outdoor conditions, where realistic spectral variations and thermal coupling between sub-cells are naturally captured. Accordingly, the operational stability of the triple-junction (3J) devices was assessed outdoors in Neuchâtel over a four-month period (see Fig. 4e), whereas indoor MPPT measurements at elevated temperatures (Fig. 4a) were performed on wide-bandgap single-junction (1J) devices. Damp-heat testing (Fig. 4d) and outdoor monitoring were conducted on the 3J devices.

Some other technical issues also require attention, for example:

7.The rigor of the in-situ GIWAXS analysis presented in Figure 1c during the annealing of wide-bandgap perovskites is questionable. According to established literature (Nature Energy, 9, 536-547, 2024; Nature, 624, 557-563, 2023), the characteristic q-value for the 3C cubic perovskite phase is approximately 1.0 nm^{-1}, while those for the 2H, 4H, and 6H phases are approximately 8.6 nm^{-1}, 8.4 nm^{-1}, and 9.3 nm^{-1}, respectively. However, the q-values assigned to these phases in the manuscript deviate significantly from these benchmarks. More critically, the formation of the 3C phase was not observed in the experiments. The authors should provide a reasonable explanation for these discrepancies.

We appreciate the reviewer's careful assessment of our in-situ GIWAXS analysis. The apparent discrepancy in the assigned q-values arises primarily from two factors. First, in Figure 1c, the 6H reflection partially overlaps with the shoulder of the 3C (10.2 nm^{-1}) cubic phase, and the 3C label was inadvertently omitted during figure preparation. We have now corrected the labeling, clearly identifying the 3C phase at ~10 nm^{-1}. Importantly, this correction does not alter any of our scientific interpretations or conclusions. Second, the q-positions of the 4H, and 6H phases in our mixed-halide (I/Br/Cl) perovskite system (which is why we report PbX_2 rather than PbI_2) are slightly higher than those reported for pure iodide references (Nature Energy, 9, 536–547, 2024; Nature, 624, 557–563, 2023), which is consistent with the expected lattice contraction caused by Br and Cl incorporation.

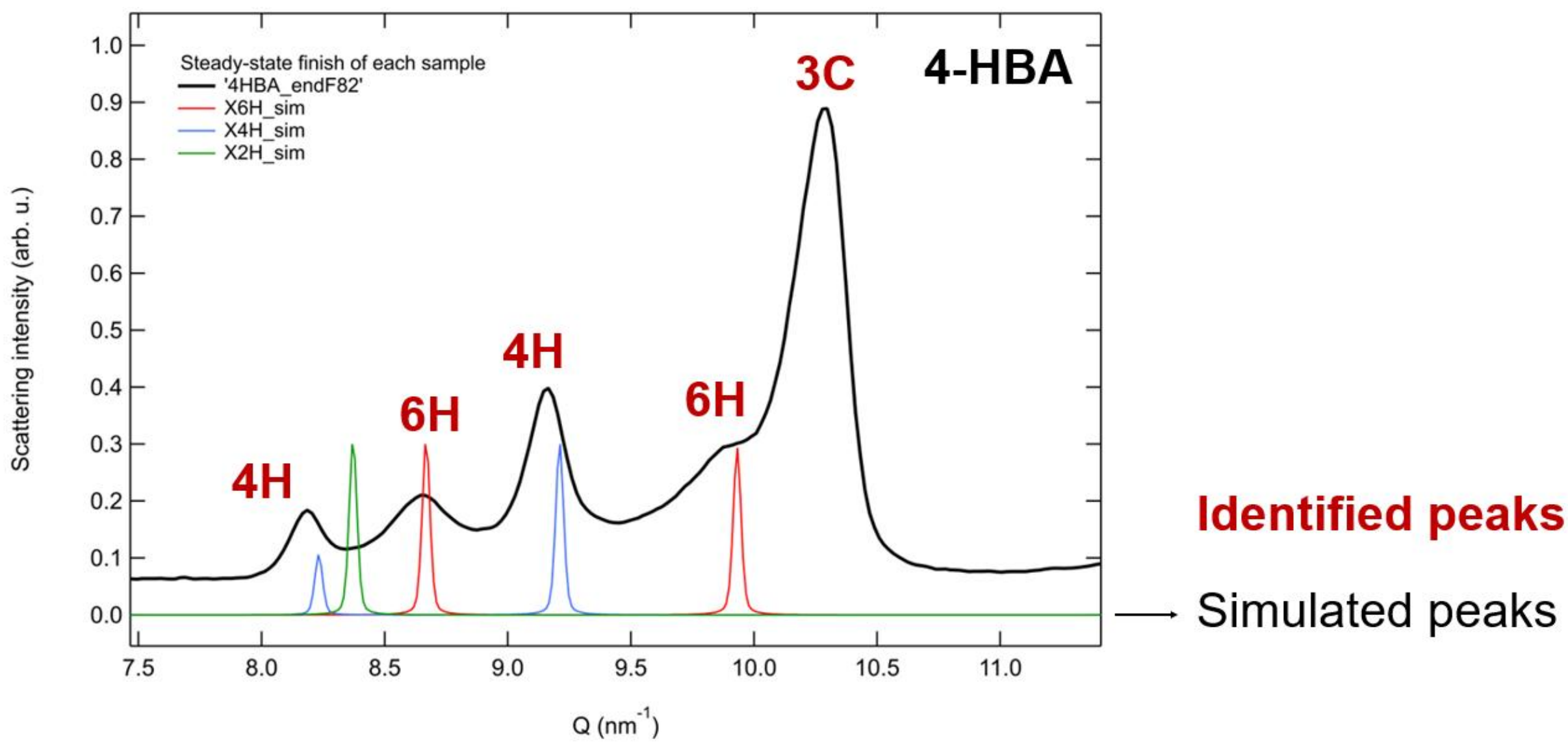

Figure R1. Identified and simulated diffraction peaks.

Following the approach of Gratia *et al.* (ACS Energy Letters **2**, 2244–2250, 2017) and using the corresponding .cif files for the 2H, 4H, and 6H polytypes, we simulated the expected diffraction peaks (see Fig.R1). We plotted them together with the experimental 4-HBA spectra. Comparison between the simulated and measured patterns enabled us to identify the presence of the 4H and 6H phases, with a possible 2H contribution near 8.4 nm^{-1}. The key distinction between the control and target perovskite samples lies in the emergence of the characteristic 4H doublet in the latter and PbX_2, indicating phase evolution upon 4-HBA incorporation.

8.In Figure 2D, the current density of single-junction MBG devices is consistently below 24 mA/cm², regardless of whether a one-step or three-step method is used. This value is strikingly low compared to the field benchmarks: for 1.52 eV perovskite materials, current densities routinely approach 26 mA/cm², with averages exceeding 25 mA/cm² (refs: Nature, 632, 301-306 (2024); Nature, 632, 536-542 (2024); Nat Commun 16, 753 (2025); Adv. Mater., 37, 2505087 (2025)). The authors must provide a detailed explanation for this significant underperformance relative to established standards.

We thank the reviewer for this observation. The single-junction devices in this study indeed exhibit EQE-integrated current densities below 24 mA cm^{-2}. This is expected because, for these experiments, we used ITO/glass without an anti-reflection coating (ARC) and did not employ high-haze textured FTO (the supply of this substrate from Asahi JAPAN is rather scarce, and we also do not have access anymore), see Fig.R2 for comparison of ITO and FTO with ARC (with 1.71 mA cm^{-2} difference). We intended to maintain an optical/electrical stack directly translatable to our 3-junction architecture rather than to maximize single-junction JSC. In prior work, using the same one-step absorber, but with optimized light management (textured FTO and ARC), we approached ~25 mA cm^{-2} from EQE, comparable to state-of-the-art EQEs (25.3 mA cm^{-2} integrated current from EQE by Yang et al. *Science* 386, 898–902 (2024)). Consequently, under the same substrate and ARC conditions, the present three-step absorbers should reasonably reach integrated current from EQEs reaching 25.5 mA cm^{-2}. Moreover, the JSC-JV to JSC-EQE mismatch for our usual scenario is only 0.25 mA cm^{-2}; lower than often observed in literature. We can only speculate about the origin of the mismatch for other laboratories, but given our small mismatches compared to regularly reported ~1mA cm^{-2}, we believe that often JSCs are slightly overestimated.

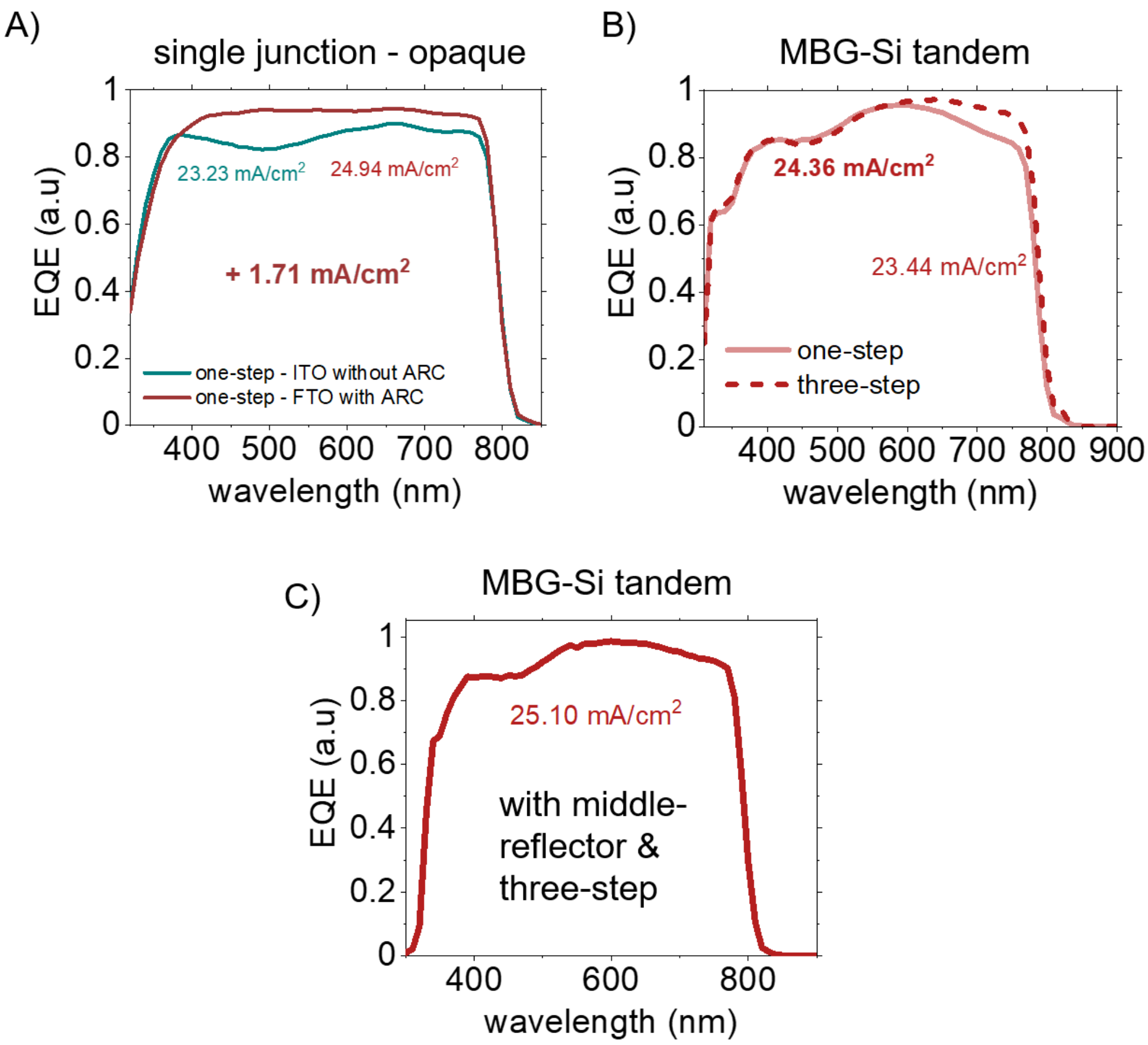

Figure R2. A) Comparison of substrates, B) EQE comparison of one-step and three-step, C) Champion EQE of MBG sub-cell of MBG-Si tandem with middle-reflector and three-step absorbers measured from the film-side with semi-transparent contacts including finger shadowing (1.5%).

Importantly, in the MBG/Si tandems fabricated here, combining the three-step MBG absorber with the intermediate SiO_x-np reflector, the MBG sub-cell reaches >25 mA cm^{-2} EQE-integrated current, despite a relatively thick top-electrode stack. To our knowledge, this is among the highest reported MBG-cell EQEs measured in a semi-transparent configuration, underscoring the significance of our approach (see Fig. R2). We have added clarifying text to highlight the EQE of the MBG-cell in MBG-Si tandems and Fig.S53.

"The integrated current density from EQE in the MBG-cell reaches 25.1 mA/cm^2, which is among the highest current densities for such a device configuration (semi-transparent, illuminated from the film side - in the case of Liu *et al.,* 23.85 mA cm^{-2}, *Nature* 628, 306–312 (2024))."

9.In Figure 2H, the increased reflection from the SiOx-np layer in the Perovskite/SiOx-np/Si structure is likely to be reabsorbed by the thick middle-bandgap perovskite layer. Therefore, comparing the reflectance spectra of Perovskite/SiOx-np/Si with that of Perovskite/Si is inaccurate. It would be more convincing to directly compare the reflectance spectra of SiOx-np/Si and bare Si.

We thank the reviewer for highlighting this point. Indeed, any light reflected at wavelengths below the perovskite bandgap (~800 nm) will be reabsorbed in the middle cell; therefore, the previously observed differences in this region were revisited. We have repeated the measurements, and in the corrected reflectance spectra, the only changes appear after the perovskite absorption edge. Specifically, an increase in reflectance is observed in region 1 (775–900 nm) and region 2 (900–1050 nm). These trends

are fully consistent with the EQE results: in the MBG-cell, enhanced reflectance from the middle reflector sharpens the perovskite absorption edge, while in the Si-cell the same enhanced reflectance reduces the photon flux transmitted to the bottom cell, thereby lowering its EQE response. Thus, the SiOx-np middle reflector is not an isolated optical enhancement, but a deliberately balanced optoelectronic element whose net benefit emerges at the triple-junction level

Following the reviewer's suggestion, we additionally measured the reflectance of SiOx-np/Si and compared it to bare Si. Because the refractive index of SiOx-np (~1.33) provides improved optical impedance matching between air (n = 1) and Si (n ≈ 3.5), it behaves as a conventional anti-reflective coating, reducing reflectance when deposited directly on Si, see **Fig.R3**. However, in the actual triple-junction device, the relevant optical interface is not SiOx-np/Si but perovskite/SiOx-np. In standard back-reflector concepts, reflectance arises when light encounters the large index drop from Si (n ≈ 3.5) to SiOx-np (n ≈ 1.33). Analogously, in our structure, the middle-reflector originates from the refractive-index drop at the perovskite (n ≈ 2.5) → SiOx-np (n ≈ 1.33) interface.

To reflect this clarification, **Fig.2h** has been updated, and the manuscript has been revised to more clearly articulate the underlying optical mechanism.

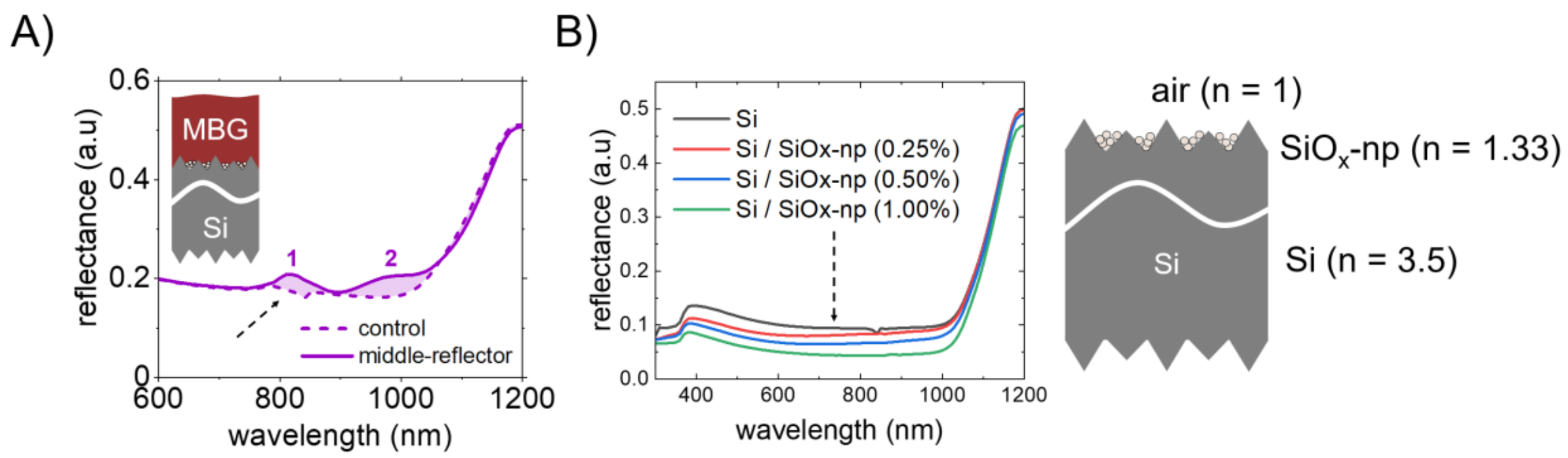

Figure R3.A) Reflectance spectrum of MBG-SiOx-Si stack, B) reflectance spectrum of SiOx-Si stack.

'The reflectance spectra of double-sided sub-micron textured Si coated with perovskite, with and without the SiOx-np middle reflector, are shown in **Fig.2(h)**. Two distinct spectral regions exhibit increased reflectance when the SiOx-np layer is introduced. **Region 1 (≈775–900 nm)** corresponds to wavelengths just beyond the perovskite band edge and is directly associated with enhanced back-reflection into the middle cell, thereby boosting the band-edge response. **Region 2 (≈900–1050 nm)** encompasses wavelengths transmitted toward the bottom Si sub-cell; here, the increased reflectance reduces the photon flux entering the Si. These spectral changes validate the formation of the intended perovskite/SiO_x-np middle reflector arising from the refractive-index drop (from 2.5 down to 1.33) at this interface. An efficient reflector forms when the optical wave encounters a transition from a higher to a lower refractive index; therefore, the perovskite/SiO_x-np interface provides the necessary index contrast, whereas the SiO_x-np/Si interface does not.'

In general, this manuscript could be reconsidered if the following two things are further conducted: 1) Efficiency is certified by a third-party authority; 2) The scientific findings are reorganized to provide in-depth insights into why this work holds significant scientific importance for triple-junction perovskite/silicon tandem solar cells.

We thank the referee for their constructive comments and for outlining a clear path toward reconsideration of the manuscript.

Regarding certification, since the initial submission, we have obtained an independent performance certificate from the Shanghai Institute of Microsystem and Information Technology (SIMIT) with efficiency above 30%.

Regarding the scientific presentation, we have substantially reorganized the manuscript to better highlight the scientific importance of the work for perovskite–silicon-based triple-junction solar cells. In particular, the revised manuscript emphasizes the mechanistic role of the 4-HBA additive, supported by new photoluminescence and device experiments, the limitations of thick middle-cell absorbers processed by conventional one-step deposition, and the function of the optical middle reflector. New ToF-SIMS, tr-SPV, fracture energy, and BACE measurements have been added to provide deeper insight into the advantages of the three-step deposition strategy and the resulting improvements in film homogeneity and device performance.

We believe these revisions address the referee's concerns and significantly strengthen the scientific impact of the manuscript.

Reviewer 4

The manuscript entitled "Perovskite-perovskite-silicon triple junction solar cells with improved carrier and photon management," with Ref #: "2025-07-17350", by authors "Artuk et al." is a research article on the demonstration of a monolithic (series-connected) perovskite-perovskite-silicon triple-junction solar cell. The authors address two key challenges, one which is the shortcomings of the open-circuit voltage (Voc) of the wide-bandgap top-cell and the other is the limited current density (due to absorber thickness) in the middle-cell. For tackling the top-cell, the authors use additive engineering via 4-hydroxybenzylamine (4-HBA), an organic compound with the molecular formula C_7H_9NO. They show that 4-HBA improves the perovskite crystallization dynamics and suppresses bulk recombination, which helped achieve impressive open-circuit voltages up to 1.405 V. The authors use many high-level characterization techniques to confirm the benefit of the additive 4-HBA, such as in-situ grazing incidence wide-angle x-ray diffraction (GIWAX), X-ray photoelectron spectroscopy (XPS), Time-of-flight secondary ion mass spectrometry (ToF-SIMs), high-dynamic-range external quantum efficiency (HDR EQE), and more. For the middle-cell, they developed a somewhat complex three-step deposition process in order to achieve a substantially "thick" perovskite absorber based roughly on the Cs0.05FA0.9MA0.05PbI3 formulation. The method to achieve the increased thickness required (1) standard one-step spin-coated antisolvent deposition, (2) thermal co-evaporation of the inorganic halide template of PbI2 and CsI, and (3) final spin-coating of FAI:MAI in isopropanol (including annealing steps). A third innovation that the authors demonstrate is the filling of the textured pyramid valleys with reflective SiOx (given the sharp change in refractive index), which helps with the insufficient absorption in the middle-cell. This idea was used back in µc/a-Si tandem and triple-junction solar cells many years ago to enhance top and middle-cell current density.

The fabrication of perovskite–perovskite–silicon triple-junction solar cells is an exceptionally challenging endeavour, demanding not only a high-level of technical complexity in terms of materials processing and device integration, but also substantial investment of time, resources, and experimental precision to achieve reproducible and high-performance devices. The authors produce an impressive record efficiency for the perovskite-perovskite-silicon triple-junction solar cell of 30.5%. The current record is actually 28.7% published only recently by Xu et al. in Nature Materials on the 23 September 2025 (see Xu et al., Stabilized perovskite phases enabling efficient perovskite/perovskite/silicon triple-junction solar cells. Nat. Mater., 1–8, 2025. doi: 10.1038/s41563-025-02367-8). The device demonstrated by the authors here shows a notable efficiency improvement of 1.8%, achieved by effectively addressing key challenges through the three innovations outlined above. These key innovations would be very welcome and of great interest and timely to the perovskite-based multi-junction solar cell field, which has seen a surge in popularity given the potential for industrial mass-scale commercialization.

We sincerely thank the reviewer for providing such detailed and constructive suggestions to enhance the quality of the manuscript, including very relevant and up-to-date references.

Overall, the manuscript is suitable for publication in "Nature", after some key revisions. Please find the detailed review below:

Main Concerns:

1. One of the key concerns is with section on "Hybrid on one-step (three-step) absorbers". The authors have not made it clear why they could not create a thicker middle-cell absorber with simply the one-step antisolvent or gas-quenching techniques and why they have to resort to such a complex three-step deposition process? The authors simply mention in Supplementary Note 1, that "Initial attempts to increase the absorber thickness by adjusting the solution molarity and spin-coating

parameters resulted in delamination of the absorber upon annealing, as shown in Fig. S22. This issue arose due to increased mechanical stress and weak adhesion between the perovskite and the hydrophobic Me-4PACz hole transport layer (22).” The authors should first provide some context and discussion as to why thick perovskite films >1µm, such as FAPbI3, cannot simply be deposited by a one-step process. Is there a primarily limitation with the deposition of thicker perovskite films using one-step techniques? For example, is it because of solubility limitations or is it because of issues with crystallisation, film morphology or phase issues? Are there any relevant examples of this in the literature?

Why thick one-step absorbers fail: mechanical and ionic constraints

Initial attempts to increase the one-step absorber thickness by raising the precursor molarity to 1.8 M (no solubility problem is observed for such a molarity) or modifying the spin-coating parameters led to film delamination upon annealing (**Fig.S26**). This behavior is attributed to increased mechanical stress between the perovskite and the hydrophobic Me-4PACz hole transport layer (*3*). Cross-sectional and top-view SEM confirmed that thicker one-step films developed wrinkled and rough morphologies, features commonly associated with stress-relief mechanisms in thick perovskite layers (see **Fig.S27-32**).

Double cantilever beam fracture measurements show an increased fracture energy (G_c) for thicker one-step films deposited on Me-4PACz (**Fig.S33**), as the film thickness increases from 600 nm to 1000 nm. Under otherwise identical deposition conditions, such a thickness increase is expected to raise the driving force for delamination (G) by a factor of (1000/600) or ~67% based on the fact that G scales linearly with film thickness. However, G_c increases by only ~50%, from 0.4 to 0.6 $J.m^{-2}$, which is insufficient to compensate for the increased mechanical driving force. This imbalance provides a plausible explanation for the delamination observed in the thicker films (Fig.S26) for fracture tests of samples containing Me-4PACz, where the perovskite absorber completely delaminated from the Si substrate, indicating that the Me-4PACz/perovskite interface was the weakest layer in the device stack.

Alternative SAMs, including 2PACz, 4PADCB, and 4PABCz, partially mitigated delamination but did not recover device performance. For devices based on 4PADCB or 4PABCz, absorber thickening beyond 600 nm to ~1000 nm led to pronounced hysteresis and degraded performance without any net gain in J_{SC} (**Fig.S34**). SEM analysis confirmed that these thick one-step films still developed wrinkled and rough morphologies despite retaining large, columnar grains (Fig. S29-31). Such morphological degradation is known to exacerbate ionic transport and recombination losses (4-5), offsetting the potential photocurrent gains from increased optical absorption. Similar observations have been reported for thick anti-solvent–processed middle cells in perovskite triple-junction devices ((Hu et al., *Energy Environ. Sci.* 17, 2800–2814 (2024)).

Finally, there is relevant literature on triple-junction devices employing thick $FAPbI_3$ middle cells (Hu et al., *Energy Environ. Sci.* 17, 2800–2814 (2024)), where gas quenching is used to overcome the limitations of thick one-step, antisolvent-processed absorbers. Consistent with their findings, we also observe the formation of a wrinkled morphology under such conditions. In contrast, our approach not only avoids wrinkle formation but also enables simultaneous bandgap reduction, offering a fundamentally different solution to the challenge of thick perovskite absorber fabrication. We added the citation to this important paper.

We have modified Supplementary Note 1 and the main text accordingly to better establish the motivation behind the development of three-step absorbers.

2. It is opportune that the three-step technique the authors propose, with the co-evaporation followed by sequential solution processing of the FAI:MAI in isopropanol do not remove the underlying one-

step deposited perovskite. However, there are still a few questions to be asked here. Especially if this is the first time this three-step technique has been demonstrated by the authors. For example, what does the homogeneity of the middle-cell perovskite look like? Some cross-sectional SEM with EDX/EDS and/or ToF-SIMs analysis across this layer would be valuable here (single-junctions or simply layers on substrates is sufficient). Does the three-step process end up producing some sort of graded bandgap perovskite absorber (see Ergen et al., Graded bandgap perovskite solar cells. Nat. Mater., 16, 522–525, 2017. doi: 10.1038/nmat4795)? Furthermore, could there be a limitation with the charge extraction given limited carrier diffusion lengths in the actual perovskite absorber layer going to thicker films?

We thank the reviewer for raising this important point regarding the three-step deposited absorbers, which represents our first demonstration of this approach. The deposition of the second absorber employs solvents orthogonal to the first layer (e.g., IPA), enabling damage-free processing. The homogeneity of the three-step absorber depends on the degree of conversion of the second, hybrid-deposited layer. In early developments, under-converted films with insufficient organohalide supply (see Fig.R4) exhibited residual PbI_2 between layers, leading to inhomogeneity. However, optimizing the conversion eliminated residual PbI_2 and resulted in a homogeneous microstructure (see Fig.R4).

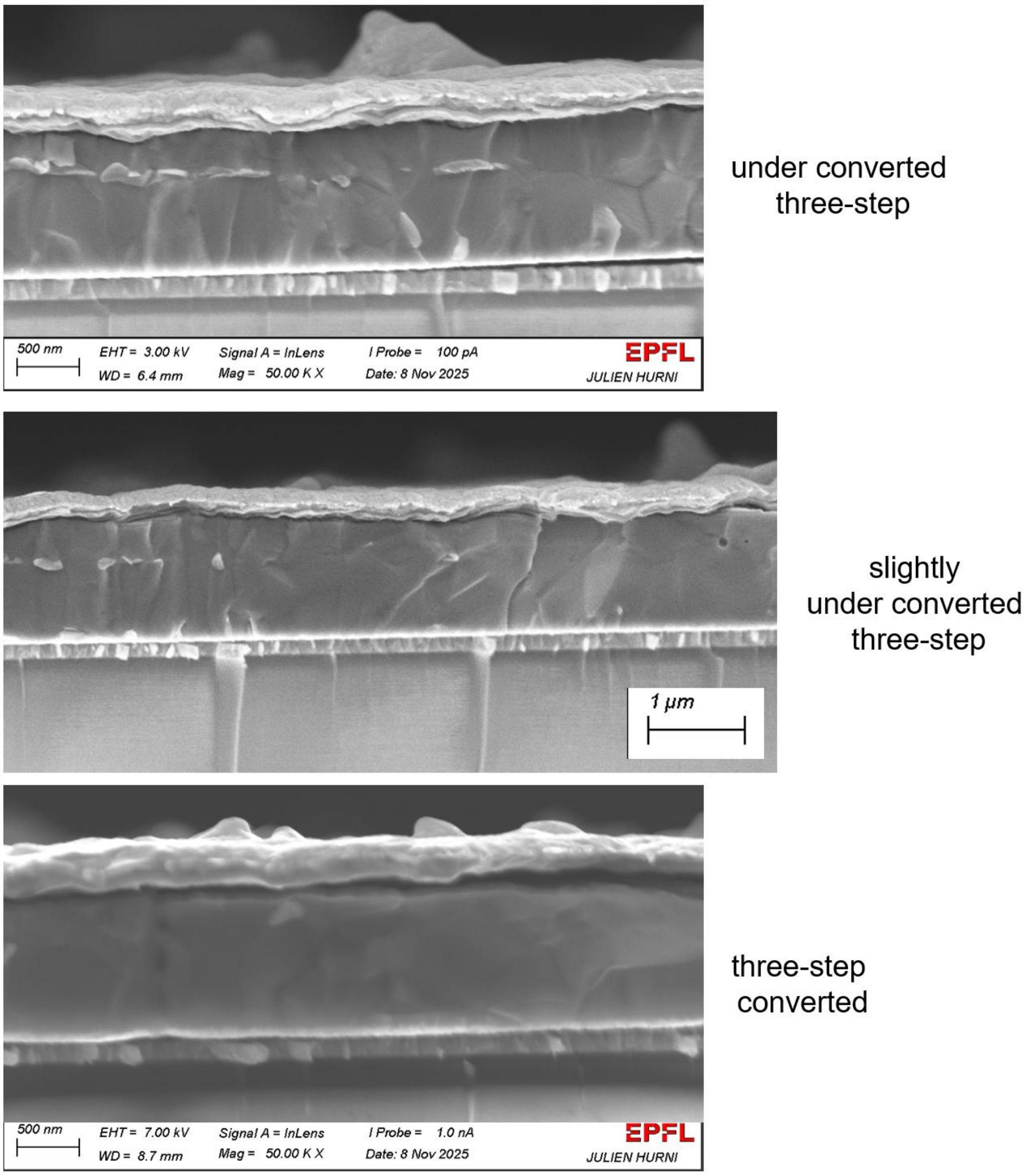

Figure R4. Optimization of the three-step absorbers, cross-section SEM image to identify conversion and PbI2-residuals.

To further confirm this homogeneity, and following the reviewer's suggestion, we performed cross-sectional EDX analyses. However, the resolution of cross-sectional SEM-EDX is insufficient to draw firm conclusions regarding compositional uniformity across the film thickness (see Fig. R5, where residual PbI_2 cannot be reliably identified). We refrain from referencing the work by Ergen et al., which was retracted for lack of reproducibility.

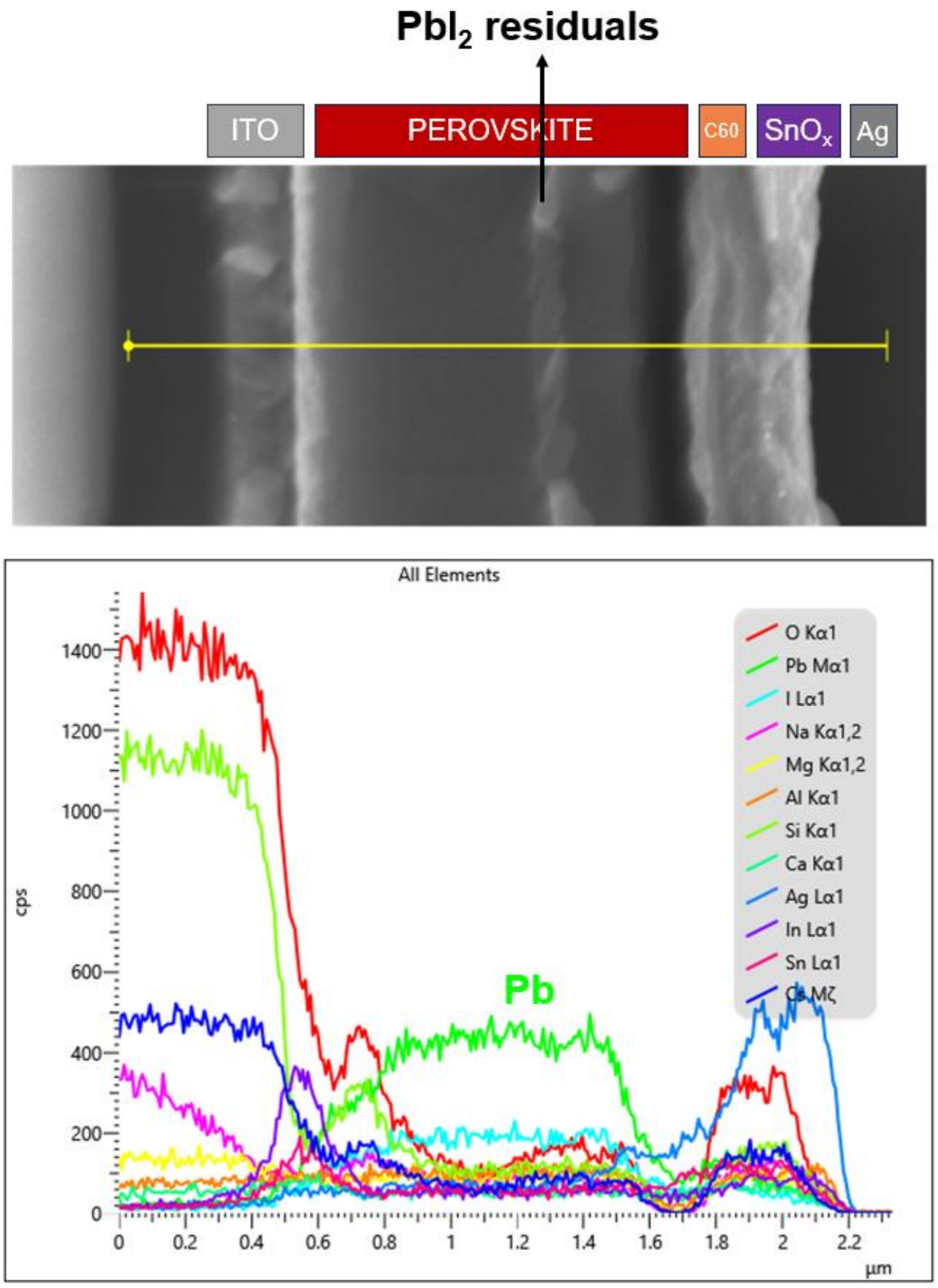

Figure R5. Cross-sectional SEM-EDX on underconverted three-step absorber with PbI2-residuals.

Second, ToF-SIMS measurements are performed on four different samples (one-step thin, one-step thick, three-step underconverted, three-step converted). ToF-SIMS depth profiling reveals that the key components (Cs^+, Pb^+, I^+, FA^+, and MA^+) are uniformly distributed throughout the perovskite absorber layers for both the one-step and three-step deposition routes (Fig.R6, 7 or Fig.S38, 39, 40). This consistent elemental homogeneity indicates that the three-step process does not introduce any vertical compositional grading or graded junction. The PbI^- fragment signal provides clear insight into the incomplete conversion observed in the under-converted three-step films. Specifically, these films show a pronounced PbI^- accumulation near the top surface (see Fig.R7 for linear scale comparison), corresponding to localized excess PbI_2 and consistent with the discontinuous PbI_2 residuals identified in the cross-section SEM (Fig. 2(c) and Fig. S40). Once the three-step conversion is optimized (0.3 M organohalide), this PbI^- enrichment disappears, and the PbI^- distribution becomes uniform across the thickness, in agreement with the SEM evidence of a fully intermixed, continuous absorber layer. Together, these TOF-SIMS results corroborate the structural observations and confirm that the optimized three-step process yields a homogeneous perovskite absorber without vertical composition gradients, whereas under-converted films exhibit localized Pb-I-rich regions confined to the upper part of the layer.

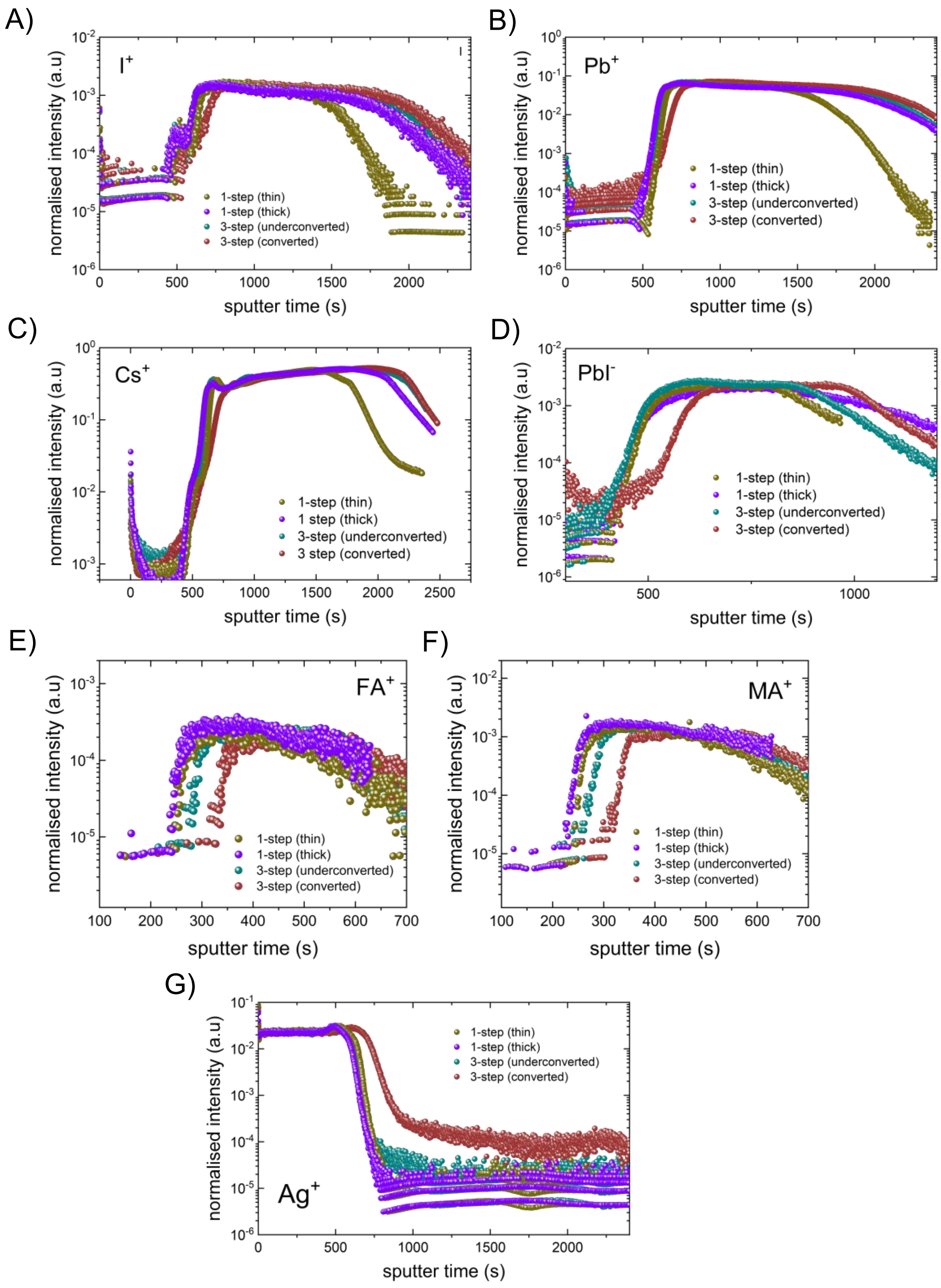

Figure R6.ToF-SIMS depth profiling of four different solar cells with four different absorbers. Due to the thicker Ag thin films on the 3-step converted device, the signals in that sample are shifted in the x-axis. Except the underconverted 3-step sample that shows a gradient in the PbI- signal from top and to bottom of the film, the other signals show relatively homogenous distribution across the thickness.

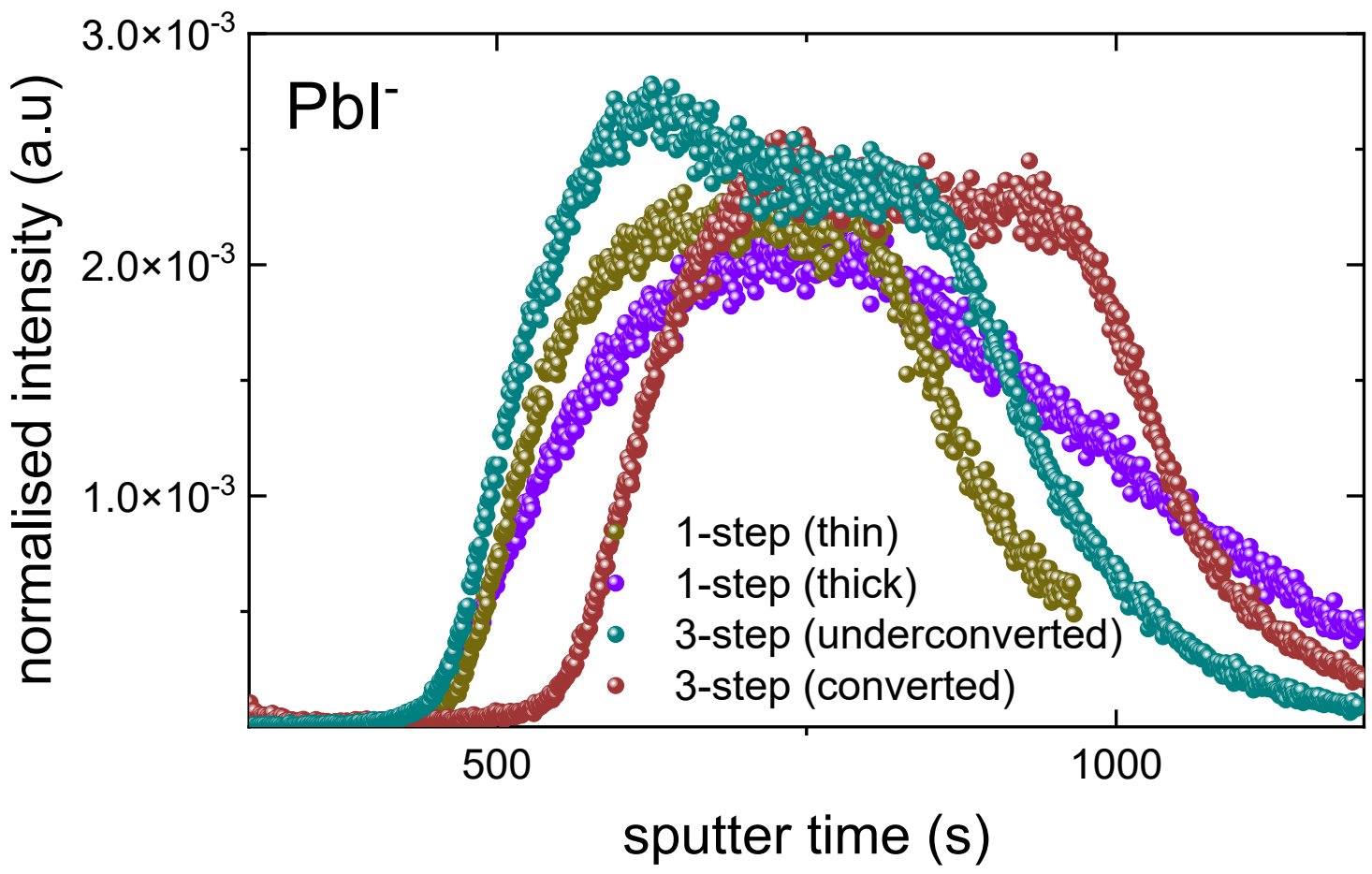

Figure R7. Linear scale PbI- peak to illustrate the differences. Investigation of other signals in linear scale did not alter any differentiable trend between samples.

In the thick one-step films, the reverse-scan series resistance (Fig.S34, extracted at V_{OC}) remains comparable to that of the thin one-step counterparts, indicating similar charge-transport characteristics across the device. This suggests that carrier mobility in these absorbers remains sufficient to prevent additional transport losses even as the thickness increases up to 1000 nm. Moreover, owing to the relatively high built-in voltage in these devices, the mobility requirements are less stringent, consistent with recent findings on perovskite transport dynamics (Gutierrez-Partida et al., *ACS Appl. Mater. Interfaces* **17**, 11176–11186 (2025)).

Tr-SPV measurements show that 3-step deposition improves electron extraction but slightly reduces hole extraction (see Fig.S37). This reduction is most likely due to the increased film thickness, which forces holes to diffuse longer distances before reaching the hole-selective interface. Two opposing mechanisms emerge: (1) increased carrier density and enhanced hole extraction at the C_{60} interface due to the higher thickness, and (2) reduced hole extraction caused by the longer diffusion path. Given that carrier lifetimes are long relative to extraction times (in our previous work (*Nat Commun* **16**, 8672 (2025)), the 1.65 eV absorber with similar PLQYs (2-3% PLQY) to the MBG absorber utilized here achieved lifetimes of 5.3 µs, which corresponds to ca. 3.7 µm diffusion length if we take mobility of as 1 cm^2/Vs following Musiienko et al., Nat Commun 15, 316 (2024)), the second effect is expected to be less detrimental. We also exclude alternative explanations such as the formation of specific hole traps (Musiienko et al., Nat. Commun. 15, 316 (2024)) or differing electron and hole diffusion lengths, because the PL intensity increases and the SPV transient shape remains essentially unchanged, with no accelerated decay.

We hypothesize that the thick one-step films experience enhanced ionic losses arising from microstructural deformation, manifested as a wrinkled and rough morphology (see BACE measurements for mobile ionic density quantification, Fig.S36). These increased ionic losses counteract the expected photocurrent gains from greater film thickness, leading to comparable JSC values (see Fig.S34) and more pronounced hysteresis. In contrast, the three-step films retain microstructural integrity with smooth surfaces, enabling efficient charge extraction and suppressing ionic losses. As a result, they maintain transport properties comparable to thinner films, sustaining high fill factors and delivering higher current densities.

We included new ToF-SIMS (Fig.S38, S39, S40), tr-SPV (Fig.S37), and BACE (Fig.S36) measurements in the revised manuscript to establish the motivation for, and advantages of, the three-step method, as well as to reveal the homogeneity of the resulting films.

3. The authors should provide some discussion (including references) with the use of sub-micron textured pyramids. There have been many publications in the literature for the use of such the sub-micron textured pyramids to be compatible with solution-processed perovskites. It would seem highly appropriate here given that current-matching due to the limitations of the middle-cell thickness is a key aspect in this work. How does the sub-micron textured pyramid heights and the way it reflects/absorbs the incoming light differ to that for standard industrial heterojunction silicon solar cells with ~5 µm features (for example, see Chen et al., Blade-Coated Perovskites on Textured Silicon for 26%-Efficient Monolithic Perovskite/Silicon Tandem Solar Cells. Joule, 4(4), 850–864, 2020. doi: 10.1016/j.joule.2020.01.008 and Li et al., Nanoscale Size Control of Si Pyramid Texture for Perovskite/Si Tandem Solar Cells Enabling Solution-Based Perovskite Top-Cell Fabrication and Improved Si Bottom-Cell Response. Adv. Mater. Interfaces, 10(35), 2300504, 2023. doi: 10.1002/admi.202300504)?

We thank the reviewer for raising this important point regarding the front texturing. Over the past several years, we have systematically investigated multiple surface-texturing configurations compatible with solution processing. These include:

1. **Double-sided textured (DST)** surfaces featuring pyramid sizes up to approximately 2.5 µm, as reported in Turkay et al., *ACS Energy Lett.* 10, 1397–1403 (2025).
2. **Double-sided submicron textured (DSNT)** surfaces with pyramid sizes below 1 µm, as demonstrated in *ACS Energy Lett.* 10, 1397–1403 (2025) and *Nat. Commun.* 16, 8672 (2025).

In this work, we first compared the optical and electrical performance of single-junction SHJ devices fabricated on DST and DSNT substrates (see EQE and JV characteristics for 4 cm^2 devices below). The results indicate that the differences in optical response and overall device performance are marginal.

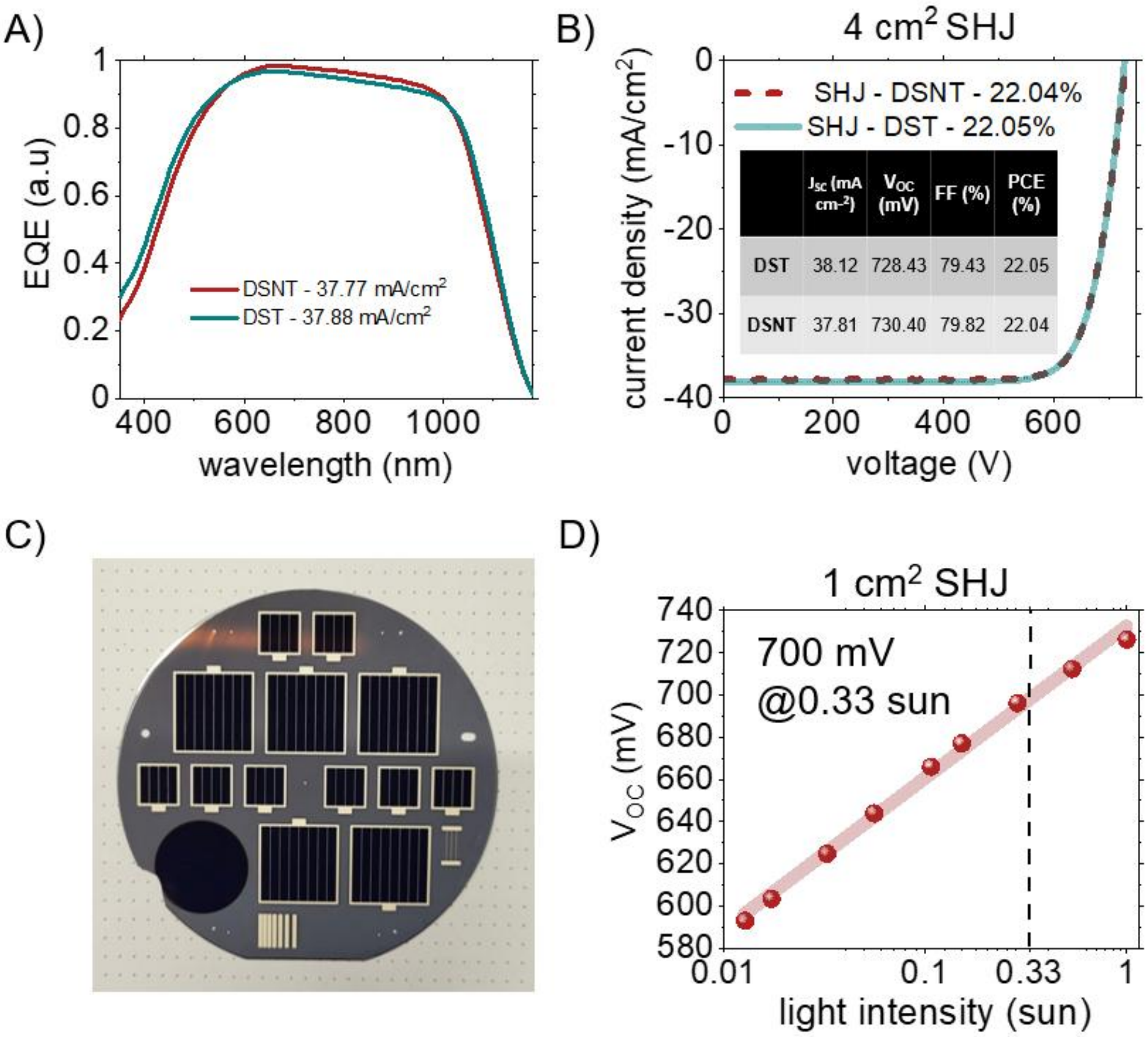

Figure R8. Single-junction SHJ Silicon solar cell performance comparison of double-side textured and double-side nanotextured configurations.

Subsequently, we evaluated the optical performance of DST and DSNT substrates in perovskite–silicon tandem architectures. The bottom-cell EQE response differed by only about 0.2 mA cm⁻², a negligible variation considering that the bottom cell in triple-junction configurations operates with an excess current exceeding 2 mA cm⁻².

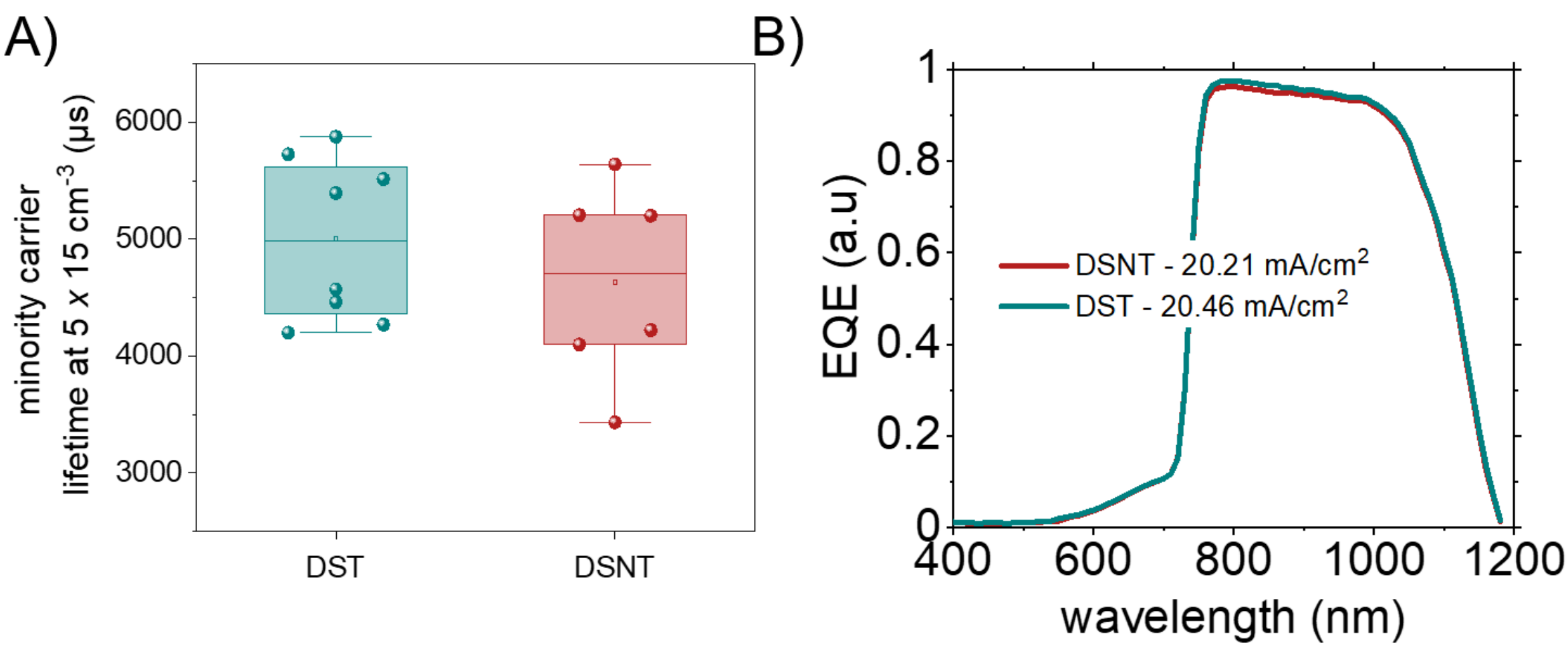

Figure R9. Minority carrier lifetime and optical performance of bottom-cells with DST or DSNT texturing.

It is worth noting that the main motivation for adopting asymmetric texturing, as pursued by LONGi (Liu et al., *Nature* 635, 596–603 (2024).), arises from the differences in passivation quality achieved by their a-Si/nc-Si(p) stack, which performs optimally on larger pyramid textures. However, implementing asymmetric texturing requires multiple additional processing steps: protecting the front side with a thin film (e.g., SiNx), performing rear-side etching, and subsequently removing the protective layer, adding at least three extra fabrication processes.

In contrast, our experiments show comparable passivation quality for both DST and DSNT surfaces (see Fig.R9), making DSNT a more practical and efficient choice. Therefore, we pursue symmetric double-sided submicron texturing (DSNT) in this study, as it offers simpler processing while maintaining equivalent performance.

Moreover, in triple-junctions, we still compared the performance of triple-junction devices employing sub-micron pyramids (average height ≈260 nm) to those using micron-sized pyramids with an average height of ~900 nm, reaching up to 2 μm at their maximum. The latter corresponds to the baseline texturing used for our double-side-textured bottom cells, which differs from the industry standard of ~5 μm. We confirmed the superior performance and yield on sub-micron textures, which we attribute to microstructural differences and possible shunt points associated with large pyramids.

In the revised manuscript, we have added several new references suggested by the reviewer and have reformulated the relevant sections in the main text to clarify the rationale behind the chosen texture configuration and its implications for optical and electronic performance. Moreover, the single-junction Si solar cell results are included in the Fig.S55, S56, and triple-junctions results are included in Fig.S60.

"We adopt a symmetric double-sided sub-micron-textured configuration owing to its simpler fabrication process and competitive device performance compared to the asymmetric design reported by Liu *et al.* (*63*, *64*). In contrast to their observations, the a-Si(i)/a-Si(p) or nc-Si(p) passivation stack in our study provides comparable surface passivation quality on both sub-micron- and micron-scale textured bottom cells, while maintaining comparable optical performance in the bottom-cell (see **Fig. S55, S56**) (*65*). "

"First, we compared the performance of triple-junctions with sub-micron pyramids (average feature size 260 nm) and pyramids with an average feature size of ~1.5 μm (which is the baseline for our double-side textured bottom-cells, unlike the industry standard of ~5 μm) (*66*). After confirming the superior performance and yield on sub-micron textures, which we attribute to microstructural differences and possible shunt points associated with large pyramids (see **Fig. S60**), we compared the performance of the one-step absorber with that of the three-step absorber, as shown in **Fig. S59**."

Other concerns and corrections:

Page 2, Line 62: It is unclear when the authors state: "Perovskite-silicon tandem solar cells have surpassed III-V tandem technology, achieving 34.8% efficiency, approaching the theoretical limit of 42.2% (1, 2)." Is not the record for III-V on silicon 36.1% (triple-junction) and the record for perovskite-silicon 34.85% (dual-junction)? It would be clearer to make the claim that this is for "dual-junction" and find the correct reference here, it seems to be the GaAsP/Si record from OSU/UNSW/SolAero of 23.4% from 2020. Perhaps it would make more sense to simply state that this is the "dual-junction record" with no need to list the 23.4% (III-V researchers probably do not target dual-junction III-V on Si as it is not particularly attractive to do so given efficiencies are higher for triple-junctions.

We thank the reviewer for this suggestion and for raising this point, and we simplified the first paragraph accordingly. We only include the comparison of III-V triple-junctions to perovskite-Si-based triple-junctions and updated the references.

Page 2, Line 71: There is actually a recent newly published efficiency record for triple-junction perovskite-perovskite-silicon solar cells (see Xu et al. Stabilized perovskite phases enabling efficient perovskite/perovskite/silicon triple-junction solar cells. Nat. Mater., 1–8, 2025. doi: 10.1038/s41563-025-02367-8).

We thank the reviewer for this suggestion and included this reference in the introduction.

Page 2, Line 80: For context, the authors could also mention some other ways the field has tried to try to reduce Voc losses in wide-bandgap perovskites especially for perovskite-based multi-junction solar cells. For example, use of Rb+ (Wang et al. Suppressed phase segregation for triple-junction perovskite solar cells. Nature, 618, 74–79, 2023. doi: 10.1038/s41586-023-06006-7 and Zheng et el. Strain-induced rubidium incorporation into wide-bandgap perovskites reduces photovoltage loss. Science, 388(6742), 88–95, 2025. doi: 10.1126/science.adt3417), propane-1,3-diammonium iodide (Wang et al. Halide homogenization for low energy loss in 2-eV-bandgap perovskites and increased efficiency in all-perovskite triple-junction solar cells. Nat. Energy, 9, 70–80, 2024. doi: 10.1038/s41560-023-01406-5), or triple-cation triple halide (Heydarian et al. Minimizing Open-Circuit Voltage Losses in Perovskite/Perovskite/Silicon Triple-Junction Solar Cell with Optimized Top Cell. Solar RRL, 9(3), 2400645, 2025. doi: 10.1002/solr.202400645).

We thank the reviewer for this suggestion and have included these references in the second paragraph and modified accordingly.

In perovskite-based multijunctions, additive and compositional engineering have been demonstrated to address the former challenge (*11–14*). In perovskite-Si-based triple junctions, Liu *et al.* improved the bulk quality of the WBG absorber by incorporating cyanate into the wide-bandgap perovskite lattice, achieving an efficiency of 27.6% and a V_{OC} of 3.13V (*15*). Recently, Xu *et al.* utilized ammonium propionic acid in both medium- and wide-bandgap absorbers to improve their phase stability, enabling an efficiency of 28.7% (*6*). Nonetheless, performance remained constrained by open-circuit voltage of 3.08 V and a middle-cell-limited current density of 11.82 mA.cm^{-2}.

Page 2, Line 89: "Moreover, a middle-reflector composed of low refractive index SiOx-nanoparticles (SiOx-np) accumulating in the valleys of submicron-textured silicon between the middle- and bottom-cell further enhances the middle-cell current density." Did the authors examine the interface between the SiOx and the perovskite with SAM in between? For example, does the interface show or demonstrate an improvement or loss to the ITO in terms of quasi-fermi level splitting (QFLS) analysis?

The SiOx-np/perovskite interface exhibits high quality, as indicated by the absence of any difference in QFLS between glass/perovskite and glass/SiOx-np/perovskite, suggesting similarly high interface quality for both SiOx and SiOx-np. As demonstrated in our previous work (Joule 8, 1965–1978 (2024)), SiOx-np reduces the contact area between ITO/SAM and perovskite. If the ITO/SAM/perovskite interface is of relatively low quality with additional interface losses, reducing this contact area using SiOx-np particles mitigates such losses through localized contacting, a concept similar to PERC. Comparable improvements have been reported for PTAA/AlOx-np in the literature (Science 379, 683–690 (2023)).

In our case, however, the ITO/SAM/MBG perovskite interface is already nearly loss-free, which explains why SiOx-np does not provide additional interface passivation that would typically influence QFLS and VOC. Conversely, reducing the contact area between ITO/SAM and perovskite beyond a certain point introduces resistive losses (see Fig. S52, where FF decreases and Rs increases as SiOx-np concentration rises), consistent with our previous observations (Joule 8, 1965–1978 (2024)). This creates a trade-off between optical gains and resistive losses. However, we note that for triple-junction devices operating at relatively low current density (≈12.5 mA cm^{-2}), compared to tandem devices (≈21 mA cm^{-2}) or single-junction Si devices (≈40 mA cm^{-2}), the impact of resistive losses is significantly smaller.

The situation differs slightly for the WBG absorber, where the ITO/SAM/WBG interface introduces additional losses (see Fig.R10), likely due to imperfect band alignment at the HTL interface. In this case, incorporating SiOx-np reduces non-radiative losses by ≈20 mV (see Fig.10).

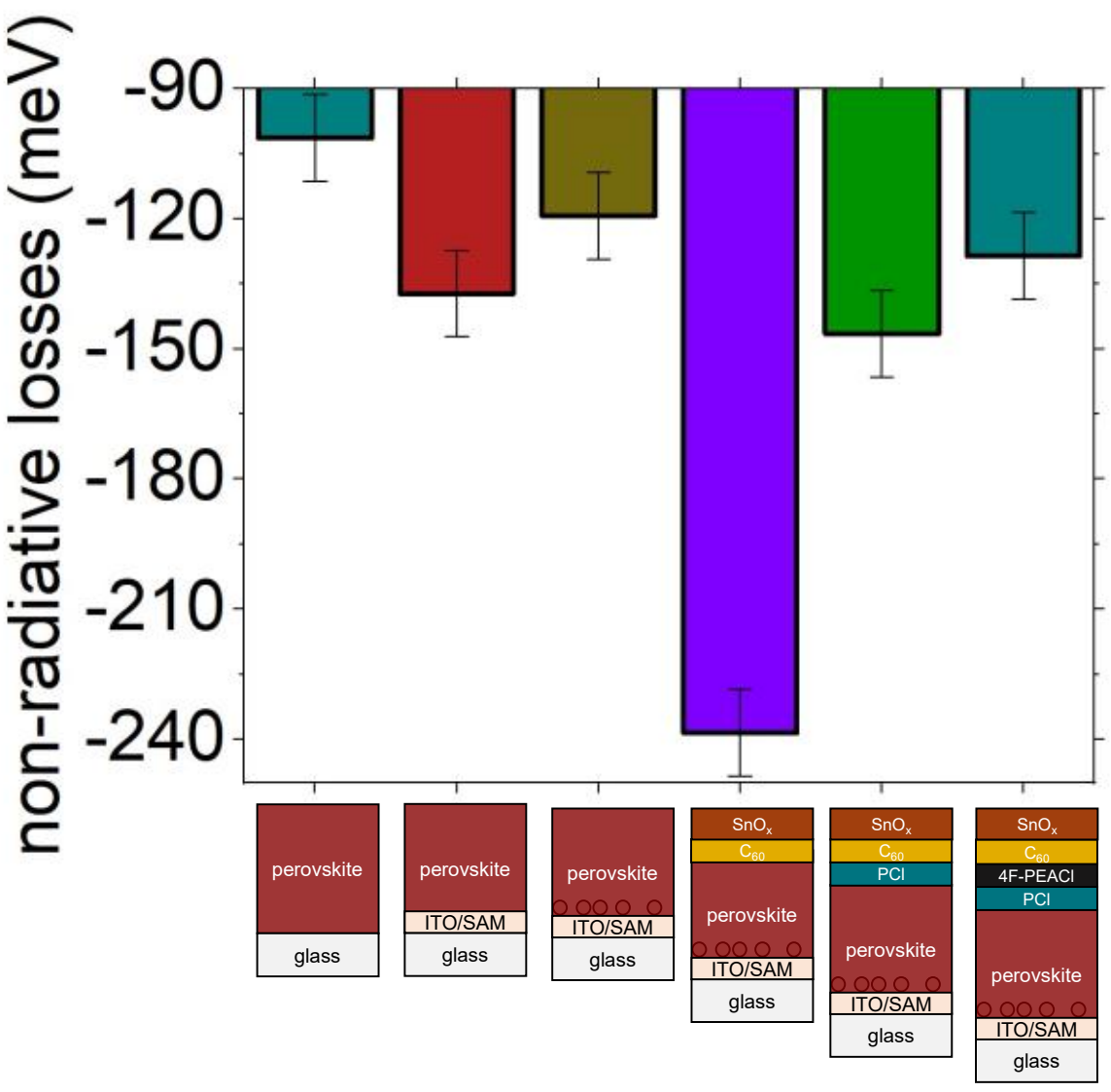

Figure R10. Loss analysis of WBG perovskite solar cells.

Page 3, Line 120: The term ‘stereoisomers’ may not be entirely accurate in this context. 2-HBA, 3-HBA, and 4-HBA are positional (constitutional) isomers, differing by the position of the hydroxyl group on the aromatic ring. Strictly speaking, stereoisomers of hydroxybenzylamine would only arise if chirality were introduced at the benzylic carbon or under particular structural constraints. Perhaps the authors might consider clarifying this wording to avoid potential confusion.

We thank the reviewer for raising this point. Following their suggestion, we have corrected the term ‘stereoisomer’ to ‘positional isomer’ throughout the manuscript.

Pg, 3, Line 122: "…stereoisomers, 4-HBA provided the highest passivation quality, reducing non-radiative losses to ~ 130 meV." Chemically, was there a reason why this is the case between 2-, 3-, and 4-HBA?

Among the three positional isomers of HBA, para-HBA (4-HBA) provides the highest passivation quality, as evidenced by the lowest non-radiative losses (~130 meV). This behavior arises from the distinct spatial arrangement of its hydroxyl functional group, which dictates both molecular orientation and interfacial interactions with the perovskite surface.

In the 4-HBA, the functional groups are positioned symmetrically on opposite ends of the aromatic ring, minimizing intramolecular hydrogen bonding and enabling more ordered coordination with perovskite surface species such as Pb^{2+}, FA^{+}, and Cs^{+}. Although the calculated binding energies of 2-, 3-, and 4-HBA toward these defect sites are relatively similar (highlighting similar chemical passivation capabilities), the film formation kinetics differ markedly among the isomers.

Specifically, the weaker interaction energy of 4-HBA with the $CsPbI_3$ results in slower crystallization dynamics, facilitating enhanced grain growth and a reduced defect density. In contrast, 2- and 3-HBA, which exhibit higher affinities towards the perovskite building blocks (e.g., $CsPbI_3$) and thus faster reaction kinetics, particularly with 2-3-HBA acting as a catalytic species, promote rapid nucleation compared to 4-HBA, and slightly lower bulk quality. Therefore, we hypothesize that the superior bulk quality achieved with 4-HBA compared to 2-3-HBA primarily originates from its moderated film formation kinetics rather than stronger defect binding. Nonetheless, all three positional isomers significantly improve defect passivation relative to the control, underscoring the effectiveness of the underlying defect chemistry.

In the main text, we have included the following sections to address and discuss these points in detail.

"The marginally higher interaction energy of 2-HBA with $CsPbI_3$ may serve a catalytic role, accelerating the reaction kinetics relative to 4-HBA."

"4-HBA results in the slowest process among the three positional isomers, which is then inversely correlated with the adsorption energy calculated from DFT (see **Table S1**) to the perovskite building block, $CsPbI_3$. In fact, the slowest isomer also achieves the lowest non-radiative losses, pointing out the importance of crystallization dynamics to the resultant film quality."

Page 6, Line 230. Have the author quantified the benefit of having reduced surface area between the perovskite and hole transport layer by using the SiOx. Is significant reduction in surface recombination seen as a result? Have the authors reasonably quantified this?

Yes, utilization of SiOx-np at the ITO/SAM/WBG interface, SiOx-np reduces the NR-losses from ca. 140 meV down to 120 meV. Such a 20 meV gain originates from reducing the contact area between the WBG perovskite and the ITO/Me-4PACz. Since there is an offset between the E_{VBM} alignment of WBG perovskite and Me-4PACz, such losses originate (*Adv. Energy Mater.* 13, 2303135 (2023)) (compared to relatively well-aligned MBG perovskite).

Page 6, Line 238. The authors state, "without affecting Jsc". However, from Figure S21, it looks as though the Jsc decreases slightly with the BMP? Is that simply a smaller spread in the samples that were tested? Would "without drastically affecting Jsc" be more suitable here?

We thank the reviewer for raising this point. Indeed, the J_{sc} of the champion devices for BMP and 4-HBA is slightly lower, which we hypothesize originates from the reduced ink molarity caused by the addition of the additive from the stock solution, slightly decreasing the absorber thickness (see ToF-SIMS results – Fig.S18). Accordingly, we have corrected the sentence using the reviewer's suggested wording.

Figure 2A) caption: "... reverse scan JV curves..." otherwise "... reverse scan JV curve of the wide-bandgap..."

We corrected the caption accordingly.

Figure 2C) and Figure 2F) Scale bar values could be shown directly onto the SEM image for better clarity.

We included the scale bar values directly on SEM images to improve clarity.

Page 7, Line 248. The order of the references should be Randi et al. then Lin et al. (17, 31)

We corrected the sentence accordingly.

Page 7, Line 266. Weak or ‘forbidden’ reflections in FA-based perovskites have been shown to originate from subtle octahedral tilting, a structural distortion that is easily overlooked in conventional XRD but crucially suppresses the transition to hexagonal 4H/6H polytypes. Doherty et al. provided clear evidence of this tilt-stabilized noncubic phase, directly linking such faint peaks to enhanced phase stability (see Doherty et al. Stabilized tilted-octahedra halide perovskites inhibit local formation of performance-limiting phases. Science, 374(6575), 1598–1605, 2021. doi: 10.1126/science.abl4890).

We thank the reviewer for this insightful comment and for highlighting the relevance of octahedral tilting in FA-based perovskites. Following this suggestion, we conducted additional grazing-incidence wide-angle X-ray scattering (GIWAXS) measurements and performed one-dimensional integrations, which are now directly compared with conventional XRD data (Fig. S42). These measurements confirm that the weak diffraction feature is present exclusively in the three-step–processed absorber, while it is absent in the control sample. Notably, the control and three-step films also do not exhibit pronounced preferential orientation; in contrast, in highly oriented films, weak or nominally forbidden reflections can be readily overlooked in standard laboratory XRD. Consistent with the findings of Doherty *et al.*, we therefore consider it plausible that this weak reflection arises from subtle octahedral tilting, which stabilizes a noncubic photoactive phase, suppresses the formation of performance-limiting 4H/6H polytypes, and may contribute to the enhanced phase stability of the nearly pure $FAPbI_3$ absorber deposited via the hybrid method. The corresponding discussion has been revised accordingly in the manuscript.

Page 9, Figure 3C). Why have the authors opted to use NaF as the front anti-reflection layer as opposed to the more common MgF2 or LiF?

We utilized NaF due to its slightly lower refractive index (n = 1.32 for NaF vs. 1.38 for MgFx at 550 nm, according to [https://refractiveindex.info/]), which offers marginally better antireflective properties, and its lower evaporation temperature, which improved repeatability during the evaporation process. However, we note that LiF and NaF are more hygroscopic compared to MgFx. During periods of high humidity (>60% RH), NaF forms a ‘skyscraper-like’ microstructure (see Fig.S79). This issue was observed only recently; therefore, we do not recommend using NaF in humid laboratory environments. We have added a note in the Supplementary Information to inform readers of this observation. We will further investigate the light-trapping capabilities of such ‘skyscraper-like’ microstructure.

“We note that NaF, while offering slightly better antireflective properties (due to slightly lower refractive index compared to MgF_x) and improved evaporation repeatability (with evaporation temperatures lower than MgF_x), is highly hygroscopic. Under high humidity conditions (>60% RH),

NaF can form a 'skyscraper-like' microstructure (see Fig. S80), and therefore its use is not recommended in humid lab environments."

Page 9, Figure 3F). Integrated Jsc values for each of the top, middle, and bottom-cells are required here. Nearly all publications in perovskite-based multi-junction solar cells show these values.

Here we included the graphs with integrated EQE for the champion 3J (Fig.R11), which is in line with the measured JV (12.45 mA cm^{-2} from JV and 12.42 mA cm^{-2} from EQE), but in the main text, we prefer not to integrate the EQEs due to the following reasons explained by our colleagues with extensive know-how in multijunction measurements from Fraunhofer ISE by examples from tandems and triple-junctions in detail;

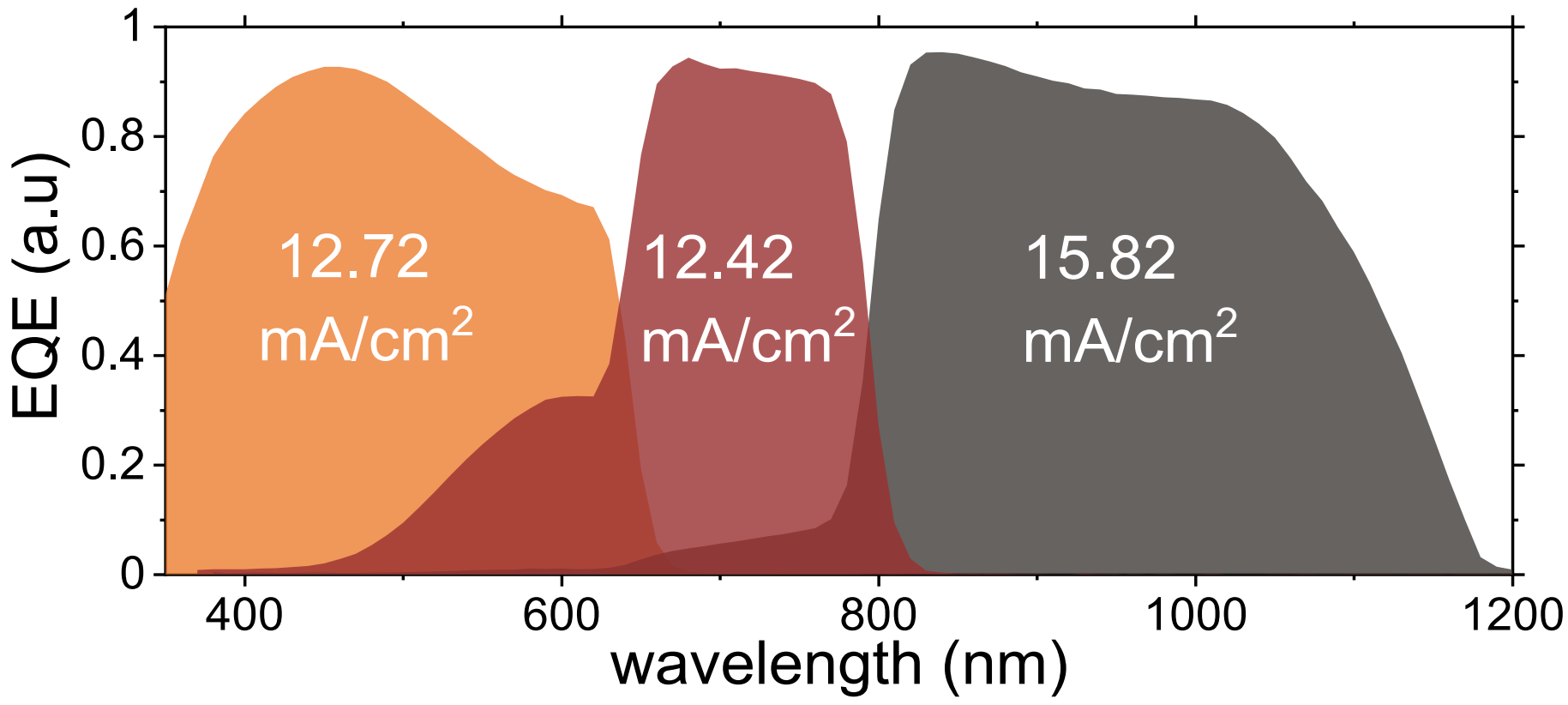

Figure R11. Champion EQE of the triple-junction with integrated current densities.

"Measuring an absolute EQE is already challenging for single junction solar cells (*Appl. Opt.* 26, 1701–1708 (1987)). For multi-junction solar cells, the sub-cell to be measured needs to be brought into short-circuit conditions, which is realized by a bias voltage. However, the sub-cell voltages cannot be determined individually in monolithic tandem devices, the operational point can differ from the actual sub-cell short-circuit condition. This can lead to measurement artifacts and a wrong height of the EQE if the measured sub-cell has a non-ideal behavior, as for example a low parallel resistance, (*Prog. Photovolt. Res. Appl.* 11, 499–514 (2003) which is often the case in perovskite silicon tandem solar cells. Similar effects can come from luminescent coupling effects (*IEEE J. Photovolt.* 3, 879–887 (2013)). These effects are well-known in the III-V community, but unfortunately, often not considered in the perovskite community, so far. So far, we have been discussing tandem solar cells in general. If perovskite is involved, additionally, dynamic effects can influence the EQE. There are several publications from different research groups which show that the height of the EQE of a perovskite solar cell can be dependent on measurement conditions, e.g., bias light intensity (see for examples these publications, *Sol. Energy Mater. Sol. Cells* 172, 66–73 (2017), *Energy Technol.* 10, 2200748 (2022), *Curr. Appl. Phys.* 16, 898–904 (2016)). It has to be noted that for solar simulator adjustment, only relative EQEs are needed (*Prog. Photovolt. Res. Appl.* 10, 243–255 (2002), *Prog. Photovolt. Res. Appl.* 32, 372–389 (2024)). The relative EQE (shape of the EQE) is fortunately, mostly not affected by the measurement conditions. Thus, the measured EQEs can be used to adjust the solar simulator in order to get a reliable IV measurement. The graph in Fig. R12 (presented by Bett et al. at SiliconPV 2022) shows an example of measured EQEs for a perovskite silicon tandem solar cell. The measurement was done twice, with and without white bias illumination in addition to the selective bias light.

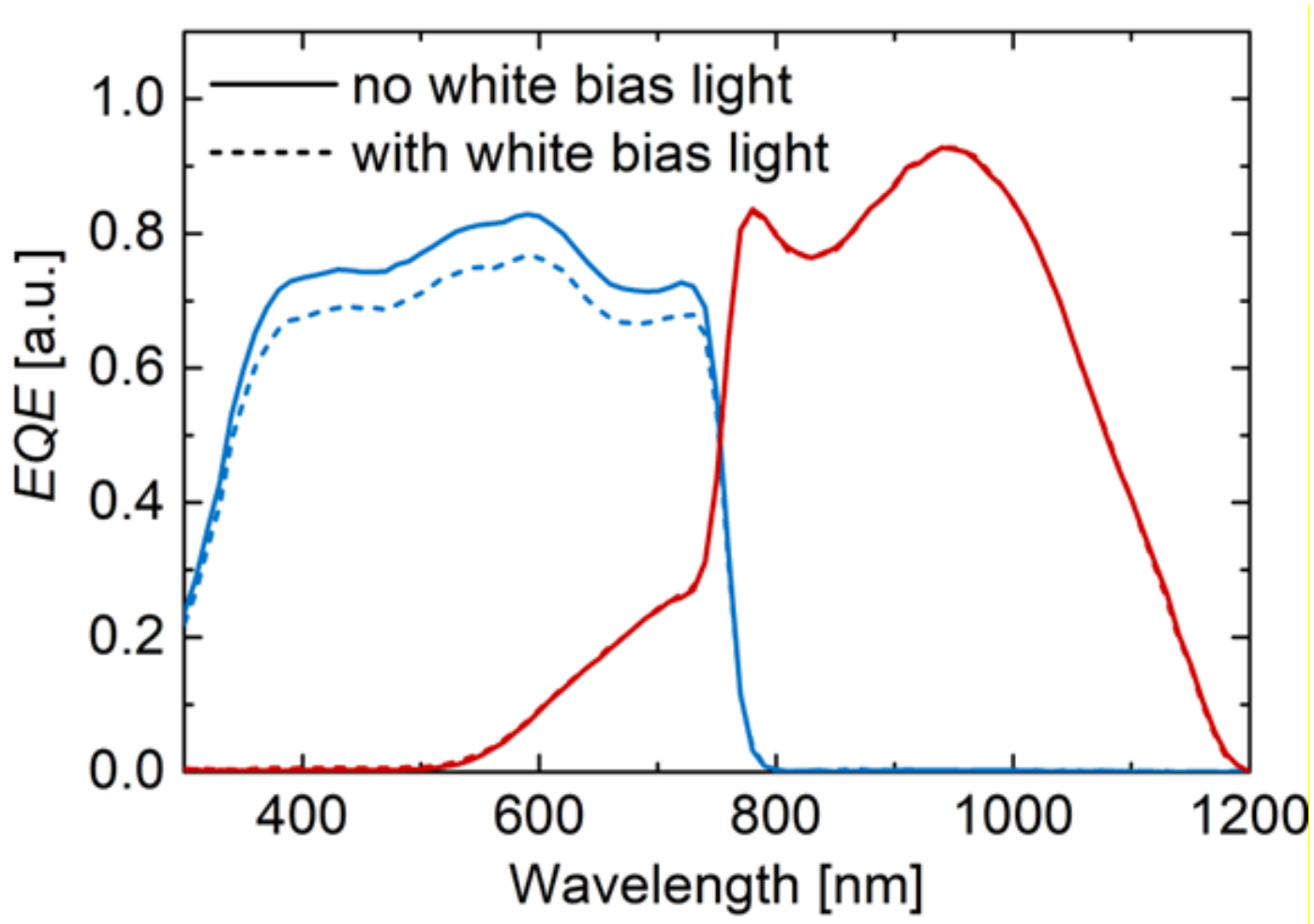

Figure R12. Impact of white bias light on the absolute EQE of a perovskite silicon tandem solar cell.

In this example, the bias light affects the height of the perovskite EQE; the silicon EQE is not affected. Determining the current densities from integrating these EQEs, one could conclude that the perovskite is the limiting sub-cell (considering the measurement with bias light) or that the silicon is the limiting sub-cell (considering the measurement without bias light). Thus, integrated current density values from measured EQEs can lead to a misinterpretation of the current matching situation. The current density value obtained from the perovskite EQEs would differ by more than 7% which shows that a current density value obtained from integrated measured EQEs is by far not accurate. For the reasons discussed above, a measured EQE unfortunately cannot be considered as absolute, and therefore, current density values by integrating measured EQEs cannot be determined. For the same reason, also Fraunhofer ISE Callab is not giving integrated current density values from measured EQEs in their certification reports.

This becomes more complicated in the case of triple-junction solar cells as for measurement of each subcell multiple selective bias lights are used, each of which could have different impact on the EQE signals. An example is shown for the measurement of bottom cell where the blue LED and red LED are applied, and the intensity of blue LED is varied during measurements. For simplicity, the measurement is limited to a single wavelength at 900 nm, where the maximum signal is expected. Increasing the intensity of the blue LED strengthened the signal at 900 nm.

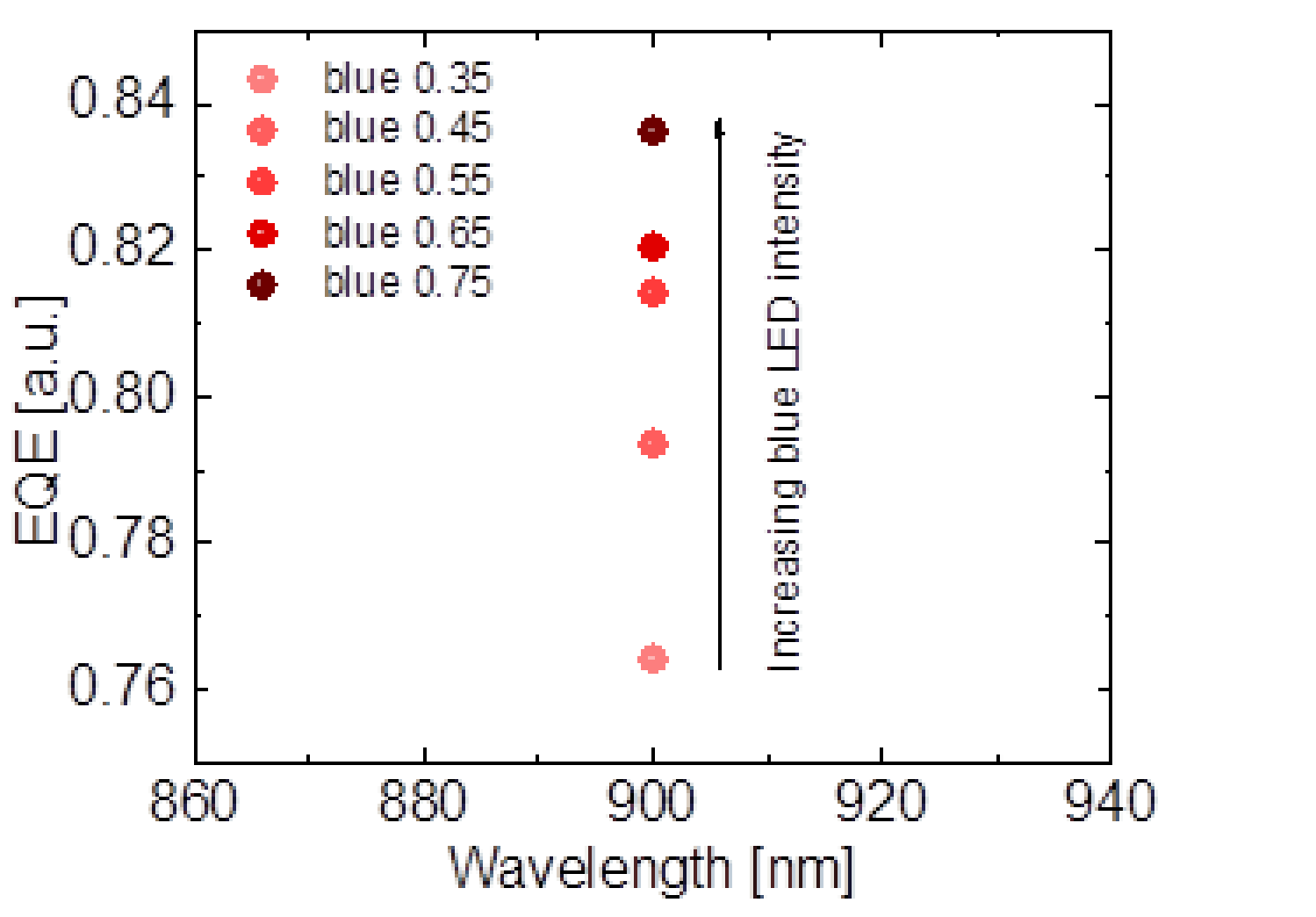

Figure R13. Changes in the middle-cell EQE at 900 nm, by different blue LED intensities.

In addition, in the case of reverse bias breakdown of a subcell, the current of the subcell changes. This is especially important for the measurement of top cells. During the measurement, the top cell becomes

the limiting subcell, if the bias voltage applied during the measurement is not sufficient, it sees a negative voltage, leading to its breakdown (Solar RRL 9, 2400376 (2024)).

For comparison, a III–V/III–V//Si triple-junction solar cell was measured on in-house setup and at Fraunhofer ISE CalLab. The in-house EQE values are higher than those measured at CalLab (Fig.R14). However, the deviation differs among the sub-cells, so a single scaling factor cannot be applied. After normalizing both datasets, the relative EQE (shape) shows excellent agreement between both measurements, which is what is needed for solar-simulator spectral adjustment, as discussed earlier."

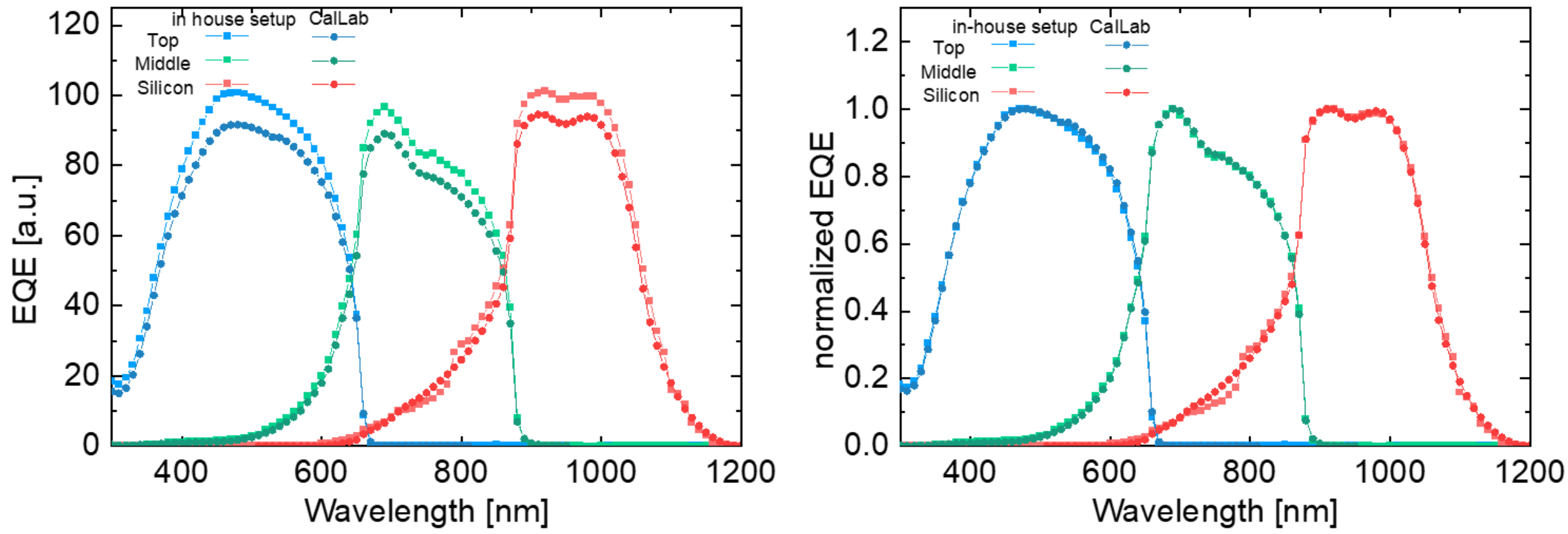

Figure R14. Triple-junction measurement comparison between ISE and CalLab, indicating major differences.

Page 10, Line 356. The authors mention that Si bottom-cell performs on average rather poorly (iVOC = 0.679V) with detrimental low PL areas. Could the authors provide some PV parameters for stand-alone single-junction silicon heterojunction bottom cells that were used to make the triple-junction devices in this work? Perhaps as an additional figure or table in the Supplementary materials? At the very least provide a reference(s) to silicon hetero-junction solar cells made in the past by the same research institute. Higher Voc can be expected not only from reduced processing steps and handling challenges highlighted by the authors, but also from the increased light capture that results when the two additional middle- and top-cell perovskite absorbers are absent in single-junctions. Having single-junction silicon heterojunction solar cell data used in this work would provide a strong basis for comparison and understanding of upper device efficiency limits.

We thank the reviewer for raising this important point. The iVOC was measured on an actual 2.5 × 2.5 cm² triple-junction device using a 1 cm² shadow mask, consistent with the JV measurements. However, silicon, due to its long carrier diffusion lengths, is susceptible to downsizing effects, particularly because unpassivated edges can introduce perimeter recombination. At our institute, 1 cm² SHJ solar cells fabricated on 4-inch substrates (with edge passivation) typically exhibit an average VOC of 725 mV (see Fig.R8) on 1-inch substrates (with unpassivated edges) up to 720 mV, whereas 4 cm² cells fabricated on 4-inch wafers with edge passivation achieve VOC values up to 730 mV under 1-sun illumination.

In response to the reviewer's suggestion, we have included the JV performance data of 1 cm² single-junction SHJ cells measured under various illumination intensities. At approximately 0.33 sun, the VOC of these devices decreases to around 700 mV, which remains about 20 mV higher than the iVOC value obtained from mapping (680 mV). We attribute this difference to partial degradation of surface passivation, likely induced during device processing. The impact of handling-induced damages has been previously investigated and reported (Joule 8, 1234–1248 (2024)).

These data, along with an additional figure and explanatory note, have been added to the Supplementary Information. We believe this provides a solid basis for comparison and helps contextualize the efficiency limits observed in the triple-junction devices.

Supplementary Information, page 3: "…SiOx-np solution in Ethanol (for WBG) and 0.1% SiOx-np for MBG in EtOH (20 nm diameter)…" Is Ethanol not the same as EtOH?

We have corrected the terminology and now use 'Ethanol' consistently throughout the manuscript.

Supplementary Information, page 3: "For the BMP in WBG, 0.4 mg/mL…," this should read "For the bimolecular passivation (BMP) in WBG, 0.4 mg/mL…"

We have improved the readability of the sentence in accordance with the reviewer's suggestion.

Supplementary Information, page 4: On the description of the double-side nanotextured devices, are both the front-side and rear-side the same texturing process? That is, are they both the same sub-micron textured pyramids? If so, why did the authors not consider the standard texturing process (~5 µm) for the rear side and sub-micron texturing for the front side of the silicon heterojunction solar cell? For example, see Jia et al. Efficient perovskite/silicon tandem with asymmetric self-assembly molecule. Nature, 644, 912–919, 2025. doi: 10.1038/s41586-025-09333-z.

We utilize double-sided sub-micron (nanotextured) symmetric texturing. While asymmetric texturing is an option, it requires significant additional processing steps (e.g., SiNx protection deposition on the front side, second etching of the rear side, and subsequent SiNx removal from the front side). One reason LONGi employs asymmetric texturing is to improve passivation quality, as their nc-Si(p) layer performs better on larger pyramids (Liu et al., *Nature* 635, 596–603 (2024)).

In our case, we do not observe any difference in lifetimes between double-side-textured cells (>1.5 µm pyramids) and double-side-nanotextured bottom cells (see lifetime comparison), while the DST configuration provides minor optical improvements (Fig.R8).

Supplementary Information, page 4: "…is deposited at 200°C (45. …" ???

Here, one of the parentheses of reference 45 was missing; we corrected it.

Supplementary Information, page 5: What does CsFA-TH stand for? Could the authors please provide the full term in the unabbreviated form?

We updated the abbreviation to WBG perovskite absorber instead of CsFA-TH (CsFA-based triple-halide absorber).

Supplementary Information, page 5: "…coordinated via three shared I-bonds)." Seems to be a hanging ")" there.

Corrected.

Supplementary Information, page 6: "1.000 W/m2" to "1,000 W/m2"

Corrected.

Supplementary Information, page 6: Isn't ~0.50 V/s very fast for a typical scan rate? Why have the author chosen this scan rate? Later on in the paragraph, the scan rate for the ~1 cm2 is 0.1 V/s. Why the variation here?

We thank the reviewer for raising these points. For devices with three pixels on one substrate, we observe relatively metastable effects on pixels that remain under illumination and open-circuit while other pixels are being measured. Similar observations have been reported in the literature and are likely related to interface passivation layers such as PI or PCl (*Adv. Energy Mater.* **15**, 2404726 (2025)). To minimize the influence of these transient effects, we perform relatively fast JV scans for single-junction WBG devices.

Regarding hysteresis, we observe negligible differences between scan rates of 0.5 V/s and 0.1 V/s. For larger devices, where each device contains only one pixel, this issue does not occur; therefore, we use slower scan rates to approach steady-state conditions. For champion cells, we report the stabilized maximum power point tracking, averaging over 300 s, which we identify as the steady-state efficiency. We believe this represents the most reliable approach.

Supplementary Information, page 6: "Pv-tools" should be "PV-tools"

Corrected.

Supplementary Information, page 7: "… June 3, 2025. sGlobal horizontal…" Please correct.

Corrected.

Supplementary Information, page 9: "linkam" should be capitalized, "Linkam".

Corrected.

Supplementary Information, page 21: For Figure S13, is there a colour or symbol differentiation between the C-O and O-H?

Corrected.

Supplementary Information, page 25. For Figure S18-S19, it is not clear what the difference is between the samples simply with reference to the names 32R, 72R, 42HBA, and 92HBA. There does not seem to be an explanation anywhere in the text.

Corrected.

Overall, this manuscript presents high-quality research that makes a timely and substantial contribution to the rapidly advancing field of perovskite-based multi-junction solar cells. The authors report a record-efficiency perovskite–perovskite–silicon triple-junction device by addressing wide-bandgap Voc losses, middle-cell thickness limitations, and optical management through filling of SiOx in textured interfaces. The work demonstrates both originality and technical innovation, and its findings would be of interest to the readership of Nature.

Regarding certification, while independent testing is normally expected for record devices, the authors reasonably justify its absence. They note that two leading institutes could not certify triple-junctions and a third had a long delay, and instead worked with Fraunhofer ISE to validate results against EPFL using an LED-based simulator. This provides a credible basis for confidence in the reported metrics.

Some aspects, however, need further strengthening before publication. The three-step deposition method for the middle-cell absorber should be studied more carefully through cross-sectional characterization (e.g., SEM with EDX/EDS or ToF-SIMS) to confirm uniformity in thick perovskite films. Further discussion is also needed on the limitations of one-step deposition for >1 μm absorbers and on the role of sub-micron nano-textures compared to standard micron-scale silicon texturing, to

place the work more firmly within the broader literature.

We thank the referee for their positive and constructive assessment of our work, for recognizing the significance of the reported record-efficiency perovskite-perovskite-silicon triple-junction devices, and for acknowledging the substantial technical challenges associated with their fabrication.

First, we expanded the discussion and experimental evidence explaining the limitations of thick one-step perovskite absorbers by including cross-sectional SEM, time-resolved surface photovoltage (tr-SPV), fracture-energy, and bias-assisted charge-extraction (BACE) measurements. These results elucidate the challenges associated with microstructural deformation, mobile ion density, and mechanical instability.

Second, in response to the referee's request for deeper insight into the three-step deposition method, we added new cross-sectional SEM and ToF-SIMS analyses to directly assess compositional uniformity across the middle-cell absorber, confirming the homogeneity achieved with this approach. Complementary tr-SPV, BACE, and fracture-energy measurements further substantiate the optoelectronic and mechanical advantages of the three-step process.

In addition, we clarified the role of sub-micron nano-textures relative to conventional micron-scale silicon texturing, placing the optical management strategy more clearly within the context of the existing literature. Finally, to further strengthen the relevance and robustness of the work, we included new wafer-scale results demonstrating fully fabricated 4 cm^2 and 54 cm^2 devices on 4-inch wafers with industrially relevant bottom-cells using screen-printed metallization, achieving efficiencies exceeding 27 and 26% respectively.

We believe these additions substantially strengthen the manuscript and fully address the referee's concerns.

Referee #2 (Remarks to the Author):

The authors have provided a comprehensive response to the previous comments and have supplemented the study with independent certification reports from SIMIT. However, as previously noted in comments #1, #3, and #4, the use of the 4-hydroxybenzylamine additive, the three-step deposition strategy, and the SiOx nanoparticle reflector are well-documented in the existing literature. Therefore, referring to these as 'innovations' in the manuscript abstract is inaccurate. It appears that the current work is largely a composite of established methods; while the improvement in efficiency for the perovskite-perovskite-silicon triple-junction solar cells is notable, the revised manuscript still lacks new scientific insights. Frankly, the work reads more like a technological process report than a scientific study. We reiterate the view that 'too many perovskite papers simply use some additives (whether random or well-known), achieve high efficiency, and then construct a narrative to get published.' Given the lack of novelty and the data issues, we do not recommend the acceptance of this manuscript.

We sincerely thank the referees for the careful reassessment of our revised manuscript and for articulating the remaining concerns with clarity. We appreciate the opportunity to further refine the scientific positioning of our work. Below, we respond point-by-point and clarify how the revised manuscript addresses the issues raised.

Major concerns are as follows:

1. Regarding the responses to comments 1–4#, I must emphasize that although the revised manuscript shows minor differences from prior literature—concerning the additive, the three-step perovskite deposition method, and the SiOx nanoparticle reflector—such marginal variations fail to establish the novelty and scientific significance required for work in the field of triple-junction solar cells.

We thank the reviewer for reiterating this important point. We fully agree that additive engineering, multi-step deposition, and optical reflectors are not new *per se*. Our intention is not to claim the invention of these concepts. Rather, the central contribution of this work lies in demonstrating how these approaches can be synergistically integrated and recontextualized to resolve architecture-specific bottlenecks in monolithic triple-junction devices.

In the revised manuscript, we have removed language that could be interpreted as overstating "innovation" and instead emphasize the following conceptual advances:

1. Bulk crystallization modulation of 4-HBA in high-bromide WBG perovskites, rather than its previously reported surface-reconstruction role.
2. Identification of mechanical and ionic limitations in thick middle-bandgap absorbers, and demonstration that three-step hybrid deposition fundamentally alters adhesion, stress distribution, and carrier dynamics, not merely thickness.
3. Quantified optoelectronic trade-off analysis of a low-index intermediate reflector, explicitly linking refractive-index contrast, series resistance increase, and current redistribution across subcells.

The scientific advance is therefore not the isolated use of each tool, but the mechanistic understanding of how carrier management and photon management must be co-optimized to unlock >30% efficiency in a monolithic perovskite–perovskite–Si triple-junction architecture.

2. There is a clear logical contradiction regarding the scientific problem statement in the revised manuscript. The authors state in the Introduction that 'wide-bandgap (WBG, >1.80 eV) perovskite absorbers suffer from elevated non-radiative losses and require high bromide content, which leads to phase segregation, reducing stability, and increasing non-radiative recombination…' Subsequently, however, they claim that 'In perovskite-based, additive and compositional engineering have been demonstrated to address this (former) challenge.'
If additive engineering has already resolved these challenges, what is the significance of the extensive study on the 4-HBA additive in this work? Furthermore, where is the scientific innovation? These self-contradictory statements are one of the primary reasons I consider the novelty of this manuscript to be insufficient."

We appreciate this observation and recognize that our previous wording may have unintentionally implied that additive engineering had already fully resolved the challenges associated with wide-bandgap perovskites. This was not our intention. We have therefore reformulated the Introduction to clarify that additive and compositional strategies have partially addressed the challenges of WBG absorbers.

While additive and compositional engineering have improved wide-bandgap absorbers, high-bromide (>1.8 eV) compositions required for triple-junction architectures continue to exhibit substantial bulk non-radiative recombination and thermal instability under operating conditions. Importantly, our work does not claim to eliminate halide phase segregation, but rather to reduce its extent. More critically, we demonstrate that bulk recombination, rather than residual phase segregation alone, remains the dominant voltage-limiting pathway in these systems. Suppressing this bulk recombination enables substantial voltage gains even when a measurable degree of phase segregation persists.

Beyond defect suppression, we further show that the incorporation of 4-HBA improves interfacial band alignment at the hole-transport interface, thereby reducing interfacial recombination losses in addition to bulk recombination. This combined control of crystallization dynamics, defect density, and interfacial energetics distinguishes our approach from prior additive reports that primarily focused on surface passivation or compositional tuning. By incorporating 4-HBA as a bulk crystallization modulator, we achieve single-junction Voc values up to 1.405 V and a triple-junction Voc of 3.24 V, exceeding previous reports. We have revised the Introduction accordingly to clarify that additive engineering has not "solved" the wide-bandgap challenge but remains an open and powerful design space, particularly in high-bromide systems where the mechanistic interplay between crystallization kinetics, defect formation, phase stability, and band alignment is not yet fully understood. Our contribution lies in identifying bulk crystallization control, defect suppression, and improved interfacial energetics as the dominant levers for voltage enhancement in perovskite–perovskite–silicon triple-junction architectures.

3. Regarding the analysis of the 4-HBA additive engineering, although the authors attempted in their response to distinguish bulk doping from previously reported surface-phase reconstruction strategies, their exposition of the core passivation mechanism remains confined to the chemical coordination between the amino group and Pb^{2+}. The authors also acknowledge similarities in the mechanistic discussion of chemical passivation with that the previously reported work by De Wolf et al. This study has not sufficiently demonstrated the unique advantages of introducing molecules into the bulk phase.

The innovation is thus confined to the adjustment of "Pb passivation at different sites" rather than representing a breakthrough in understanding the passivation mechanism.

We thank the referee for encouraging us to sharpen the mechanistic distinction. While Pb^{2+} coordination is indeed a central interaction, our findings extend beyond surface chemical passivation.

In our work, 4-HBA is incorporated directly into the precursor ink and acts as a transient crystallization regulator, fundamentally differing from the previous report of 4-HBA. DFT calculations, NMR analysis, and in-situ GIWAXS collectively demonstrate that 4-HBA stabilizes precursor adducts and retards crystallization kinetics. This slower crystallization enables larger domains, preferential out-of-plane orientation, and reduced defect density. Importantly, ToF-SIMS confirms that 4-HBA is distributed throughout the absorber thickness, rather than being confined to interfaces, distinguishing this approach from surface-reconstruction strategies.

Furthermore, by comparing positional isomers of hydroxybenzylamine, we establish a structure–function relationship linking adsorption energetics, crystallization kinetics, and non-radiative losses. This provides a transferable molecular design principle for high-bromide perovskites: moderated crystallization through transient coordination reduces bulk recombination in wide-bandgap absorbers. We have reorganized the mechanistic section to emphasize this broader insight.

4. The application of SiOx nanoparticles on textured silicon substrates has been reported on multiple occasions (Joule, 8, 1735-1753, 2024; Nature Communications, 16, 7331, 2025). Specifically, the Nature Communications study explicitly elucidated the utilization of nanoparticles for optical anti-reflection and light management, which significantly overlaps with the mechanism of action of SiOx in this study. In the revised manuscript, the authors continue to focus primarily on anti-reflection properties, failing to provide novel scientific insights beyond existing understanding.

We acknowledge that SiOx nanoparticle-based light management has been previously reported. Our contribution lies in implementing and quantitatively analyzing this concept within a monolithic perovskite–perovskite–silicon triple-junction architecture, where current matching and series resistance constraints are significantly different than in tandem devices.

In the revised manuscript, we explicitly quantify the trade-offs introduced by the middle reflector (especially via device modeling to precisely estimate the contributions of resistive losses, see Table 2). The enhanced reflectance at the perovskite/SiOx-np interface increases middle-cell absorption near the band edge, while slightly reducing bottom-cell current and increasing series resistance due to reduced contact area. We provide direct measurements and equivalent-circuit analysis demonstrating that the net device efficiency gain arises from a balanced redistribution of photocurrent across subcells.

We have also clarified the optical mechanism to ensure that the refractive-index contrast responsible for reflection is correctly described at the perovskite/SiOx-np interface.

$\approx$ 1.33, consistent with a porous or silica-like medium, whereas the *Nature Communications* study

5. The artificial compilation of key data in the revised manuscript is unacceptable for a journal of Nature's standing. For example, the triple-junction device relies on a 50% Br content (1.9 eV), while the single-junction analysis is limited to 30% Br (1.8 eV). Because the compositions are not identical, the challenges faced by these two bandgaps are not on the same level! It is clear that the authors are

cherry-picking data to fit a predetermined narrative, failing to provide a targeted solution for the top wide-bandgap perovskite in the triple-junction device.

We appreciate this important comment and fully agree that clarity and consistency are essential.

The project was initially conducted using 1.8 eV absorbers to enable systematic mechanistic optimization and isolate interfacial effects. After establishing the mechanistic framework, the bandgap was increased to 1.9 eV to satisfy current-matching requirements for the final triple-junction architecture.

Importantly, the beneficial effects of 4-HBA are not limited to a single composition. We now explicitly demonstrate that:

- The performance enhancement persists at 1.9 eV compositions (which was the optimum bandgap in the champion triple-junction), yielding high V_{OC} values that surpass current literature benchmarks for wide-bandgap perovskites (see Figure R1 or S23 for PLQY measurements of UWBG absorber with and without 4-HBA).
- The additive is effective across a broad compositional window spanning 1.55 eV to 1.9 eV.
- Newly included PLQY data for the 1.9 eV absorber confirm that the non-radiative recombination suppression remains strong at higher bandgap compositions.

This compositional universality, independent of halide ratio and A-site cation formulation, strongly supports that 4-HBA operates through a general interfacial and defect-passivation mechanism rather than a composition-specific effect.

Thus, the 1.8 eV devices establish a mechanistic understanding under controlled conditions, while the 1.9 eV absorbers demonstrate transferability under realistic triple-junction constraints. We believe this clarification resolves the concern regarding data consistency and highlights the broad applicability of 4-HBA across wide-bandgap perovskite systems.

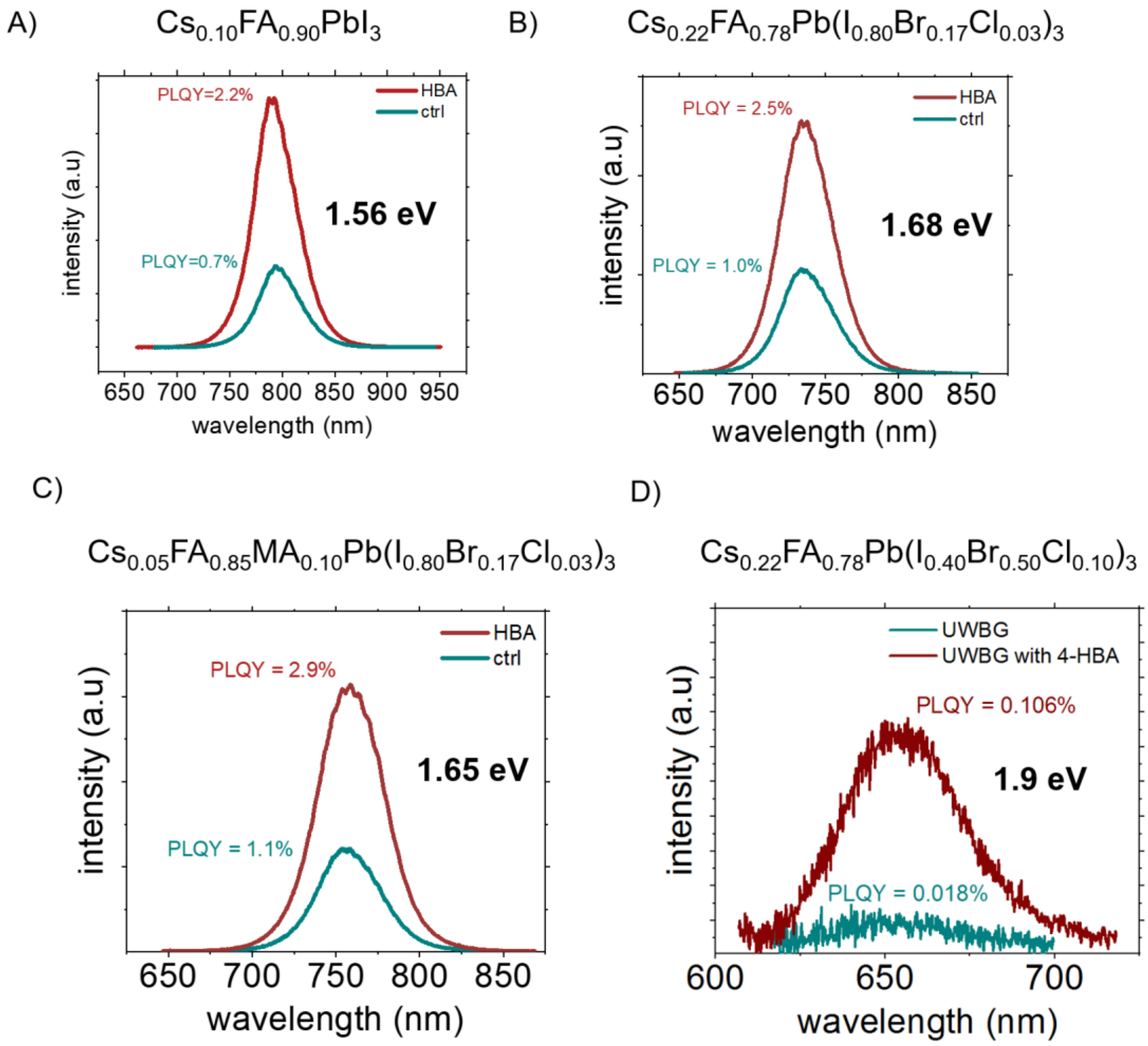

Figure R1. Universality of 4-HBA showing PLQY improvements in absorbers with bandgap ranging from 1.56 eV to 1.9 eV.

6. Is SIMIT qualified to certify the efficiency of triple-junction tandem solar cells? The accuracy of tandem solar cell efficiency measurements is critically dependent on the spectral match of the solar simulator. What is the spectral mismatch factor of the YSS-T155-2M simulator? Does it satisfy the standard requirements for testing the efficiency of triple-junction tandem solar cells?

We thank the referee for raising these important points regarding certification rigor.

SIMIT is fully qualified to certify multi-junction solar cells and follows internationally recognized testing standards. For the present devices, certification was conducted using a YSS-T155-2M AAA solar simulator. As the referee correctly notes, the accuracy of multi-junction efficiency measurements critically depends on spectral matching.

According to IEC 60904-1-1:2017, the spectral matching factor Zi for each junction should lie within 1.00 ± 0.03, while a tighter target range of ±0.01 is recommended when achievable. For our certified devices, the reported spectral mismatch factors were within ±0.015 of unity for all subcells, which is well within the IEC-required ±0.03 range and close to the recommended ±0.01 target.

SIMIT further follows a unified stabilized maximum power point tracking (MPPT) protocol comparable to those used by Fraunhofer ISE CalLab and JRC-ESTI. Certification requires that the device reaches a stabilization criterion defined by a rate of change in Pmax below ±0.2% per minute. Devices that do not meet this stabilization threshold are not certified. NREL applies a slightly different asymptotic JV

approach but with comparable stabilization criteria. Our certified devices satisfied the SIMIT stabilization requirements.

The measurement uncertainty reported by SIMIT for the certified triple-junction devices is 3%. For context, we compiled recent certified perovskite–Si tandem records and their reported uncertainties from the NREL chart. These range between approximately 0.9% and 3.3%, depending on the testing center. The 3% uncertainty reported by SIMIT is therefore fully within the range of established international certification laboratories despite having one additional junction compared to perovskite-Si tandems that increases the complexity. We have clarified this comparison in the revised manuscript to provide transparency and context.

Table 1. Extracted data from NREL chart on perovskite-Si tandem record efficiencies and uncertainties compared to the certified EPFL-CSEM triple-junction device.

**Device**	**Group**	**Efficiency (%)**	**Uncertainty (%)**	**Testing Center**
Perovskite-Si	KAUST	33.7	3.3	JRC-ESTI
Perovskite-Si	LONGi	33.9	0.9	NREL
Perovskite-Si	LONGi	34.6	1	JRC-ESTI
Perovskite-Si	LONGi	34.85	1	NREL
Perovskite-Si	LONGi	35	2.8	JRC-ESTI
Perovksite-perovskite-Si	**EPFL-CSEM**	30.02	3	SIMIT

**Measurement Method**	The solar simulator was adjusted to ensure the spectral mismatch factor for each sub-cell remained within 1.000±0.015. Irradiance was set to 1 Sun based on the reference cell calibration value and spectral mismatch factor of the current-limiting junction.<br>A bidirectional logarithmic voltage sweep, from open circuit voltage (Voc) to short circuit current (Isc) and back, was performed within a single flash in accordance with IEC 60904-1:2020.<br>MPP-Tracking was conducted for 300s using the P&O method, with a sampling interval of 1 s. It was considered to have reached steady-state when the rate of change in Pmax during the final 60 seconds was less than ±0.2%/min. The reported results correspond to the average values over the last 30 seconds of the tracking period.

Figure R2. Screenshot of the measurement methodology reported by SIMIT in the certification report.

7. Regarding the responses to comment #8, the explanation for the current density of the single-junction MBG devices remains unconvincing. Numerous publications (Nat. Energy, 10, 1371-1381, 2025; Nat. Photonics, 20, 55-62, 2026; Angew. Chem. Int. Ed., 64, e202505876, 2025) have demonstrated that devices (~1.52 eV) based on the same substrate can achieve significantly higher currents than those in this study. Particularly within the triple-junction device system addressed in this study, single-junction current densities exceeding 26 mA cm^{-2} have been achieved (Nature Materials, 2025, DOI: 10.1038/s41563-025-02367-8).

In the revised manuscript, the perovskite film thickness exceeds 1 μm in both the planar SEM (Figure 2c) and the textured SEM (Figure 2c). For an intermediate-bandgap perovskite, a current density of only 23.76 or 23.36 mA/cm^2 is clearly defies common sense in this research field! Most importantly,

the EQE (Figure 2g and 2i) shows a significant drop in the 600–800 nm range, which is markedly inconsistent with the claimed film thickness. The authors are requested to verify the consistency of these data.
Therefore, these data in the revised manuscript clearly do not support the authors' claim: "To overcome the current density limitations in the middle cell, a three-step perovskite deposition strategy enables the formation of thick…(Line 97-98)".

We appreciate the referee's careful evaluation of these data and the opportunity to clarify.

Figures 2g and 2i indeed correspond to devices employing the one-step absorber with approximately 600 nm thickness, not the three-step absorber. These figures were intentionally designed to isolate the optical effect of the middle reflector, and the three-step absorber was not integrated into those specific stacks. We have now clarified this more explicitly in the revised manuscript to avoid confusion.

The performance of the three-step absorber is presented in Fig. S43 and related supplementary figures. In semi-transparent MBG–Si tandem configurations, where the three-step absorber and illumination from the electron-transport-layer side are relevant, we observe a clear enhancement in the 600–800 nm region. The integrated current density of the MBG subcell reaches 25.1 mA cm^{-2} in semi-transparent configuration, which is among the highest values reported for such architectures.

It is important to note that opaque single-junction glass devices are not directly representative of the final triple-junction architecture. In particular, illumination direction plays a critical role. For the three-step absorber, the modified interface is the electron-selective interface, which is the illuminated side in tandem configurations but not in opaque single-junction measurements on glass. Consequently, opaque single-junction current densities cannot directly validate the architecture-specific improvements targeted for semi-transparent tandem operation.

We have revised the manuscript to clearly separate one-step optical reflector studies from three-step absorber optimization and to emphasize that the current-density improvements are demonstrated under architecture-relevant illumination conditions.

8. The presentation logic for PV performance in Figure 3 requires adjustment to more clearly highlight the innovative contributions of this study. It is recommended that Cell-1, Cell-2, and Cell-3 in Figure 3 be replaced with independent optimization control groups corresponding specifically to the innovations of this study. Specifically, please present data for 3-junctions optimized with: (1) 4-HBA additive, (2) the three-step deposition strategy, (3) SiOx nanoparticle reflectors, rather than devices with varied SAMs. This will allow for a visual isolation of the independent contribution of each specific innovation to device performance, avoiding confusion caused by variations in other factors.

We thank the referee for this constructive suggestion. We agree that clearly isolating the contribution of each innovation is important for readability and scientific transparency.

In the revised manuscript, we reorganized the performance discussion to more explicitly reflect the stepwise integration of each development into the triple-junction baseline. Figure R2 presents the sequential device evolution, beginning from the baseline architecture and progressively incorporating: (1) the 4-HBA additive in the wide-bandgap top cell, (2) the three-step deposition strategy for the middle-bandgap absorber, and (3) the SiOx nanoparticle middle reflector.

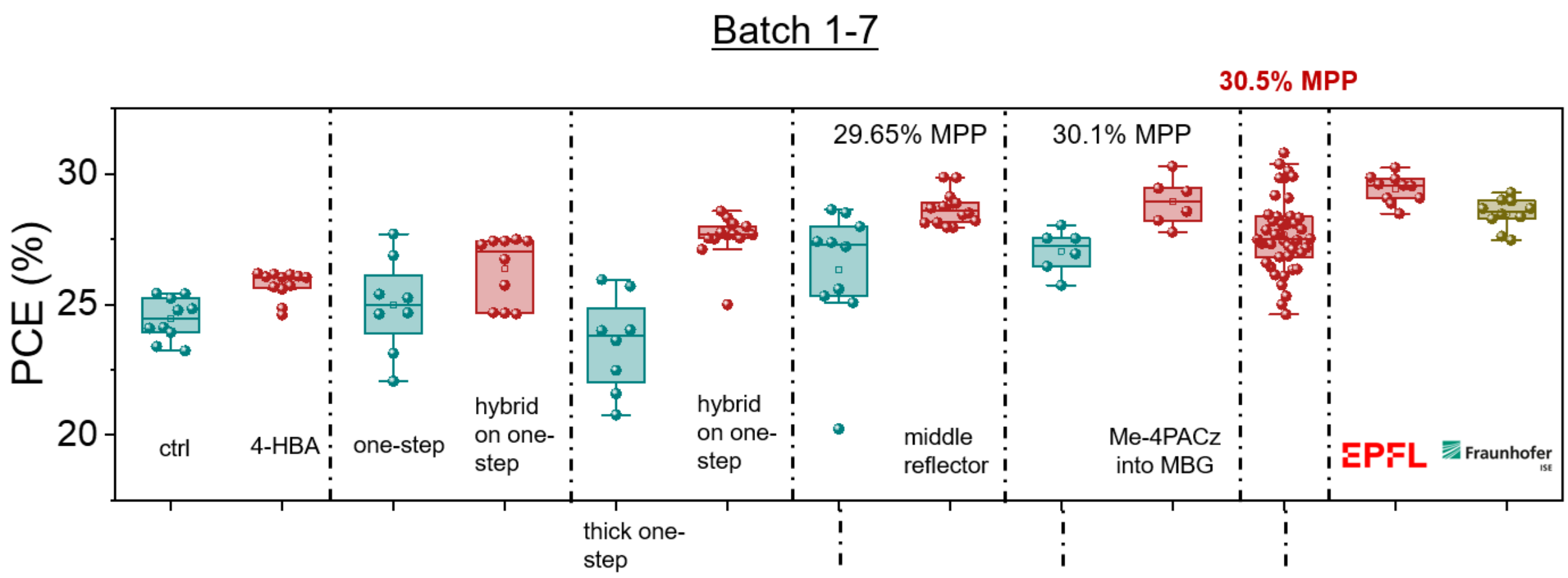

Figure R3. Device performance along the first 7 batches of fabrications with different conditions tested.

9. Figure 2h and 2i in the revised manuscript display the reflection and absorption of the middle-bandgap perovskite layer. The data reveal that following SiOx-np optimization, both reflection and absorption of this layer increase simultaneously-a phenomenon that contravenes fundamental physical principles. Furthermore, the authors assert that SiOx-np, functioning as an intermediate reflective layer, reflects photons with wavelengths >775 nm back into the middle cell while reducing transmission to the bottom cell. This claim is directly contradicted by the data presented in Figure R3b.

We appreciate the opportunity to clarify this point.

There is no violation of physical principles. The key distinction lies in the optical stack configuration. When SiOx-np is deposited directly on silicon without an overlying high-index perovskite layer, it functions as an anti-reflective coating due to improved impedance matching between air and silicon. This scenario corresponds to Fig. R3b.

However, in the actual triple-junction device architecture, the relevant interface is perovskite ($n \approx 2.5$) to SiOx-np ($n \approx 1.33$). At this high-to-low refractive index transition, Fresnel reflection occurs. Photons near the perovskite band edge that are not initially absorbed propagate to this interface, are partially reflected back into the perovskite layer, and are then reabsorbed. This increases effective optical path length and enhances band-edge absorption.

This mechanism is analogous to back reflectors in silicon solar cells and perovskite–Si tandems, where enhanced infrared reflectance increases absorption in the bottom cell by increasing reflection. In our case, enhanced reflectance near 775–900 nm increases middle-cell absorption while slightly reducing transmission to the silicon bottom cell. The experimental EQE data, optical simulations, and device-level results are mutually consistent.

We have revised the manuscript to more clearly distinguish between the anti-reflective configuration and the middle-reflector configuration and to explicitly describe the relevant refractive-index contrasts in each case.

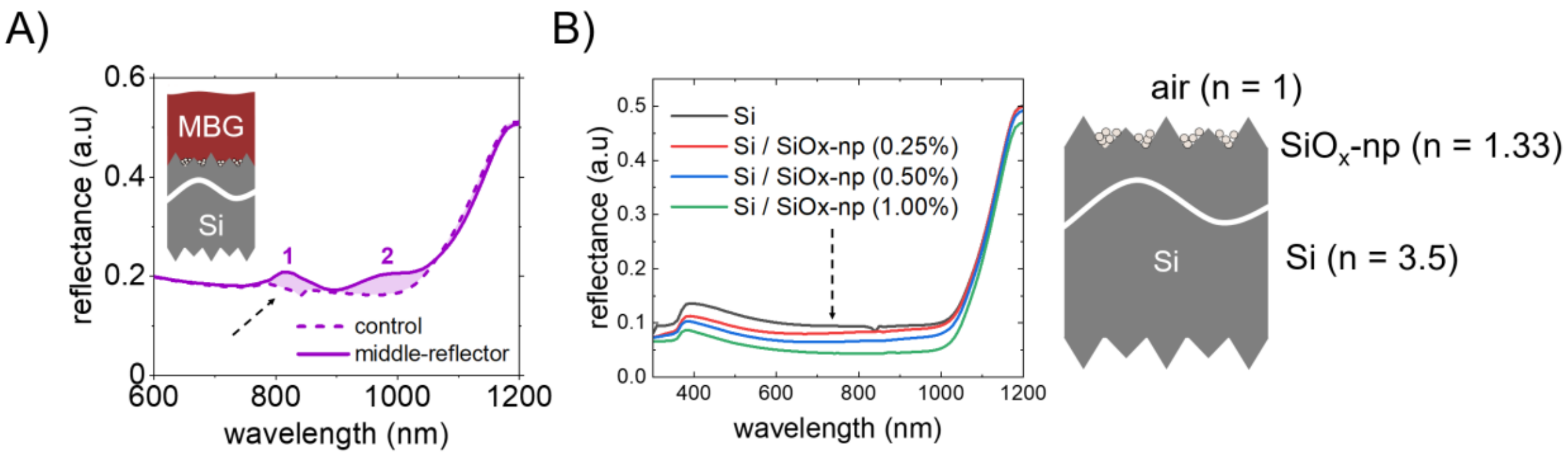

Figure R4.A) Reflectance spectrum of MBG-SiOx-Si stack, B) reflectance spectrum of SiOx-Si stack.

10. The wide-bandgap perovskite in the three-junction device compound primarily consists of FA^+ and Br^- ions, yet the authors' theoretical calculations are based on the $CsPbI_3$ model. Given the significant discrepancy between this model and the actual material composition, the authors are requested to clarify the specific rationale and justification for selecting this particular model.

We focused on $CsPbI_3$ as a prototypical perovskite since it is the simplest model that still captures the essence of the interactions. We have investigated the role of the cation, and **Table S1** shows that FA^+ behaves similarly to Cs^+. Regarding the halide composition, we note that both the single-junction and triple-junction devices contain substantial amounts of I^- (60% and 40%, respectively). Based on the binding motives shown in **Fig S6**, it is reasonable to assume that $CsPbBr_3$ should behave similarly as $CsPbI_3$ in its interaction with HBA. This is also in line with our TOF-SIMS results shown in **Fig. S18**.

Furthermore, the universality of 4-HBA was experimentally verified across multiple compositions with bandgaps ranging from 1.55 eV to 1.9 eV, demonstrating that its effect is not restricted to a single iodide-rich model system. We have clarified the rationale for model selection and its limitations in the revised manuscript.

11. In Figure 4a, why is the initial value of the normalized PCE not 1? The retention rates labeled on the right y-axis (24.5% and 39.5%) are easily confused with the PCE values.

We thank the referee for this observation. The figure presents normalized PCE values to facilitate comparison of degradation trends independent of initial efficiency differences. The initial absolute PCE values of the devices are now explicitly provided in the Supplementary Note 14 to avoid ambiguity. Moreover, we have removed the retention values in the figure to avoid confusion.

12. The authors expanded the effective active area of the triple-junction device from 1 cm^2 to 54 cm^2 and provided an analysis of performance losses in Supplementary Note 2. However, the analysis appears overly broad, lacking a clear logical prioritization of the primary and secondary loss mechanisms.

We appreciate the reviewer's comment regarding the logical structure of Supplementary Note 2. We respectfully disagree that the analysis is overly broad; however, we acknowledge that the hierarchy of

loss mechanisms was not stated explicitly enough. In the revised Supplementary Note 2, we have reorganized the discussion to clearly prioritize the dominant and secondary scaling losses. We have clarified this hierarchy explicitly in the revised Supplementary Note to improve logical flow and ensure that the dominant and secondary loss mechanisms are clearly distinguished.

13. The authors utilized non-radiative recombination energy loss (NR-loss) analysis for molecular screening (Figures S1 and S2). However, both the main text and supplementary materials lack detailed descriptions of the key testing and calculation procedures.

We thank the referee for pointing this out. In the revised Methods section, we now explicitly state the calculation procedure for non-radiative losses using the relation:

$$\text{NR-loss} = (kT/q) \cdot \ln(\text{PLQY})$$

and provide sufficient detail to ensure reproducibility of the analysis.

14. The data and figure processing lack rigor. For example: The current layout and clarity of Figures 4b and 4c do not meet the publication standards of a high-quality journal. The authors are requested to readjust the layout and improve the image resolution to enable accurate interpretation by readers.

We thank the referee for this comment. Figures 4b and 4c have been reformatted for improved clarity and higher resolution. Scale and legend placement have been optimized to meet publication standards and to ensure accurate interpretation by readers.

Referee #3 (Remarks to the Author):

I co-reviewed this manuscript with one of the reviewers who provided the listed reports.

Referee #4 (Remarks to the Author):

The manuscript entitled "30%-efficient perovskite-perovskite-silicon triple-junction solar cells with improved carrier and photon management" with Ref # 2025-07-17350, by Artuk et al., reports monolithic (series-connected) perovskite-perovskite-Si triple-junction photovoltaics reaching certified percentage conversion efficiencies (PCEs) at or above 30%. The study targets two central performance bottlenecks specifically in triple-junction perovskite-perovskite-Si triple-junction solar cells: (i) open-circuit voltage (Voc) limitations in the wide-bandgap (WBG) top sub-cell, and (ii) photocurrent limitation in the middle-bandgap (MBG) perovskite sub-cell due to constraints on absorber thickness and related transport and stability penalties. The authors propose a co-ordinated "carrier and photon management" approach: a non-volatile additive (4-hydroxybenzylamine, 4-HBA) to regulate WBG crystallization and passivate defects, then a 3-step/hybrid deposition method to form thick MBG absorbers while retaining extraction and suppressing detrimental mobile ion effects, and a special optical "middle reflector" based on low refractive index SiOx nanoparticles accumulating in textured silicon valleys to redistribute photons toward the MBG sub-cell. Importantly, the revised submission includes third-party certification from the Shanghai Institute of Microsystem and Information Technology (SIMIT) and expanded mechanistic evidence, as stated in detail in the rebuttal from the authors.

Overall, the revised manuscript has been substantially strengthened compared with the prior version, particularly through: (1) independent certification of >30% efficiency and steady-state reporting and (2) reorganization and added characterization intended to sharpen mechanistic claims. The work sits at an appropriate point in the perovskite multi-junction PV field: triple-junction solar cell demonstrations are increasingly expected to justify not only record metrics but also a clear, transferable step forward in the progression toward higher PCEs. The present study is close to that standard and is likely suitable for publication in Nature after some further minor revisions below are addressed:

We sincerely thank the referee for the careful and constructive evaluation of the revised manuscript and for the positive assessment that the work is close to the standard expected for publication in *Nature*. We particularly appreciate the referee's acknowledgement of the strengthened mechanistic presentation and the inclusion of independent certification and stabilized reporting. We note that, due to editorial length constraints, we were required to substantially reduce the main text in the revised version. As a result, several detailed discussions and methodological clarifications have been relocated to the Supplementary Notes to ensure completeness while maintaining compliance with the journal's formatting requirements. Below, we address each point in detail and describe the corresponding revisions implemented in the manuscript and Supplementary Information.

General Comments and Concerns:

Firstly, thank you to the authors for making the necessary revisions to the manuscript. The clarity and detail have been improved, especially with the contributions from the additional authors. The revised manuscript now highlights the mechanistic role of 4-HBA and of the optical middle reflector. This is the correct overall direction, however, the text would benefit from minor revisions to more clearly present the work as a coherent and broadly applicable framework.

The main text should clearly separate general, transferable mechanisms from device-specific optimizations. In particular, the authors should state which effects of 4-HBA are expected to be broadly applicable (for example, its influence on defect formation and recombination pathways) and which performance gains depend on specific architectural choices, such as the SAM stack or surface texturing.

We thank the reviewer for highlighting this point. We have added the following discussion in Supplementary Note 4:

The reduction of bulk non-radiative recombination by 4-HBA constitutes a general, material-level improvement that is expected to be broadly transferable across device architectures and absorbers (see Supplementary Note 5), as it originates from suppressed defect formation and the passivation of existing defects within the perovskite absorber. However, in complete devices, bulk improvements can only be translated into voltage gains if the contact interfaces do not impose additional non-radiative recombination losses. Accordingly, to fully benefit from the bulk enhancement induced by 4-HBA and reach 1.405 V, both interfaces were passivated to minimize surface recombination velocities.

Importantly, once interfacial recombination channels are sufficiently suppressed, bulk losses become more clearly reflected in QFLS and device-level metrics. Conversely, recombination-active interfaces can mask bulk improvements; without optimized SAM and C60 passivation, the intrinsic bulk benefits introduced by 4-HBA would not fully translate into device-level voltage gains. Therefore, while 4-HBA addresses broadly applicable bulk limitations, optimized hole- and electron-contact interfaces remain essential to reveal and harness these improvements in high-performance WBG devices.

This is not a request for additional experiments, but for clearer explanation. The manuscript would benefit from one short paragraph that explicitly links the key experiments to the main claims. For example, the authors could briefly state which measurements support improved crystallization and which support defect passivation, as well as which support reduced recombination, and clarify which alternative explanations were excluded. Although the rebuttal notes the addition of PL, device measurements, ToF-SIMS, transient SPV, fracture energy, and BACE data, the manuscript should ideally present these results as parts of a single coherent story, rather than as separate pieces of supporting evidence.

We appreciate this request and agree that the manuscript benefits from a more explicit "logic bridge" between measurements and claims. We therefore added a concise paragraph in the main text at the end of each section that connects the key experiments to the central mechanisms.

Taken together, DFT, NMR, GIWAXS, and spectroscopic analyses indicate that 4-HBA coordinates with Pb^{2+} in the precursor solution without forming secondary phases, thereby maintaining ink stability while moderating crystallization kinetics. This interaction is consistent with transient stabilization of precursor species and delayed nucleation, resulting in improved film microstructure and reduced defect density. ToF-SIMS measurements show that 4-HBA is distributed throughout the absorber layer, supporting bulk passivation rather than interface-localized modification. Across multiple compositions, the incorporation of 4-HBA consistently reduces non-radiative recombination losses.

This three-step deposition strategy provides a synergistic improvement that extends beyond mere thickness enhancement, simultaneously reducing the bandgap, maintaining efficient charge extraction, and enabling thicker absorbers with enhanced mechanical adhesion to SAM to deliver higher current densities without performance degradation. Unlike the one-step process, it prevents film delamination

and the formation of rough, wrinkled morphologies, while not elevating ionic losses, crucial for sustaining high current densities.

With the middle-reflector, the current density increases in the middle-cell and decreases in the Si bottom-cell, hence promising a better current distribution in the triple-junction device. Thus, the SiO_x-np middle reflector is not an isolated optical enhancement, but a deliberately balanced optoelectronic element whose net benefit emerges at the triple-junction level. When combined with the three-step-processed absorber, our model predicts a triple-junction PCE above 31% (see **Table S2**).

Secondly, the certification and stabilized efficiency reporting are a major strength, but reporting clarity can be improved. Given that independent certification is a central for record devices, and the revised submission now includes SIMIT certification reports and comparisons with in-house curves (Figs. S70–S72) and additional inter-lab comparisons (Fraunhofer ISE) for sub-30% devices. Although, this is a strong response to prior review requests, the main text should ensure that the reader cannot misinterpret which numbers are (i) in-house reverse scan, (ii) in-house stabilized MPP, (iii) certified reverse scan, and (iv) certified steady-state. The manuscript does provide these categories but given the prominence of the "30%" headline, it would be beneficial to present them in a compact, explicit manner in the Results, with consistent terminology and matching device IDs.

We thank the referee and fully agree that record-level work must present measurement categories in a precise and unambiguous manner. In the revised Results section, we now report all efficiencies in a compact and explicit format with consistent terminology, clearly distinguishing between in-house reverse-scan, in-house stabilized MPP, certified reverse-scan, and certified steady-state values.

In addition, we have included Supplementary Notes 10 and 11 to further clarify the differences between in-house and certified measurement procedures, including scan conditions and stabilization protocols.

Throughout the Introduction, Abstract, and Conclusion, we consistently report the certified stabilized efficiency of 30.02% to avoid ambiguity and ensure coherence across the manuscript.

The reviewer appreciates the authors' detailed discussion in the rebuttal regarding the limitations of integrating measured EQEs in monolithic multi-junction devices, particularly in the presence of bias-dependent effects, luminescent coupling, and non-ideal sub-cell behavior. These considerations are well established in the III–V community and are indeed relevant for perovskite–silicon tandems and triple-junctions, as correctly described. The concern about potential misinterpretation of current matching based on absolute EQE integration is therefore well-taken. That said, it would still be valuable for readers to have access to estimated short-circuit current densities for the individual top, middle, and bottom sub-cells, even if these values are not presented in the main figure and are accompanied by appropriate caveats. One possible compromise would be to report integrated EQE-derived current densities for representative devices in the Supplementary Information, together with a short Supplementary Note explicitly outlining the known limitations, potential artifacts, and measurement sensitivities. This would preserve methodological rigour while still aligning with common practice in the perovskite multi-junction literature, where such estimates are routinely reported to facilitate comparison and interpretation.

We appreciate the referee's balanced position on this point and agree that providing EQE-integrated current-density estimates, accompanied by appropriate caveats, is valuable for readers. We have therefore included representative EQE-integrated current densities for the top, middle, and bottom

subcells in the Supplementary Information (Fig. S69). In the corresponding figure caption, we explicitly outline the known limitations associated with EQE analysis in monolithic multi-junction devices, including bias-dependent carrier extraction, luminescent coupling between subcells, series-connection constraints, and measurement sensitivities.

This approach maintains methodological rigor while aligning with established community practice for reporting and benchmarking multi-junction device performance.

Minor Concerns:

The authors could add a short statement clarifying whether the certified steady-state measurement conditions (illumination spectrum, temperature control, preconditioning, stabilization time) are fully matched to the in-house stabilized protocol, or if not, what differences exist.

We thank the referee for this point. We have added a short clarification indicating whether the certified steady-state protocol is fully matched to the in-house stabilization procedure and, where differences exist, what they are (Supplementary Note 11).

Ensure the certified steady-state values are given equal prominence to reverse scans, especially since the work emphasizes stabilized efficiencies.

We agree and have revised the text to emphasize stabilized performance, with steady-state certified values discussed alongside reverse-scan values rather than being presented as secondary. We also adjusted wording in the abstract and Results to ensure that the narrative consistently reflects the importance of stabilized reporting.

For the middle-cell three-step/hybrid deposition, there is outstanding performance, but reproducibility and trade-offs should be explicit (possibly list some pros and cons). The manuscript reports that a champion device reached reverse-scan efficiency of 31.15% with stabilized efficiency 30.9%, and batch statistics of 22 devices average 29.65%. This is impressive and suggests good repeatability at the device level. However, the three-step MBG deposition concept, while clearly effective, raises questions that should be addressed more directly:

• For example, is the process window tight or broad? The text implies robust performance but does not clearly communicate yield in terms of how many wafers/batches were attempted and how representative the champion batch is.

• What is the primary trade-off? For example, does increased thickness introduce higher series resistance, more pronounced hysteresis, or new failure modes under damp heat and outdoor conditions?

We thank the referee for emphasizing the importance of clearly addressing reproducibility and process trade-offs, particularly for a multi-step fabrication route that is likely to attract strong community interest. We have now included a dedicated discussion in Supplementary Note 8 and Fig. S82.

Regarding yield and representativeness, in addition to the batches reported in the initial submission, we fabricated four additional batches during the review process using approximately twelve wafers (nine pixels per wafer), corresponding to more than 100 devices. Excluding shunted pixels, each batch achieved average efficiencies above 29%. This level of reproducibility also enabled subsequent scaling to wafer-scale devices, where uniformity requirements are significantly more stringent. In the second revision, we further fabricated an additional batch of 54 cm^2 devices, achieving a slightly improved efficiency compared to the previous batch, reaching 26.3%.

Compared to purely hybrid-processed absorbers, the three-step approach exhibits a broader and more forgiving process window. In our implementation, full or near-complete conversion of the evaporated PbI_2 layer is achieved over an organohalide concentration range of approximately 0.25–0.35 M, within which device performance remains stable. At 0.25 M, a minor PbI_2 excess remains; this does not measurably affect initial performance but is likely detrimental to long-term stability, potentially facilitating autocatalytic degradation pathways (22,23). In contrast, purely hybrid processing without an underlying one-step absorber requires tighter control, with an optimal concentration centered at 0.30 M. Outside this narrow window, conversion becomes incomplete or microstructural quality deteriorates (Fig. S81).

We attribute the broader processing window of the three-step route to the presence of the underlying one-step absorber, which acts as a reservoir of inorganic and organic halides. This reservoir partially buffers deviations in conversion chemistry and reduces sensitivity to small fluctuations in precursor concentration or local solvent dynamics. At the same time, one-step deposition remains intrinsically simpler and marginally more repeatable at the laboratory scale, as it involves fewer sequential processing steps and therefore fewer potential sources of cumulative variability.

The primary trade-off of the three-step approach emerges under under-converted conditions, when the organohalide concentration falls below the optimal window. In this regime, residual PbI_2 can persist at the buried interface, leading to increased series resistance, reduced fill factor, and more pronounced hysteresis. Fully converted or slightly under-converted three-step absorbers, however, do not exhibit systematic increases in series resistance or hysteresis relative to the one-step baseline, indicating that increased thickness does not impose transport limitations when conversion is complete. Residual PbI_2 in thicker interfacial regions is detrimental in this configuration, as it impedes carrier extraction and may accelerate degradation pathways reported in the literature.

• The Supplementary Information indicates that residual PbI2 can appear in “under-converted” three-step absorbers (Fig. S67), while one-step absorbers can look more homogeneous in some cross-sections, which could confuse readers if not carefully explained. The authors should ensure the message is unambiguous for when and why residual PbI2 is observed, whether it is beneficial, neutral, or detrimental, and how it correlates with device-to-device spread.

We thank the reviewer for clarifying this point. We included a detailed note under Fig.S67 regarding the residual PbI_2 observed in this batch of devices.

We note that despite the visible presence of PbI_2 residuals in this batch of devices, all high-energy photons ($\lambda < 600$ nm) are absorbed in the top subcell. As a consequence, the PbI_2 is not directly exposed to high-energy illumination, which could otherwise (i) contribute to parasitic absorption losses and (ii) accelerate photo-induced degradation pathways. Accordingly, no initial performance deterioration is observed.

However, we consider it likely that residual PbI_2 could negatively affect long-term operational stability, particularly under extended thermal and electrical stress, and therefore regard its presence as undesirable from a durability perspective.

• For the stability discussions, there is good progress, but could the authors align the narrative with realistic deployment expectations? The Supplementary Figures include damp heat testing (noting 22.9% relative PCE loss after 500 h for an encapsulated 3J device) and outdoor monitoring setup images with no visible degradation on a specific date. This is useful, but the stability story would benefit from providing a succinct "stability summary" in the main text describing the test conditions (temperature, RH, encapsulation type, MPP tracking versus periodic JV, and duration). Even if the detailed plots remain in the Supplementary Information, the reader needs a coherent takeaway in the main paper (1-2 sentences). No new experiments are necessarily required, but the stability claims must be carefully framed to avoid overstating "stability" based on limited windows.

We thank the referee and agree that the stability narrative must be carefully framed. In the revised main text, we have added one to two paragraphs summarizing test conditions, results and the principal takeaway, without overstating the conclusions.

Operational stability of unencapsulated wide-bandgap single-junction devices (with and without 4-HBA) was evaluated under maximum power point tracking in N_2 at progressively elevated temperatures up to 80 °C for a total of 4,500 h (**Fig. 4(a); Fig. S77, Supplementary Note 14**). While both control and 4-HBA devices retained ~70% of their initial efficiency after ~2,000 h, control devices subsequently exhibited accelerated degradation, resulting in a final PCE retention of 24.3% (best case) after 4,500 h. In contrast, 4-HBA devices showed slower degradation kinetics, retaining up to 39.5% of their initial efficiency. Optical inspection revealed more pronounced formation of PbI_2-rich degradation phases in the control devices (**Fig. 4(b)**), consistent with the lower Pb^0 content observed by XPS in 4-HBA-modified films (**Fig. 1(e)**).

Encapsulated triple-junction devices (glass/glass) were further subjected to damp heat testing (85 °C/85% RH). After 500 h, devices exhibited relative PCE losses of 13.8% and 22.9% (**Fig. 4(d); Fig. S78**). Outdoor testing in Neuchâtel, Switzerland, over four months showed stable performance ratios during low-temperature operation, with moderate reductions at elevated temperatures but no visible degradation (**Fig. 4(e); Fig. S79–S80**).

Detailed stability plots and full descriptions of the experimental setup remain in the Supplementary Information due to word limit constraints. Nevertheless, the main manuscript now provides a clear interpretive anchor, explicitly stating the test conditions (including encapsulation strategy, temperature and relative humidity where applicable, monitoring protocol, and test duration). We have also refined the language throughout to ensure that the term "stability" is used strictly within the context of the demonstrated testing windows and is not extrapolated beyond the reported conditions.

• In Supplementary Note 1, it may be premature to describe the Me-4PACz/perovskite interface as the "weakest layer" in the device stack unless fracture energy measurements have been performed for all relevant interfaces throughout the full stack. In the broader perovskite–silicon tandem literature, the

perovskite/C60 interface is more frequently identified as one of the mechanically weakest interfaces when assessed by fracture energy. The authors may wish to consider recent work by Poma et al., EES Solar (accepted February 2026), "Incorporating a Naphthalene Diimide Polymer into a Fullerene Electron-Transport Layer to Improve the Fracture Energy of Perovskite Solar Cells," which directly addresses this issue (although on the electron transport layer side). This reference may also be relevant to the discussion in the main text (Page 8, Lines 327–333). Including for stability as C60 is often the weakest link.

We appreciate this careful observation and agree that the original phrasing could be interpreted too broadly. We have therefore revised the text to clarify that the fracture-energy discussion refers specifically to the tested stack configuration and does not imply that the Me-4PACz/perovskite interface is universally the weakest interface across the entire device stack. We acknowledge that in many perovskite and tandem architectures, the perovskite/C60 interface is frequently reported as mechanically weak. Accordingly, we have softened the statement and reframed the conclusion to remain strictly limited to the measured configuration.

In addition, we now cite the suggested work by Poma et al. (EES Solar, accepted Feb 2026) as contextual support for the broader discussion of mechanically weak interfaces and the importance of ETL-side adhesion for device stability. This reference has been incorporated in Supplementary Note 7 to appropriately situate our findings within the evolving literature on interfacial mechanical robustness.

Despite these improvements to the adhesion of the bottom interface, the perovskite/C_{60} interface — along with the adjacent C_{60}/ALD-SnO_x interface — remains among the mechanically weakest regions of the device stack due to limited interfacial adhesion, warranting further interfacial engineering to enhance mechanical robustness and long-term stability (*19–21*). Recent studies by Poma *et al.* and Schutt *et al.* have demonstrated that incorporating alternative interlayers or modified electron-transport materials at this interface can substantially increase fracture energy, thereby improving mechanical durability.

Minor Concerns and Suggestions:

• The rebuttal explains why symmetric double-sided nanotexturing was used and why asymmetric texturing adds processing complexity. This is a reasonable response, however, in the manuscript, the authors could consider adding one sentence clarifying whether the present optical gains are expected to persist under asymmetric industrial texturing approaches.

We thank the referee. We have added a short sentence clarifying that the optical mechanism of the middle reflector is not intrinsically tied to symmetric texturing, and that similar photon redistribution effects are expected under asymmetric industrial texturing approaches, although the absolute balance between bottom-cell response and current mismatch may differ due to altered back reflection of silicon.

For the silicon bottom-cell architecture, we employ a symmetric double-sided sub-micron-textured configuration, selected for its fabrication simplicity and compatibility with high device performance. Compared to asymmetric texturing strategies reported previously, this configuration enables streamlined processing while maintaining competitive optoelectronic characteristics. While slight increases in bottom-cell current can be achieved with asymmetric texturing, this can also enhance current mismatch between the bottom and middle cells, in some cases leading to a modest increase in fill factor. Importantly, the optical working principle of the middle reflector—namely, photon

redistribution—is not intrinsically dependent on symmetric texturing and is expected to operate effectively under both symmetric and asymmetric configurations, although the precise current balance may differ due to modified back-reflection of silicon.

• A short paragraph in the Discussion could help clarify what stability milestones remain for perovskite-perovskite-Si triple-junctions (for example, damp heat duration expectations) and where this work currently stands, without overclaiming (more for future work and directions) (optional).

We agree that it is useful to place the results in the context of realistic deployment expectations without overclaiming. We therefore added a sentence at the end of the stability section in the main text.

The stability demonstrated here provides an initial benchmark for perovskite–silicon triple-junction devices; however, extended damp-heat, thermal cycling (*49*), outdoor monitoring, and high-temperature light-operational testing will be required to meet industry qualification standards.

• Page 10, Line 414: When the authors refer to overall efficiency gain of 0.66%abs, it's not clear from looking at Table S2. Can the authors be more explicit with what numbers they are referring to please?

Of course, we refer to these two efficiency numbers with middle-reflector integration (highlighted in red).

Condition	Rs total (ohm.cm^2)	Top-cell current (mA/cm^2)	Middle-cell current (mA/cm^2)	Bottom-cell current (mA/cm^2)	V_{OC} (V)	J_{SC}(mA/cm^2)	FF (%)	PCE (%)
**Baseline**	13.5	12.75	11.00	17.00	3.110	11.018	86.23	29.55
**Rs + 1 ohm.cm^2**	14.5	12.75	11.00	17.00	3.110	11.018	85.90	29.44
**3-step absorbers**	14.5	12.75	11.75	16.25	3.110	11.767	84.60	**30.98**
**Middle-reflector + 3-step**	14.5	12.75	12.25	15.50	3.110	12.265	82.92	**31.64**
**Matched**	14.5	12.50	12.50	15.50	3.110	12.500	80.62	31.36

• Several multi-panel figures are quite compact. If allowable within the Nature journal format, the authors could opt to use slightly larger axis labels or a simplified legend would help with readability.

We thank the referee for this practical suggestion. We agree that clarity and readability of multi-panel figures are essential. We will coordinate with the editorial team to ensure that all figures meet visibility and legibility standards in the final layout, including appropriate axis-label sizing and optimized legend formatting within *Nature*'s production guidelines.

• Page 13, Figure 4(E). The PR could be explained in detail, as it's not really a common metric in the PV field.

We thank the referee for pointing out that the performance ratio (PR) may not be a familiar metric for all readers in the photovoltaic community, particularly outside of outdoor PV and systems testing. In the revised Supplementary Information, we provide a detailed definition and calculation procedure for PR in the Methods section under outdoor testing, including the relevant normalization to irradiance and temperature where applicable. To improve accessibility in the main manuscript, we have also updated the Fig. 4e caption to explicitly direct readers to the Supplementary Methods for the PR definition and calculation details, and we added a brief clarifying statement in the main text to indicate that PR is used here as a normalized metric to compare outdoor energy yield and performance retention under variable real-world irradiance conditions.

To evaluate the field performance of the encapsulated 3J cells, time series data were analyzed from two devices tested outdoors in Neuchâtel, Switzerland, between February 6 and June 3, 2025. Global horizontal irradiance (GHI) was measured using a pyranometer co-located with the test setup.

The performance ratio (PR) was computed and analyzed over time to extract long-term trends and identify potential anomalies. The PR is a dimensionless metric that compares the actual output to its expected output under standard test conditions (STC). It normalizes the device's power output by both its nominal rating and the incident irradiance, and is defined as:

$$PR(t) = \frac{P_{MPP}(t)/P_{nom}}{G(t)/G_{STC}}$$

Where $P_{MPP}(t)$ is the maximum power point measured at time t (in mW), P_{nom} is the nominal power rating of the device under STC, $G(t)$ is the measured irradiance at time t (in W/m^2), G_{STC} is the reference irradiance under standard conditions (1,000 W/m^2).

PR values outside the plausible range (i.e., below 0.3 or above 1.3) were excluded to filter out erroneous data and outliers. To study temporal evolution, the 10-minute PR values were aggregated into daily

statistics. For each day, the average PR was calculated along with its day-to-day variability, using standard deviation as a measure of spread. To reduce noise in the daily data and emphasize long-term behavior, a Gaussian smoothing filter was applied to the daily PR averages ($\sigma = 8$), to effectively smooth out short-term fluctuations while preserving broader performance patterns over time. Additionally, confidence intervals were added above and below the smoothed trendline using the daily standard deviation.

• Page 9, JV and EQE in Fraunhofer ISE: In the context of the spectral response and current-matching discussion in the Supplementary Information, it would be appropriate to also cite the earlier work by Meusel et al. on spectral response measurements and measurement artifacts in monolithic triple-junction devices (Prog. Photovolt. Res. Appl. 11, 499–514 (2003)). This paper complements Ref. 28 and provides useful historical context for spectral response artifacts in series-connected multijunction solar cells.

We thank the reviewer and include this reference in the Fig.S69 caption within the discussion of EQE measurements.

Grammatical Errors and Corrections:

We thank the referee for the careful proofreading and the detailed list of editorial corrections. All requested text edits have been implemented, including the change to "nanoparticles that accumulate in the front valleys," and we have standardized terminology throughout the manuscript to ensure consistency in the use of "sub-cell," "middle cell," and "triple-junction," as well as consistent formatting for "SiOx-np" after the first definition. We also corrected formatting issues such as "~800 nm" without spacing, removed duplicated sentences, corrected typographical spacing before references, and standardized capitalization (e.g., iVOC) and chemical nomenclature (EtOH versus ethanol) across the manuscript and Supplementary Information. We additionally conducted a final full-text consistency pass to eliminate remaining hyphenation and style inconsistencies.

Regarding the referee's query about the figure citation on Page 10, Line 399, we confirm that this reference correctly points to Fig. S47(b), which contains the schematic illustrating refractive-index relationships in the optical system, and is therefore not intended to refer to Fig. S49(b). We have nevertheless double-checked figure numbering and cross-references throughout to avoid any potential citation mismatches

Main Article:

Page 1, Line 64: "nanoparticles accumulated in the front valleys..." to "nanoparticles that accumulate in the front valleys..."

Page 2, Line 86: "...among the sub-cells; ..." standardize "sub-cells" vs "subcells" (both appear later). Or just pick one that, but please keep consistent. Similarly, for "middle-cell" throughout the text.

Page 2, Line 89: "...perovskite-Si-based triple junctions...", again standardize to "triple-junctions" to match the majority usage.

Page 2, Line 95: "...through a coordinated carrier and photon management strategy." consider hyphenating "carrier- and photon-management strategy".

Page 2, Line 101: "SiOx-nanoparticles (SiOx-np) ..." please standardize terminology to one preferred form (e.g., "SiOx-np" thereafter, and avoid switching back to "SiOx nanoparticles" later).

Page 7, Line 296:
Current: "...constrained to thicknesses below ~ 800 nm..." remove the space after the ~, so "~800 nm".

Page 10, Line 399: "Fig. 47(b)" should be "Fig. S49(b)"?

Page 10, Line 435-438: "To decrease such a mismatch in the further experiments, the top-cell bandgap was increased to ~1.9 eV from 1.8 eV by increasing the Br fraction to 50%. To decrease such a mismatch in the further experiments, the top-cell bandgap was increased to ~1.9 eV from 1.8 eV by increasing the Br fraction to 50%." This sentence has been repeated twice!

Page 12, Line 486: "(reverse scan efficiency of 27.10%) (15)." remove the double space before the reference: "...27.10%) (15)."

Page 12, Line 491: "The iVoc of the three-step middle-cell..." please standardize "iVoc" to "iVOC" to match usage in Lines 487–489 and Line 493.

In general, there are many hyphenation inconsistencies, such as subcell vs sub-cell, middle-cell, triple-junction, SiOx-nanoparticles (SiOx-np), etc. Please check carefully and keep consistent throughout the manuscript. Also, please ensure consistent use of Ethanol versus EtOH, PV-tools capitalization, etc. The rebuttal indicates these were fixed, but could the authors please do a final check.

Supplementary Materials:

Figure S37. Text is too small. Please resize for readability.

Figure S41. For the XRD figure, please consider labelling the individual peaks. E.g. (001), (011), PbI2, etc.

Figure S62. Text is too small and colour choice makes readability difficult. Please consider improving this.

We thank the reviewer for highlighting these figures. We have revised all affected panels to improve readability, including clearer labeling and enhanced visual contrast where appropriate. In addition, the proposed diffraction peaks in the XRD figure are now explicitly identified to facilitate interpretation.

After careful consideration, the revised manuscript is suitable for publication in Nature pending minor revisions. The authors have convincingly addressed the major points raised in the initial review, particularly through independent third-party certification above 30% (including steady-state values) and through expanded experimental and mechanistic analysis supporting the wide-bandgap additive strategy, the three-step middle-bandgap absorber approach, and the optical middle reflector.

The remaining comments primarily concern clarity and presentation. In particular, the manuscript would benefit from clearer performance reporting, more explicit distinction between general mechanisms and device-specific optimizations, and some general (big picture) discussion of reproducibility, trade-offs, and stability. Addressing these points should be straightforward and would further strengthen the manuscript.

We appreciate the referee’s overall assessment that the manuscript is suitable for publication pending these minor revisions. We have aimed to address the remaining clarity and presentation points thoroughly, particularly by making performance reporting unambiguous, clearly separating transferable mechanisms from device-specific choices, and improving the readability and interpretability of key figures and Supplementary material.
